# Universal DNA methylation age across mammalian tissues

**DOI:** 10.1038/s43587-023-00462-6

**Published:** 2023-08-10

**Authors:** A. T. Lu, Z. Fei, A. Haghani, T. R. Robeck, J. A. Zoller, C. Z. Li, R. Lowe, Q. Yan, J. Zhang, H. Vu, J. Ablaeva, V. A. Acosta-Rodriguez, D. M. Adams, J. Almunia, A. Aloysius, R. Ardehali, A. Arneson, C. S. Baker, G. Banks, K. Belov, N. C. Bennett, P. Black, D. T. Blumstein, E. K. Bors, C. E. Breeze, R. T. Brooke, J. L. Brown, G. G. Carter, A. Caulton, J. M. Cavin, L. Chakrabarti, I. Chatzistamou, H. Chen, K. Cheng, P. Chiavellini, O. W. Choi, S. M. Clarke, L. N. Cooper, M. L. Cossette, J. Day, J. DeYoung, S. DiRocco, C. Dold, E. E. Ehmke, C. K. Emmons, S. Emmrich, E. Erbay, C. Erlacher-Reid, C. G. Faulkes, S. H. Ferguson, C. J. Finno, J. E. Flower, J. M. Gaillard, E. Garde, L. Gerber, V. N. Gladyshev, V. Gorbunova, R. G. Goya, M. J. Grant, C. B. Green, E. N. Hales, M. B. Hanson, D. W. Hart, M. Haulena, K. Herrick, A. N. Hogan, C. J. Hogg, T. A. Hore, T. Huang, J. C. Izpisua Belmonte, A. J. Jasinska, G. Jones, E. Jourdain, O. Kashpur, H. Katcher, E. Katsumata, V. Kaza, H. Kiaris, M. S. Kobor, P. Kordowitzki, W. R. Koski, M. Krützen, S. B. Kwon, B. Larison, S. G. Lee, M. Lehmann, J. F. Lemaitre, A. J. Levine, C. Li, X. Li, A. R. Lim, D. T. S. Lin, D. M. Lindemann, T. J. Little, N. Macoretta, D. Maddox, C. O. Matkin, J. A. Mattison, M. McClure, J. Mergl, J. J. Meudt, G. A. Montano, K. Mozhui, J. Munshi-South, A. Naderi, M. Nagy, P. Narayan, P. W. Nathanielsz, N. B. Nguyen, C. Niehrs, J. K. O’Brien, P. O’Tierney Ginn, D. T. Odom, A. G. Ophir, S. Osborn, E. A. Ostrander, K. M. Parsons, K. C. Paul, M. Pellegrini, K. J. Peters, A. B. Pedersen, J. L. Petersen, D. W. Pietersen, G. M. Pinho, J. Plassais, J. R. Poganik, N. A. Prado, P. Reddy, B. Rey, B. R. Ritz, J. Robbins, M. Rodriguez, J. Russell, E. Rydkina, L. L. Sailer, A. B. Salmon, A. Sanghavi, K. M. Schachtschneider, D. Schmitt, T. Schmitt, L. Schomacher, L. B. Schook, K. E. Sears, A. W. Seifert, A. Seluanov, A. B. A. Shafer, D. Shanmuganayagam, A. V. Shindyapina, M. Simmons, K. Singh, I. Sinha, J. Slone, R. G. Snell, E. Soltanmaohammadi, M. L. Spangler, M. C. Spriggs, L. Staggs, N. Stedman, K. J. Steinman, D. T. Stewart, V. J. Sugrue, B. Szladovits, J. S. Takahashi, M. Takasugi, E. C. Teeling, M. J. Thompson, B. Van Bonn, S. C. Vernes, D. Villar, H. V. Vinters, M. C. Wallingford, N. Wang, R. K. Wayne, G. S. Wilkinson, C. K. Williams, R. W. Williams, X. W. Yang, M. Yao, B. G. Young, B. Zhang, Z. Zhang, P. Zhao, Y. Zhao, W. Zhou, J. Zimmermann, J. Ernst, K. Raj, S. Horvath

**Affiliations:** 1 Department of Human Genetics, David Geffen School of Medicine, University of California, Los Angeles, Los Angeles, CA USA; 2 Altos Labs, San Diego Institute of Science, San Diego, CA USA; 3 Department of Biostatistics, Fielding School of Public Health, University of California, Los Angeles, Los Angeles, CA USA; 4 Department of Statistics, University of California, Riverside, Riverside, CA USA; 5 Zoological SeaWorld Parks and Entertainment, Orlando, FL USA; 6 Altos Labs, Cambridge Institute of Science, Cambridge, UK; 7 Bioinformatics Interdepartmental Program, University of California, Los Angeles, CA USA; 8 Department of Biological Chemistry, University of California, Los Angeles, Los Angeles, CA USA; 9 Department of Biology, University of Rochester, Rochester, NY USA; 10 Department of Neuroscience, Peter O’Donnell Jr. Brain Institute, University of Texas Southwestern Medical Center, Dallas, TX USA; 11 Department of Biology, University of Maryland, College Park, MD USA; 12 Loro Parque Fundacion, Puerto de la Cruz, Spain; 13 Department of Biology, University of Kentucky, Lexington, KY USA; 14 Division of Cardiology, Department of Internal Medicine, David Geffen School of Medicine, University of California, Los Angeles, Los Angeles, CA USA; 15 Marine Mammal Institute, Oregon State University, Newport, OR USA; 16 School of Science and Technology, Clifton Campus, Nottingham Trent University, Nottingham, UK; 17 School of Life and Environmental Sciences, the University of Sydney, Sydney, New South Wales Australia; 18 Department of Zoology and Entomology, University of Pretoria, Hatfield, South Africa; 19 Busch Gardens Tampa, Tampa, FL USA; 20 Department of Ecology and Evolutionary Biology, University of California, Los Angeles, Los Angeles, CA USA; 21 Rocky Mountain Biological Laboratory, Crested Butte, CO USA; 22 Altius Institute for Biomedical Sciences, Seattle, WA USA; 23 Epigenetic Clock Development Foundation, Los Angeles, CA USA; 24 Center for Species Survival, Smithsonian Conservation Biology Institute, Front Royal, VA USA; 25 Department of Evolution, Ecology and Organismal Biology, The Ohio State University, Columbus, OH USA; 26 AgResearch, Invermay Agricultural Centre, Mosgiel, New Zealand; 27 Department of Biochemistry, University of Otago, Dunedin, New Zealand; 28 Gulf World, Dolphin Company, Panama City Beach, FL USA; 29 School of Veterinary Medicine and Science, University of Nottingham, Nottingham, UK; 30 Department of Pathology, Microbiology and Immunology, School of Medicine, University of South Carolina, Columbia, SC USA; 31 Department of Pharmacology, Addiction Science and Toxicology, the University of Tennessee Health Science Center, Memphis, TN USA; 32 Medical Informatics, David Geffen School of Medicine, University of California, Los Angeles, Los Angeles, CA USA; 33 Biochemistry Research Institute of La Plata, Histology and Pathology, School of Medicine, University of La Plata, La Plata, Argentina; 34 Center for Neurobehavioral Genetics, Semel Institute for Neuroscience and Human Behavior, Department of Psychiatry and Biobehavioral Sciences, David Geffen School of Medicine, University of California, Los Angeles, Los Angeles, CA USA; 35 Department of Anatomy and Neurobiology, Northeast Ohio Medical University, Rootstown, OH USA; 36 Department of Environmental and Life Sciences, Trent University, Peterborough, Ontario Canada; 37 Taronga Institute of Science and Learning, Taronga Conservation Society Australia, Mosman, New South Wales Australia; 38 SeaWorld of Florida, Orlando, FL USA; 39 Zoological Operations, SeaWorld Parks and Entertainment, Orlando, FL USA; 40 Duke Lemur Center, Durham, NC USA; 41 Conservation Biology Division, Northwest Fisheries Science Center, National Marine Fisheries Service, National Oceanic and Atmospheric Administration, Seattle, WA USA; 42 Departments of Biology and Medicine, University of Rochester, Rochester, NY USA; 43 Altos Labs, San Francisco, CA USA; 44 SeaWorld Orlando, Orlando, FL USA; 45 School of Biological and Behavioural Sciences, Queen Mary University of London, London, UK; 46 Fisheries and Oceans Canada, Freshwater Institute, Winnipeg, Manitoba Canada; 47 Department of Biological Sciences, University of Manitoba, Winnipeg, Manitoba Canada; 48 Department of Population Health and Reproduction, University of California, Davis School of Veterinary Medicine, Davis, CA USA; 49 Mystic Aquarium, Mystic, CT USA; 50 Universite de Lyon, Universite Lyon 1, CNRS, Laboratoire de Biometrie et Biologie Evolutive, Villeurbanne, France; 51 Greenland Institute of Natural Resources, Nuuk, Greenland; 52 Evolution and Ecology Research Centre, School of Biological, Earth and Environmental Sciences, UNSW Sydney, Sydney, New South Wales Australia; 53 Division of Genetics, Department of Medicine, Brigham and Women’s Hospital, Harvard Medical School, Boston, MA USA; 54 Applied Translational Genetics Group, School of Biological Sciences, Centre for Brain Research, the University of Auckland, Auckland, New Zealand; 55 Vancouver Aquarium, Vancouver, British Columbia Canada; 56 SeaWorld of California, San Diego, CA USA; 57 Cancer Genetics and Comparative Genomics Branch, National Human Genome Research Institute, National Institutes of Health, Bethesda, MD USA; 58 Department of Anatomy, University of Otago, Dunedin, New Zealand; 59 Division of Human Genetics, Department of Pediatrics, University at Buffalo, Buffalo, NY USA; 60 Division of Genetics and Metabolism, Oishei Children’s Hospital, Buffalo, NY USA; 61 School of Biological Sciences, University of Bristol, Bristol, UK; 62 Norwegian Orca Survey, Andenes, Norway; 63 Mother Infant Research Institute, Tufts Medical Center, Boston, MA USA; 64 Yuvan Research, Mountain View, CA USA; 65 Kamogawa Sea World, Kamogawa, Japan; 66 Peromyscus Genetic Stock Center, University of South Carolina, Columbia, SC USA; 67 Department of Drug Discovery and Biomedical Sciences, College of Pharmacy, University of South Carolina, Columbia, SC USA; 68 Edwin S.H. Leong Healthy Aging Program, Centre for Molecular Medicine and Therapeutics, University of British Columbia, Vancouver, British Columbia Canada; 69 Institute of Animal Reproduction and Food Research of the Polish Academy of Sciences, Olsztyn, Poland; 70 Institute for Veterinary Medicine, Nicolaus Copernicus University, Torun, Poland; 71 LGL Limited, King City, Ontario Canada; 72 Evolutionary Genetics Group, Department of Evolutionary Anthropology, University of Zurich, Zurich, Switzerland; 73 Department of Ecology and Evolutionary Biology, UCLA, Los Angeles, CA USA; 74 Center for Tropical Research, Institute for the Environment and Sustainability, UCLA, Los Angeles, CA USA; 75 Department of Neurology, David Geffen School of Medicine, University of California, Los Angeles, Los Angeles, CA USA; 76 Texas Pregnancy and Life-course Health Center, Southwest National Primate Research Center, San Antonio, TX USA; 77 Department of Animal Science, College of Agriculture and Natural Resources, Laramie, WY USA; 78 Technology Center for Genomics and Bioinformatics, Department of Pathology and Laboratory Medicine, University of California, Los Angeles, Los Angeles, CA USA; 79 Centre for Molecular Medicine and Therapeutics, BC Children’s Hospital Research Institute, University of British Columbia, Vancouver, British Columbia Canada; 80 Institute of Ecology and Evolution, School of Biological Sciences, University of Edinburgh, Edinburgh, UK; 81 White Oak Conservation, Yulee, FL USA; 82 North Gulf Oceanic Society, Homer, AK USA; 83 Translational Gerontology Branch, National Institute on Aging Intramural Research Program, National Institutes of Health, Baltimore, MD USA; 84 ABS Global, DeForest, WI USA; 85 Marineland of Canada, Niagara Falls, Ontario Canada; 86 Biomedical and Genomic Research Group, Department of Animal and Dairy Sciences, University of Wisconsin—Madison, Madison, WI USA; 87 Department of Preventive Medicine, University of Tennessee Health Science Center, College of Medicine, Memphis, TN USA; 88 Department of Genetics, Genomics and Informatics, University of Tennessee Health Science Center, College of Medicine, Memphis, TN USA; 89 Louis Calder Center—Biological Field Station, Department of Biological Sciences, Fordham University, Armonk, NY USA; 90 Museum fur Naturkunde, Leibniz Institute for Evolution and Biodiversity Science, Berlin, Germany; 91 Institute of Molecular Biology, Mainz, Germany; 92 Division of Molecular Embryology, DKFZ-ZMBH Alliance, Heidelberg, Germany; 93 Department of Obstetrics and Gynecology, Tufts University School of Medicine, Boston, MA USA; 94 Cancer Research UK Cambridge Institute, University of Cambridge, Cambridge, UK; 95 Division of Regulatory Genomics and Cancer Evolution, Deutsches Krebsforschungszentrum, Heidelberg, Germany; 96 Department of Psychology, Cornell University, Ithaca, NY USA; 97 SeaWorld of Texas, San Antonio, TX USA; 98 Department of Molecular Cell and Developmental Biology, University of California, Los Angeles, Los Angeles, CA USA; 99 School of Earth, Atmospheric and Life Sciences, University of Wollongong, Wollongong, Australia; 100 Institute of Evolutionary Biology, School of Biological Sciences, University of Edinburgh, Edinburgh, UK; 101 Department of Animal Science, University of Nebraska, Lincoln, NE USA; 102 Mammal Research Institute, Department of Zoology and Entomology, University of Pretoria, Hatfield, South Africa; 103 Department of Biology, College of Arts and Science, Adelphi University, Garden City, NY USA; 104 Salk Institute for Biological Studies, La Jolla, CA USA; 105 Department of Epidemiology, UCLA Fielding School of Public Health, Los Angeles, CA USA; 106 Department of Environmental Health Sciences, UCLA Fielding School of Public Health, Los Angeles, CA USA; 107 Department of Neurology, UCLA David Geffen School of Medicine, Los Angeles, CA USA; 108 Center for Coastal Studies, Provincetown, MA USA; 109 Miami Seaquarium, Miami, FL USA; 110 The Sam and Ann Barshop Institute for Longevity and Aging Studies and Department of Molecular Medicine, UT Health San Antonio and the Geriatric Research Education and Clinical Center, South Texas Veterans Healthcare System, San Antonio, TX USA; 111 Department of Radiology, University of Illinois at Chicago, Chicago, IL USA; 112 Department of Biochemistry and Molecular Genetics, University of Illinois at Chicago, Chicago, IL USA; 113 National Center for Supercomputing Applications, University of Illinois at Urbana-Champaign, Urbana, IL USA; 114 College of Agriculture, Missouri State University, Springfield, MO USA; 115 Department of Animal Sciences, University of Illinois at Urbana-Champaign, Champaign, IL USA; 116 Department of Forensic Science, Environmental and Life Sciences, Trent University, Peterborough, Ontario Canada; 117 Department of Surgery, University of Wisconsin School of Medicine and Public Health, Madison, WI USA; 118 Shobhaben Pratapbhai Patel School of Pharmacy and Technology Management, SVKM’S NMIMS University, Mumbai, India; 119 Species Preservation Laboratory, SeaWorld San Diego, San Diego, CA USA; 120 Biology Department, Acadia University, Wolfville, Nova Scotia Canada; 121 Department of Pathobiology and Population Sciences, Royal Veterinary College, Hatfield, UK; 122 Howard Hughes Medical Institute, Department of Neuroscience, University of Texas Southwestern Medical Center, Dallas, TX USA; 123 School of Biology and Environmental Science, University College Dublin, Dublin, Ireland; 124 John G. Shedd Aquarium, Chicago, IL USA; 125 School of Biology, the University of St Andrews, Fife, UK; 126 Neurogenetics of Vocal Communication Group, Max Planck Institute for Psycholinguistics, Nijmegen, the Netherlands; 127 Blizard Institute, Faculty of Medicine and Dentistry, Queen Mary University of London, London, UK; 128 Department of Pathology and Laboratory Medicine, David Geffen School of Medicine at UCLA, Los Angeles, CA USA; 129 Division of Obstetrics and Gynecology, Tufts University School of Medicine, Boston, MA USA; 130 Center for Neurobehavioral Genetics, Jane and Terry Semel Institute for Neuroscience and Human Behavior, University of California, Los Angeles, Los Angeles, CA USA; 131 Department of Psychiatry and Biobehavioral Sciences, David Geffen School of Medicine at UCLA, Los Angeles, CA USA; 132 Fisheries and Oceans Canada, Winnipeg, Manitoba Canada; 133 Eli and Edythe Broad Center of Regenerative Medicine and Stem Cell Research, University of California, Los Angeles, CA USA; 134 Center for Computational and Genomic Medicine, Children’s Hospital of Philadelphia, Philadelphia, PA USA; 135 Department of Pathology and Laboratory Medicine, University of Pennsylvania, Philadelphia, PA USA; 136 Department of Mathematics and Technology, University of Applied Sciences Koblenz, Koblenz, Germany

**Keywords:** Evolution, Ageing, DNA methylation, Biomarkers

## Abstract

Aging, often considered a result of random cellular damage, can be accurately estimated using DNA methylation profiles, the foundation of pan-tissue epigenetic clocks. Here, we demonstrate the development of universal pan-mammalian clocks, using 11,754 methylation arrays from our Mammalian Methylation Consortium, which encompass 59 tissue types across 185 mammalian species. These predictive models estimate mammalian tissue age with high accuracy (*r* > 0.96). Age deviations correlate with human mortality risk, mouse somatotropic axis mutations and caloric restriction. We identified specific cytosines with methylation levels that change with age across numerous species. These sites, highly enriched in polycomb repressive complex 2-binding locations, are near genes implicated in mammalian development, cancer, obesity and longevity. Our findings offer new evidence suggesting that aging is evolutionarily conserved and intertwined with developmental processes across all mammals.

## Abstract (web summary)

This study identifies and characterizes evolutionarily conserved cytosine methylation patterns related to age across mammals and establishes pan-mammalian epigenetic clocks.

## Main

Aging is associated with multiple cellular changes that are often tissue specific^1^. Cytosine methylation, however, stands out, as it allows for the development of pan-tissue aging clocks (multivariate age estimators) that are applicable to all human tissues^2–4^. The subsequent development of similar pan-tissue clocks for mice and other species suggests a conserved aspect to the aging process^5–7^, thereby challenging the belief that aging is solely driven by random cellular damage accumulated over time. To investigate this, we sought to (1) develop universal age estimators applicable to all mammalian species and tissues (pan-mammalian clocks) and (2) identify and characterize cytosines with methylation levels that change with age across all mammals. For this purpose, we employed the mammalian methylation array, which we recently developed to profile methylation levels of up to 36,000 CpG sites with flanking DNA sequences highly conserved across the mammalian class^8^. We employed such profiles from 11,754 samples from 59 tissue types, originating from 185 mammalian species across 19 taxonomic orders (Supplementary Data 1.1–1.4 and Supplementary Notes 1 and 2) with ages ranging from prenatal to 139 years old (bowhead whale, *Balaena mysticetus*)^9^. These data are a subset from our Mammalian Methylation Consortium, which characterized maximum lifespan^9^. As we were interested in developing pan-mammalian clocks, we restricted the analysis to animals with known ages.

## Results

### Universal pan-mammalian epigenetic clocks

In separate articles, we described the application of the mammalian methylation array to individual mammalian species^10–19^. These studies already demonstrate that one can build dual-species epigenetic age estimators (for example, human–naked mole rat clocks)^10–17^, in contrast to first- and second-generation clocks that measure human age^4,20,21^ and mortality risk^22,23^, respectively. However, it is not yet known whether one can develop a mathematical formula to estimate age in all mammalian species. Here we present three such pan-mammalian age estimators.

**Extended Data Fig. 1:**
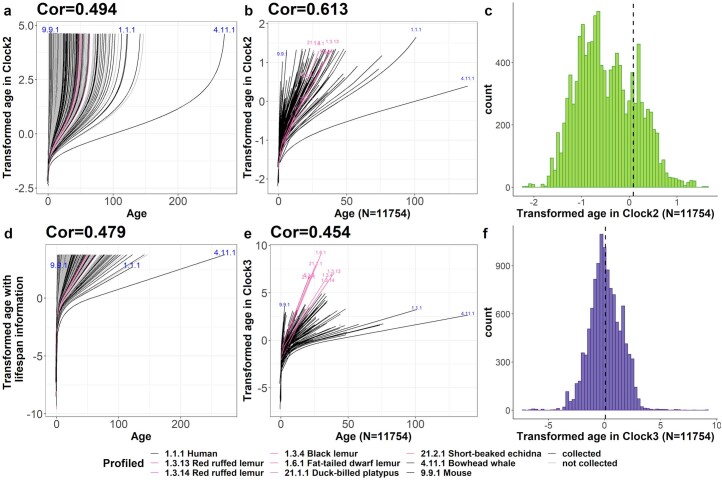
Transformed age in universal clocks. The plot displays transformed age in universal Clock 2 (**a**–**c**) and universal Clock 3 (**d**–**f**). (**a**, **b**) Loglog transformation of Relative Age (y-axis) versus age in universal Clock 2 and (**d**, **e**) log-linear age (y-axis) versus age in our universal Clock 3. Of the 969 mammalian species with available gestation time, age at sexual maturity and maximum lifespan in AnAge database, 339 species are available in our collection. We multiplied the reported maximum lifespan of non-human or non-mouse species by 1.3. Transformed ages were calculated for all the 969 species with simulated age ranging from gestation time through the modified maximum lifespan. The columns (**a**, **d**) display all the 969 species with the simulated ages. In panel d, we proposed the log-linear age with the parameter ${\boldsymbol{m}}$ formulated with maximum lifespan as the information is available for all species (${{\boldsymbol{m}}}^{{\boldsymbol{* }}}{\boldsymbol{=}}{{\boldsymbol{c}}}_{{\boldsymbol{1}}}{\boldsymbol{* }}\frac{{\boldsymbol{MaxLifespan}}{\boldsymbol{+}}{\boldsymbol{GestationT}}}{{\boldsymbol{ASM}}{\boldsymbol{+}}{\boldsymbol{GestationT}}}$ in Methods). Of the 339 species, 185 species with age information of high confidence and known tissue types were used in training universal clocks. The columns (**b**, **e**) empirically display these 185 species with the age variable (x-axis) based on the observed ages from all the samples in our collection (N = 11,754). In panel e, we applied the log-linear age formulated without knowing maximum lifespan to train Clock 3 (formula (5) in Methods). Each line represents a species marked by gray for non-profiled and marked by black or pink for profiled species in our collection, as listed in the legend. Some species such as lemurs with relatively short gestation time in regressing ${{\boldsymbol{m}}}^{{\boldsymbol{* }}}$ (formula (7) in Methods) exhibiting high log-linear ages in (**e**) are marked in pink. Each panel reports the Pearson correlation coefficient. (**c**, **f**) display the histograms of transformed ages based on all samples from the 185 species with vertical lines presenting at means.

The first, basic clock (clock 1), regresses log-transformed chronological age on DNA methylation levels of all available mammals. Although such a clock can directly estimate the age of any mammal, its usefulness could be further increased if its output were adjusted for differences in the maximum lifespan of each species as well, as this would allow biologically meaningful comparisons to be made between species with very different lifespans. To this end, we developed a second universal clock that defines individual age relative to the maximum lifespan of its species; generating relative age estimates between 0 and 1. Because the accuracy of this universal relative age clock (clock 2) could be compromised in species for which knowledge of maximum lifespan is inaccurate, we developed a third universal clock, using age at sexual maturity (ASM) and gestation time instead of maximum lifespan, as these traits are better established and explain over 69% of maximum lifespan variation on the log scale (Supplementary Data 2). This third clock is referred to as the universal log–linear age clock (clock 3). The non-linear mathematical function underlying the age transformation of clock 3 reflects the fact that epigenetic clocks tick faster during development, an observation that led to the establishment of the first pan-tissue clock for humans^4^ (Extended Data Fig. 1a,b,d,e).

### Performance of universal epigenetic clocks across species

**Fig. 1:**
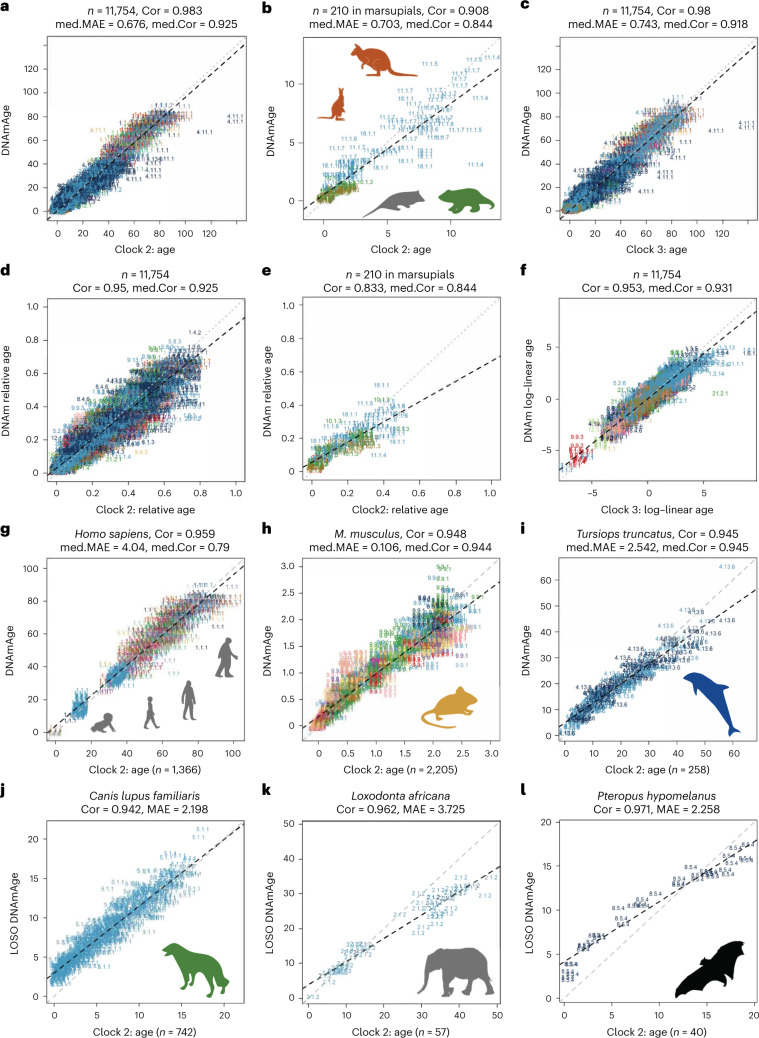
Universal clocks for transformed age across mammals. The figure displays relative age estimates of universal clock 2 (clock 2) and log–linear-transformed age of universal clock 3 (clock 3). Relative age estimates incorporate maximum lifespan and assume values between 0 and 1. Log–linear age is formulated with ASM and gestational time. **a**–**i**, Age estimated by LOFO cross-validation for clock 2 and clock 3. **j**–**l**, Age estimated via LOSO cross-validation for clock 2. The DNAm estimates of age (*y* axes) of **a**–**c** are transformations of relative age (clock 2) or log–linear age (clock 3) into units of years. **b**,**e**, Only marsupials (nine species). Each panel reports a Pearson correlation (Cor) coefficient. The gray and black dashed lines correspond to the diagonal line (*y*=*x*) and the regression line, respectively. Median correlation (med.Cor) and median of MAE (med.MAE) are calculated across species (**a**–**f**) or across species–tissue (**g**–**l**). All correlation *P* values are highly significant (*P* < 1.0 × 10^−22^). Each sample is labeled by mammalian species index and indicated by tissue color (Supplementary Data 1.3–1.4). All *P* values reported are unadjusted and two sided.

**Fig. 2:**
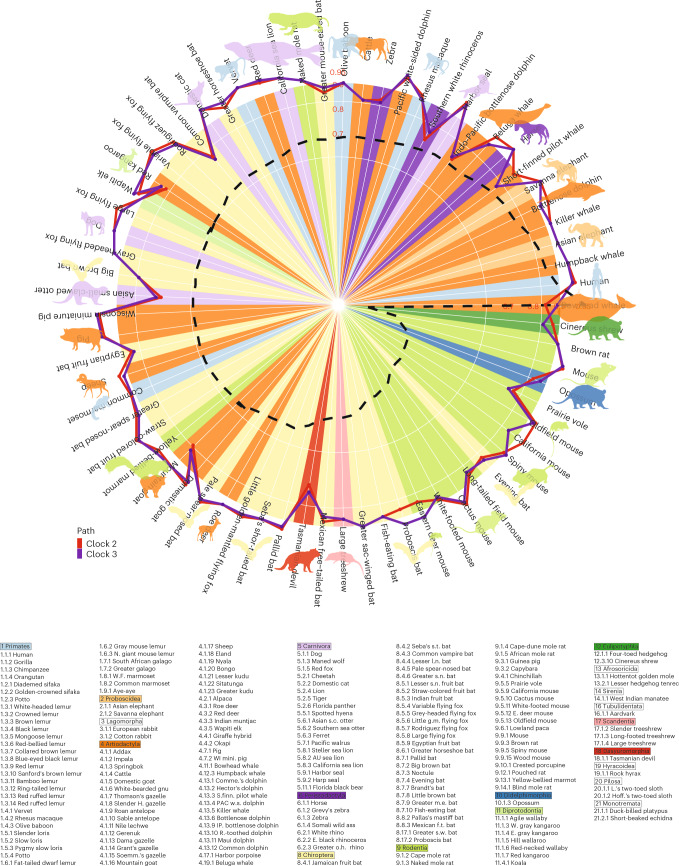
Accuracy of universal clocks are independent of species lifespan. The circle plot displays Pearson correlation between age and DNAmAge estimated by universal clocks 2 (clock 2) and 3 (clock 3) for various species. Of the 185 species, correlation analysis was performed on 69 species (with *n* ≥ 15 in a single tissue) across 12 taxonomic orders. We took log transformation of maximum lifespans of species and divided them by log (211), which is the maximum lifespan of bowhead whales. Values of the resulting ratios ranged from 0.12 (cinereus shrew) to 1 (bowhead whales). These ratios are displayed in descending order in the circle plot marked by the black dashed line, starting with the bowhead whale (1) and human (0.90) and ending with the cinereus shrew (0.12), in counterclockwise direction. In the background, circumferences with increasing radii represent increasing correlation levels up to 0.9. These correlations between age and DNAmAge were estimated by clock 2 (red path line) and clock 3 (purple path line) for each species. Colors within the circle represent the taxonomic order of the corresponding species, as listed below the circle. The median of correlation across species is 0.926 for clock 2 and 0.918 for clock 3. Straw-colored fruit bats exhibit the highest correlation (*r* = 0.985) based on clock 2, and Wisconsin miniature pigs have the second highest correlation (*r* = 0.984) based on clock 3. A majority of species with their circle lines located outside the background indicates that their correlation estimates are greater than 0.9. The text at the bottom lists the 185 species under their corresponding taxonomic order. Each taxonomic order is marked by the same color matching with the circle plot. The numbers after the first and second decimal points enumerate the taxonomic family and species, respectively. AU, Australian; Comme., Commerson’s; E., eastern; f.t., free-tailed; g.m., golden-mantled; H. (gazelle), Horn gazelle; Hoff., Hoffman’s; IP, Indo-Pacific; L.’s, Linne’s; l.n., long-nosed; m.e., mouse-eared; mini., miniature; N., northern; o.h., one horned; s.c., small-clawed; PAC w.s., Pacific white-sided; R.-toothed, Rough-toothed; Soemm., Soemmerring’s; S.finn., Short-finned; s.n., short nosed; s.t., short-tailed; s.w., sac-winged; W. western; W.F., White-fronted; WI mini., Wisconsin miniature.

**Extended Data Fig. 2:**
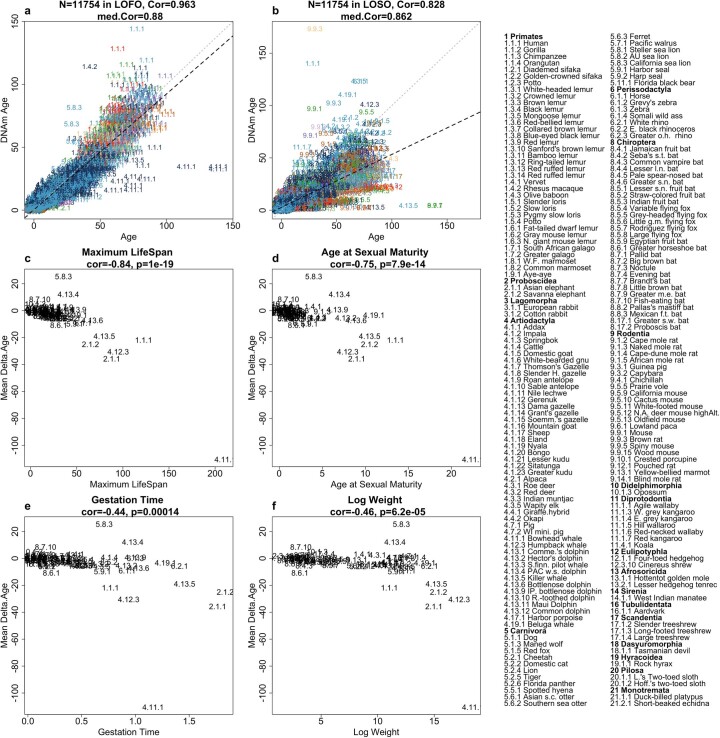
Basic universal clock for log-transformed age. **a**, **b**, Chronological age (x-axis) versus DNAmAge estimated using **a**, leave-one-fraction-out (LOFO) and **b**, leave-one-species-out (LOSO) analysis. The gray and black dashed lines correspond to the diagonal line (y = x) and the regression line, respectively. Each sample is labeled by the mammalian species index (legend). The species index corresponds to the taxonomic order, for example 1 = primates, 2 = elephants (Proboscidea) etc. (legend). The numbers after the first and second decimal points enumerate the taxonomic family and species, respectively. Points are colored by tissue type (Supplementary Data 1.4). The heading of each panel reports the Pearson correlation (cor) across all samples. Here med.Cor denotes the median value across species that contain at least 15 samples. **c**–**f**, The y-axis reports the mean difference between the LOSO estimate of DNAm age and chronological age evaluated at a fixed age defined as half the maximum lifespan (denoted as Mean Delta.Age). The scatter plots depict mean delta half lifespan per species (y-axis) versus c, maximum lifespan observed in the species, d, average age at sexual maturity e, gestational time (in units of years), and f, (log-transformed) average adult body mass in units of grams. All P-values reported are unadjusted and are based on two-sided tests.

To evaluate the clocks’ accuracy, we employed leave-one-fraction-out (LOFO) and leave-one-species-out (LOSO) cross-validation analyses. Each analysis divides the dataset differently for validation: LOFO into ten fractions with similar proportions of species and tissue types; LOSO excludes one species per iteration. The final models of the clocks use less than 1,000 CpG sites each (Supplementary Data 3.1–3.3), with 401 common genes proximal to CpG sites in both clock 2 and clock 3 (Supplementary Data 3.5). LOFO cross-validation reveals the universal clocks as highly accurate estimators of chronological age (*r* ≈ 0.96–0.98) with a median absolute error (MAE) of <1 year between chronological age and DNA methylation (DNAm)-based age estimate (DNAmAge) and a relative error of <3.3% (Figs. 1a,c and 2, Extended Data Fig. 2a, Supplementary Table 1 and Supplementary Data 4.1–4.3). Despite the mammalian array mapping fewer CpG sites to marsupials^8^, clocks 2 and 3 maintain their accuracy when analysis is confined to marsupials (for example, *r* = 0.91, median MAE < 0.80 year for clock 2; Fig. 1b). Moreover, our monotreme study (*n* = 15) produced encouraging results (for example, *r* = 0.85 for clock 2; Supplementary Data 4.1).

Using LOSO cross-validation, the clocks displayed age correlations as high as *r* = 0.941 (Supplementary Table 1), suggesting their applicability to species not included in the training set. However, for certain species, such as bowhead whales, the basic clock’s predicted epigenetic age poorly aligns with chronological age (Extended Data Fig. 2a).

For the basic clock 1, the mean discrepancy between LOSO DNAmAge and chronological age (Delta.Age) is negatively correlated with species maximum lifespan (*r* = −0.84, *P* = 1.0 × 10^−19^) and ASM (*r* = −0.75, *P* = 7.9 × 10^−14^; Extended Data Fig. 2c,d). Here, the strengths of clocks 2 and 3 come to fore as they adjust for these species characteristics during their construction (Extended Data Fig. 1).

Universal clocks 2 and 3, arguably more biologically meaningful than clock 1, achieve a correlation of *r* ≥ 0.95 between DNAm transformed age and observed transformed age, respectively (Fig. 1d,f). We will focus on them in the following text. They are pan-tissue clocks offering comparable accuracy in LOFO estimates across numerous tissue types (Fig. 1 and Supplementary Data 4.2). For instance, clock 2 yielded high age correlations in humans (LOFO estimate of *r* = 0.959 across 20 tissue types), mice (*r* = 0.948, 26 tissues) and bottlenose dolphins (*r* = 0.945, two tissues). Fig. 2 displays circle plots for the age correlation estimates in different species sorted by maximum lifespan.

Visual inspection indicates no relationship between age correlation from clocks 2 and 3 and maximum lifespan (dashed line, Fig. 2, circle). While accurately predicting age for the humpback whale and other mammals, the clocks sometimes underestimated bowhead whale reported age (mammalian species index 4.11.1 in Fig. 1a,c), possibly due to overestimation of older whales’ ages by aspartic acid racemization.

Clocks 2 and 3 provide similarly accurate LOSO age estimates between evolutionarily distant species (Supplementary Data 5.2), including dogs (*n* = 742, 93 breeds, *r* = 0.94, MAE < 2.28 years), African elephants (*r* = 0.96, MAE < 4.0 years) and flying foxes (*r* = 0.97, MAE < 2.3 years) (Fig. 1j–l). Such accuracy demonstrates these clocks’ broad relevance, tapping into conserved age-related mechanisms across mammals, including species not in the training data (Supplementary Data 5.1–5.2).

**Extended Data Fig. 3:**
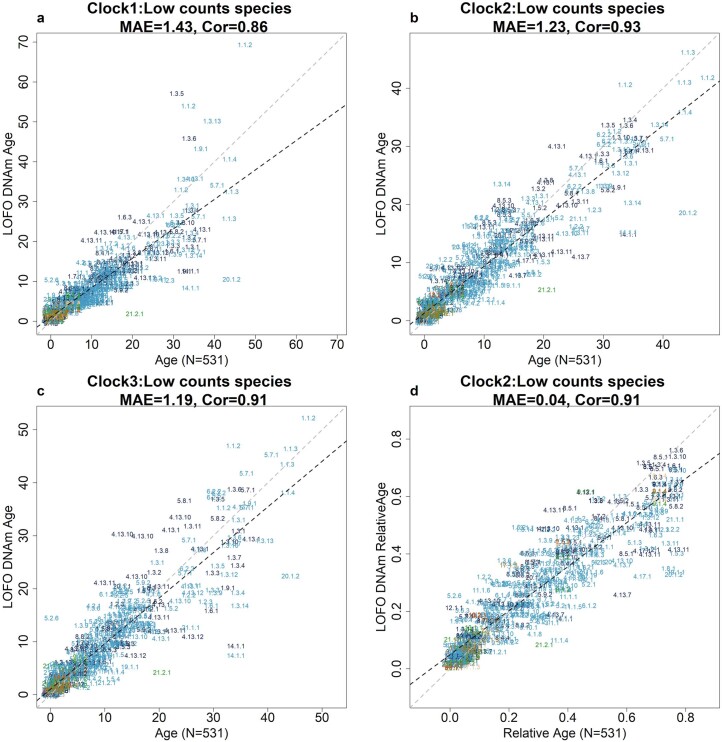
Universal clocks applied to species with fewer than 15 samples. The title of each panel lists the type of universal clock: **a**, Clock 1 = basic universal clock based on log(Age + 2), b, d, Clock 2 = universal clock for relative age, c, Clock 3 =universal clock for log-linear age. Leave-one-fraction-out (LOFO) methylation estimates versus a–c, chronological age or d, relative age for clock 2. The respective inverse transformations were applied to arrive at DNA methylation-based estimates of chronological age in years or relative age (y-axis).

The three universal clocks performed well for 114 species with fewer than 15 samples each (*r* ≈ 0.90, MAE ≈ 1.2 years for clocks 1–3; Extended Data Fig. 3a–c), exhibiting strong correlation for relative age (*r* = 0.91 for clock 2; Extended Data Fig. 3d).

### Pan-mammalian universal clocks across tissues

The significantly distinct epigenomic landscape across tissue types^24,25^ prompted an assessment of these clocks’ performance in different tissues. We assessed the tissue-specific accuracy of clock 2 for estimating relative age (*r* = 0.95, Fig. 1d) across 33 distinct tissue types, observing a median correlation of 0.91 and a median MAE for relative age of 0.027 (Supplementary Data 4.3). High age correlation was consistently observed in brain regions: whole brain (*r* = 0.991), cerebellum (*r* = 0.963), cortex (*r* = 0.957), hippocampus (*r* = 0.954) and striatum (*r* = 0.935; Extended Data Fig. 5a,d,f,g,i and Supplementary Data 4.3) as well as in organs: spleen (*r* = 0.982), liver (*r* = 0.963) and kidney (*r* = 0.963; Extended Data Fig. 5b,c,e). Blood and skin also showed high estimates of relative age correlations across different species: blood (*r* = 0.952, MAE = 0.022, 124 species) and skin (*r* = 0.942, MAE = 0.027, 92 species; Extended Data Fig. 5h,k).

### Tissue-specific pan-mammalian clocks

**Extended Data Fig. 4:**
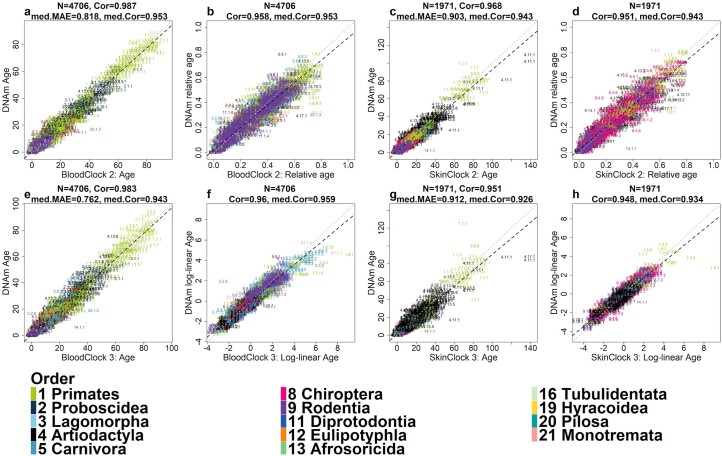
Universal clocks for specific tissues (blood, skin). These tissue-specific universal clocks were constructed in an analogous fashion to the pan-tissue clocks described in the main text. The panels show leave-one-fraction-out (LOFO) estimates (y-axis) of four clocks: universal blood clock 2 (Universal BloodClock 2) which estimates relative age, universal blood clock 3 (Universal BloodClock 3) which estimates log-linear transformation of age. Analogously, we defined Universal SkinClock2 and Universal SkinClock3. Relative age estimation incorporates maximum lifespan and gestational age and assumes values between 0 and 1. Log-linear age is formulated with age at sexual maturity and gestational time. **a**, **c**, **e**, **g**, LOFO estimates of DNAm age (y-axis, in units of years) based on transforming relative age (Clock 2) or log-linear age (Clock 3). **b**, **f**, **d**, **h**, transformed age (x-axis) versus corresponding DNAm estimates (y-axis). The title of each panel reports the Pearson correlation coefficient across all data points and the median correlation (med.Cor) and median of median absolute error (med.MAE) across all species. Each sample is labeled by mammalian species index (explained in Fig. 2) and colored by taxonomic order. The legend reports the taxonomic order and the mammalian order index as a prefix.

**Extended Data Fig. 5:**
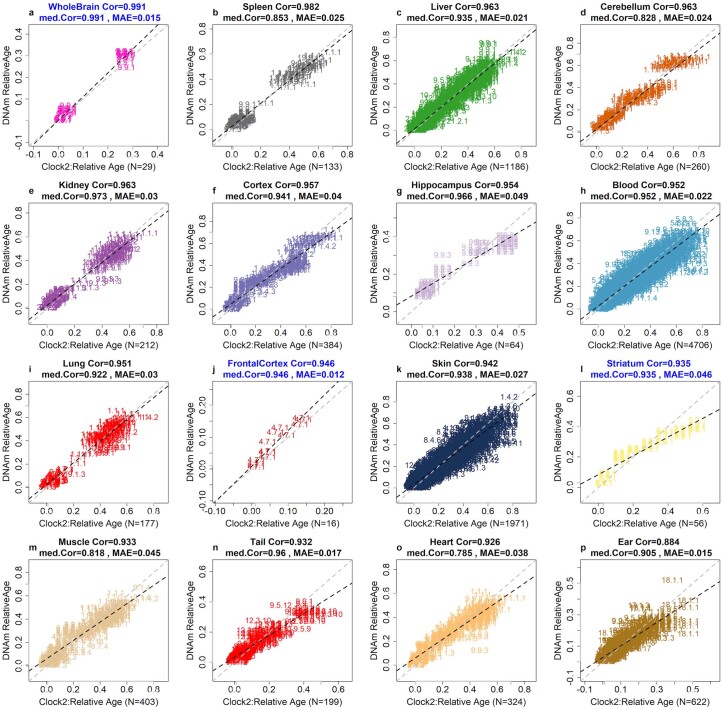
Universal clock for relative age applied to specific tissues. **a**–**p**, DNA methylation-based estimates of relative age (y-axis) versus actual relative age (x-axis). The specific tissue or cell type is reported in the title of each panel. Each sample is labeled by mammalian species index and colored by tissue type (Supplementary Data 1.3–1.4). The analysis is restricted to tissues that have at least 15 samples available. Leave-one-fraction-out cross-validation (LOFO) was used to arrive at unbiased estimates of predictive accuracy measures: median absolute error (MAE) and age correlation based on relative age. ‘Cor’ denotes the Pearson correlation coefficient based on all available samples. ‘med.Cor’ denotes the median values across all species for which at least 15 samples were available. Title is marked in blue if a tissue type was collected from a single species.

The universal pan-mammalian clocks, derived from multiple tissue types, are essentially pan-tissue clocks. We also constructed analogous clocks solely based on blood (Universal BloodClock 2 and Universal BloodClock 3) and skin (Universal SkinClock 2 and Universal SkinClock 3), the tissues most readily accessible across all species. These tissue-specific clocks tend to demonstrate slightly higher accuracy than the pan-tissue clocks when analyzing their respective tissues. Both the blood and skin clocks exhibit robust age correlations (*r* ≈ 0.983–0.987 for blood and *r* ≈ 0.951–0.968 for skin; Extended Data Fig. 4c,g).

### Human mortality risk, clinical biomarkers and lifestyle factors

**Fig. 3:**
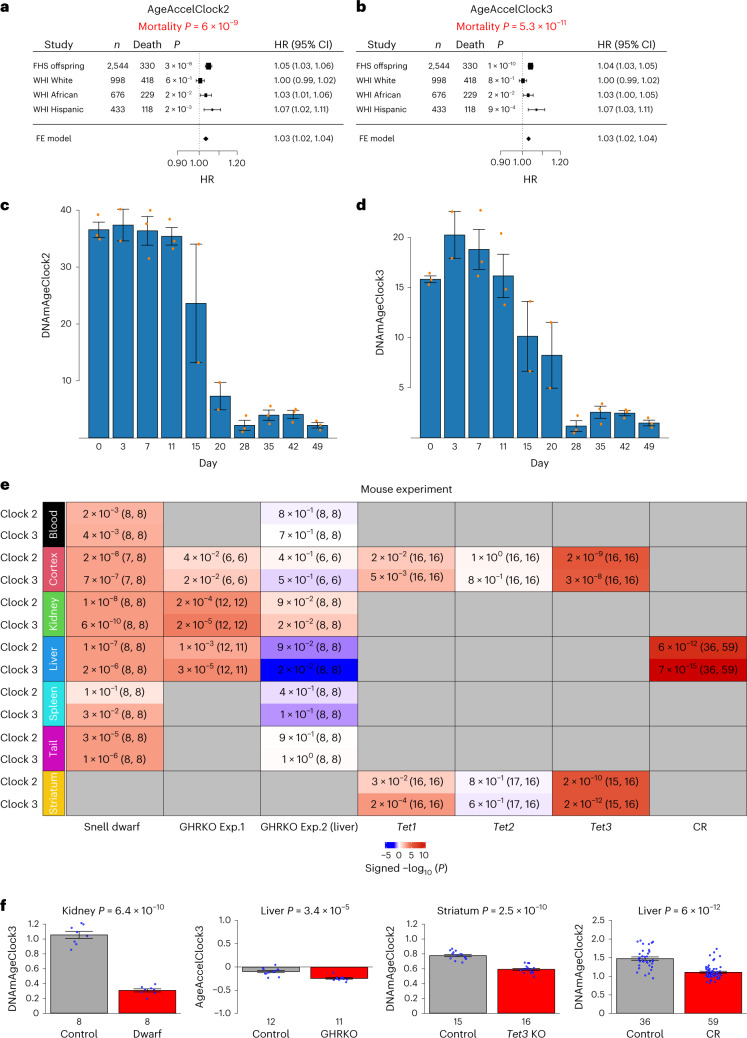
Applications of universal pan-mammalian clocks in human cohorts, reprogramming experiment and murine anti-aging studies. **a**,**b**, Forest plots representing the fixed effect (FE) model meta-analysis, combining HRs from Cox regression models for time to death, based on epigenetic age acceleration measures of clock 2 (AgeAccelClock2) and clock 3 (AgeAccelClock3) across different ethnic groups within two epidemiological cohorts. Each row indicates an HR for a 1-year increase in the age acceleration (AgeAccel) measure, along with a 95% confidence interval (CI). **c**,**d**, DNAmAge estimates of human dermal fibroblasts during OSKM-induced reprogramming. The *y* axes are DNAmAge estimates of clock 2 and clock 3 at day 0, 3, …, 42 and 49, respectively, during reprogramming^31^. **e**, Evaluations of mouse anti-age interventions: (1) age-matched Snell dwarf mutation study: 48 normal and 47 dwarf mice with ages of approximately 0.52 (mean ± s.d. = 0.52 ± 0.01) years, (2) age-matched whole-body GHRKO experiment 1 (Exp.1) with 36 normal and 35 GHRKO mice (mean ± s.d. = 0.65 ± 0.06 years), (3) age-matched GHRKO experiment 2 with GHRKO in livers only with 48 normal and 48 GHRKO genotypes (mean ± s.d. = 0.51 ± 0.03 years old), (4) Tet gene-KO study with all samples at age 0.5 years (*Tet1*, 32 controls and 32 *Tet1* KO; *Tet2*, 33 controls and 32 *Tet2* KO; *Tet3*, 31 controls and 32 *Tet3* KO) and (5) CR study in livers (59 in CR versus 36 control mice with all ages at 1.57 years old). Comparisons in experiments 2 and 3 were based on AgeAccel measures. The color gradient is based on the sign of *t*-test for controls versus experimental mice, with a positive sign indicating that the mice in the control group exhibit higher age acceleration than the mice in the experimental group. **f**, Bar plots for selective tissue types and clocks across Snell dwarf mice (eight normal and eight dwarf mice) GHRKO experiment 1 (12 normal and 11 GHRKO mice), *Tet3*-KO mice (15 normal and 16 *Tet3*-KO mice) and the entire CR experiment, respectively. The orange dots in **c** and **d** and the blue dots in **e** correspond to individual observations. The *y* axes of the bar plots depict the mean of one standard error. All *P* values reported are two sided and are unadjusted for multiple testing.

Retrospective studies indicate that human epigenetic clocks can predict mortality risk and time to death, even when adjusted for chronological age and other risk factors^23,26,27^. We tested whether this applies to pan-mammalian methylation clocks, using data from the Framingham Heart Study Offspring cohort (FHS, *n* = 2,544) and the Women’s Health Initiative (WHI, *n* = 2,107). We devised a method to impute mammalian methylation array data from human Infinium array data (Supplementary Note 5). Our meta-analysis demonstrates that both clocks 2 and 3 can predict human mortality risk after adjusting for age and other confounders. The hazard ratio (HR) for 1 year of epigenetic age acceleration was significantly associated with all-cause mortality (HR = 1.03 and *P* = 6.0 × 10^−19^ for clock 2 and HR = 1.03, *P* = 5.3 × 10^−11^ for clock 3; Fig. 3a,b), although less pronounced than specialized human clocks designed to estimate human mortality risk^22,23,28^.

We evaluated the cross-sectional associations of lifestyle factors and clinical biomarkers with clocks 2 and 3 in the same cohorts. Robust correlation analysis (biweight midcorrelation (bicor)^29^) revealed associations of both clocks with inflammation (C-reactive protein, bicor = 0.12, *P* = 9.9 × 10^−16^) and dyslipidemia (triglyceride levels, *P* = 3.2 × 10^−7^; Supplementary Table 2). Less significant associations were for fasting glucose levels (*P* = 0.0093), body mass index (*P* = 0.011), smoking status (*P* = 0.027) or physical exercise (*P* = 0.0064). While these are nominally significant, they are far weaker than those observed with custom clocks for human mortality risk^23,28^.

### Heritability analysis in humans

To investigate whether genetic control within a species influences the epigenetic aging rates measured by pan-mammalian clocks, we used human pedigree data from the FHS. Pedigree-based polygenic models of epigenetic age, adjusted for age and sex, yielded significant narrow-sense heritability estimates for clock 2 (${h}^{2}$ = 0.44, *P* = 3.4 × 10^−8^) and clock 3 (${h}^{2}$ = 0.41, *P* = 4.0 × 10^−7^). These heritability estimates for pan-mammalian clocks are on par with that of Horvath’s human pan-tissue clock (${h}^{2}$ = 0.39, *P* = 4.0 × 10^−7^)^4^.

### Epigenetic reprogramming reverses epigenetic age

Epigenetic clocks, such as the human pan-tissue clock, suggest that cellular reprogramming based on the Yamanaka factors (collectively termed as OSKM: OCT4, SOX2, KLF4, and c-MYC) induces age reversal^4,30^. To examine whether the universal clocks show a similar age-reversal pattern during reprogramming, we applied clock 2 and clock 3 to a previously published reprogramming dataset in human dermal fibroblasts^31^. We imputed the mammalian methylation array data on the basis of the existing human Infinium array data. Both clocks suggest age reversal after OSKM transduction (Fig. 3c,d). Notably, universal clock 2 showed a decrease in epigenetic age in partially reprogrammed cells after 11 d (Fig. 3c), mirroring observations with human epigenetic clocks^4,30,32^.

### Transgenic mice for studying the somatotropic axis

Growth hormone, generated by somatotropic cells, stimulates body tissue growth, including bone. The somatotropic axis (growth hormone and insulin-like growth factor 1 (IGF-1) levels and their cognate receptors) is central to aging and longevity studies^33^. Decreased growth hormone–IGF-1 signaling extends longevity in various species, including mice^34^. A full-body growth hormone receptor-knockout (KO) (GHRKO) mouse holds the official record for being the longest-lived representative of *Mus musculus*, living 1 week shy of 5 years^33^.

We examined whether reduced growth hormone–IGF-1 pathway activity slows universal pan-mammalian clocks, using three mouse models: (1) Snell dwarf mice, lacking growth hormone production and hence living longer^35,36^, (2) full-body GHRKO mice with increased lifespan^37^ and (3) liver-specific GHRKO mice, showing lowered serum IGF-1 levels but not lifespan increase.

Clock 2 and 3 analyses revealed that Snell dwarf mice exhibit a significantly lower epigenetic age across all considered tissues than wild-type mice (cerebral cortex, Student’s *t*-test, *P* = 2.0 × 10^−8^; kidney, *P* = 6.0 × 10^−10^; liver, *P* = 1.0 × 10^−7^; tail, *P* = 1.0 × 10^−6^; blood, *P* = 2.0 × 10^−3^; spleen, *P* = 0.03; Fig. 3e,f). Similarly, full-body GHRKO mice showed lower epigenetic age in several tissues (liver, *P* = 3.0 × 10^−5^; kidney, *P* = 2.0 × 10^−5^; cerebral cortex, *P* = 0.02; Fig. 3e,f).

Growth hormone receptor signaling stimulates IGF-1 liver synthesis, suggesting that dwarf mice’s epigenetic age reversal may be due to lower circulating IGF-1 levels. This hypothesis, however, is not supported by our epigenetic age measurements of liver-specific GHRKO mice, which exhibit a non-significant difference from the wild-type controls (Fig. 3e). Both clocks 2 and 3 show that the liver-specific GHRKO mice are not epigenetically younger than wild-type mice (Fig. 3e). Unlike full-body GHRKO mice, liver-specific GHRKO mice do not possess a longevity advantage^38,39^.

### Caloric restriction in mice

Caloric restriction (CR), which also slows the somatotrophic axis (growth hormone–IGF-1), is associated with prolonged lifespan in several mouse strains^40,41^. Previous studies using mouse clocks have shown that CR reduces the rate of epigenetic aging in liver samples^5–7^. Using existing methylation data from a murine study of CR^42^, we find that clocks 2 and 3 yield a reduced epigenetic age for mouse liver samples (*P* = 6.0 × 10^−12^ for clock 2, *P* = 7.0 × 10^−15^ for clock 3; Fig. 3e,f). These results for pan-mammalian clocks align with those obtained with mouse-specific clocks^5,43,44^.

### TET enzyme-KO studies in mice

TET enzymes are instrumental in active DNA demethylation. Because hydroxymethylation mediated by TET enzymes is prevalent in brain tissue, we applied the universal clocks to brain tissue samples from *Tet1*-, *Tet2*- and *Tet3*-KO mice. Analysis with our universal clocks revealed that *Tet3*-KO mice exhibit a reduced rate of epigenetic aging (cerebral cortex, *P* = 3.0 × 10^−9^ and striatum, *P* = 2.0 × 10^−12^; Fig. 3e,f). By contrast, significant epigenetic age-reversal effects in brain tissue were relatively weak for *Tet1* (cerebral cortex, *P* = 6.0 × 10^−3^ and striatum, *P* = 2.0 × 10^−4^; Fig. 3e) and could not be observed for *Tet2*-KO mice (*P* > 0.6; Fig. 3e).

The differential effect of *Tet3* KO versus *Tet1* or *Tet2* KO in neurons echoes the results of an epigenetic reprogramming study in mouse retinal ganglion cells (*Oct4*, *Sox2* and *Klf4* (ref. ^45^)).

### Meta epigenome-wide association study of age across species

Universal clocks, founded on penalized regression models, consist solely of CpG sites that are most predictive of age. Consequently, most other age-related CpG sites are not included in the final regression models.

**Fig. 4:**
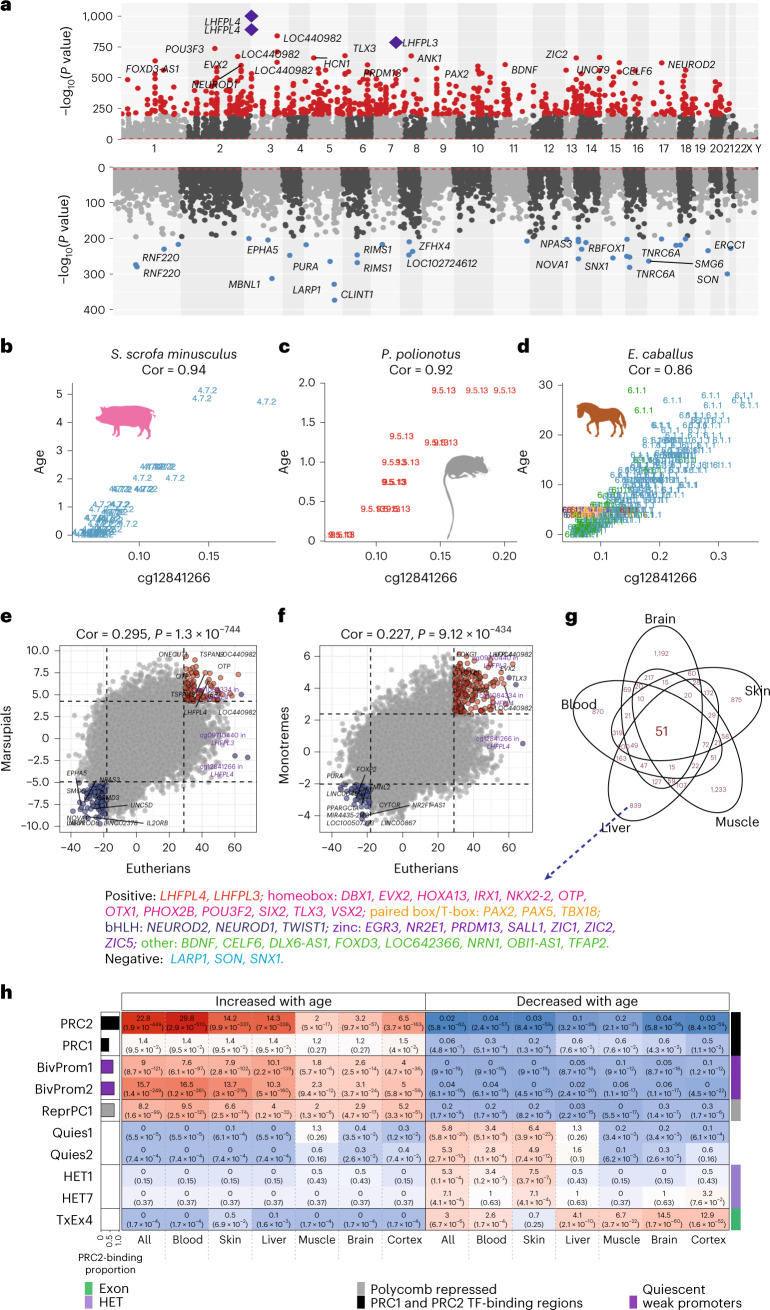
Meta-analysis of methylation change in function of chronological age across species and tissues. **a**–**d**,**g**,**h**, Eutherian EWAS of age. **a**, Meta-analysis −log_10_ (*P* values) for age-related CpG sites (annotated by proximal genes) on chromosomes (*x* axis in hg38). Top and bottom, CpG sites that gain or lose methylation with age, respectively. CpG sites in red and blue denote highly significant positive and negative age correlation (*P* < 10^−200^), respectively. The most significant CpG (cg12841266, *P* = 1.41 × 10^−1,001^) resides in exon 2 on the *LHFPL4* gene in humans and most mammals, followed by cg11084334 (*P* = 2.59 × 10^−891^). These two CpG sites and cg097720 (*P* = 4.97 × 10^−787^) located in the paralog gene *LHFPL3* are marked with purple diamonds. **b**–**d**, Scatterplots of cg12841266 versus chronological age (years) in mini pigs (*Sus scrofa minusculus*) (**b**), Oldfield mice (*Peromyscus polionotus*) (**c**) and horses (*Equus caballus*) (**d**). Tissue samples are labeled by the mammalian species index and colored by tissue type as detailed in Supplementary Data 1.1–1.4. **e**,**f**, Correlation analysis between *Z* scores of EWAS of age in eutherians versus marsupials (**e**) and eutherians versus monotremes (**f**). **g**,**h**, Annotations of the top 1,000 CpG sites with increased or decreased methylation with age that were identified in EWAS meta-analysis across all species and tissues (results in **a**) (brain, cortex, blood, liver, muscle and skin tissues). **g**, The overlap of age-associated CpG sites across various organs, based on the top 1,000 CpG sites showing positive or negative age correlation in EWAS. The Venn diagram includes 51 age-associated CpG sites shared across all organs, adjacent to 38 genes (35 with positive and three with negative age correlation) categorized by protein family. The 35 positive genes are color coded based on their protein family: two in LHFPL, 12 in homeobox, three in paired box or T-box, three in bHLH, seven in zinc finger and eight in others. **h**, Selected universal chromatin state and polycomb group protein enrichment results. ORs (*P* values) are presented in each cell. The color gradient is based on −log_10_ (hypergeometric *P* value) times sign of OR > 1. The complete results are listed in Extended Data Fig. 7. State annotation can be found in Supplementary Data 8.2. HET denotes heterochromatin. Except for the hypergeometric analysis in **h**, all figure *P* values are unadjusted and two sided.

To identify all age-related CpG sites, we carried out two-stage meta-analysis across species and tissues in eutherians (98% of the samples). Our epigenome-wide association study (EWAS) of age indicated that CpG sites becoming increasingly methylated with age (positively correlated with age) are conserved across tissues and species (Fig. 4a).

**Extended Data Fig. 6:**
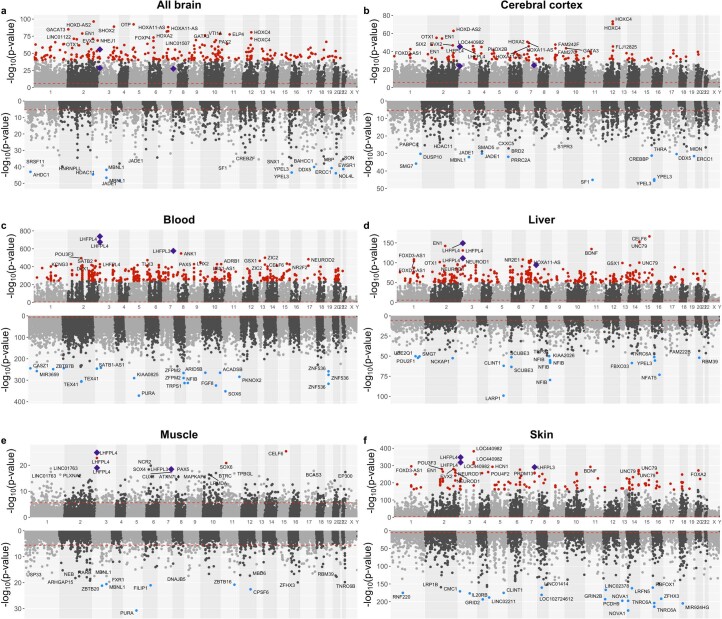
Meta-analysis of chronological age in mammalian samples across specific tissue types. Meta-analysis p-value (-log base 10 transformed) versus chromosomal location (x-axis) according to human genome assembly 38 (hg38) in (**a**), brain tissues (across multiple brain regions), (**b**) cerebral cortex, (**c**) blood, (**d**) liver, (**e**) muscle and (**f**) skin tissues. The upper and lower panels of the Manhattan plot depict the CpG sites that gain/lose methylation with age. In panel a, P values were calculated via two-stage meta-analysis that combined EWAS results across strata formed by species/brain-tissue (with n ≥ 15 samples, Methods). CpGs are colored in red and blue if they exhibit highly significant positive and negative age correlations according to a meta analysis P < 1.0 × 10^−40^, 1.0 × 10^−30^, 1.0 × 10^−250^, 1.0 × 10^−50^, 1.0 × 10^−20^ and 1.0 × 10^−150^ for a–f, respectively. Red dashed horizontal lines denote Bonferroni correction. Gene names are annotated for the top 20 CpGs with positive and negative associations, respectively. CpGs are labeled by adjacent genes. Purple color and diamond shapes mark CpGs of particular interest: cg12841266 and cg11084334 in *LHFPL4* and cg09710440 in *LHFPL3*. All P-values presented in this figure are unadjusted and computed using two-sided tests.

Imposing a stringent unadjusted significance threshold of *α* = 10^−200^ limited our analysis to fewer than 1,000 CpG sites across all eutherian species and tissues (Fig. 4a and Supplementary Data 6.1). Of the 832 resulting age-related CpG sites, those most significantly associate with age were cg12841266 (*P* = 1.4 × 10^−1,001^) and cg11084334 (*P* = 2.6 × 10^−891^), both located in exon 2 of *LHFPL4* (hg38). Notably, cg12841266 exhibited a correlation ≥0.8 in 28 species (Supplementary Data 7; three examples are shown in Fig. 4b–d). Another CpG, cg09710440, resides in exon 1 of *LHFPL3* (*P* = 5.0 × 10^−787^), a paralog of *LHFPL4* (Fig. 4a, Extended Data Fig. 6 and Supplementary Data 6.1–6.7). As *LHFPL4* and *LHFPL3* are in human chromosomes 3 and 7, respectively, their consistent age-related gain of methylation is not due to physical proximity.

Beyond *LHFPL4* and *LHFPL3*, other significant gene pairs among the top 30 age-related CpG sites include *ZIC1* (chromosome 3) and *ZIC2* (chromosome 13), *PAX2* (chromosome 10) and *PAX5* (chromosome 9) and *CELF6* (chromosome 15) and *CELF4* (chromosome 18; Supplementary Data 6.1). Located on separate chromosomes, their shared age-related methylation changes cannot be due to physical proximity, indicating a likely functional role in aging. Intriguingly, each gene pair encodes proteins with activities in development.

**Fig. 5:**
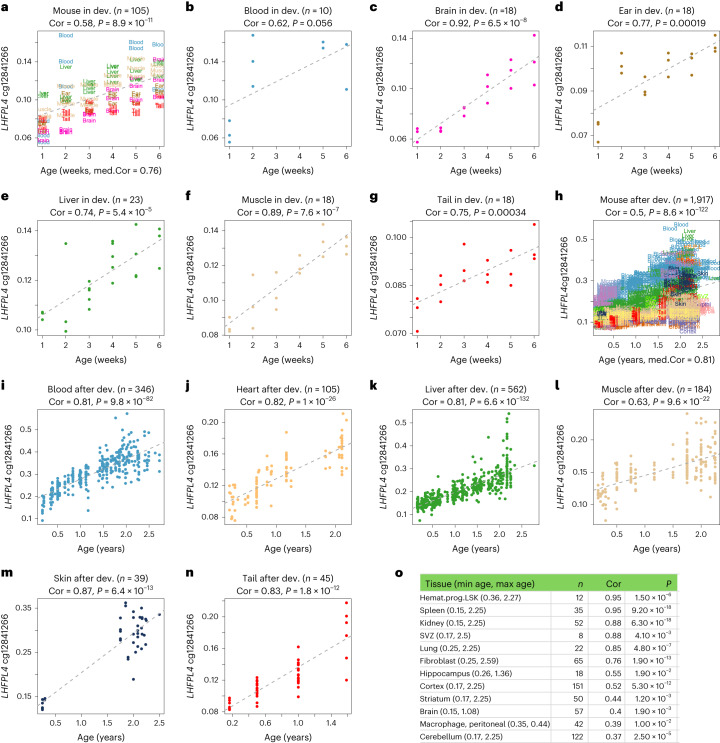
Methylation levels of cg12841266 (*LHFPL4*) versus chronological age in mouse tissues. Results are reported for different tissues and age groups. **a**–**g**, Postnatal development (dev.) (from 1 week to 6 weeks). **h**–**o**, Age effects in adult mice. Mean ± s.d.^96^ of chronological age is 3.5 ± 1.7 (1.0–6.0) weeks in the developmental age group and 1.12 ± 0.72 (0.15–2.78) years in the post-developmental group. **a**,**h**, All tissues combined. Each dot (sample) is colored by the tissue type. **o**, Pearson correlations between the CpG site and age in additional mouse tissues and cell types from the Mammalian Methylation Consortium. Hemato.prog.LSK, hematopoietic progenitor cells with lineage^−^Sca-1^+^c-Kit^+^ phenotype; max, maximum; min, minimum; *n*, sample size; SVZ, subventricular zone. Pearson correlation coefficients and nominal (unadjusted) two-sided correlation test *P* values are shown.

We observed that numerous cytosines change during the initial 6 weeks of murine postnatal development. In particular, *LHFPL4* cg12841266 displayed a positive correlation (*r* > 0.6) with age across murine tissues, especially in the brain and muscle (Fig. 5a–g). High age correlations were also evident in older mice (ranging from 0.2 years to 2.5 years; Fig. 5h–o).

**Fig. 6:**
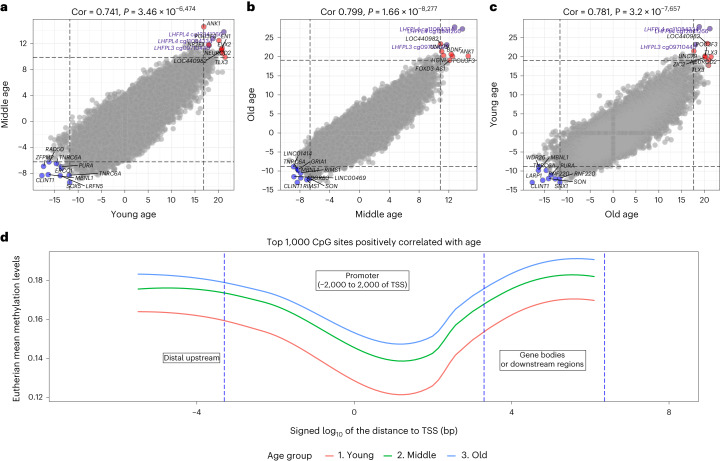
EWAS of age in three different age groups. For each species, the age groups were defined with respect to the average ASM obtained from the Animal Aging and Longevity Database (AnAge) (de Magalhaes et al.^86^). We defined the three age groups using intervals defined by multiples of ASM: young age is defined as age <1.5 × ASM, middle age is defined as age between 1.5ASM and 3.5ASM, and old age is defined by age ≥3.5ASM. Each axis reports *a Z* score from the meta-analysis EWAS of age across all mammalian species and tissues. Each dot corresponds to a CpG site. Labels are provided for the top ten hypermethylated or hypomethylated CpG sites according to the product of *Z* scores in *x* and *y* axes. CpG sites that are located in *LHFPL4* and *LHFPL3* are colored in purple. The Pearson correlation coefficient and corresponding nominal (unadjusted) two-sided correlation test *P* value can be found in the title. **a**, EWAS of age in young animals versus EWAS in middle-aged animals. **b**, EWAS of age in middle-aged animals versus EWAS in old animals. **c**, EWAS of age in young animals versus EWAS of age in old animals. The high pairwise correlations indicate that conserved aging effects in mammals are largely preserved in different age groups. Many of the top CpG sites for conserved aging effects in young mammals remain the top CpG sites for conserved aging effects in old mammals. Specifically, we analyzed the mean methylation levels in eutherians across the three age groups. **d**, Mean methylation (*y* axis) across the top 1,000 CpG sites positively correlated with age according to the EWAS across all mammalian tissue types (Fig. 4a). The *x* axis denotes the distance to the closest TSS in a log_10_ scale of bp. The positive TSS indicates the direction from 5′ to 3′, and the negative TSS indicates from the direction from 3′ to 5′. The horizontal phase is categorized into three regions: distal upstream → promoter → gene bodies. The mean methylation levels are bounded by 0.2, reflecting that fact that CpG sites beginning with lower methylation levels have higher propensity to increase with age.

We obtained a broad overview of age association across different temporal domains by repeating our two-stage meta-EWAS for young, middle and old-age groups (Fig. 6a–c). Importantly, methylation changes related to age in young animals strongly align with those seen in middle-aged or old animals, refuting the idea that these changes are purely tied to organismal development (Fig. 6a–c). This observation is further reinforced by visualizing the mean methylation levels (*β* values) of age-related CpG sites relative to their distances from transcriptional start sites (TSS; Fig. 6d).

### EWAS of age in marsupials and monotremes

We extended the age-related EWAS analysis to marsupials and monotremes. The top age-related CpG sites for marsupials were found near genes involved in development, including *GRIK2* (*P* = 8.8 × 10^−21^; Supplementary Data 6.8), encoding a neurotransmitter-associated glutamate receptor, and *ZIC4* (*P* = 2.7 × 10^−19^), encoding a zinc finger protein. The age-related EWAS in monotremes implicated cg22777952 in *FOXB1* (*P* = 8.1 × 10^−10^; Supplementary Data 6.9), encoding a forkhead box protein. Moderate positive correlation with eutherian age-related methylation changes was observed (*r* = 0.295 in marsupials, Fig. 4e; *r* = 0.227 in monotremes, Fig. 4f), in part due to the lower sample numbers in these groups. However, the age effect on methylation of cg11084334 (not cg12841266) in *LHFPL4* is preserved in marsupials (*P* = 4.8 × 10^−7^; Fig. 4e) and monotremes (*P* = 2.4 × 10^−5^; Fig. 4f), despite these limitations.

### Meta-analysis of age-related CpG sites across specific tissues

To understand age-related CpG sites across species and tissues, we focused on six tissues with many available species: brain (whole and cortex), blood, liver, muscle and skin. We performed an EWAS meta-analysis on 935 whole brains (18 species–brain tissue categories, eight species), 391 cortices (six species), 4,513 blood samples (56 species), 1,063 livers (ten species), 354 muscle samples (five species) and 2,363 skin samples (65 species; Supplementary Data 1.6–1.11).

Consistently across all tissues, CpG sites with positive age correlations outnumbered those with negative correlations (Extended Data Fig. 6). While many age-related cytosines were either specific to individual organs (Supplementary Data 6.2–6.7) or shared between several organs, 51 CpG sites (48 positively and three negatively age related) were common to all five organs (Fig. 4g and Supplementary Table 3). In total, 35 genes were proximal to the 48 positive CpG sites, and three genes were proximal to the three negative CpG sites. Interestingly, 20 of these 35 genes encode transcription factors (TFs), including 11 homeobox proteins, seven zinc finger TFs and two paired box proteins, involved in developmental processes including embryonic development (Supplementary Table 3). The relevance of this becomes evident below, where the chromatin state, function and tissue-specific accessibility associated with the location of age-related CpG sites are described.

### Analyses of chromatin states of DNA bearing age-related cytosines

We observed that 57% of the top 1,000 positively age-related CpG sites were situated in a CpG island (human genome), while only 2% of the top 1,000 negatively age-related CpG sites resided there (EWAS of age across all tissues; Supplementary Data 6.1).

**Extended Data Fig. 7:**
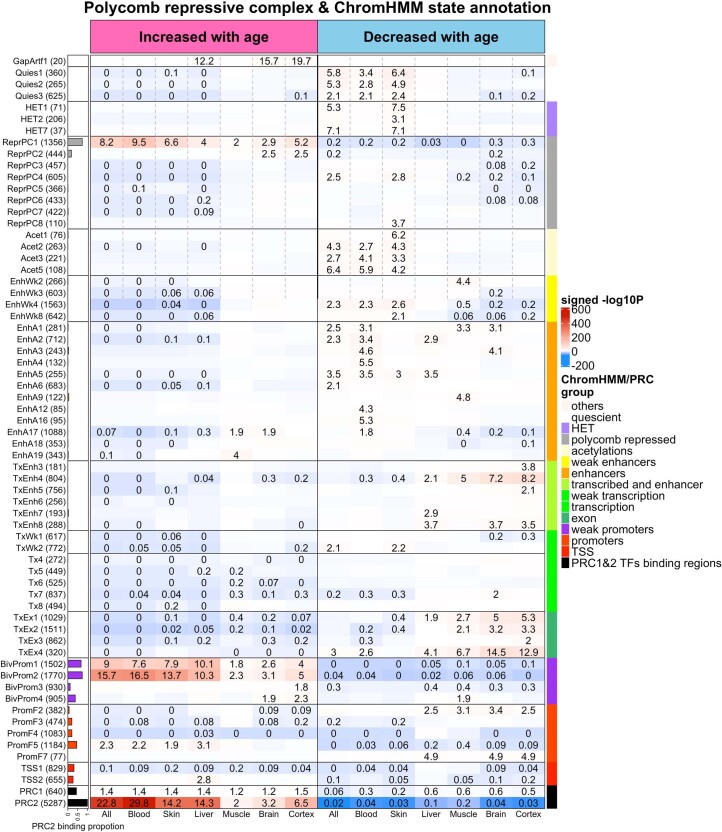
Chromatin state analysis of age-related CpGs. The heatmap color-codes the hypergeometric overlap analysis between age-related CpGs (columns) and two groupings of CpGs (1) universal chromatin states analysis^1^ and (2) binding by polycomb repressive complex 1 and 2 (PRC1, PRC2) defined based on ChIP-Seq datasets in ENCODE^53^, see the last two rows. The first column shows a bar plot that reports the proportion of CpGs that are known to be bounded by PRC2 that ranges from zero to one (PRC2). Note that chromatin states that contain a high proportion of PRC2 bound CpGs overlap significantly with the top 1,000 CpGs that increased with age across tissues and mammal species. For each row (chromatin state or PRC annotation), the table reports odds ratios (OR) from hypergeometric test results for the top 1,000 CpGs that increased/decreased with age from meta-EWAS of age across all, blood, skin, liver, muscle, brain and cerebral cortex tissues, respectively. Unadjusted hypergeometric P values based on one-sided are listed in Supplementary Data 8.3–8.9. The heatmap color gradient is based on −log10 (unadjusted hypergeometric P value) multiplied by the sign of OR greater than one. Red colors denote OR greater than one in contrast with blue colors for OR less than one. Legend lists states based on their group category and PRC group. The y-axis lists state or PRC name and number of mammalian array CpGs inside parentheses. The left/right panel lists the results based on the top 1,000 CpGs with positive/negative age correlation. We displayed 63 universal chromatin states that show significant enrichment/depletion at *P* < 0.001 in any of the tissues. HET, heterochromatin; exon, transcription and exons; weak promoters, bivalent promoters; promoters, promoter flanking.

To understand the epigenetic context of age-related CpG sites, we accessed a detailed universal chromatin state annotation of the human genome. This resource, derived from 1,032 experiments mapping 32 chromatin marks across 100+ human cell and tissue types^46^ (Fig. 4h, Extended Data Fig. 7 and Supplementary Data 8.2–8.9), allowed us to overlay the positions of the top 1,000 age-related CpG sites. We found that positively age-related CpG sites were significantly enriched in states associated with polycomb repressive complex 2 (PRC2)-binding sites (states BivProm1, BivProm2, ReprPC1). These CpG sites localized to PRC2-binding sites, as defined by embryonic ectoderm development (EED), enhancer of zeste 2 PRC2 subunit (EZH2) and PRC2 subunit (SUZ12) binding (the first row of Fig. 4h). This PRC2 enrichment could be observed for all tissue types collectively (odds ratio (OR) = 22.8, hypergeometric *P* = 1.9 × 10^−449^) and when analyzed individually: blood (OR = 29.8, *P* = 2.9 × 10^−510^), liver (OR = 14.3, *P* = 7.3 × 10^−338^), skin (OR = 14.3, *P* = 9.9 × 10^−337^), cortex (OR = 6.5, *P* = 3.7 × 10^−163^) and brain (OR = 3.2, *P* = 9.7 × 10^−57^). Indeed, the majority of the top 1,000 positively age-related CpG sites were significantly enriched in PRC2-binding sites: 80.8% (808 CpG sites) in blood, 67.5% in liver and 67.2% in skin (Supplementary Data 8.1).

PRC2, a transcriptional repressor complex, is a key contributor to H3K27 methylation, a chromatin modification linked to transcriptional repression^47^. Importantly, PRC2-mediated histone 3 lysine 27 (H3K27) methylation is crucial for establishing bivalent promoters, which house histones with both H3K27 trimethylation (H3K27me3) and histone 3 lysine 4 trimethylation (H3K4me3). As such, it is consistent that positively age-related CpG sites are also found to be enriched in bivalent promoter states (rows 3 and 4 of Fig. 4h). They show even greater presence in a bivalent state associated with more H3K27me3 than H3K4me3 (BivProm2) than in BivProm1, associated with more balanced levels of these histone modifications^46^. The top EWAS hit, *LHFPL4* cg12841266, in a bivalent state (BivProm2) and PRC2-binding region (EED-, EZH2-, SUZ12-binding sites), exemplifies this (Supplementary Data 8.1). These mammalian results echo those from human studies^48,49^, in which tissue-independent age-related gain of methylation is characterized by cytosines that are located in PRC2-binding sites and bivalent chromatin domains.

We found that ORs for the overlap between positively age-related CpG sites and PRC2-binding sites were markedly higher in proliferative tissues (blood, skin, liver) than in non-proliferative tissues (skeletal muscle, brain, cerebral cortex; Fig. 4h). The distinction between proliferative and non-proliferative tissues also manifested when considering negatively age-related CpG sites (those that lose methylation levels with age). In highly proliferative tissues (blood, skin), age-related loss of methylation was seen in CpG sites located in select heterochromatin (HET1, HET7), which are marked by histone 3 lysine 9 trimethylation, or inactive chromatin states (Quies1, Quies2), as listed in Supplementary Data 8.2 and Vu & Ernst^46^. Conversely, in non-proliferative tissues, age-related methylation loss could be seen in the exon- and high-expression-associated transcription state TxEx4 (OR = 12.9, *P* = 1.6 × 10^−52^ in the cerebral cortex and OR = 6.7, *P* = 3.7 × 10^−22^ in skeletal muscle). TxEx4 is far less enriched with age-related cytosines that lose methylation in proliferative tissues such as blood (OR = 2.6, *P* = 1.7 × 10^−4^) or skin (OR = 0.7, *P* = 0.25).

### Overlap with late-replicating domains

**Extended Data Fig. 8:**
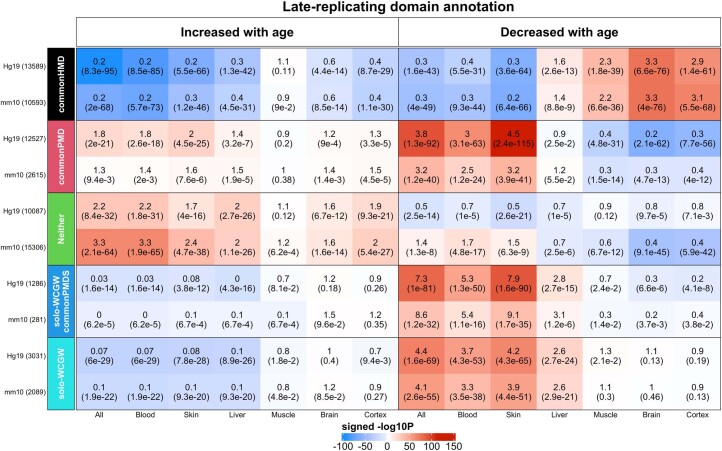
Overlap with late-replicating domains. The heatmap color-codes the hypergeometric overlap analysis between age-related CpGs (columns) and CpGs related to late-replicating domains in hg19 and mm10 assembly^50^, respectively. Two groups of late-replicating domains were analyzed (1) common PMD/HMD structures: highly methylated domains (commonHMD), partially methylated domains (commonPMD), and neither (Neither), and (2) solo-WCGW structures: genome-wide (solo-WCGW) and those in the common PMD regions (solo-WCGW commonPMDs). The y-axis lists categories of late-replicating domains and number of mammalian array CpGs inside parentheses for Hg19 and mm10 genome, respectively. For each row, the table reports odds ratios (OR) from hypergeometric test results for the top 1,000 CpGs that increased/decreased with age from meta-EWAS of age across all, blood, skin, liver, muscle, brain, and cerebral cortex tissues, respectively. The heatmap color gradient is based on -log10 (unadjusted hypergeometric P value) multiplied by the sign of OR greater than one. Red colors denote OR greater than one in contrast with blue colors for OR less than one. The left/right panel lists the results based on the top 1,000 CpGs with positive/negative age correlation. Unadjusted P values are reported and derived from one-sided hypergeometric tests.

Our chromatin state analysis of age-related loss of methylation demonstrated that it is important to distinguish proliferating tissues (blood, skin) from non-proliferative tissues (brain, muscle). Consequently, we examined the correlation between DNA replication and methylation. Late-replicating genome domains, prone to partial methylation, show pronounced methylation loss in solo WCGW cytosines (CpG sites flanked by A or T on either side^50^). We overlaid the top 1,000 age-related CpG sites (positive or negative) on the reported late-replicating domains, which are enriched with partially methylated domains (PMDs)^50^. As previously reported for human tissues^50^, we observed age-related loss of methylation in PMDs and solo WCGW sites in mammalian tissues that proliferate, such as blood and skin (Extended Data Fig. 8 and Supplementary Data 9). Notably, the top 1,000 negatively age-related CpG sites overlap significantly with CpG sites that are both common PMDs and solo WCGW sites (hg19): skin (OR = 7.9, *P* = 1.6 × 10^−90^), blood (OR = 5.3, *P* = 1.5 × 10^−50^) and all tissues (OR = 7.3, *P* = 4.4 × 10^−81^; Extended Data Fig. 8). Contrastingly, non-proliferative tissues, such as the brain, show a different pattern: CpG sites losing methylation with age are enriched in highly methylated domains (HMDs, OR = 3.3, *P* = 1.9 × 10^−74^) over PMDs (OR = 0.2, *P* = 4.9 × 10^−64^). CpG sites gaining methylation with age show weaker overlap with both PMDs and highly methylated domains. Similar findings were observed in late-replicating mouse genome domains (mm10; Extended Data Fig. 8). In summary, pan-mammalian CpG sites losing methylation with age are enriched in late-replicating regions of highly proliferative tissues.

### Functional enrichment analysis of age-related CpG sites

**Fig. 7:**
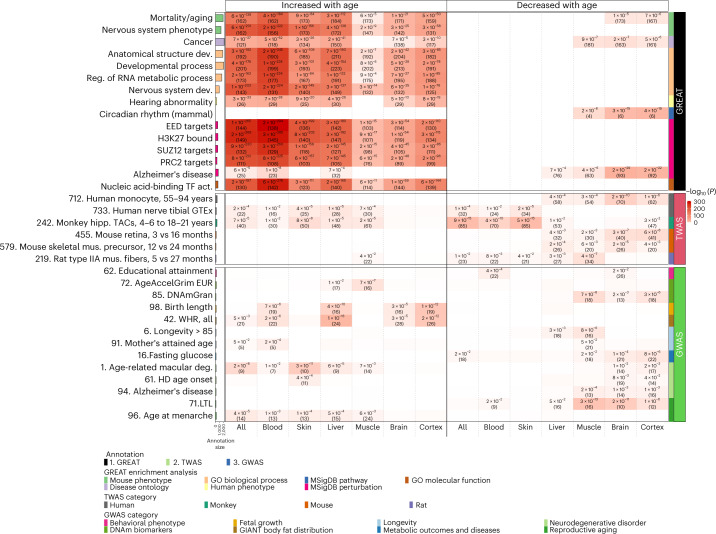
Biological pathways and functional gene sets enriched in age-related CpG sites. Selected results from (1) genomic region-based GREAT functional enrichment (top), (2) gene-based EWAS–TWAS enrichment analysis (middle) and (3) genomic region-based EWAS–GWAS enrichment analysis (bottom). All enrichment analyses were based on hypergeometric tests with background based on the mammalian array. The bar plots in the first column report the total number of genes at each studied gene set adjusted based on the background. The left and right parts of the *x* axis list the top 1,000 CpG sites that increased or decreased with age from meta-EWAS of age across all blood, skin, liver, muscle, brain and cerebral cortex tissues, respectively. On the right side, the first column color band depicts the three types of enrichment analyses. The second column color band depicts (1) six ontologies in the GREAT analysis, (2) four species in our TWAS collections and (3) seven categories of human complex traits in the GWAS as described in the legend. The heatmap color codes −log_10_ (hypergeometric *P* values). Unadjusted hypergeometric *P* values (number of overlapped genes) are reported in the heatmap provided (1) false discovery rate < 0.05, *P* < 0.001 and the number of overlapped genes ≥3 for GREAT analysis, (2) *P* < 0.05 for EWAS–TWAS and (3) *P* < 0.05 for EWAS–GWAS. Comprehensive results can be found in Supplementary Data 10, 12 and 13. Abbreviations: act., activity; deg., degeneration; AgeAccelGrim, epigenetic age acceleration derived from the mortality clock: GrimAge^23^; DNAmGran, DNAm granulocyte (Supplementary Note 5); GIANT, Genetic Investigation of ANthropometric Traits; GTEx, Genotype–Tissue Expression; HD, Huntington’s disease; hipp., hippocampal; LTL, leukocyte telomere length; MSigDB, Molecular Signatures Database; mus., muscle; OPCs, oligodendrocyte precursor cells; reg., regulation; TACs, transiently amplifying progenitor cells; WHR, waist-to-hip ratio.

We used the Genomic Regions Enrichment of Annotations Tool (GREAT) to annotate the potential function of *cis* regulatory regions of age-related CpG sites^51^. We sought to identify biological processes and pathways potentially associated with the top 1,000 positively and negatively age-related CpG sites (Fig. 7 and Supplementary Data 10.1–10.17). To avoid array-design bias, we used mammalian array CpG sites as a background set in our hypergeometric enrichment test.

Analysis of CpG sites positively correlated across all tissues revealed ‘nervous system development’ as a highly significant gene ontology (GO) term (*P* = 1.3 × 10^−203^). This term was consistent across blood (*P* = 1.9 × 10^−224^), liver (*P* = 2.6 × 10^−137^), muscle (*P* = 3.4 × 10^−14^), skin (*P* = 1.7 × 10^−145^), brain (*P* = 6.4 × 10^−35^) and cortex (*P* = 1.0 × 10^−78^). Other significant GO terms included ‘developmental process’, ‘regulation of RNA metabolic process‘, ‘nucleic acid-binding TF activity‘, ‘pattern specification’ and ‘anatomical structure development’ (Fig. 7). The GREAT analysis also indicated that a significant proportion of the top 1,000 positively age-related CpG sites are located in PRC2 target sites (*P* = 8.3 × 10^−212^), which was also true for individual core PRC2 subunits (SUZ12, EED or EZH2; Fig. 7). It follows that these CpG sites were also enriched in promoters with the H3K27me3 modification in embryonic stem cells for all tissues (*P* = 1.9 × 10^−269^), blood (*P* = 2.8 × 10^−285^), liver (*P* = 3.4 × 10^−182^), muscle (*P* = 9.0 × 10^−17^), skin (*P* = 7.8 × 10^−202^), brain (*P* = 1.4 × 10^−54^) and cortex (*P* = 2.7 × 10^−115^; Fig. 7). As PRC2 plays a critical role in development, these results reinforce the epigenetic link between development and aging. This connection is supported by observations that developmentally compromised mice, due to growth hormone receptor (GHRKO) ablation or anterior pituitary gland removal (Snell mice), show reduced rates of epigenetic aging in multiple tissues, as measured by universal epigenetic age clocks (Fig. 3e).

While positively age-related CpG sites (across all tissues) were enriched in 2,961 GO or Molecular Signatures Database terms at a false discovery rate of 0.05 (Supplementary Data 10.1), negatively age-related CpG sites were enriched in only three. Negatively age-related CpG sites in brain and muscle were enriched in genes associated with circadian rhythm (brain, *P* = 3.3 × 10^−15^; cerebral cortex, *P* = 4.0 × 10^−19^; muscle, *P* = 2.3 × 10^−8^; Fig. 7) and Alzheimer’s disease-related gene sets (for example, *P* = 1.8 × 10^−29^ in brain and *P* = 2.4 × 10^−22^ in the cerebral cortex in Fig. 7). These CpG sites also overlapped with gene sets related to mitochondrial function in brain, cerebral cortex and muscle (for example, *P* = 3.6 × 10^−7^; Supplementary Data 10.2).

The GREAT analysis showed enrichment of both positively and negatively age-related CpG sites in mortality or aging gene sets, cancer (Fig. 7) and targets of three Yamanaka factors: SOX2, MYC and OCT4 (Supplementary Data 10.3). Of the 341 genes proximal to positively age-related CpG sites, 162 were implicated in mortality or aging (*P* = 6.3 × 10^−138^; Fig. 7). Similar enrichments were seen in specific tissues: blood (*P* = 3.8 × 10^−184^), liver (*P* = 2.7 × 10^−112^), muscle (*P* = 6.2 × 10^−5^), skin (*P* = 9.1 × 10^−84^), combined brain tissues (*P* = 1.2 × 10^−21^) and the cerebral cortex (*P* = 5.0 × 10^−50^).

As inflammation increases with aging, we assessed the overlap with inflammation-related gene sets (Supplementary Data 10.4). Positively age-related CpG sites are enriched in the gene set associated with inflammation in the murine pancreas (all tissues, *P* = 8.4 × 10^−21^ and skin, *P* = 9.4 × 10^−20^). Negatively age-related CpG sites are enriched in Toll-like signaling (GO:0034121) genes (muscle, *P* = 9.2 × 10^−8^).

Both positively and negatively age-related CpG sites are enriched in immunologic signature gene sets associated with interleukin (IL; for example, IL-6, IL-23) and transforming growth factor (TGF)-β1 exposure in type 17 helper T cells (Supplementary Data 10.4) for notably brain (${P}_{{\rm{negative}}}$ = 6.1 × 10^−11^) and cerebral cortex (${P}_{{\rm{negative}}}$ = 9.1 × 10^−8^) and, to a lesser extent, skin (${P}_{{\rm{positive}}}$ = 4.0 × 10^−4^) tissues.

Concerns that these highly significant enrichments may be a result of potential biases in the mammalian methylation array platform could be discounted after sensitivity analysis, as reported in Supplementary Note 3.

### TF binding

**Extended Data Fig. 9:**
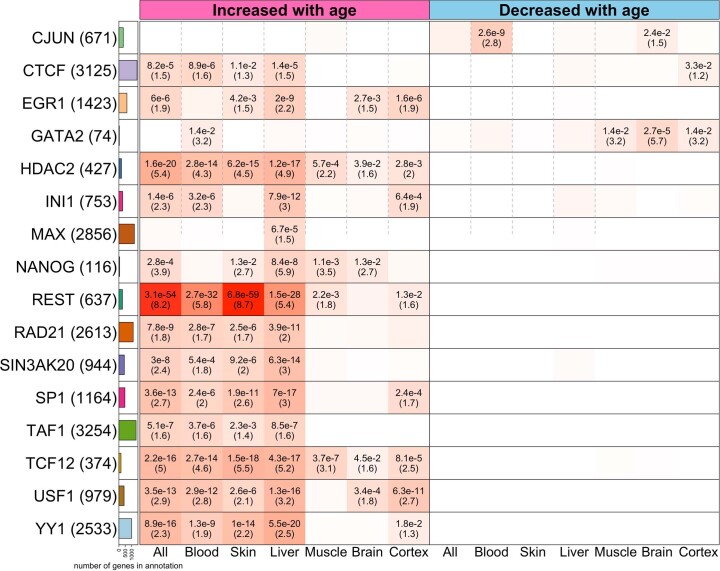
Enrichment with Transcription factor binding regions. We studied the overlapping genomic regions between (1) the CpG sites located in the binding regions of 68 transcription factors (TF) in hg19 and (2) the top 1000 CpGs that increased/decreased with age from EWAS of age across mammalian tissues. TF results (y-axis, rows) versus mammalian EWAS of age are stratified by tissue type (x-axis, columns). The left/right panels of the x-axis list the top 1000 CpGs that increased/decreased with age from meta-EWAS of age across all tissues, blood only, skin only, liver, muscle, brain and cerebral cortex, respectively. The y-axis lists the names of transcription factors and number of mammalian array CpGs located in the binding sites. Background in hypergeometric tests was based on the genes present in our mammalian array. The bar plots in the first column report the total number of genes at each TF according to the background. The heatmap color codes -log10 (unadjusted hypergeometric P value). Unadjusted, one-sided hypergeometric P values (odds ratio) are listed on the heatmap provided *P* < 0.05.

We used the CellBase^52^ and ENCODE databases^53^ to annotate CpG sites with binding sites for 68 TFs identified through chromatin immunoprecipitation followed by sequencing (ChIP–seq) in 17 cell types. If a CpG site overlapped with the binding site of a TF (hg19) in at least one cell type, it was assigned to that TF. Analysis of the most significant age-related CpG sites across mammals showed that the REST TF was the most significant TF for the top 1,000 positively age-related CpG sites across all tissues (OR = 8.4, *P* = 3.1 × 10^−54^), especially in proliferative tissues such as blood (OR = 5.8, *P* = 2.7 × 10^−32^), skin (OR = 8.7, *P* = 6.8 × 10^−59^) and liver (OR = 5.4, *P* = 1.5 × 10^−28^). REST TF enrichment was less significant in non-proliferative tissues such as muscle (OR = 1.8, *P* = 2.2 × 10^−3^), cerebral cortex (OR = 1.6, *P* = 0.01) and brain (OR = 1.4, *P* = 0.09; Extended Data Fig. 9 and Supplementary Data 11).

REST TF ChIP–seq analysis was performed on five cell lines, including a human embryonic stem cell line (Supplementary Data 11.1). REST is known for repressing neuronal genes in non-neuronal tissues, which could explain the weak enrichments in brain regions. Notably, CpG cg12841266 near *LHFPL4* is within the REST-binding region.

Substantial binding enrichments were observed for transcription factor 12 (TCF12) and histone deacetylase 2 (HDAC2). TCF12 is part of the basic helix–loop–helix (bHLH) E-protein family, associated with neuronal differentiation, and top positively age-related CpG sites are proximal to another bHLH gene, *NEUROD1* (Supplementary Table 3 and Supplementary Data 11). Lower enrichments were noted for CCCTC-binding factor (CTCF) and Nanog homeobox (NANOG). For the top 1,000 negatively age-related CpG sites, fewer significant TF binding enrichments emerged, with JUN (c-Jun) in blood (OR = 2.8, *P* = 2.6 × 10^−9^) and brain (OR = 1.5, *P* = 0.024; Extended Data Fig. 9) being exceptions.

### Age-related CpG sites and age-related transcriptomic changes

**Extended Data Fig. 10:**
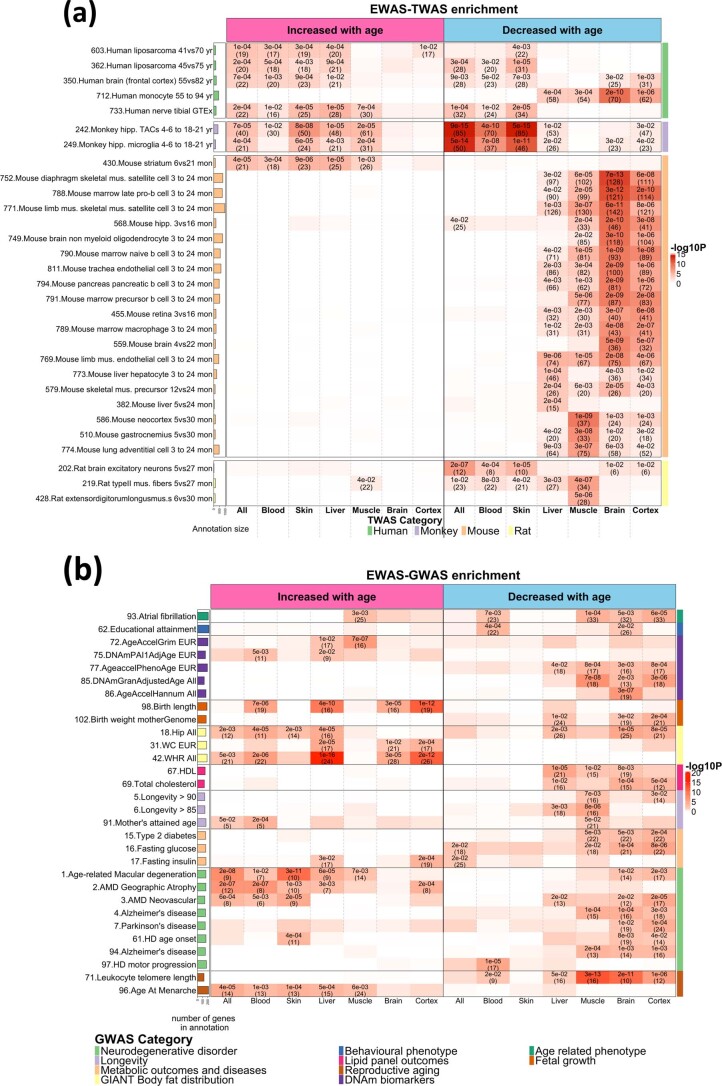
EWAS-TWAS and EWAS-GWAS enrichment. Panel (**a**) illustrates the overlap between genes identified in transcriptome-wide association studies (TWAS) across various cell types or species, and the top 1,000 CpGs that have increased/decreased with age in EWAS across mammalian tissues. TWAS results are stratified by tissue type, including all tissues, blood, skin, liver, muscle, brain, and cerebral cortex. Overlapping genes with P < 0.05 are reported. Similarly, Panel (**b**) demonstrates the overlaps between the top 2.5% genes implicated in genome-wide association studies (GWAS) of human complex traits, and the top 1,000 CpGs that have increased/decreased with age in EWAS across mammalian tissues. GWAS results are also stratified by tissue type, with significant overlaps reported where P < 0.05. Both panels utilize unadjusted, one-sided hypergeometric P values, with a background for hypergeometric tests derived from genes (panel a) or genomic regions (panel b) in our mammalian array. The heatmap color encodes -log10 P values. The right-side annotation indicates (a) the species categories for TWAS collections and (b) phenotype categories for GWAS collections. Further details for TWAS and GWAS indices are available in Supplementary Data 12 & 13. Abbreviations: (a) hipp.=hippocampus, MPNST = malignant peripheral nerve sheath tumor, mus.=muscle, TACs = transiently amplifying progenitor cells. (b) All = All ancestries, EUR = European ancestry, AFR = African American ancestry, FTD = frontotemporal dementia, WHR = waist to hip ratio.

We studied whether the top 1,000 positively and negatively age-related CpG sites neighbor genes with age-correlated mRNA levels. Using GenAge^54^ and Enrichr^55,56^ databases, we scrutinized age-specific transcriptome-wide association studies (TWAS) in four mammalian species. The EWAS–TWAS overlap analysis (Fig. 7, Extended Data Fig. 10a and Supplementary Data 12) indicates significant overlaps between age-related CpG sites and transcriptomic age changes in several species, including Genotype–Tissue Expression (GTEx) human tibial nerve samples, normal monkey hippocampal samples (*P* = 9 × 10^−15^) and various rat and mouse tissues. However, the age-related EWAS and TWAS overlap is generally weak and tissue specific.

### Age-related CpG sites and genome-wide association studies of human traits

We compared proximal genes of the top 1,000 positively and negatively age-related CpG sites with the top 2.5% of genes implicated in various human genome-wide association studies (GWAS). Notable enrichments were seen in genes associated with waist-to-hip ratio for positively age-related CpG sites in livers (${P}_{{\rm{positive}}}$ = 1.0 × 10^−16^), and with human length at birth for positively age-related CpG sites in the cortex (${P}_{{\rm{positive}}}$ = 1.0 × 10^−12^) and liver (${P}_{{\rm{positive}}}$ = 2.0 × 10^−10^; Fig. 7). Significant enrichments (defined here as nominal *P* < 5.0 × 10^−4^) were also seen with genes linked to mother’s longevity (mother attained age; ${P}_{{\rm{positive}}}$ = 2.0 × 10^−4^; Fig. 7, Extended Data Fig. 10b and Supplementary Data 13.1–13.7), human longevity for negatively age-related CpG sites in muscle (${P}_{{\rm{negative}}}$ = 8.0 × 10^−6^), epigenetic age acceleration on the mortality clock (GrimAge ${P}_{{\rm{positive}}}$ = 7.0 × 10^−7^ in muscle), age-related macular degeneration (${P}_{{\rm{positive}}}$ = 2.0 × 10^−8^ in all tissues), Alzheimer’s disease (${P}_{{\rm{negative}}}$ = 1.0 × 10^−4^ in brain), leukocyte telomere length (${P}_{{\rm{negative}}}$ = 3.0 × 10^−13^ in muscle and ${P}_{{\rm{negative}}}$ = 2 × 10^−11^ in brain) and age at menarche (${P}_{{\rm{positive}}}$ = 4.0 × 10^−5^ in all tissues). Overall, our GWAS overlap analysis indicates that pan-mammalian age-related CpG sites are proximal to genes influencing human development (birth length, menarche), obesity and longevity.

### Single-cell ATAC-seq analysis in human bone marrow

**Fig. 8:**
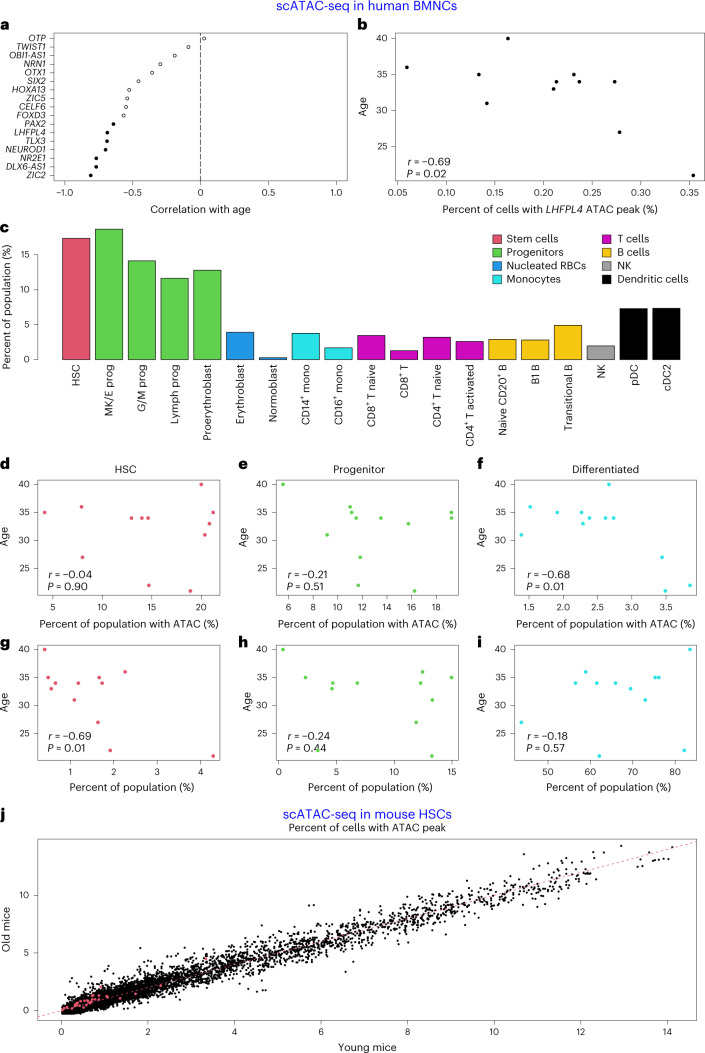
scATAC-seq analysis in human bone marrow and mouse HSCs. **a**–**i**, Results using human BMNCs. **j**, Murine HSCs. **a**, scATAC-seq results for 17 of the 35 genes (listed in Supplementary Table 3) that show a called ATAC peak in the region overlapping with our top CpG sites with positive age correlation. The *y* axis lists the gene symbol. The *x* axis reports the Pearson correlation between chronological age and the percentage of cells with an scATAC-seq signal overlapping the respective CpG site (labeled by the adjacent gene). The genes are ordered by correlation estimate (from the most negative). A negative correlation estimate indicates that the accessibility of the CpG site decreases with chronological age. Each dot presents a gene. Seven genes with *P* < 0.05 are marked with a solid shape. **b**, scATAC-seq analysis results for *LHFPL4*. The *y* axis depicts chronological age, and the *x* axis shows the percentage of cells with an scATAC-seq signal. **c**, Percentage of cells identified containing scATAC-seq signal in one of the seven significantly associated genes averaged across all samples. Cells are split into the called identities using the scRNA-seq measurement including HSCs, the various progenitors and differentiated cells. DC, dendritic cell; mono, monocyte; MK/E prog, megakaryocyte-erythroid progenitor; G/M prog, granulocyte-monocyte progenitor; NK, natural killer; prog, progenitor; RBC, red blood cell. **d**–**f**, The percentage of these three cell populations (HSC (**d**), progenitor (**e**) and differentiated cell type (**f**)) that contain at least one ATAC-seq signal in any of the seven significant genes, plotted against the age of each individual (*y* axis). **g**–**i**, The percentage of these three cell populations per individual (HSC (**g**), progenitor (**h**) and differentiated cell type (**i**)), plotted against the age of each individual. **j**, The percentage of cells with called ATAC peaks overlapping with our mammalian CpG sites in young mouse (10-week) versus old mouse (20-month) HSCs. The red dots denote 33 of the top 35 positively age-related CpG sites (listed in Supplementary Table 3) that map to the mouse genome. The red dashed line corresponds to the diagonal line (*y*=*x*). All *P* values reported are unadjusted and two sided.

Low-methylated regions distant from TSS correlate with open chromatin, TF binding and enhancers^57^. Hence, our top positively age-related pan-mammalian CpG sites (initially low in methylation, gaining methylation with age) could imply a gradual loss of these open chromatin regions. To validate this, we examined the association between the top 35 positively age-related CpG sites (Supplementary Table 3) and chromatin accessibility in single cells from human bone marrow mononuclear cells (BMNCs). Single-cell assay for transposase-accessible chromatin with sequencing (scATAC-seq) data from a recent study^58^ employed 10x Multiome technology to profile both ATAC and gene expression within the same cell across ten donors of varying age. Overlaying the genomic regions of the top 35 CpG sites (Supplementary Table 3) with the called ATAC peaks within the BMNC dataset identified 17 genes, including *LHFPL4* (Supplementary Data 14.1 and Fig. 8a).

We calculated the percentage of cells per individual with the respective peak. A strong, statistically significant negative correlation (Fig. 8b) was found between age and the number of cells with the ATAC peak overlapping cg12841266 in *LHFPL4*. This shows that, with age (as methylation increases), open chromatin cell number decreases. Of 17 gene regions, 16 correlated negatively with age, with seven being statistically significant (*P* < 0.05; Fig. 8a). The hypermethylated sites were highly enriched for this age-associated accessibility loss (*P* < 0.001; Fig. 8b). The significant genes (*LHFPL4*, *TLX3, ZIC2*, *PAX2*, *NR2E1*, *NEUROD1*, *DLX6-AS1*) are related to developmental processes (Supplementary Table 3). *ZIC5*, another Zic family gene, also showed a nearly significant negative age correlation (*r* = −0.54, *P* = 0.07; Supplementary Data 14.1). No scATAC-seq signal was detected in the cg09710440 region of *LHFPL3*, possibly due to proximity to a bivalent gene’s TSS (232 bp).

We examined whether the seven significant ATAC peaks identified a particular cell type subset. Due to the sparsity of scATAC-seq data, we determined the fraction of each cell group containing at least one of these regions. We found that stem cell–progenitor populations had a higher proportion of open chromatin at these sites than differentiated cells (mean of 14.9% versus mean of 2.9%; Fig. 8c). This suggests that the observed age-related reduction of open chromatin states could be due to the loss (for example, death or differentiation) of progenitor cells in the tissue.

We studied three cell groups: hematopoietic stem cells (HSCs), progenitor cells and differentiated cells. Age showed a negative correlation with the percentage of HSCs (*r* = −0.69, *P* = 0.01) but no significant correlation with progenitor or differentiated cells (Fig. 8g–i). Next, we analyzed the correlation between age and the proportion of cells containing an ATAC peak in at least one of the seven significant CpG regions (Fig. 8d–f). Differentiated cells demonstrated a significant loss of ATAC signal in these regions with age (*r* = −0.68, *P* = 0.01; Fig. 8f), whereas no change was seen in HSCs or progenitor cells (Fig. 8d,e). This suggests that these regions, gaining methylation and losing accessibility with age, belong to a differentiated cell population. Lastly, analyzing increasing lists of positively age-related CpG sites, we noted that the percentage of cells with an ATAC peak at these locations decreasing with age in human BMNCs (median correlation < −0.2 across the top 500 or 1,000 positively age-related CpG sites).

### scATAC-seq analysis in murine HSCs

We tested whether our human HSC findings extended to murine HSCs by analyzing another public scATAC-seq dataset from murine HSCs with four replicates each in young (10-week) and old (20-month) mice^59^. This dataset provided access to our age-related CpG sites in 4,492 young and 3,300 old HSCs. Of the top 35 positively age-related CpG sites, 33 overlapped with ATAC peaks (Supplementary Data 14.2). We then calculated the proportion of HSCs in each age group with the respective peak. The proportion of old HSCs with a peak near *Lhfpl4* was not significantly different from that of young HSCs (OR = 0.94, *P* = 0.7), implying no observable age-related chromatin compactification in murine HSCs. This was also true for the other 32 CpG sites and their associated peaks. Contrarily, the proportion of old HSCs with an ATAC peak was significantly higher than that of young HSCs for five CpG sites (near *Bdnf*, *Isl1*, *Twist1*, *Nr2e1*, *Sall1*; Fisher exact *P* value < 0.05; Supplementary Data 14.2), indicating age-related chromatin opening (Fig. 8j), aligning with Itokawa et al.’s report^59^.

## Discussion

The consistent age-related alterations in DNA methylation profiles across mammalian species challenges the view that aging is simply due to the random accumulation of cellular damage. Our Mammalian Methylation Consortium investigated this question with an extensive set of DNA methylation profiles from 348 species^9^, using 174 eutherian, nine marsupial and two monotreme species in this study.

We found a set of CpG sites in DNA sequences conserved across mammals consistently changing with age, predominantly gaining methylation. These CpG sites are often in PRC2-binding sites and the bivalent chromatin states BivProm1 and BivProm2, regulating the expression of genes involved in the process of development^47,60,61^, which is one of the most conserved biological processes that threads through all mammalian species. Examples of age-related CpG sites include those near *LHFPL4* and *LHFPL3*. The known function of *LHFPL4* in synaptic clustering of γ-aminobutyric acid (GABA) receptors does not provide a clear connection to aging across tissues. Nevertheless, the specificity of their methylation change with age is clear, considering their distinct chromosomal locations, as observed with gene pairs such as *LHFPL3*–*LHFPL4*, *ZIC2*–*ZIC5*, *PAX2*–*PAX5* and *CELF4*–*CELF6*.

The scATAC-seq analysis of BMNCs revealed that age-correlated CpG sites are located in regions that lose chromatin accessibility with age in differentiated cells but not in progenitor cells. This suggests that methylation likely instigates such chromatin compaction^62^, hindering PRC2 access to its target sites. We observed this phenomenon in human bone marrow, where (1) top age-related PRC2 targets are open in substantially more progenitor cells than differentiated cells and (2) the percentage of progenitor cells with open age-related PRC2 targets did not diminish with age. Similarly, the percentage of murine HSCs with open age-related PRC2 targets did not diminish with age. By contrast, the percentage of differentiated human bone marrow cells with open PRC2 targets diminished with age, underscoring the need for further research into other differentiated cell types.

When it comes to age-related gain of methylation, it is important to distinguish proliferative tissues from non-proliferative tissues such as the brain and muscle. The overlap between PRC2-binding sites and positively age-related changes is far more pronounced in proliferative tissues than in non-proliferative tissues (Fig. 4h). The dichotomy between proliferative and non-proliferative tissues is even more pronounced when it comes to characterizing age-related loss of methylation.

In proliferative tissues, negatively age-related CpG sites are often located in quiescent chromatin states, heterochromatin and PMDs. Interestingly, PMDs are in late DNA-replication regions. As methylation of replicated DNA is slow and only completed very late in S and G2 phases, late-replicated regions of the DNA are particularly disadvantaged in this regard. Indeed, progressive methylation loss in PMDs is exploited as a mitotic clock, which also correlates very well with chronological age^50^. As such, their identification as pan-mammalian negatively age-related CpG sites is entirely consistent with studies observed in human cells. Interestingly, this late-replication effect on DNA methylation can be prevented by the binding of histone 3 lysine 36 trimethylation (H3K36me3) to these regions^50^. This appears to be mediated by H3K36me3 recruitment of DNA methyltransferase 3 (DNMT3) to unmethylated and newly replicated DNA. Conversely, functional loss of NSD1, the enzyme that generates H3K36me3, leads to hypomethylation of DNA and accelerated epigenetic aging^63,64^. Age-related loss of methylation in non-proliferative tissues (brain and muscle), on the other hand, is observed at CpG sites located in an exon-associated transcription state (TxEx4), which is the most highly enriched state for transcription termination sites and is associated with the highest gene expression levels across many cell and tissue types^46^.

Unlike CpG sites that gain methylation with age, CpG sites that lose methylation are typically not related to developmental genes. Instead, they are related to genes of circadian rhythm and mitochondria, the functions of which are progressively eroded with age. Finally, the *LARP1* gene, which is proximal to the highest-ranked hypomethylated cytosine in the liver and second across all tissues, encodes an RNA-binding protein that is involved in several processes, including post-transcriptional regulation of gene expression and translation of downstream targets of mammalian target of rapamycin (mTOR)^65^. mTOR has very well-documented links with aging and longevity^66^ and is also linked to epigenetic aging^67,68^. Overall, we provide collective evidence that the methylated mammalian age-related CpG sites that we identified are not merely stochastic marks accrued with age. They are instead methylation changes that capture multiple facets of mammalian aging.

The deterministic features of these age-related changes on the mammalian epigenome make a compelling case that aging is not solely a consequence of random cellular damage accrued in time. It is instead a pseudo-programmed process that is also intimately associated with mammalian development that begins to unfold from conception. This is supported by and is consistent with the finding that genes proximal to age-related CpG sites were also identified by GWAS of human development features such as length at birth and age at menarche. A large body of literature including those by Williams in 1957 (refs. ^69,70^) has suggested a connection between growth and development and aging. More recently, several authors have suggested epigenetics to be the link between these two processes^69,71–80^. This notion is further supported by the recent demonstration of age reversal through the expression of Yamanaka factors^45,81–84^, which can also be observed for our universal pan-mammalian clocks (Fig. 3c,d).

According to the pseudo-programmatic theory of aging, the process of aging is very much a consequence of the process of development, and the ticking of the epigenetic clock reflects the continuation of developmental processes^69,80^. As predicted by the epigenetic clock theory of aging, universal epigenetic clocks provide a continuous readout of age from early development to old age in all mammals, as this feature underlies the continuous and largely deterministic process of aging from conception to tissue homeostasis^74^. Consistent with this theory, pan-mammalian methylation clocks are slowed by conditions that delay growth and/or development including Snell mice and full-body GHRKO mice. The successful construction of universal clocks is a compelling mathematical demonstration of the deterministic element in the process of aging that transcends species barriers within the mammalian class. Indeed, the centrality of PRC2, which is also present in non-mammalian classes, implies that the process of aging that is uncovered here is likely to be shared by vertebrates in general. Our human epidemiological studies and mouse interventional studies show that pan-mammalian clocks relate to human and mouse mortality risk, respectively. Cross-sectional epidemiological studies in humans reveal that the pan-mammalian clocks correlate with markers of inflammation (C-reactive protein) and dyslipidemia (triglyceride levels).

Our study has certain limitations. The study primarily focuses on highly conserved DNA sequences, thus limiting our examination to approximately 36,000 CpG sites of the tens of millions that exist in most mammalian genomes. Additionally, our array platform exhibits a slight bias, featuring more probes that align with eutherian genomes than with marsupial genomes^8^.

Overall, our results demonstrate that select epigenetic aging effects are universal across all mammalian species and capture multiple processes and manifestations of age that have thus far been thought to be unrelated to each other. We expect that the availability of pan-mammalian epigenetic clocks will open the path to uncovering interventions that modulate conserved aging processes in mammals.

## Methods

### Ethics

All local ethical guidelines were followed, and necessary approvals from respective human ethical review boards and animal ethical committees were duly obtained. Details can be found in Supplementary Notes 1, 2 and 4.

### Statistics and reproducibility

Data collection and analysis were not performed blind to the conditions of the experiments. In the ensuing sections, we delineate the quality-control measures for our samples and the statistical methods employed in each analysis, with additional details provided in Supplementary Notes 1 and 5.

### Tissue samples

We used a subset of the data from the Mammalian Methylation Consortium for which age information was available^9^. The tissue samples are described in Supplementary Data 1.1–1.4, and related citations are listed in Supplementary Notes 1 and 2. We used the SeSAMe normalization method^85^.

### Quality controls for establishing universal clocks

Our epigenetic clocks were trained and evaluated on samples with highly confident age assessments (less than 10% error). We focused on typical aging patterns, hence excluding tissues from preclinical anti-aging or pro-aging intervention studies.

### Species characteristics

Species characteristics such as maximum lifespan (maximum observed age) and ASM were obtained from an updated version of AnAge^86^ (https://genomics.senescence.info/species/index.html). To facilitate reproducibility, we have posted this modified and updated version of AnAge in Supplementary Data 1.13.

### Three universal pan-mammalian clocks

We applied elastic net regression models to establish three universal mammalian clocks for estimating chronological age across all tissues (*n* = 11,754 from 185 species) in eutherians (*n* = 11,439 from 174 species), marsupials (*n* = 210 from nine species) and monotremes (*n* = 15 from two species). The three elastic net regression models, implemented using the glmnet 4.1-7 package in R, corresponded to different outcome measures described in the following:

1. log-transformed chronological age: $\log ({\rm{Age}}+2)$, where an offset of 2 years was added to avoid negative numbers in case of prenatal samples,
2. $$-{\rm{log}}(-{\rm{log}}({\rm{RelativeAge}}))$$ and
3. log–linear transformed age.

DNAmAge estimates of each clock were computed via the respective inverse transformation. Age transformations used for building universal clocks 2 and 3 incorporated a selection of three species characteristics: gestational time $({\rm{GestationT}})$, age at sexual maturity (${\rm{ASM}}$) and maximum lifespan $({\rm{MaxLifespan}})$. All of these species variables surrounding time are measured in units of years.

#### loglog transformation of relative age for clock 2

Our measure of relative age leverages gestation time and maximum lifespan. We define relative age $({\rm{RelativeAge}})$ and apply the double logarithmic ${\rm{loglog}}$ transformation:

1 $${\rm{RelativeAge}}=\frac{{\rm{Age}}+{\rm{GestationT}}}{{\rm{MaxLifespan}}+{\rm{GestationT}}}$$

2 $${\rm{loglogAge}}=-\log \left(-\log \left({\rm{RelativeAge}}\right)\,\right).$$

By definition, ${\rm{RelativeAge}}$ is between 0 and 1, and ${\rm{loglogAge}}$ is positively correlated with age. The incorporation of gestation time is not essential. We simply include it to ensure that ${\rm{RelativeAge}}$ takes on positive values. We used the double logarithmic transformation to link relative age to the covariates (cytosines) for the following reasons. First, the transformation maps the unit interval to the real line. Second, this transformation ascribes more influence on exceptionally high and low age values (Extended Data Fig. 1a–c). Third, this transformation is widely used in the context of survival analysis. Fourth, this non-linear transformation worked better than the identity transformation in terms of age correlation and calibration.

The regression model underlying universal clock 2 predicts ${\rm{loglogAge}}$. To arrive at the DNAmAge, one needs to apply the inverse transformation to ${\rm{loglogAge}}$ based on the double exponential transformation:

3 $$\begin{array}{l}{\rm{DNAmAge}}=\exp \left(-\exp \left(-{\rm{loglogAge}}\right)\right)\\ \qquad\qquad\qquad\times \left({\rm{MaxLifespan}}+{\rm{GestationT}}\right)-{\rm{GestationT}}.\end{array}$$

All species characteristics (for example, maximum lifespan, gestational time) come from our updated version of AnAge. We were concerned that the uneven evidence surrounding the maximum age of different species could bias our analysis. While billions of people and many mice have been evaluated for estimating the maximum age of humans (122.5 years) or mice (4 years), the same cannot be said for any other species. To address this concern, we made the following assumption: the true maximum age is 30% higher than that reported in AnAge for all species except for humans and mice. Therefore, we multiplied the reported maximum lifespan of non-human or non-mouse species by 1.3. Our predictive models turn out to be highly robust with respect to this assumption.

#### Transformation based on log–linear age for clock 3

Our measure of log–linear age leverages ${\rm{ASM}}$. The transformation has the following properties: it takes the logarithmic form when the chronological age is young, and it takes the linear form otherwise. It is continuously differentiable at the change.

First, we define a ratio of the age relative to ASM, termed ${\rm{RelativeAdultAge}}$, as the following:

4 $${\rm{RelativeAdultAge}}=\frac{{\rm{Age}}+{\rm{GestationT}}}{{\rm{ASM}}+{\rm{GestationT}}},$$

where the addition of ${\rm{GestationT}}$ ensures that the ${\rm{RelativeAdultAge}}$ is always positive. To model a faster rate of change during development, we used a log–linear transformation on ${\rm{RelativeAdultAge}}$ based on a function that generalizes the original transformation proposed for the human pan-tissue clock^4^:

5 $$y=f\left(x{\rm{;}}\,m\right)=\left\{\begin{array}{ll}\frac{x\,}{m}-1,&\frac{x}{m}\ge 1\\ {\rm{log }}\frac{x\,}{m},&\frac{x}{m} < 1\,\end{array}\right.$$

6 $${f}^{-1}\left(y{\rm{;}}\,m\right)=\left\{\begin{array}{ll}m\left(y+1\right),& y\ge 0\\ m{e}^{y},& y < 0\,\end{array}\right.\,.$$

In the function *f*(*x*;*m*), *x* denotes ${\rm{RelativeAdultAge}}$, *m* represents a parameter and f represents the log-linear transformation. The output, *y*, is the results of applying the function *f* to *x* and *m*. This transformation is designed to reflect a higher rate of change for younger RelativeAdultAges when *x* ≤ *m*. This transformation ensures continuity and smoothness at the change point at $x=m$.

In the following, we describe the estimation of the parameter *m*. To ensure that the maximum value of $y$ is the same across all species, the parameter $m$ should be proportional to the maximum of $x$ for each species, that is, the best value for *m* would be the oracle value

${m}^{* }={c}_{1}\left(\frac{{\rm{MaxLifespan}}+{\rm{GestationT}}}{{\rm{ASM}}+{\rm{GestationT}}}\right)\,$ (Extended Data Fig. 1d).

The proportionality constant ${c}_{1}$ controls the distribution of $y$. We chose the value of ${c}_{1}$ so that $y$ follows approximately a normal distribution with mean zero. Because we wanted to define clock 3 without using ${\rm{MaxLifespan}}$, we opted to use the ratio $\frac{{\rm{GestationT}}}{{\rm{ASM}}}$ as a surrogate for the oracle value ${m}^{* }$. We achieved this approximation by fitting the following regression model with all mammalian species available in our AnAge database,

7 $$\log \frac{{\rm{MaxLifespan}}+{\rm{GestationT}}}{{\rm{ASM}}+{\rm{GestationT}}}\approx 2.92+0.38\times \log \frac{{\rm{GestationT}}}{{\rm{ASM}}}.$$

The two log variables in equation (7) have moderate correlation (*r* = 0.5). Subsequently, we defined $\hat{m}$ as follows:

8 $$\hat{m}={c}_{2}{\left(\frac{{\rm{GestationT}}}{{\rm{ASM}}}\right)}^{0.38},$$

where ${c}_{2}={c}_{1}{e}^{2.92}.$ We chose ${c}_{2}=5.0$ so that log–linear age termed *y* in equation (5) follows approximately a normal distribution with mean zero (median = 9.0 × 10^−4^, skewness = −0.02; Extended Data Fig. 1f). Setting *x*=RelativeAdultAge in equation (5) results in

9 $$f({\rm{RelativeAdultAge}};\,\hat{m})=\left\{\begin{array}{c}\frac{{\rm{RelativeAdultAge}}\,}{\hat{m}}-1,\,{\rm{RelativeAdultAge}}\ge \hat{m}\\ \log \frac{{\rm{RelativeAdultAge}}\,}{\hat{m}},\,{\rm{RelativeAdultAge}} < \hat{m}\,\end{array}\right. \,.$$

Universal clock 3 predicts ${\rm{loglinearAge}}$ (denoted as $y$). To arrive at an age estimate, we employ both equations (4) and (6):

10 $${\rm{DNAmAge}}=\left\{\begin{array}{c}\hat{m}\times \left({\rm{ASM}}+{\rm{GestationT}}\right)\times \left(y+1\right)-{\rm{GestationT}}\,,\,y\ge 0\\ \hat{m}{\times \left({\rm{ASM}}+{\rm{GestationT}}\right)\times e}^{y}-{\rm{GestationT}}\,,\,y < 0\end{array}\right.\,.$$

#### Statistics for performance of model prediction

To validate our model, we used DNAmAge estimates from LOFO and LOSO analyses, respectively. At each type of estimate, we computed Pearson correlation coefficients and MAE between DNAm-based and observed variables across all samples. Correlation and MAE were also computed at the species level, limited to the subgroup with *n* ≥ 15 samples (within a species). We reported the medians for the correlation estimates (median correlation) and the medians for the MAE estimates (med.MAE) across species. Analogously, we repeated the same analysis at the species–tissue level, limited to the subgroup with at least 15 samples (within a species–tissue category).

For Extended Data Fig. 2, we evaluated the difference Delta.Age ($\Delta {\rm{Age}}$) between the LOSO estimate of DNAmAge and chronological age at half the maximum lifespan (0.5 × ${\rm{MaxLifespan}}$). As expected, $\Delta {\rm{Age}}={\rm{LOSO}}\; {\rm{DNAmAge}}-(0.5\times {\rm{MaxLifespan}})$ is negatively correlated with species maximum lifespan.

### Epigenetic age acceleration

To adjust for age, we defined epigenetic age acceleration (AgeAccel) as the raw residual resulting from regressing DNAmAge (from universal clocks 2 and 3) on chronological age. By definition, the resulting AgeAccel measure is not correlated with chronological age.

### Human epidemiological cohort studies

We applied our universal clocks 2 and 3 to 4,651 individuals from (1) the FHS Offspring cohort (*n* = 2,544 Caucasians, 54% women)^87^ and (2) the WHI cohort^88,89^ (*n* = 2,107, 100% women; Supplementary Note 4). Methylation levels were profiled in blood samples using the Illumina 450k arrays. The FHS cohort had a mean (s.d.) age of 66.3 (8.9) years at blood draw, with 330 deaths during an average follow-up of 7.8 years. The WHI cohort, which enrolled postmenopausal women 50–79 years in age, consisted of three ethnic groups: 47% of European ancestry, 32% African Americans and 20% of Hispanic ancestry. These groups exhibited similar age distributions, with a mean (s.d.) age of 65.4 (7.1) years, and a mean (s.d.) follow-up time of 16.9 (4.6) years. During the follow-up, 765 women died.

#### Mortality analysis for time to death

Our mortality analysis was performed as follows. First, we applied our Array Converter algorithm (Supplementary Note 5) to yield the imputed mammalian arrays and to estimate DNAmAge values based on our universal clocks. Second, we computed AgeAccel for each cohort. Third, we applied Cox regression analysis for time to death (as a dependent variable) to assess the predictive ability of our universal clocks for all-cause mortality. The analysis was adjusted for age at blood draw and for sex in the FHS. We stratified the WHI cohort by ethnic or racial groups and combined a total of four results across FHS and WHI cohorts by fixed-effect models weighted by inverse variance. The meta-analysis was performed with the R ‘metafor’ function.

#### Human epidemiological cohort studies for lifestyle factors

We performed a robust correlation analysis (bicor^29^) between (1) our AgeAccel measures from clocks 2 and 3 and (2) 59 variables spanning diet, clinically relevant measurements and lifestyle factors. Comprehensive details of these variables and our analytical approaches, inclusive of meta-analysis, are elucidated in Supplementary Note 5.

#### Polygenic models for heritability analysis

We calculated the narrow-sense heritability of our clocks by employing polygenic models as defined in SOLAR^90^ and its R interface solarius^91^ as detailed in Supplementary Note 5.

#### OSKM reprogramming cells in human dermal fibroblasts

We applied our universal clock 2 and clock 3 to a previously published dataset (GSE54848)^31^ in which the authors had transfected human dermal fibroblasts with the Yamanaka factors (OSKM) over a 49-d period. The successfully transformed cells were collected and profiled on the human Illumina 450k arrays. Similar to the applications for the FHS and WHI cohorts, we applied our Array Converter algorithm (Supplementary Note 5) to yield the imputed mammalian arrays and to estimate DNAmAge based on our universal clocks. The clocks were applied to a total of *n* = 27 samples across experiment days 0, 3, 7, 11, 15, 20, 28, 35, 42 and 49, respectively.

### Murine anti-aging studies

None of the samples from the murine anti-aging studies were used in the training set of the universal clocks, that is, these are truly independent test data. Clocks 2 and 3 were evaluated in five mouse experiments (independent test data): (1) Snell dwarf mice (*n* = 95), (2) GHRKO experiment 1 (GHRKO, *n* = 71 samples), (3) GHRKO experiment 2 (*n* = 96 samples), (4) three Tet experiments: *Tet1* KO (*n* = 64), *Tet2* KO (*n* = 65) and *Tet3* KO (*n* = 63) and (5) CR (*n* = 95). Details can be found in Supplementary Note 6.

### Meta-analysis for EWAS of age

In our primary EWAS of age, we focused on samples from eutherians (*n* = 65 species) for which each species has at least 15 samples from the same tissue type. In secondary analyses, we also studied aging effects in marsupials (*n* = 4 marsupial species that had at least ten same-tissue type samples) and monotremes (only *n* = 2 species). Data distribution was assumed to be normal, but this was not formally tested.

Our meta-analysis for EWAS of age in eutherian species combined Pearson correlation test statistics across species–tissue strata that contained at least 15 samples each. The minimum sample size requirement resulted in 143 species–tissue strata from 65 eutherian species (Supplementary Data 1.5). To counter the dependency patterns resulting from multiple tissues from the same species, the meta-analysis was carried out in two steps. First, we meta-analyzed the EWAS of different tissues for each species separately. These tissue-specific summary statistics were combined within the same species to represent the EWAS results at species level. Second, we meta-analyzed the resulting 65-species EWAS results across species to arrive at the final meta-EWAS of age. In each meta-analysis step, we used the unweighted Stouffer’s method as implemented in R. In more detail, we gathered 68 blood samples from 27 distinct lemur species and 23 skin samples from 23 distinct lemur species, each species–tissue stratum with at most three samples. We therefore combined those 68 blood samples to perform blood EWAS in lemurs. Similarly, we combined the 23 skin samples for skin EWAS in lemurs. As listed in Supplementary Data 1.5, the combined species in lemurs was denoted by Strepsirrhine in the column ‘Species Latin Name’.

EWAS of age in marsupials was based on a two-step meta-analysis in which we relaxed the threshold of sample size in the species–tissue category to *n* ≥ 10 (Supplementary Data 1.12). Due to a small sample size in monotremes (*n* = 15), we combined all monotreme samples into a single dataset.

#### Brain EWAS

We applied the two-step meta-analysis approach to the brain EWAS results based on more than 900 brain tissues (cerebellum, cortex, hippocampus, hypothalamus, striatum, subventricular zone and whole brain) from eight species including human, vervet monkey, mice, olive baboon, brown rat and pig species (Supplementary Data 1.6).

#### EWAS of a single tissue

For the cerebral cortex brain region, we simply combined tissue-specific EWAS results across different species using the unweighted Stouffer’s method (Supplementary Data 1.7). Similarly, we carried out the one-step meta-analysis EWAS of blood, liver, muscle and skin (Supplementary Data 1.8–1.11). Details can be found in Supplementary Note 5.

All the Manhattan plots were generated based on a modified version of the gmirror function in R.

#### Stratification by age groups

To assess whether the age-related CpG sites in young animals relate to those in old animals, we split the data into three age groups: young-age (age < 1.5ASM), middle-age (age between 1.5ASM and 3.5ASM) and old-age (age ≥ 3.5ASM) groups. The threshold of sample size in species–tissue was relaxed to *n* ≥ 10. The age correlations in each age group were meta-analyzed using the above-mentioned two-step meta-analysis approach.

### Polycomb repressive complex

Polycomb repressive complex annotations were defined based on the binding of at least two transcriptional factor members of polycomb repressor complex 1 (PRC1 with subgroups RING1, RNF2, BMI1) or PRC2 (with subgroups EED, SUZ12 and EZH2) in 49 available ChIP–seq datasets from ENCODE^53^.

We identified 640 and 5,287 CpG sites in the array that were located in regions bound by PRC1 and PRC2, respectively. We performed a one-sided hypergeometric analysis to study both enrichment (OR > 1) and depletion (OR < 1) patterns for our age-related markers based on the top 1,000 CpG sites increased with age and the top 1,000 CpG sites decreased with age from the EWAS of age.

### Universal chromatin state analysis

To annotate our age-related CpG sites based on chromatin states, we assigned a state for all our mammalian CpG sites based on a recently published universal ChromHMM chromatin state annotation of the human genome^46^. The underlying hidden Markov model was trained with over 1,000 datasets of 32 chromatin marks in more than 100 cell and tissue types. This model then produced a single chromatin state annotation per genomic position that is applicable across cell and tissue types, as opposed to producing an annotation that is specific to one cell or tissue type. A total of 100 distinct states were generated and categorized into 16 major groups according to the parameters of the model and external genome annotations^46^ (described in Supplementary Data 8.2).

We performed a one-sided hypergeometric analysis to study both enrichment (OR > 1) and depletion (OR < 1) patterns for our age-related markers based on the top 1,000 CpG sites with a positive correlation with age and the top 1,000 CpG sites with a negative correlation with age across different eutherian species.

### Analysis of late-replicating domains

The annotation of late-replicating domains (hg19 and mm10) was obtained from Zhou et al.^50^, as described in Supplementary Note 5.

### GREAT enrichment analysis

We applied the GREAT analysis software tool^51^ to the top 1,000 positively age-related and the top 1,000 negatively age-related CpG sites from the EWAS of age. GREAT implemented foreground–background hypergeometric tests over genomic regions where we input all CpG sites of the mammalian array as background and the genomic regions of the 1,000 CpG sites as foreground. This approach yielded hypergeometric *P* values that were not confounded by the number of CpG sites within a gene (Supplementary Note 5).

### EWAS–TWAS overlap analysis

Our EWAS–TWAS-based overlap analysis related the gene sets found by our EWAS of age with the gene sets from our in-house TWAS database. The TWAS database, along with our analytical approaches, is described in Supplementary Note 5.

### EWAS–GWAS overlap analysis

Our EWAS–GWAS overlap analysis linked the gene sets discovered in our EWAS of age with those identified in published large-scale GWAS studies of various phenotypes (Supplementary Note 5).

### Transcription factor binding analysis

We used the CellBase database^52^, incorporating ENCODE^53^ TF binding sites for our analysis (Supplementary Note 5).

### Single-cell ATAC-seq of human bone marrow

Recent advances have enabled the sequencing of ATAC profiles within single cells, enabling assessment of the proportion of cells containing an open chromatin region^58^. We cross-referenced the top 35 CpG sites with positive age correlation across mammalian tissues with publicly available scATAC-seq data (Supplementary Table 3). We downloaded 10x Multiome count data in AnnData format as H5AD from the Gene Expression Omnibus (accession number GSE194122). The ATAC array data were managed using the Python package anndata^92^. hg38 ATAC peak locations were extracted from the metadata ‘.var’ section using anndata. Peak locations were overlapped with probe locations using GenomicRanges^93^ for the top 35 CpG sites. The overlapping peaks were then used to extract the processed counts for each cell. The proportion of cells containing an ATAC peak for each individual sample was calculated. A correlation was calculated by comparing this value against the age of each individual sample. The cell type for each barcode was extracted from the observable object. We subsequently computed the proportion of each cell type containing an ATAC peak in one of the seven significantly correlated regions (*LHFPL4*, *TLX3*, *ZIC2*, *PAX2*, *NR2E1*, *NEUROD1* and *DLX6-AS1*). Progenitor cells were grouped as MK/E progenitors, G/M progenitors, lymph progenitors and proerythroblasts, and differentiated cells were grouped as CD14^+^ monocytes, CD16^+^ monocytes, CD8^+^ T naive, CD8^+^ T, CD4^+^ T naive, CD4^+^ T activated, naive CD20^+^ B, B1 B, transitional B and NK. The percentage of each of the three populations (HSC, progenitor and differentiated cells) was calculated, and the proportion of cells containing an ATAC peak in one of the seven significantly correlated regions was calculated. To confirm enrichment for the hypermethylated sites showing decrease in chromatin accessibility with age, we randomly selected 1,000 sets of 17 ATAC peaks and compared the mean correlation with age of the selected regions to the 1,000 sampled sets of regions.

#### Mouse single-cell ATAC-seq in hematopoietic stem cells

We downloaded the publicly available data (H5, meta and fragment files of Illumina HiSeq 1500 array data) from Itokawa et al.^59^ (GSE162662).

scATAC-seq data were profiled in four biological replicates in young (10-week) and old (20-month) mice. The ATAC-seq data were managed and analyzed with R Signac^94^. We applied Fisher’s exact test to ascertain whether locations with differential accessibility between young and old animals were enriched with the 33 top positively age-related CpG sites (OR > 1 indicates a higher proportion in the old group). Further analytical details, including ATAC-seq data quality controls, are presented in Supplementary Note 5.

### URLs

The following URLs are available: AnAge (https://genomics.senescence.info/species/index.html), GREAT (http://great.stanford.edu/public/html/), late-replicating domains (https://zwdzwd.github.io/pmd), UCSC Genome Browser (http://genome.ucsc.edu/index.html).

### Reporting summary

Further information on research design is available in the Nature Portfolio Reporting Summary linked to this article.

## Supplementary information

Supplementary Information Supplementary Tables 1–3 and Notes 1–6.

Reporting Summary

Supplementary Data 1–14.

Supplementary Code

## Data Availability

The individual-level data from the Mammalian Methylation Consortium can be accessed from several online locations. All data from the Mammalian Methylation Consortium are posted on Gene Expression Omnibus (complete dataset, GSE223748). Subsets of the datasets can also be downloaded from accession numbers GSE174758, GSE184211, GSE184213, GSE184215, GSE184216, GSE184218, GSE184220, GSE184221, GSE184224, GSE190660, GSE190661, GSE190662, GSE190663, GSE190664, GSE174544, GSE190665, GSE174767, GSE184222, GSE184223, GSE174777, GSE174778, GSE173330, GSE164127, GSE147002, GSE147003, GSE147004, GSE223943 and GSE223944. Additional details can be found in Supplementary Note 2. The mammalian data can also be downloaded from the Clock Foundation webpage: https://clockfoundation.org/MammalianMethylationConsortium. The mammalian methylation array is available through the non-profit Epigenetic Clock Development Foundation (https://clockfoundation.org/). The manifest file of the mammalian array and genome annotations of CpG sites can be found on Zenodo (10.5281/zenodo.7574747). All other data supporting the findings of this study are available from the corresponding author upon reasonable request.

## Code availability

The chip manifest files, genome annotations of CpG sites and the software code for universal pan-mammalian clocks can be found on GitHub^95^ at https://github.com/shorvath/MammalianMethylationConsortium/tree/v2.0.0. The individual R code for the universal pan-mammalian clocks, EWAS analysis and functional enrichment studies can be also found in the Supplementary Code.
