# Author Correction: Universal DNA methylation age across mammalian tissues

**DOI:** 10.1038/s43587-023-00499-7

**Published:** 2023-09-06

**Authors:** A. T. Lu, Z. Fei, A. Haghani, T. R. Robeck, J. A. Zoller, C. Z. Li, R. Lowe, Q. Yan, J. Zhang, H. Vu, J. Ablaeva, V. A. Acosta-Rodriguez, D. M. Adams, J. Almunia, A. Aloysius, R. Ardehali, A. Arneson, C. S. Baker, G. Banks, K. Belov, N. C. Bennett, P. Black, D. T. Blumstein, E. K. Bors, C. E. Breeze, R. T. Brooke, J. L. Brown, G. G. Carter, A. Caulton, J. M. Cavin, L. Chakrabarti, I. Chatzistamou, H. Chen, K. Cheng, P. Chiavellini, O. W. Choi, S. M. Clarke, L. N. Cooper, M. L. Cossette, J. Day, J. DeYoung, S. DiRocco, C. Dold, E. E. Ehmke, C. K. Emmons, S. Emmrich, E. Erbay, C. Erlacher-Reid, C. G. Faulkes, S. H. Ferguson, C. J. Finno, J. E. Flower, J. M. Gaillard, E. Garde, L. Gerber, V. N. Gladyshev, V. Gorbunova, R. G. Goya, M. J. Grant, C. B. Green, E. N. Hales, M. B. Hanson, D. W. Hart, M. Haulena, K. Herrick, A. N. Hogan, C. J. Hogg, T. A. Hore, T. Huang, J. C. Izpisua Belmonte, A. J. Jasinska, G. Jones, E. Jourdain, O. Kashpur, H. Katcher, E. Katsumata, V. Kaza, H. Kiaris, M. S. Kobor, P. Kordowitzki, W. R. Koski, M. Krützen, S. B. Kwon, B. Larison, S. G. Lee, M. Lehmann, J. F. Lemaitre, A. J. Levine, C. Li, X. Li, A. R. Lim, D. T. S. Lin, D. M. Lindemann, T. J. Little, N. Macoretta, D. Maddox, C. O. Matkin, J. A. Mattison, M. McClure, J. Mergl, J. J. Meudt, G. A. Montano, K. Mozhui, J. Munshi-South, A. Naderi, M. Nagy, P. Narayan, P. W. Nathanielsz, N. B. Nguyen, C. Niehrs, J. K. O’Brien, P. O’Tierney Ginn, D. T. Odom, A. G. Ophir, S. Osborn, E. A. Ostrander, K. M. Parsons, K. C. Paul, M. Pellegrini, K. J. Peters, A. B. Pedersen, J. L. Petersen, D. W. Pietersen, G. M. Pinho, J. Plassais, J. R. Poganik, N. A. Prado, P. Reddy, B. Rey, B. R. Ritz, J. Robbins, M. Rodriguez, J. Russell, E. Rydkina, L. L. Sailer, A. B. Salmon, A. Sanghavi, K. M. Schachtschneider, D. Schmitt, T. Schmitt, L. Schomacher, L. B. Schook, K. E. Sears, A. W. Seifert, A. Seluanov, A. B. A. Shafer, D. Shanmuganayagam, A. V. Shindyapina, M. Simmons, K. Singh, I. Sinha, J. Slone, R. G. Snell, E. Soltanmaohammadi, M. L. Spangler, M. C. Spriggs, L. Staggs, N. Stedman, K. J. Steinman, D. T. Stewart, V. J. Sugrue, B. Szladovits, J. S. Takahashi, M. Takasugi, E. C. Teeling, M. J. Thompson, B. Van Bonn, S. C. Vernes, D. Villar, H. V. Vinters, M. C. Wallingford, N. Wang, R. K. Wayne, G. S. Wilkinson, C. K. Williams, R. W. Williams, X. W. Yang, M. Yao, B. G. Young, B. Zhang, Z. Zhang, P. Zhao, Y. Zhao, W. Zhou, J. Zimmermann, J. Ernst, K. Raj, S. Horvath

**Affiliations:** 1 Department of Human Genetics, David Geffen School of Medicine, University of California, Los Angeles, Los Angeles, CA USA; 2 Altos Labs, San Diego Institute of Science, San Diego, CA USA; 3 Department of Biostatistics, Fielding School of Public Health, University of California, Los Angeles, Los Angeles, CA USA; 4 Department of Statistics, University of California, Riverside, Riverside, CA USA; 5 Zoological SeaWorld Parks and Entertainment, Orlando, FL USA; 6 Altos Labs, Cambridge Institute of Science, Cambridge, UK; 7 Bioinformatics Interdepartmental Program, University of California, Los Angeles, CA USA; 8 Department of Biological Chemistry, University of California, Los Angeles, Los Angeles, CA USA; 9 Department of Biology, University of Rochester, Rochester, NY USA; 10 Department of Neuroscience, Peter O’Donnell Jr. Brain Institute, University of Texas Southwestern Medical Center, Dallas, TX USA; 11 Department of Biology, University of Maryland, College Park, MD USA; 12 Loro Parque Fundacion, Puerto de la Cruz, Spain; 13 Department of Biology, University of Kentucky, Lexington, KY USA; 14 Division of Cardiology, Department of Internal Medicine, David Geffen School of Medicine, University of California, Los Angeles, Los Angeles, CA USA; 15 Marine Mammal Institute, Oregon State University, Newport, OR USA; 16 School of Science and Technology, Clifton Campus, Nottingham Trent University, Nottingham, UK; 17 School of Life and Environmental Sciences, the University of Sydney, Sydney, New South Wales Australia; 18 Department of Zoology and Entomology, University of Pretoria, Hatfield, South Africa; 19 Busch Gardens Tampa, Tampa, FL USA; 20 Department of Ecology and Evolutionary Biology, University of California, Los Angeles, Los Angeles, CA USA; 21 Rocky Mountain Biological Laboratory, Crested Butte, CO USA; 22 Altius Institute for Biomedical Sciences, Seattle, WA USA; 23 Epigenetic Clock Development Foundation, Los Angeles, CA USA; 24 Center for Species Survival, Smithsonian Conservation Biology Institute, Front Royal, VA USA; 25 Department of Evolution, Ecology and Organismal Biology, The Ohio State University, Columbus, OH USA; 26 AgResearch, Invermay Agricultural Centre, Mosgiel, New Zealand; 27 Department of Biochemistry, University of Otago, Dunedin, New Zealand; 28 Gulf World, Dolphin Company, Panama City Beach, FL USA; 29 School of Veterinary Medicine and Science, University of Nottingham, Nottingham, UK; 30 Department of Pathology, Microbiology and Immunology, School of Medicine, University of South Carolina, Columbia, SC USA; 31 Department of Pharmacology, Addiction Science and Toxicology, the University of Tennessee Health Science Center, Memphis, TN USA; 32 Medical Informatics, David Geffen School of Medicine, University of California, Los Angeles, Los Angeles, CA USA; 33 Biochemistry Research Institute of La Plata, Histology and Pathology, School of Medicine, University of La Plata, La Plata, Argentina; 34 Center for Neurobehavioral Genetics, Semel Institute for Neuroscience and Human Behavior, Department of Psychiatry and Biobehavioral Sciences, David Geffen School of Medicine, University of California, Los Angeles, Los Angeles, CA USA; 35 Department of Anatomy and Neurobiology, Northeast Ohio Medical University, Rootstown, OH USA; 36 Department of Environmental and Life Sciences, Trent University, Peterborough, Ontario Canada; 37 Taronga Institute of Science and Learning, Taronga Conservation Society Australia, Mosman, New South Wales Australia; 38 SeaWorld of Florida, Orlando, FL USA; 39 Zoological Operations, SeaWorld Parks and Entertainment, Orlando, FL USA; 40 Duke Lemur Center, Durham, NC USA; 41 Conservation Biology Division, Northwest Fisheries Science Center, National Marine Fisheries Service, National Oceanic and Atmospheric Administration, Seattle, WA USA; 42 Departments of Biology and Medicine, University of Rochester, Rochester, NY USA; 43 Altos Labs, San Francisco, CA USA; 44 SeaWorld Orlando, Orlando, FL USA; 45 School of Biological and Behavioural Sciences, Queen Mary University of London, London, UK; 46 Fisheries and Oceans Canada, Freshwater Institute, Winnipeg, Manitoba Canada; 47 Department of Biological Sciences, University of Manitoba, Winnipeg, Manitoba Canada; 48 Department of Population Health and Reproduction, University of California, Davis School of Veterinary Medicine, Davis, CA USA; 49 Mystic Aquarium, Mystic, CT USA; 50 Universite de Lyon, Universite Lyon 1, CNRS, Laboratoire de Biometrie et Biologie Evolutive, Villeurbanne, France; 51 Greenland Institute of Natural Resources, Nuuk, Greenland; 52 Evolution and Ecology Research Centre, School of Biological, Earth and Environmental Sciences, UNSW Sydney, Sydney, New South Wales Australia; 53 Division of Genetics, Department of Medicine, Brigham and Women’s Hospital, Harvard Medical School, Boston, MA USA; 54 Applied Translational Genetics Group, School of Biological Sciences, Centre for Brain Research, the University of Auckland, Auckland, New Zealand; 55 Vancouver Aquarium, Vancouver, British Columbia Canada; 56 SeaWorld of California, San Diego, CA USA; 57 Cancer Genetics and Comparative Genomics Branch, National Human Genome Research Institute, National Institutes of Health, Bethesda, MD USA; 58 Department of Anatomy, University of Otago, Dunedin, New Zealand; 59 Division of Human Genetics, Department of Pediatrics, University at Buffalo, Buffalo, NY USA; 60 Division of Genetics and Metabolism, Oishei Children’s Hospital, Buffalo, NY USA; 61 School of Biological Sciences, University of Bristol, Bristol, UK; 62 Norwegian Orca Survey, Andenes, Norway; 63 Mother Infant Research Institute, Tufts Medical Center, Boston, MA USA; 64 Yuvan Research, Mountain View, CA USA; 65 Kamogawa Sea World, Kamogawa, Japan; 66 Peromyscus Genetic Stock Center, University of South Carolina, Columbia, SC USA; 67 Department of Drug Discovery and Biomedical Sciences, College of Pharmacy, University of South Carolina, Columbia, SC USA; 68 Edwin S.H. Leong Healthy Aging Program, Centre for Molecular Medicine and Therapeutics, University of British Columbia, Vancouver, British Columbia Canada; 69 Institute of Animal Reproduction and Food Research of the Polish Academy of Sciences, Olsztyn, Poland; 70 Institute for Veterinary Medicine, Nicolaus Copernicus University, Torun, Poland; 71 LGL Limited, King City, Ontario Canada; 72 Evolutionary Genetics Group, Department of Evolutionary Anthropology, University of Zurich, Zurich, Switzerland; 73 Department of Ecology and Evolutionary Biology, UCLA, Los Angeles, CA USA; 74 Center for Tropical Research, Institute for the Environment and Sustainability, UCLA, Los Angeles, CA USA; 75 Department of Neurology, David Geffen School of Medicine, University of California, Los Angeles, Los Angeles, CA USA; 76 Texas Pregnancy and Life-course Health Center, Southwest National Primate Research Center, San Antonio, TX USA; 77 Department of Animal Science, College of Agriculture and Natural Resources, Laramie, WY USA; 78 Technology Center for Genomics and Bioinformatics, Department of Pathology and Laboratory Medicine, University of California, Los Angeles, Los Angeles, CA USA; 79 Centre for Molecular Medicine and Therapeutics, BC Children’s Hospital Research Institute, University of British Columbia, Vancouver, British Columbia Canada; 80 Institute of Ecology and Evolution, School of Biological Sciences, University of Edinburgh, Edinburgh, UK; 81 White Oak Conservation, Yulee, FL USA; 82 North Gulf Oceanic Society, Homer, AK USA; 83 Translational Gerontology Branch, National Institute on Aging Intramural Research Program, National Institutes of Health, Baltimore, MD USA; 84 ABS Global, DeForest, WI USA; 85 Marineland of Canada, Niagara Falls, Ontario Canada; 86 Biomedical and Genomic Research Group, Department of Animal and Dairy Sciences, University of Wisconsin—Madison, Madison, WI USA; 87 Department of Preventive Medicine, University of Tennessee Health Science Center, College of Medicine, Memphis, TN USA; 88 Department of Genetics, Genomics and Informatics, University of Tennessee Health Science Center, College of Medicine, Memphis, TN USA; 89 Louis Calder Center—Biological Field Station, Department of Biological Sciences, Fordham University, Armonk, NY USA; 90 Museum fur Naturkunde, Leibniz Institute for Evolution and Biodiversity Science, Berlin, Germany; 91 Institute of Molecular Biology, Mainz, Germany; 92 Division of Molecular Embryology, DKFZ-ZMBH Alliance, Heidelberg, Germany; 93 Department of Obstetrics and Gynecology, Tufts University School of Medicine, Boston, MA USA; 94 Cancer Research UK Cambridge Institute, University of Cambridge, Cambridge, UK; 95 Division of Regulatory Genomics and Cancer Evolution, Deutsches Krebsforschungszentrum, Heidelberg, Germany; 96 Department of Psychology, Cornell University, Ithaca, NY USA; 97 SeaWorld of Texas, San Antonio, TX USA; 98 Department of Molecular Cell and Developmental Biology, University of California, Los Angeles, Los Angeles, CA USA; 99 School of Earth, Atmospheric and Life Sciences, University of Wollongong, Wollongong, Australia; 100 Institute of Evolutionary Biology, School of Biological Sciences, University of Edinburgh, Edinburgh, UK; 101 Department of Animal Science, University of Nebraska, Lincoln, NE USA; 102 Mammal Research Institute, Department of Zoology and Entomology, University of Pretoria, Hatfield, South Africa; 103 Department of Biology, College of Arts and Science, Adelphi University, Garden City, NY USA; 104 Salk Institute for Biological Studies, La Jolla, CA USA; 105 Department of Epidemiology, UCLA Fielding School of Public Health, Los Angeles, CA USA; 106 Department of Environmental Health Sciences, UCLA Fielding School of Public Health, Los Angeles, CA USA; 107 Department of Neurology, UCLA David Geffen School of Medicine, Los Angeles, CA USA; 108 Center for Coastal Studies, Provincetown, MA USA; 109 Miami Seaquarium, Miami, FL USA; 110 The Sam and Ann Barshop Institute for Longevity and Aging Studies and Department of Molecular Medicine, UT Health San Antonio and the Geriatric Research Education and Clinical Center, South Texas Veterans Healthcare System, San Antonio, TX USA; 111 Department of Radiology, University of Illinois at Chicago, Chicago, IL USA; 112 Department of Biochemistry and Molecular Genetics, University of Illinois at Chicago, Chicago, IL USA; 113 National Center for Supercomputing Applications, University of Illinois at Urbana-Champaign, Urbana, IL USA; 114 College of Agriculture, Missouri State University, Springfield, MO USA; 115 Department of Animal Sciences, University of Illinois at Urbana-Champaign, Champaign, IL USA; 116 Department of Forensic Science, Environmental and Life Sciences, Trent University, Peterborough, Ontario Canada; 117 Department of Surgery, University of Wisconsin School of Medicine and Public Health, Madison, WI USA; 118 Shobhaben Pratapbhai Patel School of Pharmacy and Technology Management, SVKM’S NMIMS University, Mumbai, India; 119 Species Preservation Laboratory, SeaWorld San Diego, San Diego, CA USA; 120 Biology Department, Acadia University, Wolfville, Nova Scotia Canada; 121 Department of Pathobiology and Population Sciences, Royal Veterinary College, Hatfield, UK; 122 Howard Hughes Medical Institute, Department of Neuroscience, University of Texas Southwestern Medical Center, Dallas, TX USA; 123 School of Biology and Environmental Science, University College Dublin, Dublin, Ireland; 124 John G. Shedd Aquarium, Chicago, IL USA; 125 School of Biology, the University of St Andrews, Fife, UK; 126 Neurogenetics of Vocal Communication Group, Max Planck Institute for Psycholinguistics, Nijmegen, the Netherlands; 127 Blizard Institute, Faculty of Medicine and Dentistry, Queen Mary University of London, London, UK; 128 Department of Pathology and Laboratory Medicine, David Geffen School of Medicine at UCLA, Los Angeles, CA USA; 129 Division of Obstetrics and Gynecology, Tufts University School of Medicine, Boston, MA USA; 130 Center for Neurobehavioral Genetics, Jane and Terry Semel Institute for Neuroscience and Human Behavior, University of California, Los Angeles, Los Angeles, CA USA; 131 Department of Psychiatry and Biobehavioral Sciences, David Geffen School of Medicine at UCLA, Los Angeles, CA USA; 132 Fisheries and Oceans Canada, Winnipeg, Manitoba Canada; 133 Eli and Edythe Broad Center of Regenerative Medicine and Stem Cell Research, University of California, Los Angeles, CA USA; 134 Center for Computational and Genomic Medicine, Children’s Hospital of Philadelphia, Philadelphia, PA USA; 135 Department of Pathology and Laboratory Medicine, University of Pennsylvania, Philadelphia, PA USA; 136 Department of Mathematics and Technology, University of Applied Sciences Koblenz, Koblenz, Germany

**Keywords:** Evolution, Ageing, DNA methylation, Biomarkers

Correction to: *Nature Aging* 10.1038/s43587-023-00462-6, published online 10 August 2023.

In the version of Supplementary Data initially published with this article, data were missing from Table S1.13 (Updated AnAge version in Class Mammalia), which now appear in an updated Supplementary Data file in the online version of the article.

