# Supplementary material for: Universal DNA methylation age across mammalian tissues
Source: Nat Aging. 2023 Aug 10;3(9):1144–66. doi: 10.1038/s43587-023-00462-6 (PMC10501909; doi:10.1038/s43587-023-00462-6)
Supplement: Supplementary file 1 — Supplementary Tables 1–3 and Notes 1–6. [file 43587_2023_462_MOESM1_ESM.pdf]

nature aging

Resource

https://doi.org/10.1038/s43587-023-00462-6

Universal DNA methylation age across mammalian tissues

In the format provided by the authors and unedited

nature aging

Resource

https://doi.org/10.1038/s43587-023-00462-6

Universal DNA methylation age across mammalian tissues

In the format provided by the authors and unedited

Supplementary Figures

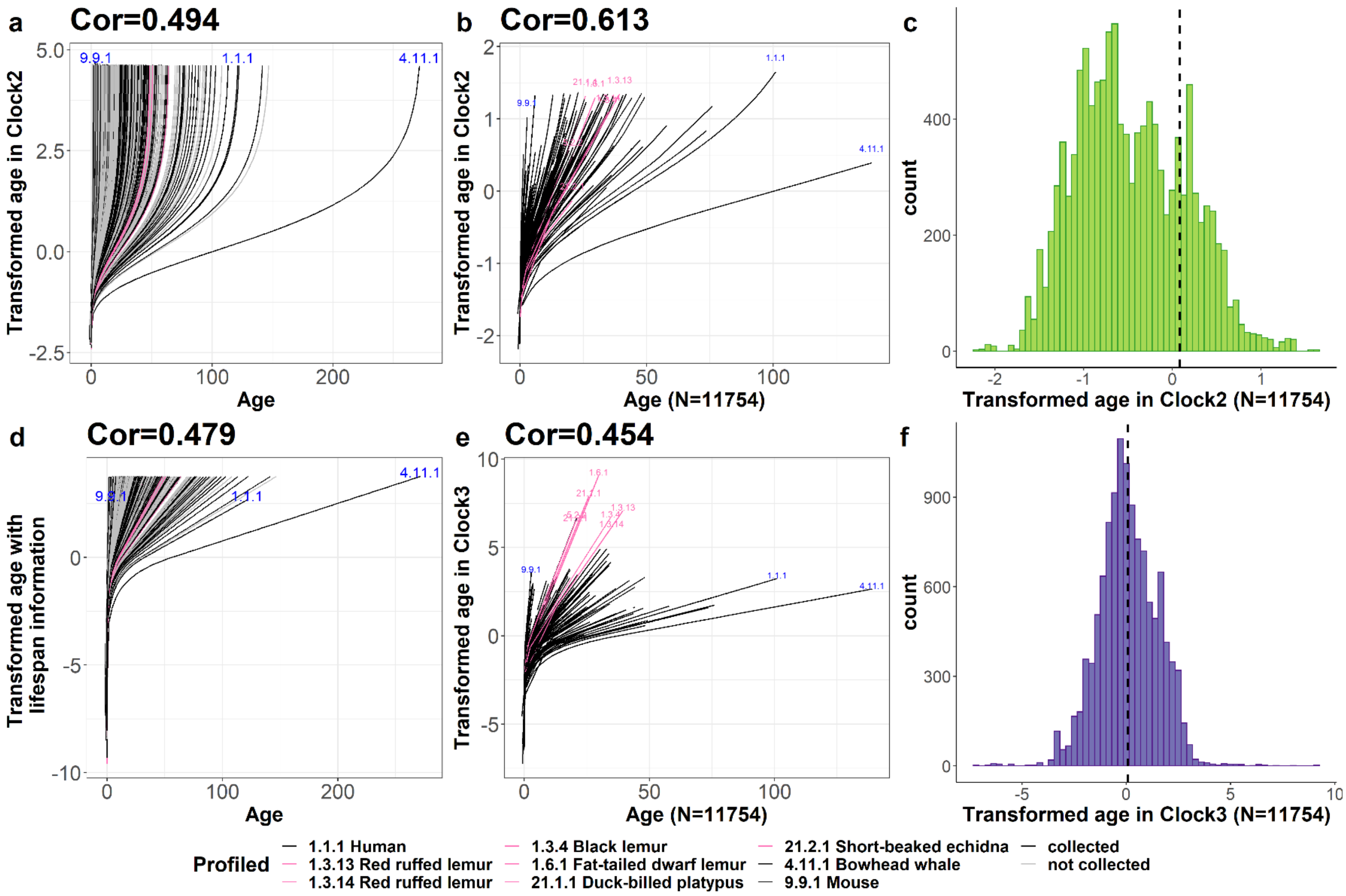

Extended Data Figure 1. Transformed age in universal clocks. The plot displays transformed age in universal Clock 2 (**a--c**) and universal Clock 3 (**d-f**). (**a, b**) Loglog transformation of Relative Age (y-axis) versus age in universal Clock 2 and (**d, e**) log-linear age (y-axis) versus age in our universal Clock 3. Of the 969 mammalian species with available gestation time, age at sexual maturity and maximum lifespan in AnAge database, 339 species are available in our collection. We multiplied the reported maximum lifespan of non-human or non-mouse species by 1.3. Transformed ages were calculated for all the 969 species with imputed age ranging from gestation time through the modified maximum lifespan. The columns (**a,d**) display all the 969 species with the imputed ages. In panel d, we proposed the log-linear age with the parameter $\boldsymbol{m}$ formulated with maximum lifespan as the information is available for all species ($\boldsymbol{m}^* = \boldsymbol{c_1} * \frac{MaxLifespan+GestationT}{ASM+GestationT}$ in Methods). Of the 339 species, 185 species with age information of high confidence and known tissue types were used in training universal clocks. The columns (**b, e**) empirically display these 185 species with the age variable (x-axis) based on the observed ages from all the samples in our collection (N=11,754). In panel **e**, we applied the log-linear age formulated without knowing maximum lifespan to train Clock 3 (formula (5) in Methods). Each line represents a species marked by gray for non-profiled and marked by black or pink for profiled species in our collection, as listed in the legend. Some species such as lemurs with relatively short gestation time in regressing $\boldsymbol{m}^*$ (formula (7) in Methods) exhibiting high log-linear ages in (**e**) are marked in pink. Each panel reports the Pearson correlation coefficient. (**c,f**) display the histograms of transformed ages based on all samples from the 187 species with vertical lines presenting at means.

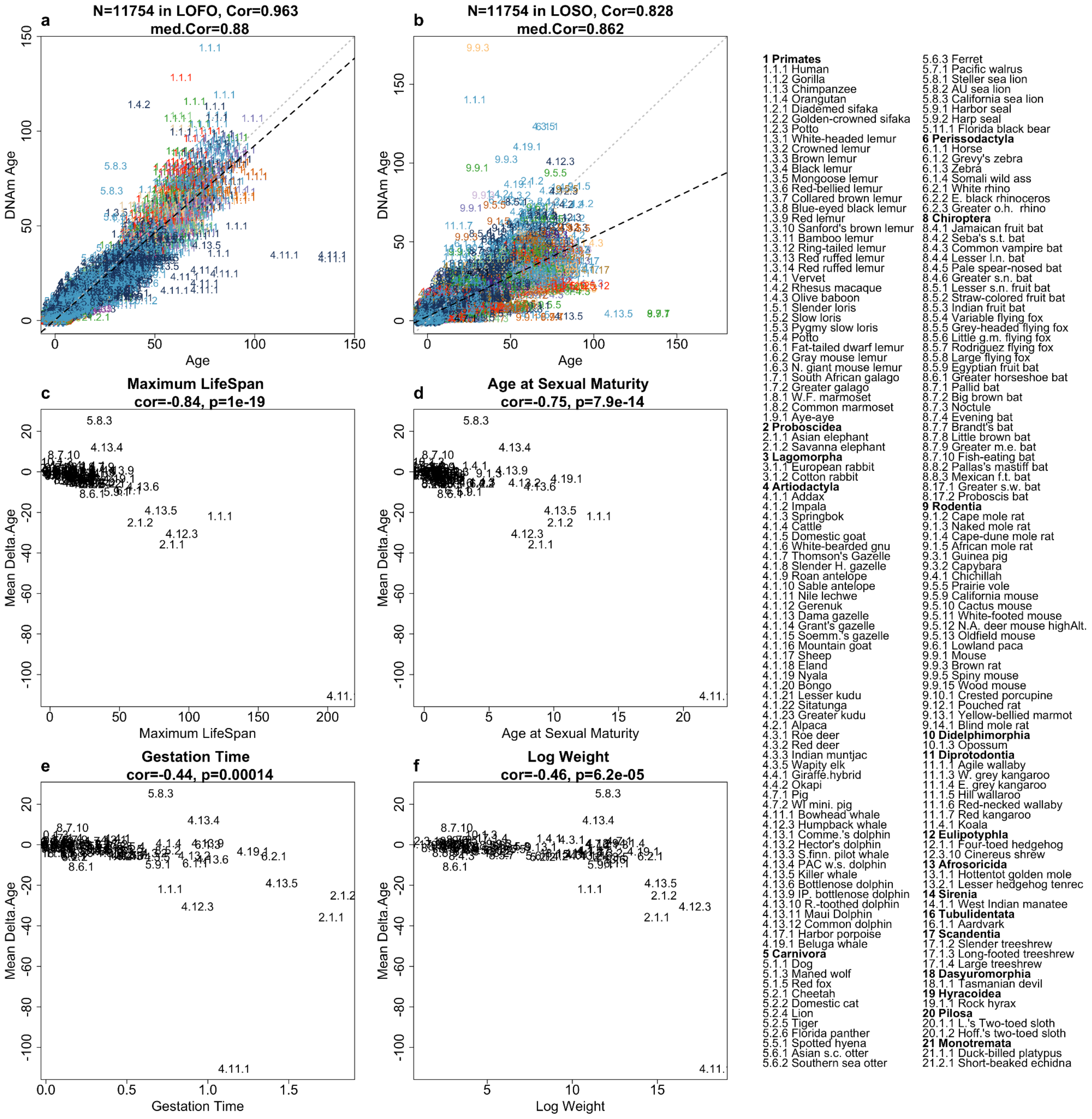
a
N=11754 in LOFO, Cor=0.963
med.Cor=0.88
DNAm Age
Age
b
N=11754 in LOSO, Cor=0.828
med.Cor=0.862
DNAm Age
Age
c
Maximum LifeSpan
cor=-0.84, p=1e-19
Mean Delta.Age
Maximum LifeSpan
d
Age at Sexual Maturity
cor=-0.75, p=7.9e-14
Mean Delta.Age
Age at Sexual Maturity
e
Gestation Time
cor=-0.44, p=0.00014
Mean Delta.Age
Gestation Time
f
Log Weight
cor=-0.46, p=6.2e-05
Mean Delta.Age
Log Weight
1 Primates
1.1.1 Human
1.1.2 Gorilla
1.1.3 Chimpanzee
1.1.4 Orangutan
1.2.1 Diademed sifaka
1.2.2 Golden-crowned sifaka
1.2.3 Potto
1.3.1 White-headed lemur
1.3.2 Crowned lemur
1.3.3 Brown lemur
1.3.4 Black lemur
1.3.5 Mongoose lemur
1.3.6 Red-bellied lemur
1.3.7 Collared brown lemur
1.3.8 Blue-eyed black lemur
1.3.9 Red lemur
1.3.10 Sanford's brown lemur
1.3.11 Bamboo lemur
1.3.12 Ring-tailed lemur
1.3.13 Red ruffed lemur
1.3.14 Red ruffed lemur
1.4.1 Vervet
1.4.2 Rhesus macaque
1.4.3 Olive baboon
1.5.1 Slender loris
1.5.2 Slow loris
1.5.3 Pygmy slow loris
1.5.4 Potto
1.6.1 Fat-tailed dwarf lemur
1.6.2 Gray mouse lemur
1.6.3 N. giant mouse lemur
1.7.1 South African galago
1.7.2 Greater galago
1.8.1 W.F. marmoset
1.8.2 Common marmoset
1.9.1 Aye-aye
2 Proboscidea
2.1.1 Asian elephant
2.1.2 Savanna elephant
3 Lagomorpha
3.1.1 European rabbit
3.1.2 Cotton rabbit
4 Artiodactyla
4.1.1 Addax
4.1.2 Impala
4.1.3 Springbok
4.1.4 Cattle
4.1.5 Domestic goat
4.1.6 White-bearded gnu
4.1.7 Thomson's Gazelle
4.1.8 Slender H. gazelle
4.1.9 Roan antelope
4.1.10 Sable antelope
4.1.11 Nile lechwe
4.1.12 Gerenuk
4.1.13 Dama gazelle
4.1.14 Grant's gazelle
4.1.15 Soemm.'s gazelle
4.1.16 Mountain goat
4.1.17 Sheep
4.1.18 Eland
4.1.19 Nyala
4.1.20 Bongo
4.1.21 Lesser kudu
4.1.22 Sitatunga
4.1.23 Greater kudu
4.2.1 Alpaca
4.3.1 Roe deer
4.3.2 Red deer
4.3.3 Indian muntjac
4.3.5 Wapity elk
4.4.1 Giraffe.hybrid
4.4.2 Okapi
4.7.1 Pig
4.7.2 WI mini. pig
4.11.1 Bowhead whale
4.12.3 Humpback whale
4.13.1 Comme.'s dolphin
4.13.2 Hector's dolphin
4.13.3 S.finn. pilot whale
4.13.4 PAC w.s. dolphin
4.13.5 Killer whale
4.13.6 Bottlenose dolphin
4.13.9 IP. bottlenose dolphin
4.13.10 R.-toothed dolphin
4.13.11 Maui Dolphin
4.13.12 Common dolphin
4.17.1 Harbor porpoise
4.19.1 Beluga whale
5 Carnivora
5.1.1 Dog
5.1.3 Maned wolf
5.1.5 Red fox
5.2.1 Cheetah
5.2.2 Domestic cat
5.2.4 Lion
5.2.5 Tiger
5.2.6 Florida panther
5.5.1 Spotted hyena
5.6.1 Asian s.c. otter
5.6.2 Southern sea otter
5.6.3 Ferret
5.7.1 Pacific walrus
5.8.1 Steller sea lion
5.8.2 AU sea lion
5.8.3 California sea lion
5.9.1 Harbor seal
5.9.2 Harp seal
5.11.1 Florida black bear
6 Perissodactyla
6.1.1 Horse
6.1.2 Grevy's zebra
6.1.3 Zebra
6.1.4 Somali wild ass
6.2.1 White rhino
6.2.2 E. black rhinoceros
6.2.3 Greater o.h. rhino
8 Chiroptera
8.4.1 Jamaican fruit bat
8.4.2 Seba's s.t. bat
8.4.3 Common vampire bat
8.4.4 Lesser l.n. bat
8.4.5 Pale spear-nosed bat
8.4.6 Greater s.n. bat
8.5.1 Lesser s.n. fruit bat
8.5.2 Straw-colored fruit bat
8.5.3 Indian fruit bat
8.5.4 Variable flying fox
8.5.5 Grey-headed flying fox
8.5.6 Little g.m. flying fox
8.5.7 Rodriguez flying fox
8.5.8 Large flying fox
8.5.9 Egyptian fruit bat
8.6.1 Greater horseshoe bat
8.7.1 Pallid bat
8.7.2 Big brown bat
8.7.3 Noctule
8.7.4 Evening bat
8.7.7 Brandt's bat
8.7.8 Little brown bat
8.7.9 Greater m.e. bat
8.7.10 Fish-eating bat
8.8.2 Pallas's mastiff bat
8.8.3 Mexican f.t. bat
8.17.1 Greater s.w. bat
8.17.2 Proboscis bat
9 Rodentia
9.1.2 Cape mole rat
9.1.3 Naked mole rat
9.1.4 Cape-dune mole rat
9.1.5 African mole rat
9.3.1 Guinea pig
9.3.2 Capybara
9.4.1 Chichillah
9.5.5 Prairie vole
9.5.9 California mouse
9.5.10 Cactus mouse
9.5.11 White-footed mouse
9.5.12 N.A. deer mouse highAlt.
9.5.13 Oldfield mouse
9.6.1 Lowland paca
9.9.1 Mouse
9.9.3 Brown rat
9.9.5 Spiny mouse
9.9.15 Wood mouse
9.10.1 Crested porcupine
9.12.1 Pouched rat
9.13.1 Yellow-bellied marmot
9.14.1 Blind mole rat
10 Didelphimorphia
10.1.3 Opossum
11 Diprotodontia
11.1.1 Agile wallaby
11.1.3 W. grey kangaroo
11.1.4 E. grey kangaroo
11.1.5 Hill wallaroo
11.1.6 Red-necked wallaby
11.1.7 Red kangaroo
11.4.1 Koala
12 Eulipotyphla
12.1.1 Four-toed hedgehog
12.3.10 Cinereus shrew
13 Afrosoricida
13.1.1 Hottentot golden mole
13.2.1 Lesser hedgehog tenrec
14 Sirenia
14.1.1 West Indian manatee
16 Tubulidentata
16.1.1 Aardvark
17 Scandentia
17.1.2 Slender treeshrew
17.1.3 Long-footed treeshrew
17.1.4 Large treeshrew
18 Dasyuromorphia
18.1.1 Tasmanian devil
19 Hyracoidea
19.1.1 Rock hyrax
20 Pilosa
20.1.1 L.'s Two-toed sloth
20.1.2 Hoff.'s two-toed sloth
21 Monotremata
21.1.1 Duck-billed platypus
21.2.1 Short-beaked echidna

Extended Data Figure 2. Basic universal clock for log-transformed age. a, b, Chronological age (x-axis) versus DNAmAge estimated using a, leave-one-fraction-out (LOFO) and b, leave-one-species-out (LOSO) analysis. The gray and black dashed lines correspond to the diagonal line (y=x) and the regression line, respectively. Each dot (tissue sample) is labeled by the mammalian species index (legend). The species index corresponds to the taxonomic order, e.g. 1=primates, 2=elephants (Proboscidea) etc. (legend). The numbers after the first and second decimal points enumerate the taxonomic family and species, respectively. Points are colored by tissue type (Data S1.4). The heading of each panel reports the Pearson correlation (cor) across all samples. Here med.Cor (or med.MAE) denotes the median value across species that contain at least 15 samples. **c–f**, The y-axis reports the mean difference between the LOSO estimate of DNAm age and chronological age evaluated at a fixed age defined as half the maximum lifespan (denoted as Mean Delta.Age). The scatter plots depict mean delta half lifespan per species (y-axis) versus c, maximum lifespan observed in the species, d, average age at sexual maturity e, gestational time (in units of years), and f, (log-transformed) average adult body mass in units of grams. All P-values reported are unadjusted and are based on two-sided tests.

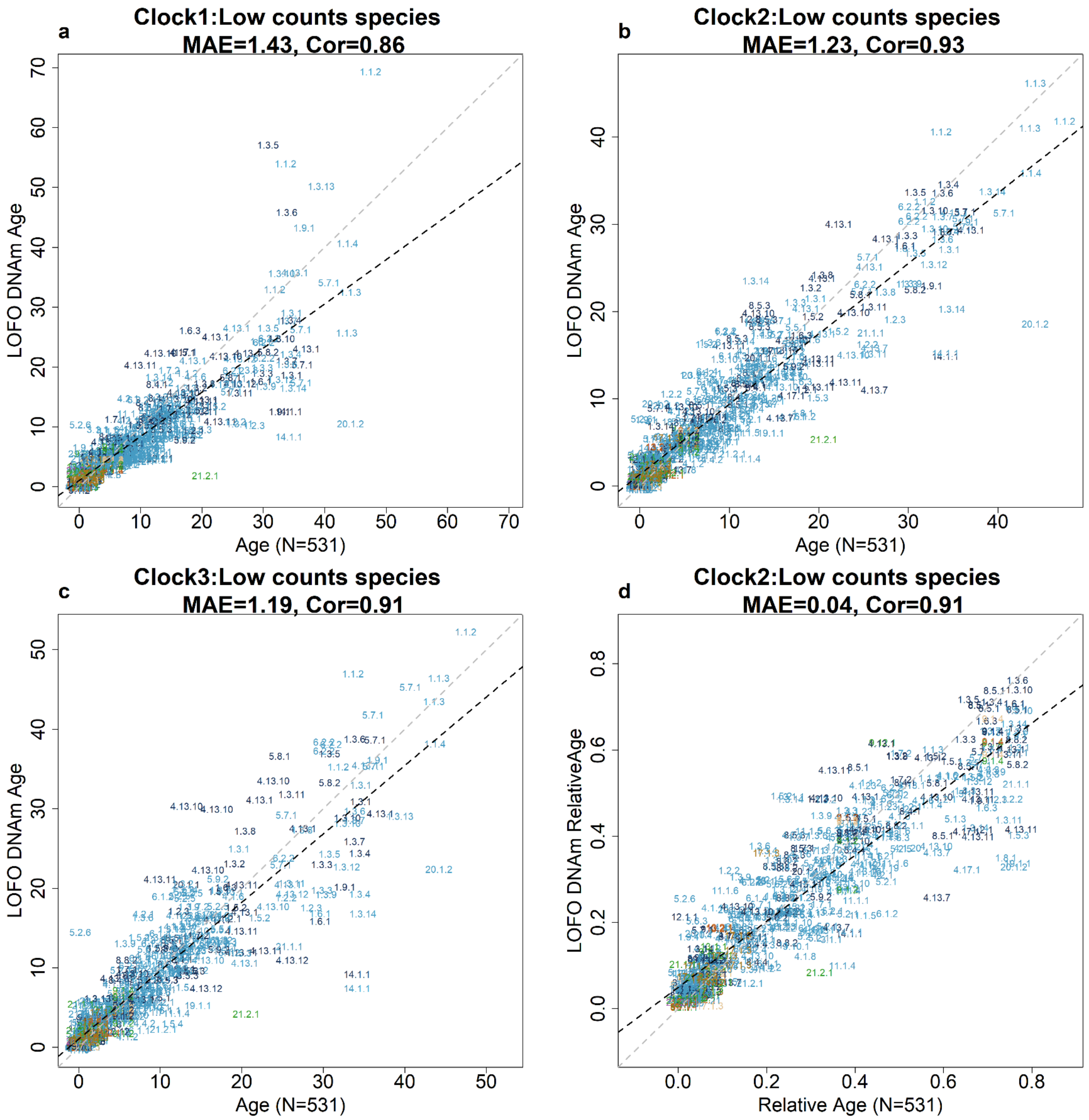

Extended Data Figure 3. Universal clocks applied to species with fewer than 15 samples. The title of each panel lists the type of universal clock: **a,** Clock 1=basic universal clock based on log(Age+2), **b,d**, Clock 2=universal clock for relative age, **c,** Clock 3 =universal clock for log-linear age. Leave-one-fraction-out (LOFO) methylation estimates versus a–c, chronological age or d, relative age. The respective inverse transformations were applied to arrive at DNA methylation-based estimates of chronological age in years or relative age (y-axis).

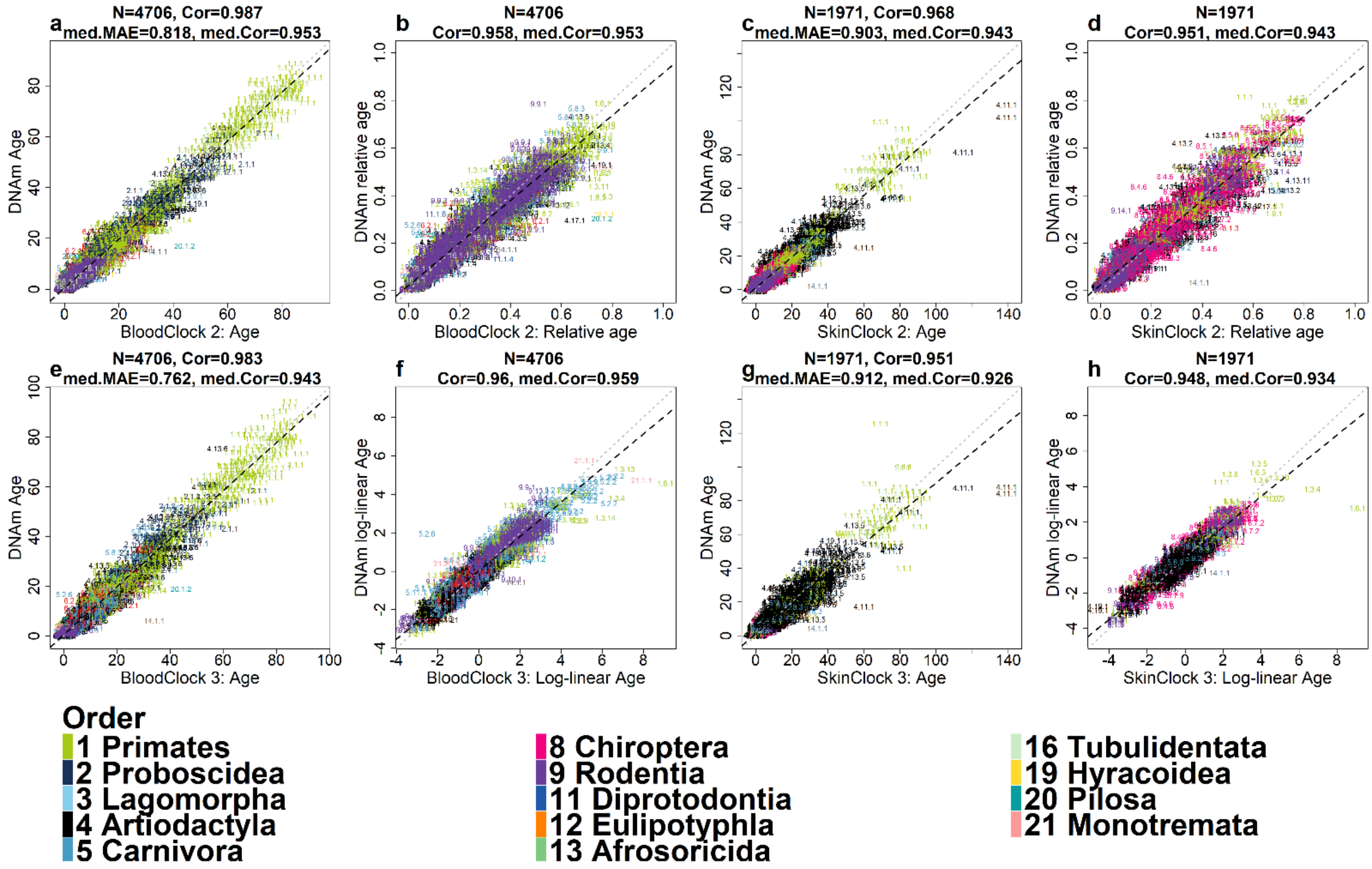

Extended Data Figure 4. Universal clocks for specific tissues (blood, skin). These tissue-specific universal clocks were constructed in an analogous fashion to the pan-tissue clocks described in the main text. The panels show leave-one-fraction-out (LOFO) estimates (y-axis) of four clocks: blood universal clock 2 (BloodClock 2) which estimates relative age, blood universal clock 3 (BloodClock 3) which estimates log-linear transformation of age. Analogously, we defined SkinClock2 and SkinClock3. Relative age estimation incorporates maximum lifespan and gestational age and assumes values between 0 and 1. Log-linear age is formulated with age at sexual maturity and gestational time. **a,c,e, g**, LOFO estimates of DNAm age (y-axis, in units of years) based on transforming relative age (Clock 2) or log-linear age (Clock 3). **b,f,d,h**, transformed age (x-axis) versus corresponding DNAm estimates (y-axis). The title of each panel reports the Pearson correlation coefficient across all data points and the median correlation (med.Cor) and median of median absolute error (med.MAE) across all species. Each sample is labeled by mammalian species index (explained in Figure 2) and colored by taxonomic order. The legend reports the taxonomic order and the mammalian order index as a prefix.

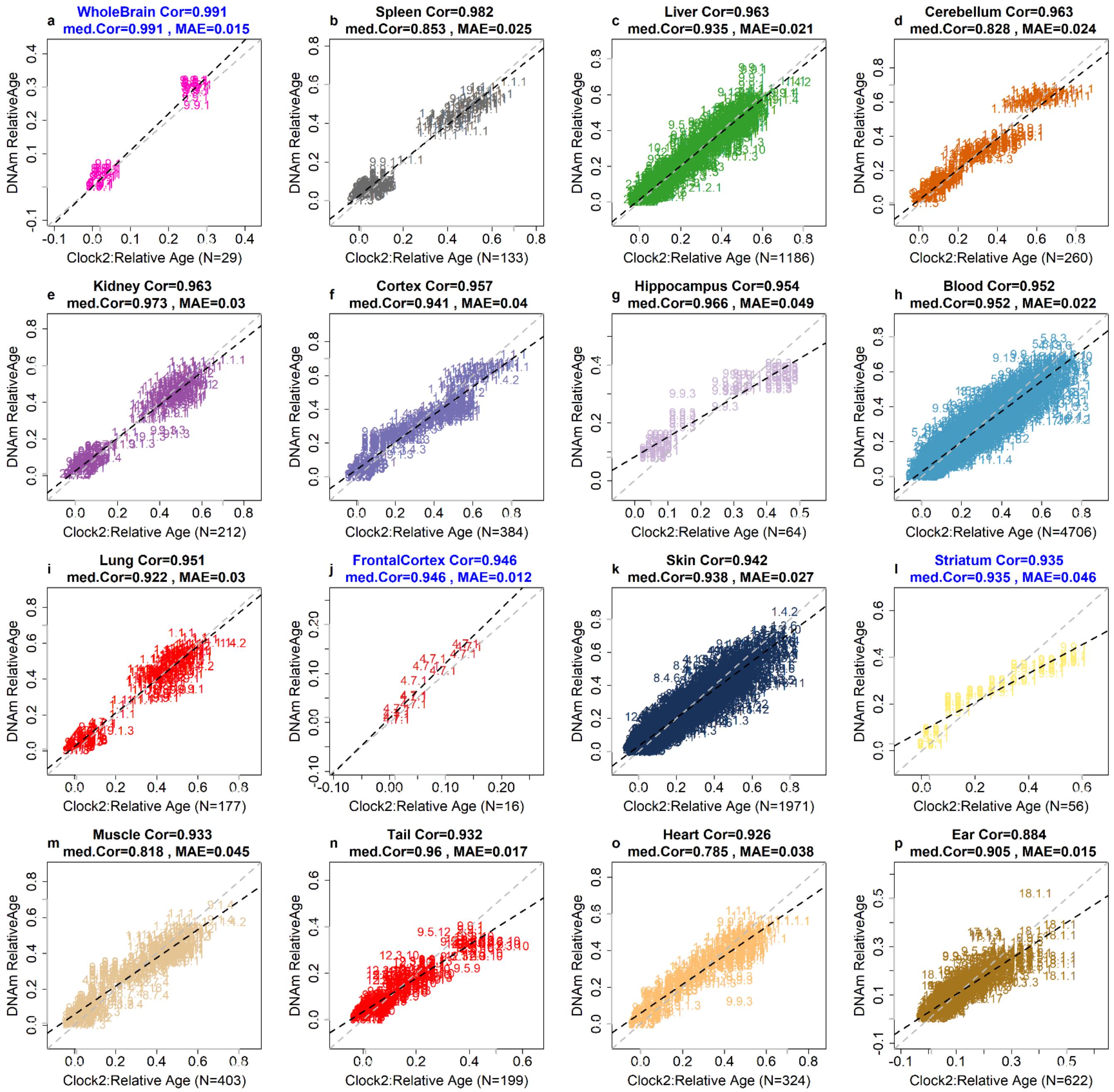

a WholeBrain Cor=0.991
med.Cor=0.991 , MAE=0.015
Clock2:Relative Age (N=29)
b Spleen Cor=0.982
med.Cor=0.853 , MAE=0.025
Clock2:Relative Age (N=133)
c Liver Cor=0.963
med.Cor=0.935 , MAE=0.021
Clock2:Relative Age (N=1186)
d Cerebellum Cor=0.963
med.Cor=0.828 , MAE=0.024
Clock2:Relative Age (N=260)
e Kidney Cor=0.963
med.Cor=0.973 , MAE=0.03
Clock2:Relative Age (N=212)
f Cortex Cor=0.957
med.Cor=0.941 , MAE=0.04
Clock2:Relative Age (N=384)
g Hippocampus Cor=0.954
med.Cor=0.966 , MAE=0.049
Clock2:Relative Age (N=64)
h Blood Cor=0.952
med.Cor=0.952 , MAE=0.022
Clock2:Relative Age (N=4706)
i Lung Cor=0.951
med.Cor=0.922 , MAE=0.03
Clock2:Relative Age (N=177)
j FrontalCortex Cor=0.946
med.Cor=0.946 , MAE=0.012
Clock2:Relative Age (N=16)
k Skin Cor=0.942
med.Cor=0.938 , MAE=0.027
Clock2:Relative Age (N=1971)
l Striatum Cor=0.935
med.Cor=0.935 , MAE=0.046
Clock2:Relative Age (N=56)
m Muscle Cor=0.933
med.Cor=0.818 , MAE=0.045
Clock2:Relative Age (N=403)
n Tail Cor=0.932
med.Cor=0.96 , MAE=0.017
Clock2:Relative Age (N=199)
o Heart Cor=0.926
med.Cor=0.785 , MAE=0.038
Clock2:Relative Age (N=324)
p Ear Cor=0.884
med.Cor=0.905 , MAE=0.015
Clock2:Relative Age (N=622)
DNAm RelativeAge

Extended Data Figure 5. Universal clock for relative age applied to specific tissues. DNA methylation-based estimates of relative age (y-axis) versus actual relative age (x-axis). The specific tissue or cell type is reported in the title of each panel. Each dot presents a tissue sample colored by tissue and labeled by mammalian species index (Data S1.3-1.4). The analysis is restricted to tissues that have at least 15 samples available. Leave-one-fraction-out cross-validation (LOFO) was used to arrive at unbiased estimates of predictive accuracy measures: median absolute error (MAE) and age correlation based on relative age. "Cor" denotes the Pearson correlation coefficient based on all available samples. "med.Cor" denotes the median values across all species for which at least 15 samples were available. Title is marked in blue if a tissue type was collected from a single species.

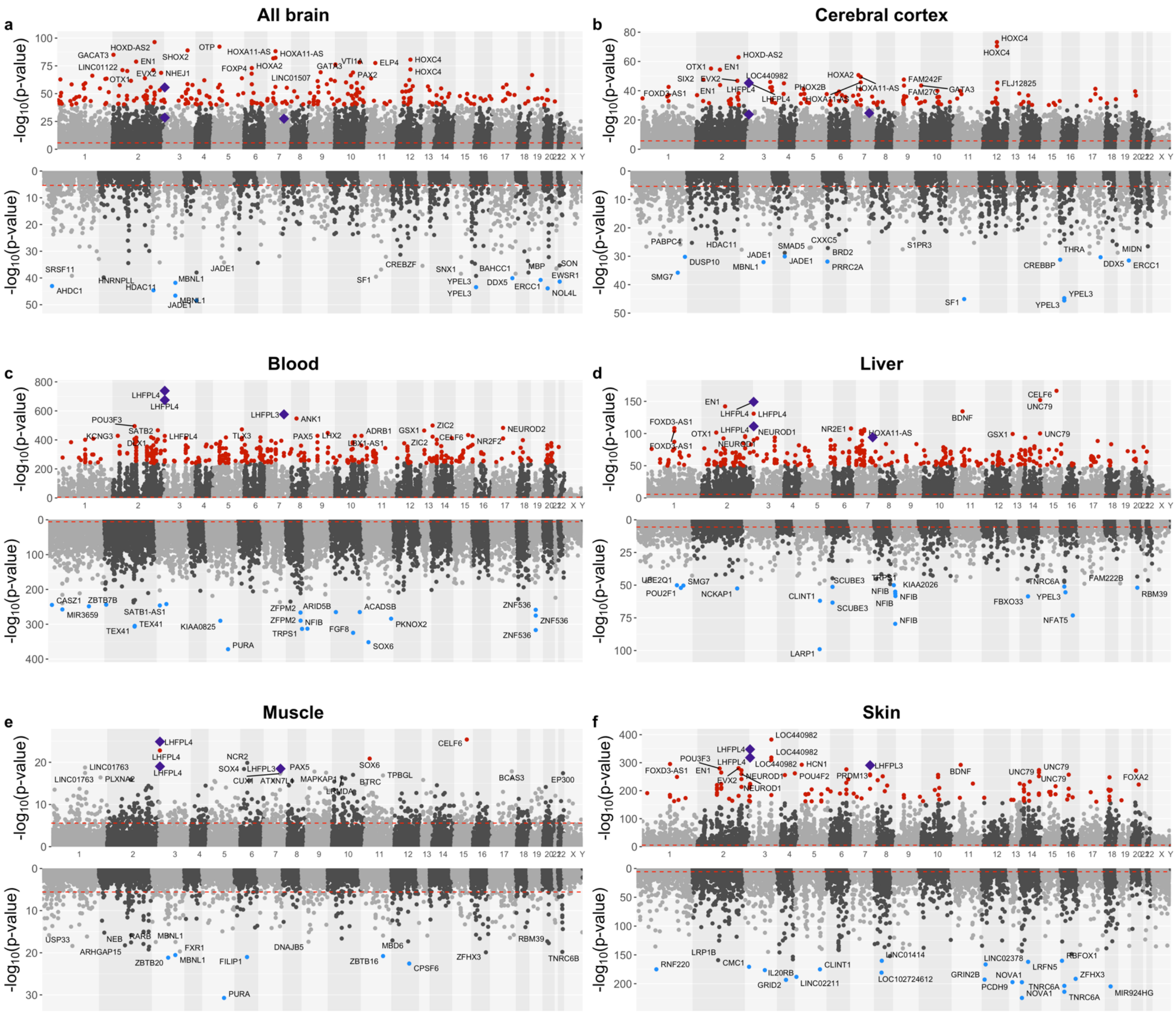
a
All brain
b
Cerebral cortex
c
Blood
d
Liver
e
Muscle
f
Skin
-log10(p-value)
HOXD-AS2
GACAT3
LINC01122
EN1
OTX1
EVX2
SHOX2
NHEJ1
OTP
HOXA11-AS
FOXP4
HOXA2
LINC01507
GATA3
VTI1A
PAX2
ELP4
HOXC4
SRSF11
AHDC1
HNRNPLL
HDAC11
MBNL1
JADE1
CREBZF
SF1
SNX1
YPEL3
BAHCC1
DDX5
ERCC1
MBP
SON
EWSR1
NOL4L
SIX2
FOXD3-AS1
LOC440982
LHFPL4
PHOX2B
FAM242F
FAM27C
FLJ12825
PABPC4
DUSP10
SMG7
HDAC11
SMAD5
CXXC5
BRD2
PRRC2A
S1PR3
CREBBP
THRA
MIDN
LHFPL3
POU3F3
KCNG3
SATB2
DLX1
TLX3
ANK1
PAX5
LHX2
LBX1-AS1
ADRB1
GSX1
ZIC2
CELF6
NR2F2
NEUROD2
CASZ1
ZBTB7B
MIR3659
SATB1-AS1
TEX41
KIAA0825
PURA
ZFPM2
ARID5B
NFIB
TRPS1
FGF8
ACADSB
SOX6
PKNOX2
ZNF536
NEUROD1
NR2E1
BDNF
UNC79
UBE2Q1
POU2F1
NCKAP1
CLINT1
SCUBE3
KIAA2026
LARP1
TNRC6A
FBXO33
NFAT5
FAM222B
RBM39
LINC01763
PLXNA2
NCR2
SOX4
CUX1
ATXN7L1
MAPKAP1
LRMDA
BTRC
TPBGL
BCAS3
EP300
USP33
ARHGAP15
NEB
RARB
ZBTB20
FXR1
FILIP1
DNAJB5
ZBTB16
MBD6
CPSF6
ZFHX3
RBM39
TNRC6B
HCN1
POU4F2
PRDM13
FOXA2
RNF220
LRP1B
CMC1
GRID2
IL20RB
LINC02211
LINC01414
LOC102724612
GRIN2B
LINC02378
PCDH9
NOVA1
LRFN5
RBFOX1
MIR924HG

Extended Data Figure 6. Meta-analysis of chronological age in mammalian samples across specific tissue types. Meta-analysis p-value (-log base 10 transformed) versus chromosomal location (x-axis) according to human genome assembly 38 (hg38) in **a**), brain tissues (across multiple brain regions), **b**) cerebral cortex, **c**) blood, **d**) liver, **e**) muscle and **f**) skin tissues. The upper and lower panels of the Manhattan plot depict the CpG sites that gain/lose methylation with age. In panel a, P values were calculated via two-stage meta-analysis that combined EWAS results across strata formed by species/brain-tissue (with n≥15 samples, Methods). CpGs are colored in red and blue if they exhibit highly significant positive and negative age correlations according to a meta analysis $P < 1.0 \times 10^{-40}$, 1.0×10^{-30}, 1.0×10^{-250}, 1.0×10^{-50}, 1.0×10^{-20} and 1.0×10^{-150} for a–f, respectively. Red dashed horizontal lines denote Bonferroni correction. Gene names are annotated for the top 20 CpGs with positive and negative associations, respectively. CpGs are labeled by adjacent genes. Purple color and diamond shapes mark CpGs of particular interest: cg12841266 and cg11084334 in *LHFPL4* and cg09710440 in *LHFPL3.* All P-values presented in this figure are unadjusted and computed using two-sided tests.

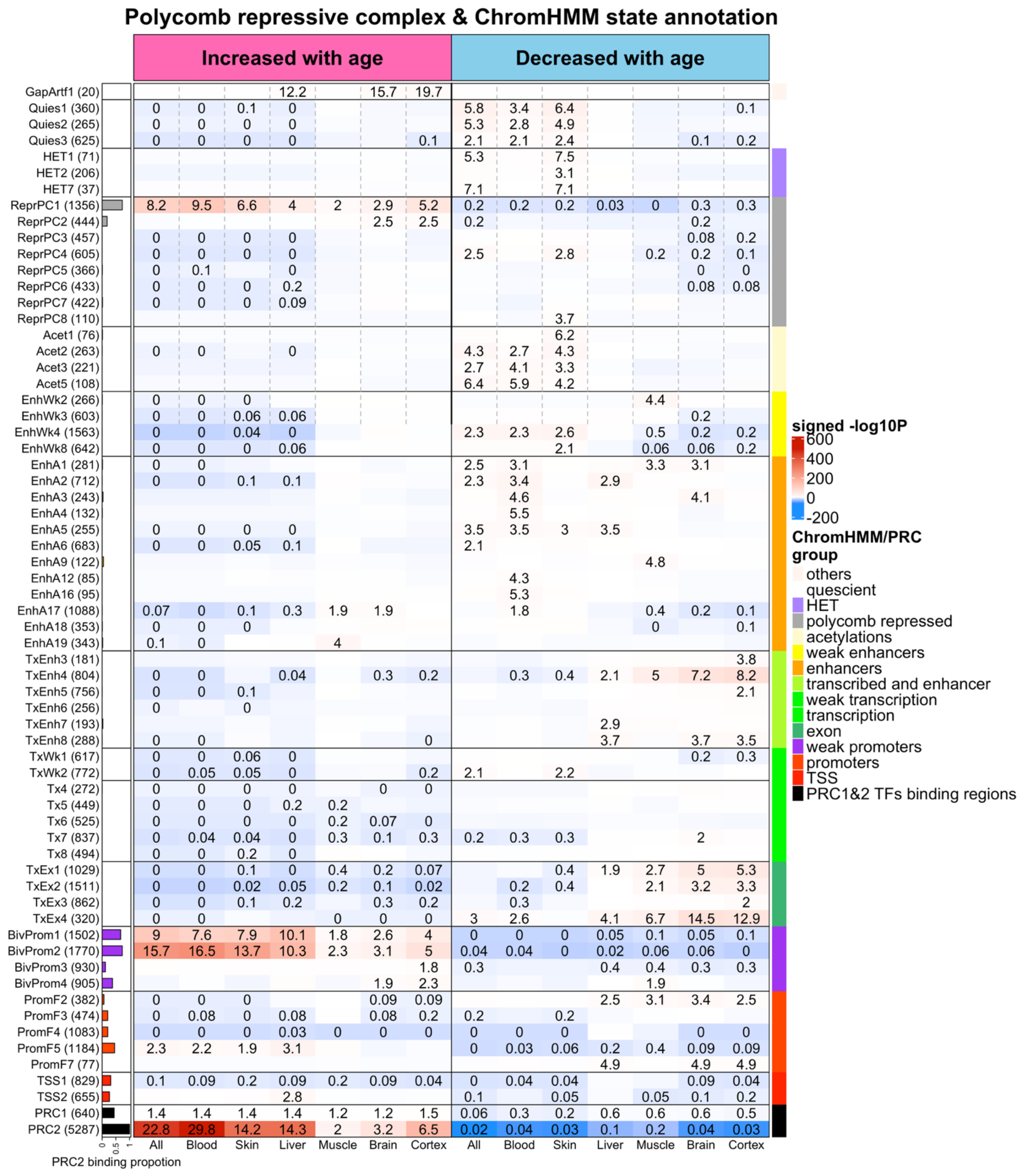
Polycomb repressive complex & ChromHMM state annotation
Increased with age
Decreased with age
GapArtf1 (20)
Quies1 (360)
Quies2 (265)
Quies3 (625)
HET1 (71)
HET2 (206)
HET7 (37)
ReprPC1 (1356)
ReprPC2 (444)
ReprPC3 (457)
ReprPC4 (605)
ReprPC5 (366)
ReprPC6 (433)
ReprPC7 (422)
ReprPC8 (110)
Acet1 (76)
Acet2 (263)
Acet3 (221)
Acet5 (108)
EnhWk2 (266)
EnhWk3 (603)
EnhWk4 (1563)
EnhWk8 (642)
EnhA1 (281)
EnhA2 (712)
EnhA3 (243)
EnhA4 (132)
EnhA5 (255)
EnhA6 (683)
EnhA9 (122)
EnhA12 (85)
EnhA16 (95)
EnhA17 (1088)
EnhA18 (353)
EnhA19 (343)
TxEnh3 (181)
TxEnh4 (804)
TxEnh5 (756)
TxEnh6 (256)
TxEnh7 (193)
TxEnh8 (288)
TxWk1 (617)
TxWk2 (772)
Tx4 (272)
Tx5 (449)
Tx6 (525)
Tx7 (837)
Tx8 (494)
TxEx1 (1029)
TxEx2 (1511)
TxEx3 (862)
TxEx4 (320)
BivProm1 (1502)
BivProm2 (1770)
BivProm3 (930)
BivProm4 (905)
PromF2 (382)
PromF3 (474)
PromF4 (1083)
PromF5 (1184)
PromF7 (77)
TSS1 (829)
TSS2 (655)
PRC1 (640)
PRC2 (5287)
0 0.5 1
PRC2 binding propotion
All Blood Skin Liver Muscle Brain Cortex All Blood Skin Liver Muscle Brain Cortex
signed -log10P
600
400
200
0
-200
ChromHMM/PRC group
others
quescient
HET
polycomb repressed
acetylations
weak enhancers
enhancers
transcribed and enhancer
weak transcription
transcription
exon
weak promoters
promoters
TSS
PRC1&2 TFs binding regions

Extended Data Figure 7. Chromatin state analysis of age-related CpGs. The heatmap color-codes the hypergeometric overlap analysis between age-related CpGs (columns) and two groupings of CpGs (a) universal chromatin states analysis [1]and (b) binding by polycomb repressive complex 1 and 2 (PRC1, PRC2) defined based on ChipSeq datasets in ENCODE [2] , see the last two rows. The first column shows a bar plot that reports the proportion of CpGs that are known to be bounded by PRC2 that ranges from zero to one (PRC2). Note that chromatin states that contain a high proportion of PRC2 bound CpGs overlap significantly with the top 1,000 CpGs that increased with age across tissues and mammal species. For each row (chromatin state or PRC annotation), the table reports odds ratios (OR) from hypergeometric test results for the top 1,000 CpGs that increased/decreased with age from meta-EWAS of age across all, blood, skin, liver, muscle, brain and cerebral cortex tissues, respectively. Unadjusted hypergeometric P values based on one-sided are listed in **Supplementary Data 5.3—5.9**. The heatmap color gradient is based on -log10 (unadjusted hypergeometric P value) multiplied by the sign of OR greater than one. Red colors denote OR greater than one in contrast with blue colors for OR less than one. Legend lists states based on their group category and PRC group. The y-axis lists state or PRC name and number of mammalian array CpGs inside parentheses. The left/right panel lists the results based on the top 1,000 CpGs with positive/negative age correlation. We displayed 63 universal chromatin states that show significant enrichment/depletion at $P < 0.001$ in any of the tissues.

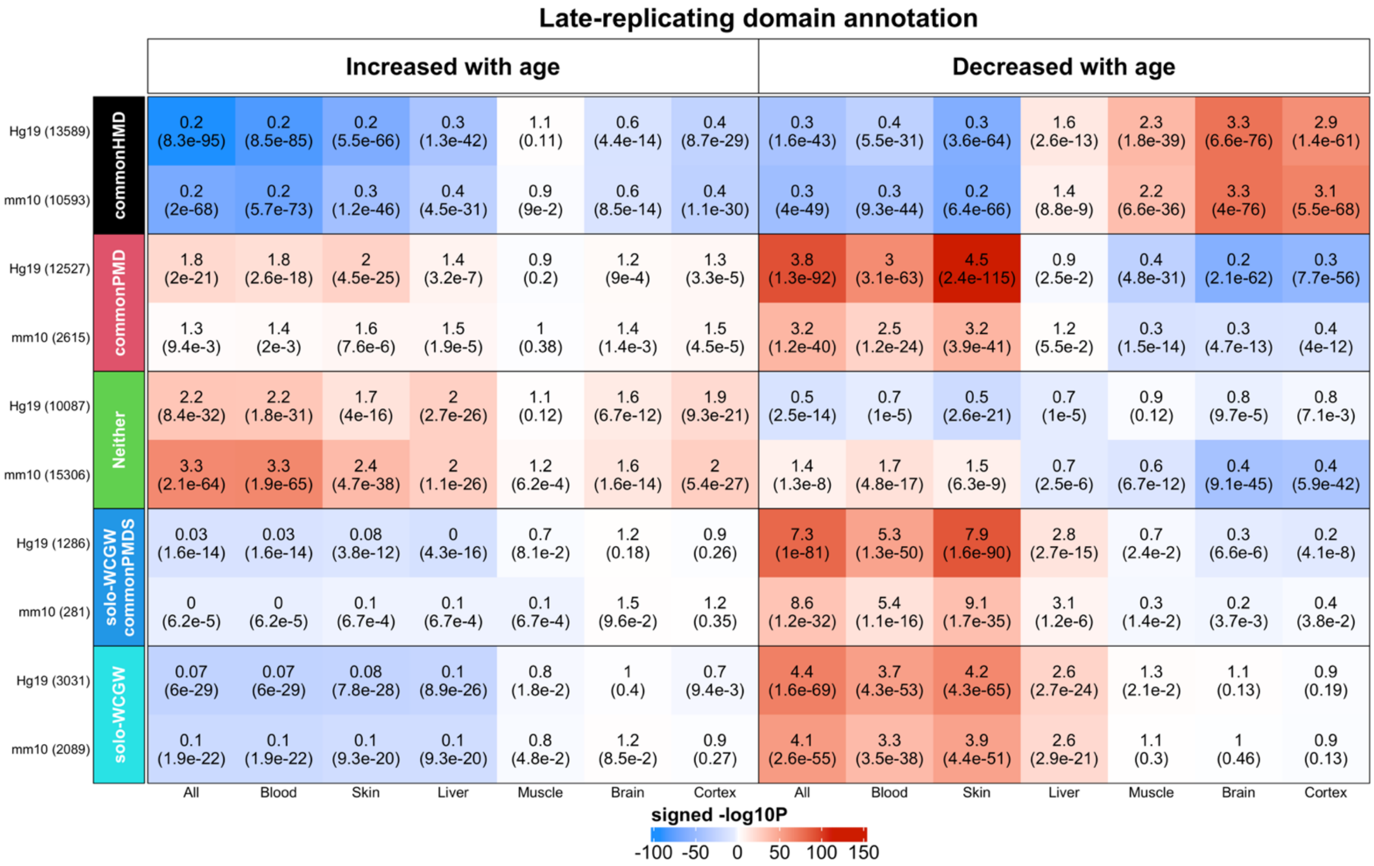

Extended Data Figure 8. Overlap with late-replicating domains. The heatmap color-codes the hypergeometric overlap analysis between age-related CpGs (columns) and CpGs related to late-replicating domains in Hg19 and mm10 assembly[3], respectively. Two groups of late-replicating domains were analyzed (**a**) common PMD/HMD structures: highly methylated domains (commonHMD), partially methylated domains (commonPMD), and neither (Neither), and (**b**) solo-WCGW structures: genome-wide (solo-WCGW) and those in the common PMD regions (solo-WCGW commonPMDs). The y-axis lists categories of late-replicating domains and number of mammalian array CpGs inside parentheses for Hg19 and mm10 genome, respectively. For each row, the table reports odds ratios (OR) from hypergeometric test results for the top 1,000 CpGs that increased/decreased with age from meta-EWAS of age across all, blood, skin, liver, muscle, brain, and cerebral cortex tissues, respectively. The heatmap color gradient is based on -log10 (unadjusted hypergeometric P value) multiplied by the sign of OR greater than one. Red colors denote OR greater than one in contrast with blue colors for OR less than one. The left/right panel lists the results based on the top 1,000 CpGs with positive/negative age correlation. Unadjusted P values are reported and derived from one-sided hypergeometric tests.

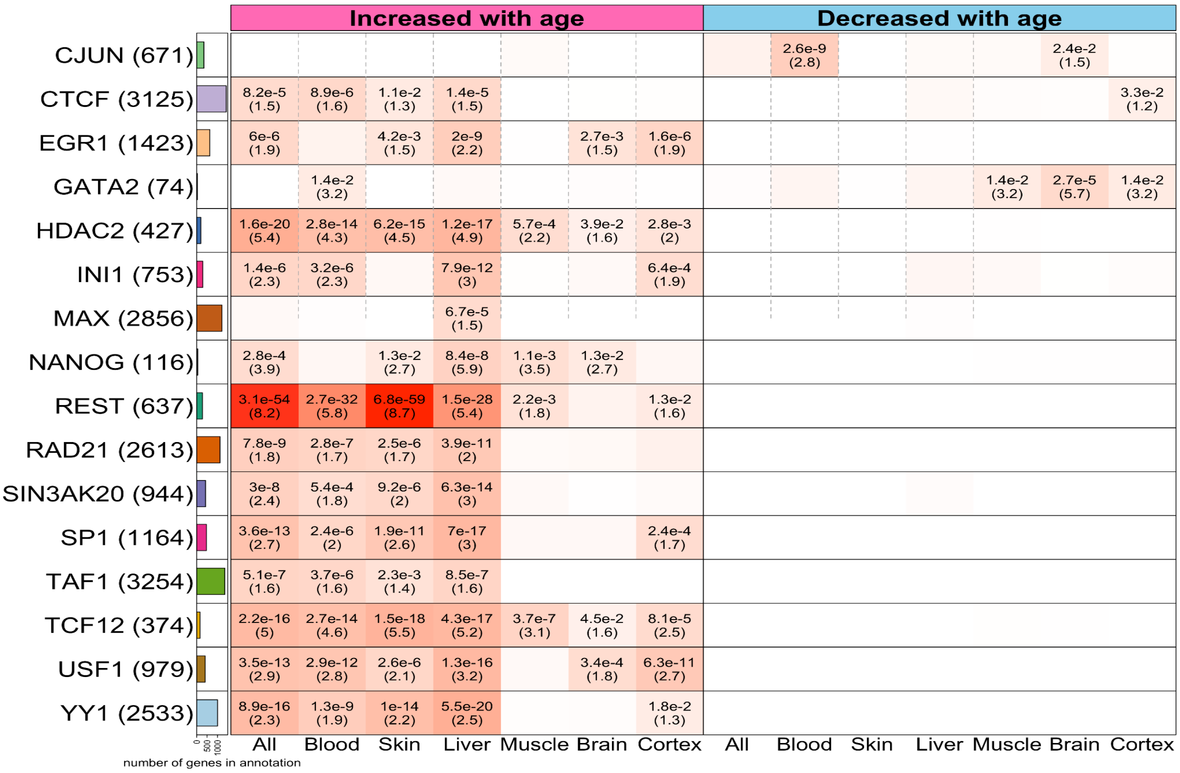

Extended Data Figure 9. Enrichment with Transcription factor binding regions. We studied the overlapping genomic regions between (1) the CpG sites located in the binding regions of 68 transcription factors (TF) in hg19 and (2) the top 1000 CpGs that increased/decreased with age from EWAS of age across mammalian tissues. TF results (y-axis, rows) versus mammalian EWAS of age are stratified by tissue type (x-axis, columns). The left/right panels of the x-axis list the top 1000 CpGs that increased/decreased with age from meta-EWAS of age across all tissues, blood only, skin only, liver, muscle, brain and cerebral cortex, respectively. The y-axis lists the names of transcription factors and number of mammalian array CpGs located in the binding sites. Background in hypergeometric tests was based on the genes present in our mammalian array. The bar plots in the first column report the total number of genes at each TF according to the background. The heatmap color codes -log10 (unadjusted hypergeometric P value). Unadjusted, one-sided hypergeometric P values (odds ratio) are listed on the heatmap provided $P<0.05$.

(a)

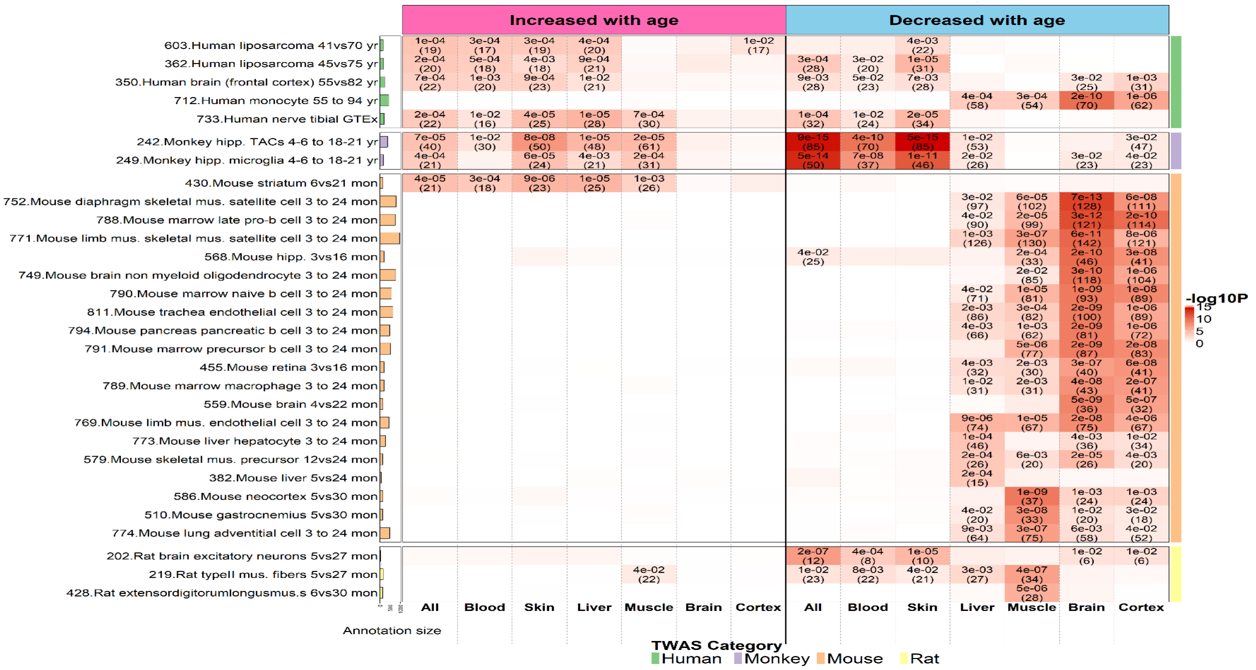

Increased with age
Decreased with age
603.Human liposarcoma 41vs70 yr
362.Human liposarcoma 45vs75 yr
350.Human brain (frontal cortex) 55vs82 yr
712.Human monocyte 55 to 94 yr
733.Human nerve tibial GTEx
242.Monkey hipp. TACs 4-6 to 18-21 yr
249.Monkey hipp. microglia 4-6 to 18-21 yr
430.Mouse striatum 6vs21 mon
752.Mouse diaphragm skeletal mus. satellite cell 3 to 24 mon
788.Mouse marrow late pro-b cell 3 to 24 mon
771.Mouse limb mus. skeletal mus. satellite cell 3 to 24 mon
568.Mouse hipp. 3vs16 mon
749.Mouse brain non myeloid oligodendrocyte 3 to 24 mon
790.Mouse marrow naive b cell 3 to 24 mon
811.Mouse trachea endothelial cell 3 to 24 mon
794.Mouse pancreas pancreatic b cell 3 to 24 mon
791.Mouse marrow precursor b cell 3 to 24 mon
455.Mouse retina 3vs16 mon
789.Mouse marrow macrophage 3 to 24 mon
559.Mouse brain 4vs22 mon
769.Mouse limb mus. endothelial cell 3 to 24 mon
773.Mouse liver hepatocyte 3 to 24 mon
579.Mouse skeletal mus. precursor 12vs24 mon
382.Mouse liver 5vs24 mon
586.Mouse neocortex 5vs30 mon
510.Mouse gastrocnemius 5vs30 mon
774.Mouse lung adventitial cell 3 to 24 mon
202.Rat brain excitatory neurons 5vs27 mon
219.Rat typeII mus. fibers 5vs27 mon
428.Rat extensordigitorumlongusmus.s 6vs30 mon
All Blood Skin Liver Muscle Brain Cortex All Blood Skin Liver Muscle Brain Cortex
Annotation size
-log10P
15
10
5
0
TWAS Category
Human Monkey Mouse Rat

(b)

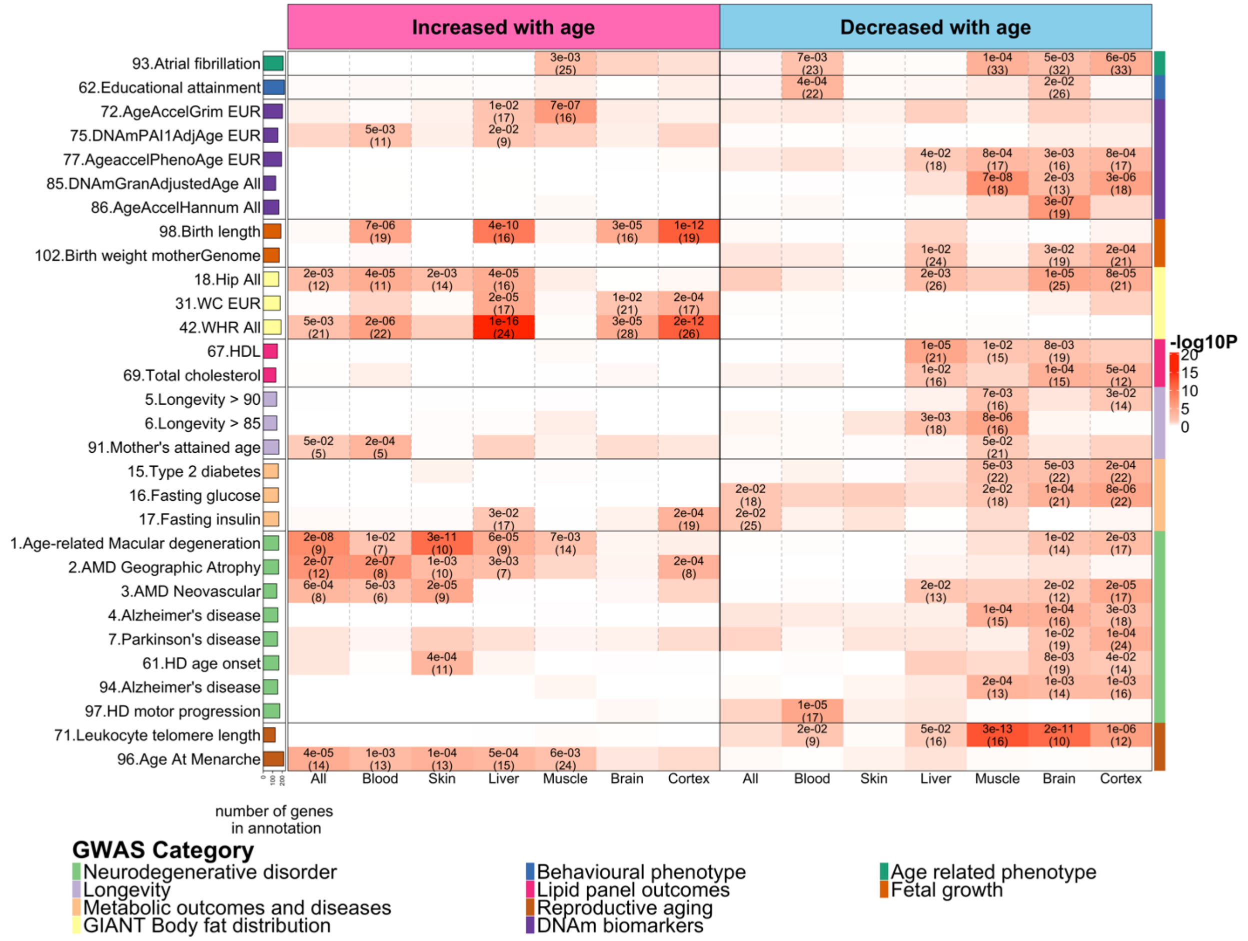

Increased with age
Decreased with age
93.Atrial fibrillation
62.Educational attainment
72.AgeAccelGrim EUR
75.DNAmPAI1AdjAge EUR
77.AgeaccelPhenoAge EUR
85.DNAmGranAdjustedAge All
86.AgeAccelHannum All
98.Birth length
102.Birth weight motherGenome
18.Hip All
31.WC EUR
42.WHR All
67.HDL
69.Total cholesterol
5.Longevity > 90
6.Longevity > 85
91.Mother's attained age
15.Type 2 diabetes
16.Fasting glucose
17.Fasting insulin
1.Age-related Macular degeneration
2.AMD Geographic Atrophy
3.AMD Neovascular
4.Alzheimer's disease
7.Parkinson's disease
61.HD age onset
94.Alzheimer's disease
97.HD motor progression
71.Leukocyte telomere length
96.Age At Menarche
All Blood Skin Liver Muscle Brain Cortex All Blood Skin Liver Muscle Brain Cortex
number of genes in annotation
-log10P
20
15
10
5
0
GWAS Category
Neurodegenerative disorder
Longevity
Metabolic outcomes and diseases
GIANT Body fat distribution
Behavioural phenotype
Lipid panel outcomes
Reproductive aging
DNAm biomarkers
Age related phenotype
Fetal growth

Extended Data Figure 10. EWAS-TWAS and EWAS-GWAS enrichment. Panel (**a**) illustrates the overlap between genes identified in transcriptome-wide association studies (TWAS) across various cell types or species, and the top 1,000 CpGs that have increased/decreased with age in EWAS across mammalian tissues. TWAS results are stratified by tissue type, including all tissues, blood, skin, liver, muscle, brain, and cerebral cortex. Overlapping genes with $P<0.05$ are reported. Similarly, Panel (**b**) demonstrates the overlaps between the top 2.5% genes implicated in genome-wide association studies (GWAS) of human complex traits, and the top 1,000 CpGs that have increased/decreased with age in EWAS across mammalian tissues. GWAS results are also stratified by tissue type, with significant overlaps reported where $P<0.05$. Both panels utilize unadjusted, one-sided hypergeometric P values, with a background for hypergeometric tests derived from genes (panel a) or genomic regions (panel b) in our mammalian array. The heatmap color encodes -log10 P values. The right-side annotation indicates (a) the species categories for TWAS collections and (b) phenotype categories for GWAS collections. Further details for TWAS and GWAS indices are available in **Supplementary Data 12&13**. Abbreviations: (a) hipp.=hippocampus, MPNST= malignant peripheral nerve sheath tumor, mus.=muscle, TACs= transiently amplifying progenitor cells. (b) All=All ancestries, EUR=European ancestry, AFR=African American ancestry, FTD= frontotemporal dementia, WHR= waist to hip ratio.

Supplementary Tables

Universal Clock	Validation scheme	DNAm Age vs age				DNAm relative age vs relative age		
		Cor	MAE	med. Cor	med. MAE	Cor	MAE	med. MAE
**Clock 1:** Basic, log(Age+2)	LOFO	0.963	0.757	0.880	0.962	0.884	0.034	0.033
**Clock 2:** RelativeAge	LOFO	0.983	0.562	0.925	0.676	0.950	0.024	0.026
**Clock 3:** log-linear age	LOFO	0.980	0.550	0.918	0.743	0.942	0.024	0.029
**Clock 1:** Basic, log(Age+2)	LOSO	0.828	1.855	0.862	1.725	0.455	0.080	0.059
**Clock 2:** RelativeAge	LOSO	0.941	1.439	0.881	1.428	0.786	0.060	0.049
**Clock 3:** log-linear age	LOSO	0.915	1.416	0.880	1.661	0.677	0.059	0.056

Supplementary Table 1. Predictive accuracy of three universal mammalian clocks.

The rows correspond to three universal mammalian clocks for 1) log-transformed age, log(Age+2), 2) relative age, and 3) log-linear age. The inverse age transformations were used to arrive at age estimates, DNA methylation age (in units of years). Each clock was evaluated with two validation schemes: the first three rows report results for leave-one-fraction-out cross validation (LOFO). The last three rows report results for leave-one-species-out cross validation (LOSO). Columns 3 and 4 report the Pearson correlation coefficient (Cor) and median absolute error (MAE, in units of years) across all samples, i.e. species and tissue type are being ignored. Columns 5 and 6 report the median value across species, i.e. median Cor (med.Cor) and median MAE (med.MAE), for species with at least 15 observations. Columns 7–9 repeated the analysis based on relative age. Since the med.Cor for relative age is the same as med Cor for age, we omitted the former. In Clocks 1 and 3, DNAm relative age was computed based on formula (1) with the variable Age replaced by DNAm Age.

Variable	Number of data	n	Bicor		P-value	
			Clock2	Clock3	Clock2	Clock3
**Diet**						
log2(Total energy)	1	2085	-0.003	-0.003	9.0E-01	8.7E-01
Carbohydrate	1	2085	0.005	0.008	8.2E-01	7.0E-01
Protein	1	2085	-0.001	0.010	9.5E-01	6.5E-01
Fat	1	2085	-0.003	-0.016	9.1E-01	4.5E-01
log2(1+Red meat)	2	4423	0.018	0.015	2.3E-01	3.3E-01
log2(1+Poultry)	2	4423	-0.017	-0.006	2.6E-01	7.1E-01
log2(1+Fish)	2	4423	-0.020	-0.005	1.8E-01	7.2E-01
log2(1+Dairy)	2	4405	-0.003	0.004	8.5E-01	7.7E-01
log2(1+Whole grains)	2	4410	-0.002	0.013	9.1E-01	4.0E-01
log2(1+Nuts)	1	2085	-0.013	-0.015	5.7E-01	5.0E-01
log2(Fruits)	2	4397	-0.016	-0.002	2.8E-01	9.1E-01
log2(Vegetables)	2	4407	0.008	0.012	5.9E-01	4.2E-01
log(OMEGA3)	1	2313	-0.005	0.008	8.2E-01	7.0E-01
log(VitaminA)	1	2337	0.015	0.018	4.7E-01	3.8E-01
log(VitaminC)	1	2336	0.023	0.024	2.6E-01	2.5E-01
log(VitaminB6)	1	2329	-0.033	-0.022	1.1E-01	2.9E-01
log(VitaminE)	1	2305	0.029	0.031	1.6E-01	1.4E-01
log(Selenium)	1	2300	-0.032	-0.028	1.3E-01	1.7E-01
log(Iron)	1	2299	-0.006	0.011	7.7E-01	6.1E-01
**log(Zinc)**	1	2321	-0.036	-0.020	**8.0E-02**	3.3E-01
log(Calcium)	1	2323	-0.003	-0.006	9.0E-01	7.8E-01
log(FolicAcid)	1	2305	-0.009	0.004	6.5E-01	8.6E-01
log(VitaminD)	1	2308	0.006	0.001	7.7E-01	9.5E-01
log(Copper)	1	2317	0.004	0.023	8.4E-01	2.7E-01
log(BrewYeast)	1	2305	0.016	0.022	4.6E-01	2.9E-01
log(BetaCaroteneSup)	1	2309	-0.003	-0.013	8.9E-01	5.4E-01
log(Magnesium)	1	2317	-0.019	-0.029	3.7E-01	1.6E-01
**Diet Biomarkers**						
Retinol	1	223	0.042	0.070	5.4E-01	3.0E-01
**Mean carotenoids**	1	223	-0.158	-0.172	**1.9E-02**	**1.0E-02**
Lycopene	1	223	-0.092	-0.084	1.7E-01	2.2E-01
**log2(alpha-Carotene)**	1	223	-0.121	-0.109	**7.2E-02**	1.1E-01
**log2(beta-Carotene)**	1	223	-0.134	-0.114	**4.6E-02**	**9.0E-02**
**log2(Lutein+Zeaxanthin)**	1	223	-0.107	-0.114	1.1E-01	**9.3E-02**

log2(beta-Cryptoxanthin)	1	223	-0.081	-0.103	2.3E-01	1.3E-01
log2(alpha-Tocopherol)	1	223	-0.012	0.002	8.6E-01	9.7E-01
log2(gamma-Tocopherol)	1	223	0.041	0.029	5.5E-01	6.6E-01

			**Measurements**			
log(A1C)	1	2541	-0.021	-0.041	2.8E-01	**3.8E-02**
**log2(C-reactive protein)**	2	4587	0.118	0.107	**9.9E-16**	**3.8E-13**
**log2(Insulin)**	1	2048	0.038	0.019	**8.8E-02**	4.0E-01
**log2(Glucose)**	2	4632	0.038	0.022	**9.3E-03**	1.3E-01
**log2(Triglyceride)**	2	4633	0.075	0.074	**3.2E-07**	**4.4E-07**
Total cholesterol	2	4633	-0.021	0.010	1.5E-01	4.9E-01
LDL cholesterol	1	2058	0.005	0.015	8.3E-01	4.9E-01
**HDL cholesterol**	2	4632	-0.047	-0.031	**1.4E-03**	**3.6E-02**
log2(Creatinine)	2	4555	0.011	0.011	4.6E-01	4.7E-01
log2(Urine Creatinine)	1	2500	-0.002	0.004	9.1E-01	8.5E-01
**FEV1**	1	2072	-0.050	-0.023	**2.3E-02**	3.0E-01
**Systolic blood pressure**	2	4651	0.040	0.021	**6.2E-03**	1.5E-01
Diastolic blood pressure	2	4650	0.020	0.022	1.7E-01	1.3E-01
**log2(Waist / hip ratio)**	2	4606	0.044	0.034	**2.6E-03**	**2.2E-02**
**BMI**	2	4627	0.038	0.022	**1.1E-02**	1.4E-01
MMSE	2	3371	-0.006	-0.007	7.5E-01	6.9E-01
Telomere length	2	1694	-0.011	-0.003	6.4E-01	9.2E-01
			**Life style**			
Education	2	4218	-0.020	-0.004	1.9E-01	7.7E-01
Income	1	2033	-0.021	0.015	3.4E-01	5.0E-01
Hand grip	1	1986	-0.009	0.012	6.9E-01	5.8E-01
**log2(1+Exercise)**	1	1996	-0.061	-0.053	**6.4E-03**	**1.8E-02**
**Current smoker**	2	4618	0.033	0.038	**2.7E-02**	**9.1E-03**
log2(1+Alcohol)	2	4626	0.005	0.013	7.5E-01	3.6E-01

Supplementary Table 2. Meta cross-sectional correlations with diet, clinical biomarkers and lifestyle factors. Robust correlation coefficients (biweight midcorrelation [bicor] [4]) between epigenetic age acceleration (AgeAccel) measures of universal pan mammalian Clock2 and Clock 3 , and 2) 59 variables including 27 self-reported diet, 9 dietary biomarkers, 17 clinically relevant measurements related to vital signs, metabolic traits, inflammatory markers, cognitive function, lung function, central adiposity and leukocyte telomere length, and 6 lifestyle factors including hand grip strength. We applied bicor analysis on the individuals from Framingham Heart Study (FHS, n up to 2544) and Women's Health Initiative (WHI, n up to 2107), stratified by gender and ethic category respectively. The columns list variables, number of datasets, number of individuals, meta bicor estimate based on AgeAccel from Clock2 and Clock3, and unadjusted meta P values from Clock2 and Clock3, respectively. The variables are marked in bold provided P<0.10 from any clock estimate. The P values are marked in red bold fonts provided P<0.05 and in blue bold fonts provided 0.05≤ P <0.1. All P values are unadjusted for multiple testing and are computed based on two-sided tests.

Rank	Chr	Gene	CpG	Protein domain	Activity	Function
**Increased with age**						
1	3	LHFPL4	cg12841266	Tetraspan transmembrane	Clustering of GABA receptors	Dev. of inhibitory synapse
2	3	ZIC1	cg08938156	Zinc finger	TF	Early Dev.
3	7	LHFPL3	cg09710440	Tetraspan transmembrane	Clustering of GABA receptors	Dev. of inhibitory synapse
4	5	TLX3	cg26844246	Homeobox	TF	Dev.
5	2	EVX2	cg09227056	Homeobox	TF	Limb Dev.
6	13	ZIC2	cg15682828	Zinc finger	transcriptional activator or repressor	important in the early stage of organogenesis of the CNS
7	1	FOXD3	cg05551621	Forkhead	TF	Embryonic Dev.
8	15	CELF6	cg23087015	RNA recognition motif (RRM) domains	RNA-binding	RNA processing
9	17	NEUROD2	cg03679521	bHLH	TF	Neural Dev.
10	11	BDNF	cg27201382	neurotrophin family of growth factors	--	Brain Dev.
11	10	PAX2	cg01486146	Paired box	TF	development of the urogenital tract, the eyes, and the CNS
12	9	PAX5	cg20766695	Paired box	TF	Early Dev.
13	5	OTP	cg24352905	Homeobox	TF	Dev. / Cell fate specification
14	16	SALL1	cg13909487	Zinc finger	TF	Stem cell regulation and Dev.
15	6	NRN1	cg16356803	neuritin family	--	Promotes neurite outgrowth
16	6	PRDM13	cg05797975	PR Domain Zinc Finger	chromatin binding *and* methyltransferase activity	transcriptional regulation
17	6	NR2E1	cg27496468	Zinc finger	Nuclear hormone receptor	Retinal Dev.
18	2	NEUROD1	cg09942248	bHLH	TF	Neural Dev.
19	6	TBX18	cg09817427	T-Box	TF	embryonic development of the sino atrial node head area
20	20	NKX2-2	cg16524928	Homeobox	TF	Spinal cord Dev.
21	6	TFAP2D	cg26834803	--	TF	Embryonic Dev.
22	2	SIX2	cg03557129	Homeobox	TF	Limb & Eye Dev.
23	7	TWIST1	cg20477718	bHLH	TF	Embryonic Dev.
24	4	PHOX2B	cg13278722	Homeobox	TF	Neural Dev.
25	13	OBI1-AS1	cg13739934	--	Anti-sense RNA	--

26	11	DBX1	cg21452781	Homeobox	TF	establishing the distinction of V0 and V1 neuronal fate
27	13	ZIC5	cg24255409	Zinc finger	TF	Early Dev.
28	2	OTX1	cg03957108	Homeobox	TF	Brain and sensory Dev.
29	5	LOC642366	cg16744551	--	Anti-sense RNA	--
30	14	VSX2	cg10707128	Homeobox	retina-specific TF	specification and morphogenesis of the sensory retina
31	7	HOXA13	cg26512254	Homeobox	TF	embryonic development
32	7	DLX6-AS1	cg02898094	--	Anti-sense RNA	Dev.
33	8	EGR3	cg02040024	Zinc finger	Transcription Regulator	Numerous Dev. processes
34	6	POU3F2	cg08921975	Homeobox	TF	Nueron Dev.
35	5	IRX1	cg02926165	Homeobox	TF	Pattern formation
**decreased with age**						
1	5	LARP1	cg12880090	regulating translation of mTORC1 downstream targets	RNA-binding	Regulation of translation
2	21	SON	cg24466972	--	RNA-binding	RNA splicing
3	15	SNX1	cg27626343	Phox (PX)	Endosomal protein	Regulates expression of EGF receptor

Supplementary Table 3. Top age-related CpGs consistently found by EWAS in different tissues of mammals. The table provides details on CpGs and genes that are in the intersection (Venn diagram) of EWAS of age in different tissues across different mammalian species (see **Fig. 1g**). For brain, blood, liver, muscle and skin we identified the top 1,000 *positively* age-related CpGs for each tissue type. The five sets of CpGs shared 48 CpGs located near 35 genes (increased with age). We only report the most significant CpG when several CpGs implicate the same gene. Conversely, the top 1,000 *negatively* age-related CpGs in the five tissue types implicated only three genes (decreased with age). We arrange the list of CpGs based on the EWAS meta P-value from all tissues, stratified by the sign or EWAS meta Z score. Columns report the rank, chromosome of the gene (CHR), HGNC gene symbol, the most significant CpG, protein domain, activity and function of gene expression. Abbreviations: Dev=development; TF=transcriptional factor, CNS= central nervous system.

Supplementary Note

Supplementary Note 1: Data from the Mammalian Methylation Consortium

The combined data was generated by the Mammalian Methylation Consortium. DNA was extracted using the DNeasy Blood & Tissue Kit from Qiagen. Nucleic acid purity was inspected with a NanoDrop spectrophotometer, and quantified using a Qubit fluorometer dsDNA BR Assay.

Below, we list the descriptions of species.

M1.Primates [5]

Ethics

This research complied with all relevant ethical regulations overseen by seven ethics review boards. The human skin samples were acquired with informed consent prior to collection of human skin samples with approved by the Oxford Research Ethics Committee in the UK; reference 10/H0605/1. Participants were not compensated. The secondary use of the other de-identified/coded human tissue samples (blood, postmortem tissues) is not interpreted as human subjects research under U.S. Department of Health & Human Services 45 CFR 46. Therefore, the need to obtain written, informed consent from human study participants was waived (secondary use of de-identified tissues). Human samples were covered by University of California Los Angeles IRB#18-000315. All procedures related to non-human primates were approved by different committees: baboons (UTHSCSA Animal Care and Use Committee), strepsirrhini (Duke Institutional Animal Care and Use Committee and the DLC Research Committee), rhesus macaques (Animal Care and Use Committee of the NIA Intramural Program) [6], vervet monkey (UCLA and VA Institutional Animal Care and Use Committees) [7], marmosets (IACUC of UTHSA) [8].

Baboon care and maintenance

All animals were given a full veterinary examination prior to recruitment to the study and no obvious cause of ill health or pathology was observed. The animals were housed in group cages at the Southwest National Primate Research Center, at Texas Biomedical Research Institute (TBRI), in San Antonio, Texas in mixed sex groups of up to 16. The remaining 4 females were housed in individual cages at the UT Health Sciences Center San Antonio (UTHSCSA).

Twenty-eight females and the ten males were fed ad libitum Purina Monkey Diet 5038 (12% energy from fat, 0.29% from glucose and 0.32% from fructose and metabolizable energy content of 3.07 kcal/g protein; Purina LabDiets, St Louis, MO, USA) (CTR). Water was continuously available to all animals. Animal health status was recorded daily.

Necropsy

None of the animals were euthanized for this project. Rather, we used left-over frozen tissue samples that had previously been collected as part of other projects. Necropsies were performed by either a qualified, experienced veterinarian or M.D investigator. At TRBI, baboons were pre-medicated with ketamine hydrochloride (10 mg/kg IM) and anesthetized using isoflurane (2%) resulting in general anesthesia as previously described [9]. Baboons were exsanguinated while under general anesthesia as approved by the American Veterinary Medical Association. At UTHSCSA four animals were euthanized using Pentobarbital at 390 mg/ml (Fatal- Plus Solution, Vortech, Dearborn, MI, USA) . Following cardiac asystole, respiratory failure and a lack of reflexive response to both skin pinch and eye touch stimulation, tissues (adipose, cerebellum, cerebral

cortex, muscle, heart, liver) were rapidly dissected and immediately frozen in liquid nitrogen.

For the studies in which fetal tissue was obtained, all animals were housed in 20 foot × 20 foot × 15 foot metal and concrete group cages at the Texas Biomedical Research Institute. Experimental animals were obtained from appropriate groups of 16 healthy female baboons of similar pre-study body weights (10–15 kg) and morphometric features. The potential day of conception was determined based on the day of ovulation and changes in sex skin color and pregnancy was confirmed at 30 days post ovulation by using ultrasonography. Details of housing, feeding, and environmental enrichment have been published elsewhere. All procedures were approved by the University of Texas Health Science Center and Texas Biomedical Research Institute internal animal care and use committees and performed in the Association for Assessment and Accreditation of Laboratory Animal Care–approved facilities.

Prior to Cesarean section, baboons were premedicated with ketamine hydrochloride (10 mg/kg, IM). Following tracheal intubation, isoflurane (2%, 2L/min, by inhalation) was used to maintain an appropriate plane of anesthesia throughout the surgery. A cesarean section was performed at gestational day 165 (0.9 of gestation) using standard sterile techniques as previously described [10]. Following hysterotomy, the umbilical cord was identified and used for fetal exsanguination with both maternal and fetal baboon under general anesthesia as approved by the American Veterinary Medical Association Panel on Euthanasia. Postoperatively, mothers were placed in individual cages and watched until they were upright under their own power. Maternal analgesia was administered for

3 days (buprenorphine hydrochloride injection; Hospira, Inc., Lake Forest, IL, USA; 0.015 mg/kg/day) post-operatively or longer if indicated. They were returned to their group cage two weeks postoperatively.

Animals were individually fed to enable precise regulation of intake either between 7:00 am and 9:00 am or 11:00 am and 1:00 pm as described in detail elsewhere [11]. Water was continuously available in each feeding cage (Lixit, Napa, California), and the animals were fed Purina Monkey Diet 5038 (Purina, St Louis, Missouri). For this study, we selected samples representing the entire primate lifespan, from neonate to old age.

Strepsirrhine primates

Strepsirrhini is a suborder of primates that includes the lemuriform primates, which consist of the lemurs of Madagascar, pottos and galagos from Africa, and the lorises from Southeast Asia. Lemuroids and lorisoids together form the more ancestral sister clade to all other living primates. As such, they lend unparalleled power to any comparative study within the primate clade.

For this study, we selected a total of 91 samples from individuals representing 26 strepsirrhine species, in most cases, the entire lifespan, from immature (infant or juvenile) to senile stages: 68 samples from peripheral blood, 23 samples from skin. The strepsirrhine primates (suborders Lemuriformes and Lorisiformes) used in this study were from the Duke Lemur Center (DLC) in Durham, NC (USA). The Duke Lemur Center is certified by both the Association for Assessment and Accreditation of Laboratory Animal Care and the American Zoological Association. The animal handling and sample collection procedures in this study were performed by a veterinarian after review and

approval by the Duke Institutional Animal Care and Use Committee and the DLC Research Committee. Both housing and sample collection met or exceeded all standards of the Public Health Service's "Policy on the Humane Care and Use of Laboratory Animals". The lemurs are housed in comparable social and housing conditions, habituated to human presence, and individually identifiable. The DLC also maintains a large collection of banked tissues, deriving from routine veterinary procedures and necropsies, amassed over the Center's 55-year history. Detailed records of life and medical history, reproduction, and social-group membership are digitally maintained [12].

Peripheral blood was collected through venipuncture with standard procedures, either during a routine veterinary procedure or at time of necropsy. Skin tissues were collected during necropsies. Whole blood was preserved in either EDTA or Lithium Heparin and stored at -80oC. Skin tissues were either frozen directly at -80oC or were first flash frozen and then stored at -80oC.

We profiled the following species: Cheirogaleus medius (Fat-tailed dwarf lemur), Daubentonia madagascariensis (Aye-aye), Eulemur albifrons (White-headed lemur), Eulemur collaris (Collared brown lemur), Eulemur coronatus (Crowned lemur), Eulemur flavifrons (Blue-eyed black lemur), Eulemur fulvus (Brown lemur), Eulemur macaco (Black lemur), Eulemur mongoz (Mongoose lemur), Eulemur rubriventer (Red-bellied lemur), Eulemur rufus (Red-fronted lemur), Eulemur sanfordi (Sanford's brown lemur), Galago moholi(South African galago), Hapalemur griseus (Bamboo lemur), Lemur catta (Ring-tailed lemur), Loris tardigradus (Slender loris), Microcebus murinus (Gray mouse lemur), Mirza zaza (Northern giant mouse lemur), Nycticebus coucang (Slow loris), Otolemur crassicaudatus (Greater galago), Perodicticus potto (Potto), Propithecus diadema

(Diademed sifaka), Propithecus tattersalli (Golden-crowned sifaka), Varecia rubra (Red ruffed lemur).

Primates from Busch Gardens

The blood samples from chimpanzees (Pan troglodytes, n=2), gorillas (Gorilla, n=3) Orangutan (n=1, Pongo pygmaeus), red ruffed lemur (n=1, Varecia variegata), and White-fronted marmoset (n=1, Callithrix geoffroyi) were opportunistically collected and banked during routine health exams from these zoo-based animals located at Busch Gardens Tampa (Tampa, Florida).

Human tissue samples

We analyzed previously generated methylation data from n=1352 human tissue samples (adipose, blood, bone marrow, dermis, epidermis, heart, keratinocytes, fibroblasts, kidney, liver, lung, lymph node, muscle, pituitary, skin, spleen) from individuals whose ages ranged from 0 to 101 years. Out of the 1352 tissues, n=655 came from women.

The tissue samples came from four sources: tissue and organ samples from the National NeuroAIDS Tissue Consortium [13,14], Blood samples from the Cape Town Adolescent Antiretroviral Cohort study [15] and the PEG study [16], skin and other primary cells provided by Ken Raj [17]. Ethics approval (IRB#15-001454, IRB#16-000471, IRB#18-000315, IRB#16-002028).

Vervet monkeys [7]

All animals used in this study were Caribbean-origin vervet monkeys (*Chlorocebus sabaeus*) from the VRC at Wake Forest School of Medicine. The VRC colony is an

extended multigenerational pedigree established from 57 founders imported from the islands of St. Kitts and Nevis in the West Indies. The introduction of new animals to the pedigree ended in the mid-1980s (Jasinska et al. 2013). The colony members are socially reared in extended family groups mimicking the natural social composition of vervet monkey troops in the wild. Group sizes range from 11 to 23 animals, with one or two intact adult males included in each group. Unfamiliar males are rotated into each group every 3–5 years. The pedigree structure is genetically confirmed (Huang et al. 2015). All colony-born vervets have known chronological age accurate to 1 day.

Beyond applications in aging studies, animals from VRC are used in a wide range of research in areas such as the efficacy and enhancement of vaccines for infectious diseases, e.g., influenza and dengue (Kim et al. 2015; Holbrook et al. 2016; Briggs et al. 2014), investigations of diabetes, metabolic disease and obesity (Kavanagh et al. 2017; Kavanagh et al. 2016; Kavanagh et al. 2013); and the development of novel non-invasive biomedical imaging methodologies (Prabhakaran et al. 2017; Maldjian et al. 2014).

Ethics statement

The Wake Forest School of Medicine facilities are certified by the Association for Assessment and Accreditation of Laboratory Animal Care. The animal handling and sample collection procedures in this study were performed by a veterinarian after review and approval by the UCLA and VA Institutional Animal Care and Use Committees. Both housing and sample collection were in compliance with the US National Research Council Committee's Guidelines for Care and Use of Laboratory Animals (National Research Council et al. 2011) and met or exceeded all standards of the Public Health Service's

“Policy on the Humane Care and Use of Laboratory Animals” (Office of Laboratory Animal Welfare n.d.).

Vervet tissue samples

For this study, we selected a total of 240 samples representing the entire vervet lifespan, from neonatal to senile stages: 144 samples from the peripheral blood, 48 samples from the liver, and 48 samples from the cortical brain area BA10. The brains were perfused to remove blood prior to dissection (Jasinska et al. 2017). The targeted brain area BA10 was very small, and brain samples were dissected as bulk tissues, collecting, to the extent feasible without the benefit of microscopy, the full thickness of the cortex while avoiding the underlying white matter (Jasinska et al. 2017). One outlier blood sample (202943350003_R03C01 from animal 1992020) was excluded from analysis on the basis of the DNAm profile. The remaining 143 blood samples included 14 pairs of biological replicates collected from 14 individuals at two different time points 3.9–10.93 years apart. Peripheral blood was collected through venipuncture with standard procedures. Liver and brain cortical tissues were collected during necropsies (Jasinska et al. 2017).

Genomic DNA was isolated from blood and liver samples primarily through Puregene chemistry (Qiagen). DNA from the liver was extracted manually and that from the blood was extracted with an automated Autopure LS system (Qiagen). DNA was extracted from old liver tissues and clotted blood samples manually with a QIAamp DNA Blood Midi Kit and DNeasy Tissue Kit according to the manufacturer’s protocol (Qiagen, Valencia, CA). DNA from BA10 was extracted on an automated nucleic acid extraction platform AnaPrep

(Biochain) with a magnetic bead-based extraction method and Tissue DNA Extraction Kit (AnaPrep).

Common marmosets [8]

Animal care and maintenance

All marmosets used in this research were housed at the Barshop Institute for Longevity and Aging Studies at UT Health San Antonio (UTHSA). The Institutional Animal Care and Use Committee (IACUC) of UTHSA is responsible for monitoring housing and animal condition regularly to ensure all guidelines are met for the safety and health of the animals. This research was reviewed and approved by the UTHSA IACUC and experiments were conducted in compliance with the US Public Health Service's Policy on Humane Care and Use of Laboratory Animals and the Guide for the Care and Use of Laboratory Animals and adhered to the American Society of Primatologists (ASP) principles for the ethical treatment of non-human primates. Animals used in this study were based on age as well as a record of relatively good health as assessed by veterinary examination. Animals in this study were born either at UTHSCSA or transferred from the Southwest National Primate Research Center (SNPRC) in San Antonio TX. At UTHSA, animals were maintained using a modified specific pathogen-free barrier facility [18].
Each animal received three diet choices daily provided ad libitum: Harlan Teklad purified marmoset diet (TD99468), Mazuri Callitrichid gel diet (5MI5) and ZuPreem.

Blood draws and clinical blood counts/chemistry: All blood draws were taken during the morning hours of 08:00-11:00 from fed, non-anesthetized animals restrained in a custom

assembly. Femoral vein blood collection (1.0-2.0 mL) was performed on each animal and blood was placed into PAXgene blood collection tubes (Qiagen). Whole blood was frozen and stored at -80˚ C for shipment to UCLA for methylation analysis.

Study samples

For these studies we selected samples representing the entire primate lifespan, from neonate to old age.

Genomic DNA was isolated from tissue samples mostly using Puregene chemistry (Qiagen). DNA from the liver was extracted manually and from blood using an automated Autopure LS system (Qiagen). From old liver tissues and clotted blood samples DNA was extracted manually using QIAamp DNA Blood Midi Kit and the DNeasy Tissue Kit according to manufacturer's protocol (Qiagen, Valencia, CA). DNA from BA10 was extracted on an automated nucleic acid extraction platform Anaprep (Biochain) using a magnetic bead-based extraction method and Tissue DNA Extraction Kit (AnaPrep).

Rhesus macaque [6]

In total, we analyzed N=281 rhesus macaque tissue samples from 8 different sources of DNA. The rhesus monkeys have been housed continuously at the NIH Animal Center, Poolesville, MD. The animal center is fully accredited by the American Association for Accreditation of Laboratory Animal Care, and all procedures were approved by the Animal Care and Use Committee of the NIA Intramural Program. Monkeys were of a heterogeneous genetic background, both Chinese and Indian origin.

Monkeys were housed individually in standard nonhuman primate caging on a 12h light/12h dark cycle, room temperature 78+/-2 degrees humidity at 60+/-20%. All monkeys had extensive visual, auditory, and olfactory but limited tactile contact with monkeys

housed in the same room. Monkeys received 2 meals per day at estimated ad libitum levels throughout the study. Water was always available ad libitum. Monkeys were monitored minimally 3 times daily by trained animal care staff.

Sample Collection

Monkeys were fasted overnight, approximately 16-18 hours. Monkeys were anesthetized with either Ketamine, 7-10 mg/kg, IM or Telazol, 3-5 mg/kg, IM. Blood samples were obtained by venipuncture of the femoral vein using a vacutainer and EDTA tubes. Samples were immediately placed on dry ice and stored at -80 degrees. Skin samples were collected at the same time from an alcohol-wiped area of the back between the shoulder blades. Omental fat, kidney, liver, lung, skeletal muscle, and brain cortex were collected during necropsies scheduled for other study purposes. At that time, tissues were flash frozen in liquid nitrogen following collection and stored at -80 degrees. These tissues were selected for use based on having matching blood samples. None of the monkeys were sacrificed for this study.

M2. Prairie voles [19]

Ethics

All experimental procedures were conducted and approved by the Institutional Animal Care and Use Committee (IACUC) of Cornell University (2013-0102) and were in accordance with the guidelines set forth by the National Institutes of Health.

Prairie vole colony

Male and female prairie voles (*Microtus ochrogaster*) were produced from laboratory-bred colonies at Cornell University, from breeding pairs that were offspring of wild caught animals captured in Champagne County, Illinois, USA. Voles are weaned and housed with littermates on postnatal day (PND) 21, and then housed with same-sex littermates

after PND42-45. All animals received rodent chow (Laboratory Rodent Diet 5001, LabDiet, St. Louis, MO, USA) and water *ad libitum* and were maintained under standard laboratory conditions (14L:10D cycle, lights on at 08:00, 20 ± 2 °C) in transparent polycarbonate cages (29 x 18 x 13 cm) lined with Sani-chip bedding and provided nesting material.

Prairie vole tissue sample collection

Ear, liver, and brain samples from the Cornell University prairie vole colony were collected from 48 male and female prairie voles at various life stages: neonatal (<1 month old), sub-adult (2-4 months old), mature adult (4-10 months old), and middle aged/old adult (>10 months old). The pair bonded male and female prairie voles used in our study cohabitated with their partners for several months and produced at least three generations of litters. Animals were euthanized via rapid decapitation, their tissues rapidly extracted and frozen on dry ice before being stored at -80C until further processing for genomic DNA extraction. Brains were coronally sectioned and brain regions from the pair bonding circuit (PBC) were micro-dissected and pooled for each animal. The PBC brain regions included the prefrontal cortex, nucleus accumbens, lateral septum, ventral pallidum, and medial amygdala, and ventral tegmental area [20]. Genomic DNA was isolated and purified using the phenol-chloroform extraction and ethanol precipitation method. A total of 144 tissue samples were collected and processed for DNA methylation analysis. One animal was removed from the study due to a mismatch with the reported sex and our DNA methylation-based sex estimator.

Peromyscus [21]

Deer mice are maintained as outbred, genetically diverse closed colonies in the Peromyscus Genetic Stock center of the University of South Carolina. The study was

approved by the Institutional Animal Care and Use Committee (IACUC) of the UofSC (protocol #: 2356-101506-042720) and were in accordance with the guidelines set forth by the National Institutes of Health. DNA was isolated from live animals by tail snips, or upon sacrifice from livers and brains by using DNeasy DNA isolation kit (Qiagen).

M3. Horses [22]

Ethics

This collection protocol was approved by the UC Davis Institutional Animal Care and Use Committee (Protocol#19037). All collection protocols were approved by the UC Davis Institutional Animal Care and Use Committee (Protocols #20751 and 21455, respectively).

Samples

We generated DNA methylation data from n=42 different horse tissues collected at necropsy. The tissue atlas was generated from two Thoroughbred mares as part of the FAANG initiative [23], with the following tissues profiled: adipose (gluteal), adrenal cortex, blood (PBMCs; only n=1 mare), cartilage (only n=1 mare), cecum, cerebellum (2 samples each from lateral hemisphere and vermis), frontal cortex, duodenum, fibroblast, heart (2 samples each from the right atrium, left atrium, right ventricle, left ventricle), hypothalamus, ileum, jejunum, keratinocyte, kidney (kidney cortex and medulla), lamina, larynx (i.e. cricoarytenoideus dorsalis muscle), liver, lung, mammary gland, mitral valve of the heart, skeletal muscle (gluteal muscle and longissimus muscle), occipital cortex, ovary, parietal cortex, pituitary, sacrocaudalis dorsalis muscle, skin, spinal cord (C1 and T8), spleen, suspensory ligament, temporal cortex, tendon (deep digital flexor tendon and superficial digital flexor tendon), uterus [23].

Blood samples were collected via venipuncture into EDTA tubes from across 24 different horse breeds (buffy coat). Most of the samples were from the Thoroughbred (TB) (n=79) and American Quarter Horse breeds (QH, n=62). For the following breeds, we had between one and six blood samples: Andalusian, Appaloosa, Arabian, Dutch Warmblood, Hanoverian, Holsteiner, Irish Sport Horse, Lipizzaner, Lusitano, mixed breed, Oldenburg, Paint or Paint cross, Percheron, Shire, Standardbred, Warmblood and Welsh Pony. The n=49 liver samples originated from necropsy collections of horses across 19 different breeds, with most of the liver samples from QHs (n=20).

M4. Naked mole-rat [24]

The NMR tissue samples were provided by two different labs: 1) Vera Gorbunova and Andrei Seluanov from the University of Rochester and 2) Chris Faulkes from the Queen Mary, University of London.

<u>Ethics for 1)</u>

All animal experiments were approved and performed in accordance with guidelines set up by the University of Rochester Committee on Animal Resources with protocol number 2009-054 (naked mole-rat).

<u>Ethics for 2)</u>

Naked mole-rats were maintained in the Biological Services Unit at Queen Mary University of London in accordance with UK Government Animal Testing and Research Guidance.

<u>Animals from the University of Rochester</u>

Naked mole-rats were from the University of Rochester colonies. All animals in the colonies are microchipped and their ages are recorded. Housing conditions are described previously [25]. All tissues except for skin biopsies and blood were obtained from frozen

tissue collection at the University of Rochester from healthy animals that were euthanized for other studies. Skin biopsies (2 mm punch) were collected from the backs of the animals under local anesthesia. Blood samples were collected from the tails. The n=3 induced pluripotent stem cells from NMR were generated as described in [26]. As a control set for the iPS study, we used n=3 fibroblasts samples from animals aged 1 and 2. Genomic DNA was extracted using Qiagen DNeasy Blood and Tissue kit and quantified using Nanodrop and Qubit.als

Study Animals from Queen Mary University

The tissues used in this study were obtained from post-mortem specimens from animals free from disease in compliance with national (Home Office) and institutional procedures and guidelines. Because sample collection was from post-mortem material, additional local ethical approval was not required for this study. Tissue samples were snap frozen in liquid nitrogen following dissection and transferred for storage at -80ºC.

M5. Sheep [27]

Sheep DNA samples for this study were derived from two distinct tissues from two strains: ear tissue from New Zealand Merino, and blood from South Australian Merino.

Ethics for ear samples

Ear tissue was obtained from females and both intact and castrated male Merino sheep during routine on-farm ear tagging procedures in Central Otago, New Zealand. As a small piece of tissue is removed during the ear tagging process that is usually discarded by the farmer, we were able to source tissue and record the year of birth without altering animal experience, in accordance with the New Zealand Animal Welfare Act (1999) and the National Animal Ethics Advisory Committee (NAEAC) Occasional Paper No 2.

Ethics for blood samples

All protocols involving OVT73 sheep were approved by the Primary Industries and Regions South Australia (PIRSA, Approval number 19/02) Animal Ethics Committee with oversight from the University of Auckland Animal Ethics Committee.

Sheep ear

The exact date of birth for each sheep is unknown, however, this was estimated to be the 18th of October each year, according to the date at which rams were put out with ewes (May 10th of each year), a predicted mean latency until mating of 1Tab2 days, and the mean gestation period from a range of sheep breeds (149 days). Mass of yearlings was recorded by the farmer for both castrated and intact male sheep at 6.5 months of age, as a part of routine growth assessment. In total, ear tissue from 138 female sheep aged 1 month to 9.1 years and 126 male sheep (63 intact, 63 castrates) aged 6 months to 5.8 years was collected and subjected to DNA extraction.

DNA was extracted from ear punch tissue using a Bio-On-Magnetic-Beads (BOMB) protocol which isolates DNA molecules using solid-phase reversible immobilisation (SPRI) beads. Approximately 3 mm punches of ear tissue were lysed in 200 µL TNES buffer (100 mM Tris, 25 mM NaCl, 10 mM EDTA, 10% w/v SDS), supplemented with 5 µL 20 mg/mL Proteinase K and 2 µL RNAse A and incubated overnight at 55 °C as per BOMB protocols. The remainder of the protocol was appropriately scaled to maximise DNA output while maintaining the necessary 2:3:4 ratio of beads:lysate:isopropanol. As such, 40 µL cell lysate, 80 µL 1.5X GITC (guanidinium thiocyanate), 40 µL TE-diluted Sera-Mag Magnetic SpeedBeads (GE Healthcare, GEHE45152105050250) and 80 µL isopropanol were combined. After allowing DNA to bind the SPRI beads, tubes were

placed on a neodymium magnetic rack for ~5 minutes until the solution clarified and supernatant was removed. Beads were washed 1x with isopropanol and 2x with 70% ethanol, and then left to air dry on the magnetic rack. 25 µL of MilliQ H_2O was added to resuspend beads, and tubes were removed from the rack to allow DNA elution. Tubes were once again set onto the magnets, and the clarified solution (containing DNA) was collected.

DNA was quantified using the Quant-iT PicoGreen dsDNA assay kit (ThermoFisher Scientific, cat # P11496). 1 µL DNA sample was added to 14 µL TE diluted PicoGreen in MicroAmp optical 96-well plates (ThermoFisher Scientific, cat #N8010560) as per manufacturer directions, sealed, and placed into a QuantStudio qPCR machine for analysis. Samples with DNA content greater than the target quantity of 25 ng/µL were diluted with MilliQ.

Sheep blood

DNA methylation was analysed in DNA extracted from the blood of 153 South Australian Merino sheep samples (80 transgenic Huntington's disease model sheep (OVT73 line) [30] and 73 age-matched controls) aged from 2.9 to 7.0 years. All protocols involving OVT73 sheep were approved by the Primary Industries and Regions South Australia (PIRSA, Approval number 19/02) Animal Ethics Committee with oversight from the University of Auckland Animal Ethics Committee. The epigenetic age of the transgenic sheep carrying the *HTT* gene was not significantly different from controls (p=0.30, Mann-Whitney U test), therefore the data derived from these animals was subsequently treated as one dataset.

300 µL thawed blood samples were treated with 2 rounds of red cell lysis buffer (300 mM Sucrose, 5 mM $MgCl_2$, 10 mM Tris pH8, 1% Triton X-100) for 10 minutes on ice, 10-minute centrifugation at 1,800 RCF, and supernatant removed between each buffer treatment. The resulting cell pellet was incubated in a cell digestion buffer (2.4 mM EDTA, 75 mM NaCl, 0.5 % SDS) and Proteinase K (500 µg/ml) at 50 °C for two hours. Phenol:Chloroform:Isoamyl alcohol (PCI, 25:24:1; pH8) was added at equal volumes, mixed by inversion, and placed in the centrifuge for 5 minutes at 14,000 RPM at room temperature (repeated if necessary). The supernatant was collected and combined with 100% ethanol at 2x volume, allowing precipitation of DNA. Ethanol was removed and evaporated, and 50 µL TE buffer (pH8) was added to resuspend genomic DNA. DNA sample concentration was initially quantified using a nanodrop, followed by Qubit.

M6. Pig [28]
Ethics

All animal procedures were approved by the University of Illinois and University of Wisconsin Institutional Animal Care and Use Committee, and all animals received humane care according to the criteria outlined in the Guide for the Care and Use of Laboratory Animals.

Porcine pan tissue samples

Porcine whole blood samples (n=146) were collected from female Large White X Landrace crossbred domestic pigs (n=84, age range 11 – 2,285 days) and Wisconsin Miniature Swine™ (n=60, age range 8 – 1,880 days) at the University of Wisconsin-Madison. Whole blood (n=16) and tissue samples (bladder, frontal cortex, kidney, liver, lung; n=16/tissue type) were collected from 16 Large White X Minnesota minipig crossbred pigs (n=9 female, n=8 male, age range 29 – 1,447 days) at the University of

Illinois at Urbana-Champaign. All blood samples were collected in EDTA tubes, aliquoted, and flash frozen in liquid nitrogen within 10 minutes of collection. Tissue samples were collected, and flash frozen within 10 minutes of euthanasia. All samples were stored at -80 until processing. Samples were shipped to the University of California, Los Angeles Technology Center for Genomics & Bioinformatics for DNA extraction and generation of DNA methylation data.

M7. Odontocete species [29-31]

<u>Ethics</u>

The study was authorized by the management of each institution and was reviewed by their respective zoo research and animal use committees.

<u>Study Animals</u>

For model development, our study population included 293 animals from nine species of odontocetes, of which the majority were from four species including beluga (n = 66), Pacific white-sided dolphins (n = 17), killer whales (n = 37) and bottlenose dolphins (n = 137), housed at nine Association of Zoos and Aquarium (AZA), Alliance for Marine Mammal Parks and Aquariums or Japanese AZA accredited zoological institutions. Known (77.8%) or estimated (based on length at capture or rescue for stranded animals) birth dates were provided by each housing institution. In addition to zoo-based animals, we included 19 skin samples from free-ranging Norwegian killer whales to the training set because the ages of these animals could be estimated within sufficient accuracy (expected error less than 8%) on the basis of several lines of evidence including GLG counts (n = 3), whereby 1 GLG was assigned per year of age, length at necropsy (n = 1), juvenile at first identification (n = 2) or minimum age estimation based on length and maturity at first sighting (n = 13). The remaining 26 animals' ages could not be estimated

within our accuracy parameters and skin samples from these animals were used to demonstrate model application for determining age and sex in wild animals.

Sample collection

Blood samples (0.5 ml min) were collected either voluntarily from the peripheral periarterial venous rete on the ventral tail fluke using an 18 to 22 gauge winged blood collection set or attached to a vacutainer collection system. Blood was collected by either the veterinary technician or veterinarian on staff and into BD Vacutainers (Becton Dickinson, Franklin Lakes, NJ) containing EDTA. Samples were inverted in the Vacutainer a minimum of 10 times and then frozen at -80°C until further testing.

Skin samples (~0.5 gm) were collected either under stimulus control or manual restraint using a sterile disposable dermal curette (Miltex, Integra Life Sciences Corp.,York, PA) from a location just posterolateral of the dorsal fin overlying the epaxial muscle. Prior to collection, a cold pack was placed on the site for several minutes prior to sampling to numb the sample site. Skin samples were placed into sterile cryovials (Nunc® Cryotubes, MilliporeSigma Corp., St. Louis, MO) and stored at -80°C until shipment on dry ice. Skin samples from non-living animals were obtained from frozen (-80°C) specimens that had been previously collected and stored during standard necropsy procedures.

Skin samples were sectioned from previously collected killer whale biopsy samples of 45 unique individuals (photo-identified) collected in August and November 2017 and from April through July 2018 in northern Norway (Jourdain, 2017). The killer whales were biopsied using an ARTS darting system (Restech, Bodø, Norway) and 25 × 9 mm or 40 × 9 mm stainless steel tips in 2017, and with an injection gun (Pneu-Dart Inc., Williamsport, PA) and 25 × 7 mm tips in 2018 as previously described (Jourdain, 2017).

In addition, tissue samples were collected from six dead, stranded killer whales, and one other individual by-caught in a herring purse-seine, in northern Norway between 2015 and 2017 (E. Jourdain, unpublished data). Skin samples were collected from the region directly posterior to the dorsal fin and stored at -20°C until analysis.

M8. Beluga whales [31]

Ethics

Skin tissue samples were collected from carcasses of beluga whales that were beach-cast, stranded dead, or taken during subsistence hunting from 1992 to 2015 in Cook Inlet, Alaska, USA (NMFS Research Permit 932-1905-00/MA-009526 through the Marine Mammal Health and Stranding Response Program).

Skin samples

Skin samples were preserved in a salt and dimethyl sulfoxide (DMSO) solution and archived at NOAA's Southwest Fisheries Science Center in La Jolla, California, USA. A total of 69 individuals were selected for the clock calibration dataset, and their chronological ages were estimated by counting tooth growth layer groups. The final calibration dataset included 67 individuals due to inconsistent molecular sex data. Teeth were analyzed by at least two readers using methods validated in the study from Lockyer et al.[32], and a consensus age provided by NOAA was used in this study. When individuals were represented by multiple teeth in the dataset, the oldest age estimate was used to mitigate error from tooth wear (*e.g.,* the count from the tooth with the greatest number of growth layer groups).

Samples of skin tissue from living CI beluga whales were collected with a biopsy dart in 2016, 2017, and 2018 (NMFS ESA/MMPA Permit #20465; McGuire et al.,[33]). Biopsy samples were frozen in the field in liquid nitrogen and later subsampled at the NOAA

Alaska Fisheries Science Center in Seattle, Washington, USA. Genomic DNA was extracted from tissue samples using a standard phenol-chloroform protocol. Extracted DNA was treated with RNAse A (1 µL of 1 mg/mL added to samples of 100 µL for 30 minutes at room temperature) and then purified and concentrated using a DNA Clean and Concentrator-5 Kit (Zymo Research Corp., USA). The concentration of genomic DNA was measured on a QUBIT 4 fluorometer (ThermoFisher Scientific, USA).

M9. Killer whales and bowhead whales

Ethnics

For bowhead (*Balaena mysticetus*) subsistence hunts, indigenous hunters had the authorization to conduct hunts and collected samples on behalf of Fisheries and Oceans Canada. Bowhead whale biopsy samples were collected in 2019 under Fisheries and Oceans Canada (DFO) license to Fish for Scientific Purposes (LFSP) S-19/20-1007-NU and Animal Care approval (AUP) FWI-ACC-2019-14. Skin samples from eastern North Pacific killer whales (*Orcinus orca*) were collected as previously described (Ford et al. 2018b) under NMFS General Authorization No. 781–1725, and scientific research permits 781-1824-01, 16163, 532- 1822-00, 532– 1822, 10045, 18786-03, 545-1488, 545-1761, and 15616.

Killer whale biopsy samples & DNA extraction

Killer whales in the eastern North Pacific are among the most intensively studied cetacean populations globally. The so-called ‘resident’ killer whale populations inhabiting coastal waters from California to Alaska comprise individually identified whales that have been studied for over 40 years [34-38]. These longitudinal studies and reliable identification of individual whales within populations through annual photographic census data has provided unique insight into population dynamics and demographics. The resolution

provided by annual documentation of births, ages at physical maturity (males) and age at first parturition (females) provides an unparalleled opportunity to validate epigenetic models of age from whale skin.

The killer whale validation dataset is rare in both the representation across age classes and the number of known-age samples from wild populations based on direct observations [34,35,39-41], providing an ideal training set for validating an epigenetic clock. Killer whale longevity is estimated to be 80 or 90 years [40,41] and samples in the current dataset represent individual whales ranging from age 0 (neonate) to 79 yrs (estimated). Reflecting killer whale age-related mortality patterns [41], the number of individuals representing older age classes diminishes as expected with n = 11 whales estimated be > 50 yrs old based on size, physical and reproductive maturity at the time of first observation.

Ages of individual killer whales were determined based on the sex and size of the animal during the year that it was first documented, following Olesiuk et al. (1990). Whales born during the study (post-1974) were aged in reference to the year in which they were born. Ages for whales that were juveniles or adults when field observations began in the early 1970s were aged based on the year they reached physical maturity or, for females, the year they gave birth to their first viable offspring [40]. Confidence estimates (0% - 100%) were assigned to each individual sampled whale included in the dataset reflecting the certainty around age estimates, frequency of encounters with the individual and age or state of physical maturity at the time of first identification [35,40,41]. Genetic samples from 131 killer whales were included in the dataset representing 118 different killer whales.

Sampled killer whales include individuals from two different ‘resident’ killer whale populations (Southern residents and Gulf of Alaska residents) as well as 11 individuals from an eastern North Pacific ‘transient’ killer whale population. Killer whale samples represented individuals ranging from age 0 yrs (neonate or fetus; 100% certainty) to age 75+ yrs (50% certainty).

Epidermal samples were collected from live killer whales using remote dart biopsy methods, and from dead stranded animals during routine post-mortem necropsy protocols [42,43]. Sampled individuals included in the methylation analyses include identified individual whales from the Southern Resident, Alaska Resident and Transient killer whale populations. Identities of individual whales were recorded photographically whenever possible at the time of sample collection. Total genomic DNA was extracted from skin biopsies either using a silica-membrane kit following manufacturer’s protocols (DNeasy Blood and Tissue kit, Qiagen, Valencia, CA), or following a standard proteinase K phenol/chloroform/isoamyl alcohol extraction protocol [44].

Bowhead whale biopsy samples & age estimates

Bowhead whales are very slow-growing and extremely long-lived baleen species that undergo periods with rapid growth (as a fetus), pauses in growth (ages 1 to about 6-7) and slow growth (age 8+) which gradually slows even more as they age [45]. For this reason, accurate age estimates of individual bowhead whales are typically limited to early life stages when multiple sources of information are available.

Estimating age for individual bowhead whales requires a combination of several different types of data including body length, length of the longest baleen plate, body condition measurements, other morphological measurements that include frequency of scars, color

of the peduncle and chin region, and aspartic acid racemization (AAR) analyses from eye lenses. Despite multiple data sources, bowhead age can only be approximated for whales more than approximately 7 years old because the variation in size at age is many times larger than the annual growth rate of individual whales. Whales 2-7 years old cannot be accurately aged without information on the longest baleen plate, which is only available for harvested whales, although body condition assessed from photogrammetry provides information to assist with age estimation.

Bowhead whale skin samples were collected from remote biopsy using a crossbow during photographic studies conducted in Cumberland Sound in 2017 and 2019, and samples collected during subsistence harvest (1996 - 2016). Data available for age estimates were limited to body length, body condition, frequency of scars, and color of the peduncle region for biopsy samples. Skin samples collected during subsistence harvest included age estimates based on body length, length of the longest baleen plate, notes on scars or peduncle color and AAR ages. Where multiple sources of data were available, ages obtained from AAR or age-at-length estimates were adjusted to take account of the additional data as below.

Age at length was estimated using data in Figure 7 of Lubetkin, et al. [46] with estimation for gradually reduced growth rates for whales older than 60 years old as suggested by their data and those of Koski et al. (1992). Yearlings were confirmed among the smallest whales by body condition using criteria in Koski et al. (2010). Body condition was also used to adjust the age of whales 2-7 years as determined by length. For example, the youngest of these whales (i.e., 2 years old) appeared to have the poorest body condition and body condition improved as young whales aged.

Age based on aspartic acid racemization was determined for 11 harvested bowhead whales (4 females; 7 males). Both eyeballs were stored at –20°C immediately after dissection from harvested whales, and one eyeball per whale was subsequently used for age estimation. Dissection of eye lenses and age estimation by the aspartic acid racemization (AAR) technique was performed using methods described in [47].

M10. Humpback whales

Ethics

Skin samples were collected by the Center for Coastal Studies under research permits issued by the U.S., National Marine Fisheries Service (21485, 16325, 20465, 14245, 633-1483, 633-1778, 932-1905), the Canadian Department of Fisheries and Oceans and IACUC #NWAK-18-02.

Humpback sample collection

Skin samples were collected from live North Atlantic humpback whales by biopsy sampling techniques[48]. Sampling was performed in the Gulf of Maine between 2003 and 2020 under the authorization of nationally issued research permits. Samples were refrigerated or frozen in the field and then archived at -80C without chemical preservative. Year of birth can be determined precisely for humpback whales first encountered as calves during an obligatory period of maternal dependency[49,50]. Otherwise, there are no reliable outward indicators of chronological age in this species. We therefore selected known-age samples for this study based on data from a long-term study of individual whales. The identity of each sampled individual was confirmed through photo-identification techniques[51] using a reference catalog of the Gulf of Maine population curated by the Center for Coastal Studies (Provincetown, MA). For individuals first seen as calves, age at sampling was the number of years between sampling and birth.

However, the exact date of birth in that year was not known for any individual, and sampling was performed over a wide window (April through November) outside of the winter breeding period. We therefore used an estimated day of birth at the peak of the winter breeding season for all individuals (February 15) to refine age at the time of sampling.

Photo-identification research on this population did not begin until the 1970s and individuals cataloged since time were not all seen first as calves. Thus, chronological age is unknown for many individuals, including the oldest whales in the population. We therefore selected samples from individuals with long sighting spans (as long or longer than the upper 20% of the available known-age data) to clarify epigenetic age patterns at and beyond the top of the validated age range. In these cases, we calculated a minimum age at sampling based on the fact the individual could not have been born later than the year before their first sighting. However, such whales could have been born in any earlier year and so this provided only a minimum bound on their chronological age.

The sex of sampled individuals was known independently from molecular genetic analysis[52], in some cases supplemented by observations of the genital slit[53] or calving history.

M11. Cats [54]

Ethics

Sample collection was approved by the Clinical Research Ethical Review Board of the RVC (URN: 2019 1947-2).

Feline and other animal blood samples

The DNA archive of the Royal Veterinary College (RVC) was searched for feline ethylenediaminetetraacetic acid (EDTA) blood samples that were residuals from previous

routine hematology testing. Cats were selected to represent the widest age range possible based on the available samples with a uniform distribution across the entire range, available breeds and neutering status. As the samples originated from cats that were presented for veterinary investigation, cats were selected to have no or minimal abnormalities on available laboratory data (hematology, serum biochemistry, endocrinology), reviewed by a board certified veterinary clinical pathologist (BSz). The DNA samples were maintained frozen at -80 °C for various amount of time (0-11 years). Samples from guinea pigs, rabbits, ferrets, and alpacas were also residual samples from routine patients presented for veterinary care. Sample collection was approved by the Clinical Research Ethical Review Board of the RVC (URN: 2019 1947-2). Genomic DNA from cat blood was extracted using the Zymo DNA extraction kit according to the manufacturer's instructions. DNA was eluted in water and quantified with picogreen kit according to the instructions provided.

Non-domestic cat species

Blood samples from cheetah (Latin name *Acinonyx jubatus*), lion (*Panthera leo nubica*), and tiger (*Panthera tigris*) were opportunistically collected and banked during routine health exams from these zoo-based animals located at Busch Gardens and White Oak Conservation.

M12. Elephants [55]

Ethics

This study was authorized by the management of each participating zoo and, where applicable, was reviewed and approved by zoo research committees. In addition, the study received IACUC approval (#18-29) at the NZP; and endorsement from the elephant Taxon Advisory Group and Species Survival Plan.

Study Animals

Our study population included 140 elephants (57 African and 83 Asian) housed in 27 AZA-accredited zoos in North America (including Canada). Known or estimated birthdates were gleaned from each species' studbooks.

Uncertain surrounding age information is encoded in our variable "ConfidenceInAgeEstimate". A value of 90% indicates that the chronological age could be off by around 5 percent. Our analysis omitted animals for whom the confidence in the age estimate was less than 90 percent. For all elephants, the birthdates used in our analyses were taken from those recorded in the regional studbooks maintained through the Association of Zoos and Aquariums Taxon Advisory Group and Species Survival Plan for each species. Elephants had known birthdates if there were captive-born (i.e., they were recorded as having been born in a captive facility in the U.S. or in a range country), or were recorded as having an estimated birthdate if they were imported (i.e., capture location and date of capture was recorded in the studbook). How age was estimated for imported individuals is not described in the studbooks but was most likely done by the broker at the time of importation based on individual morphometric measurements. The majority (>70%) of the North American population is imported as captive breeding as a population management tool was not fully implemented until the late 1990s. In our sample set we had about equal proportion of captive vs imported individuals, although we did have more imported animals reflecting the management history of the population. Our samples are made up of 65 captive-born/known birthdates (21 Africans and 26 Asians), and 77 imported/estimated birthdates (36 African ad 27 Asian).

Whole blood samples from either an ear or leg vein directly into an EDTA tube were collected between 1998 and 2019 during regular veterinary examinations and shipped frozen to the genetics lab at the Smithsonian Conservation Biology Institute Center for Conservation Genomics (SCBI-CCG). The samples were stored in an ultralow freezer (-80ºC) until DNA extraction.

M13. Yellow-bellied marmots [56]

Ethics

Data and samples were collected under the UCLA Institutional Animal Care and Use protocol (2001-191-01, renewed annually) and with permission from the Colorado Parks and Wildlife (TR917, renewed annually).

Samples

All samples were collected as part of a long-term study of a free-living population of yellow-bellied marmots in the Gunnison National Forest, Colorado (USA), where marmots were captured and blood samples collected biweekly during the their active season (May to August).

Individuals were monitored throughout their lives, and chronological age was calculated based on the date at which juveniles first emerged from their natal burrows. We only used female samples because precise age for most adult males is unavailable since males are typically immigrants born elsewhere. We selected 160 whole blood samples from 78 females with varying ages. From these, DNA methylation (DNAm) profiling worked well for 149 samples from 73 females with ages varying from 0.01 to 12.04 years.

M14. Roe deer [57]

Ethics

The protocol of capture and blood sampling under the authority of the Office Français de la Biodiversité (OFB) was approved by the Director of Food, Agriculture and Forest (Prefectoral order 2009–14 from Paris). The land manager of both sites, the Office National des Forêts (ONF), permitted the study of the populations (Partnership Convention ONCFS-ONF dated 2005-12-23). All experiments were performed in accordance with guidelines and regulations of the Ethical Committee of Lyon 1 University (project DR2014-09, June 5, 2014)

Study populations

We sampled roe deer living in two enclosed forests with markedly different environmental contexts: Trois-Fontaines (TF) and Chizé (CH). The Trois-Fontaines Forest (1,360 ha) is located in north-eastern France (48°43’N, 4°55’E) and is characterized by a continental climate, moderately severe winters and warm and rainy summers. This site has rich soils and provides high quality habitat for roe deer. In contrast, the Chizé forest (2,614 ha) is located in western France (46°50’N, 0°25’W) and is characterized by temperate oceanic climate with Mediterranean influences. This site has a low productivity due to poor quality soils and frequent summer droughts, and thereby provides a quite poor habitat for roe deer in most years. Individuals from these two populations have been intensively monitored using a long-term Capture-Mark-Recapture program since 1975 and 1977 (for Trois-Fontaines and Chizé, respectively). In each site, 10-12 days of capture using drive-netting are organized every year between December and March, which allows capturing and measuring about half the population every year. Once a roe deer is captured, its sex and body mass (to the nearest 50g) are recorded, and a basic clinical examination is

performed. All individuals included in our analyses were of known age because they were either caught as newborn in spring (see Delorme et al. 1988 for further details) or as c.a. 8 months old during winter captures, when they still have their milk teeth (most often incisors and always premolars, Flerov 1952).

Roe deer blood samples and DNA extraction

In 2016 and 2017, we collected blood samples (up to 1mL per kg of body mass) from the jugular vein. Within 30 min of sampling, the blood was centrifuged at 3000 g for 10 min and the plasma layer was removed before washing the cells with an equivalent volume of 0.9% w/v NaCl solution. After a second centrifugation, the intermediate buffy coat layer, comprising mainly leukocytes, was collected in a 1.5-mL Eppendorf tube and immediately frozen at −80 °C in a porcos freezer (Telstar SF 8025) until further use.

We extracted genomic DNA from leukocytes using the Macherey-Nagel NucleoSpin® Blood QuickPure kit. DNA purity was assessed using a Nanodrop ND-1000 spectrophotometer (Thermo Scientific, Wilmington DE, USA). For all samples, the purity absorption range was 1.7 - 2.0 for the 260/280 nm ratio and >1.8 for the 260/230 nm ratio. We selected 96 samples by balancing the numbers of individuals among ages, and between populations and sexes. DNA concentration was determined spectrophotometrically using the Qubit assay kit. DNA samples were then diluted in ultrapure water to reach a concentration of ~70 ng.μl^{-1} and displayed in a microplate to complete the DNA methylation protocol (see below). For 6 samples, the concentrations obtained after dilution were too low compared to the expected concentrations of 70 ng/µl and were excluded from the dataset. The 90 roe deer samples analysed in this study correspond to 79 individuals (i.e., 11 individuals were sampled both in 2016 and 2017)

aged from 8 months to 13.5 years of age. This age range encompasses most of the roe deer lifespan as individuals older than 15 years of age are rarely observed in the wild (the oldest age ever recorded for a roe deer monitored in the wild being 17.5 years old.

M15. Zebras [58]

Ethics

Plains zebra samples were collected under a protocol approved by the Research Safety and Animal Welfare Administration, University of California Los Angeles: ARC # 2009-090-31, originally approved in 2009.

Samples

Both whole blood (96) and remote biopsy (24) samples were obtained from a captive population of zebras maintained in a semi-wild state by the Quagga Project [59] in the Western Cape of South Africa. The population was founded in 1989 with 19 wild individuals (9 from Etosha National Park in Namibia, 10 from the Kwazulu-Natal in South Africa). Since its inception, the population has undergone strong selection in an effort to reproduce the phenotype of the extinct quagga subspecies: no stripes on legs and hindquarters, and thinner and paler stripes in the head and barrel region. During sampling, individuals were uniquely identified by their stripe patterns and their ages were derived from studbook information about dates of birth, which were typically known within one month. One exception to this is a tissue sample from a founder that was sampled as a young mare and would have been at least 25 years old at sampling but may have been slightly older. Remote biopsies were taken using an air-powered rifle affixed with a 1 mm wide by 20-25 mm deep biopsy dart and preserved in RNAlater (Qiagen). Blood samples were collected opportunistically during veterinarian visits and preserved in EDTA tubes. Most samples were collected from different individuals, except for two individuals that

each were sampled twice some years apart. All samples were stored at -20 °C. After eliminating samples with low confidence for individual identity and age, we retained 76 blood samples and 20 biopsy samples, totaling 96 zebra samples.

Three zebra data sets were analyzed in our epigenetic models: (1) only blood samples, (2) only biopsy samples, and (3) blood and biopsy samples combined. The Grevy's zebra (n=5) and Somali wild ass (n=7), are samples from zoo-based animals that were opportunistically collected and banked during routine health exams and the DNA methylation profiles from these samples have been reported previously [22].

M16. Rat [60]

The rat tissues came from 4 different labs across three countries:(i) India: Nugenics Research in collaboration with School of Pharmacy SVKM's NMIMS University (K. Singh), (ii) United States: University of Tennessee Health Science Center (H. Chen) and Medical College of Wisconsin (L.C. Solberg Woods), and (iii) Argentina: University of La Plata (R. Goya).

Ethics for (i)

The experimental protocols received approval from the Institutional Animal Ethics Committee under two distinct approval numbers. The first is CPCSEA/IAEC/P-75/2018, and the second is CPCSEA/IAEC/P-6/2018. The second approval was granted in accordance with the norms of the Committee for the Purpose of Control and Supervision of Experiments on Animals (CPCSEA), Government of India, complying with the standard guidelines for handling experimental animals. It specifically pertains to the use of male Sprague Dawley rats, aged 8 weeks (200–250 g) and 20 months (400-450g), procured from the National Institute of Bioscience, Pune, India.

Ethics for (ii)

All procedures were approved by the Institutional Animal Care and Use Committee of the University of Tennessee Health Science Center or the Medical College of Wisconsin and followed the NIH Guide for the Care and Use of Laboratory Animals.

Ethics for (iii)

All experiments with animals were performed in accordance with the Animal Welfare Guidelines of NIH (INIBIOLP's Animal Welfare Assurance No A5647-01) and approved by our Institutional IACUC (Protocol # P05-02-2017). Ethics committee approval number - CPCSEA/IAEC/P-6/2018.

Rats from Nugenics Research Group

Sprague Dawley rats of both sexes were used, from which blood, whole brain, heart and liver were harvested. Two batches or samples were prepared: the first batch was intended for training the epigenetic clock: n=42 blood samples, n=18 whole brain, n=18 heart, n=18 liver samples. The second batch of test set involved n=76 tissue samples (n=22 blood, n=18 liver, n=18 heart, n=18 hypothalamus). The test data were used to evaluate the effect of the treatment in 3 conditions: young (30-week old) and treated old samples (109 weeks old), and untreated old samples (again 109 weeks old). We evaluated 4 sources of DNA: blood (n=18), liver (n=18), heart (n=18) and hypothalamus (n=18). Ethics committee approval number - CPCSEA/IAEC/P-6/2018.

Male Sprague Dawley rats of 8 weeks (200–250 g) and 20 months (400-450g) were procured from the National Institute of Bioscience, Pune, India. Animals were housed in the animal house facility of School of Pharmacy, SVKM's NMIMS University, Mumbai

during the study under standard conditions (12:12 h light: dark cycles, 55-70% of relative humidity) at 22±2°C temperature with free access to water and standard pellet feed (Nutrimix Std-1020, Nutrivet Life Sciences, India). The animals were acclimatized to the laboratory environment for seven days before initiation of the study. The experimental protocol was approved by the Institutional Animal Ethics Committee. The approval number is CPCSEA/IAEC/P-75/2018. Rats were euthanized at different ages by an overdose of isoflurane (> 5%). Trunk blood was collected immediately and stored at −80 °C until processing. 100 μl blood sample was treated with 20 μl Proteinase K and then the volume was adjusted to 220 μl with Phosphate Buffer Saline (PBS) in 1.5 ml or 2 ml microcentrifuge tube. 200 μl buffer AL was mixed thoroughly to this mixture by vortexing, and incubated at 56°C for 10 min. Then 200 μl ethanol (96–100%) was added to the sample and mix thoroughly by vortexing and DNA were extracted using the Qiagen DNeasy blood and tissue kit, Qiagen Cat No./ID: 69504 following manufacturer's instructions. The study protocol was approved through the Institutional Animal Ethics Committee (approval no. CPCSEA/IAEC/P-6/2018) which was formed in accordance with the norms of the Committee for the Purpose of Control and Supervision of Experiments on Animals (CPCSEA), Government of India and complied with standard guidelines on handling of experimental animals.

According to the manufacturer's instructions, 20 μl Proteinase K was pipetted into a 1.5 ml or 2 ml microcentrifuge tube, to this 50–100 μl anticoagulated blood was added and the volume was adjusted to 220 μl with PBS. 200 μl Buffer AL was added and mixed thoroughly by vortexing, and incubated at 56°C for 10 min. Finally, 200 μl ethanol (96–100%) was added to the sample, and mixed thoroughly by vortexing. The mixture was

added to the DNeasy Mini spin column placed in a 2 ml collection tube and centrifuged at 6000 x g for 1 min. Further the DNeasy Mini spin column was placed in a new 2 ml collection tube (previous flow through and collection tube was discarded), 500 µl Buffer AW1 was added and centrifuged for 1 min at 6000 x g. Again, the flow-through and collection tube was discarded. The DNeasy Mini spin column was placed in a new 2 ml collection tube, 500 µl Buffer AW2 was added, and centrifuged for 3 min at 20,000 x g to dry the DNeasy membrane. The flow-through and collection tube was discarded. DNeasy Mini spin column was placed in a clean 1.5 ml or 2 ml microcentrifuge tube and 200 µl Buffer AE was pipetted directly onto the DNeasy membrane. Sample was incubated at room temperature for 1 min, and then centrifuged for 1 min at 6000 x g to elute.

Rats from Tennessee and Wisconsin

Blood samples (n=48): Male and female heterogeneous stock rats were bred at the Medical College of Wisconsin (Solberg Woods Lab) or University of Tennessee Health Science Center (Hao Chen Lab). Heterogeneous Stock (HS) populations were originally developed by breeding together eight inbred strains, followed by maintaining the colony in a manner that minimizes inbreeding, allowing fine-resolution genetic mapping of a variety of complex traits. Rats were euthanized at different ages by an overdose of isoflurane (> 5%). Trunk blood was collected immediately and stored at −80 °C until processing. Blood samples were treated with streptokinase (60-80 IU/200 µl blood, overnight incubation at 37 °C) and DNA was extracted using the QiaAmp Blood Mini Kit (Qiagen Cat No./ID: 51304) following manufacturer's instructions. All procedures were approved by the Institutional Animal Care and Use Committee of the University of Tennessee Health Science Center or the Medical College of Wisconsin and followed the

NIH Guide for the Care and Use of Laboratory Animals. Genomic DNA was isolated from tissue samples mostly using Puregene chemistry (Qiagen). DNA from the liver was extracted manually and from blood using an automated Autopure LS system (Qiagen). From tissues and clotted blood samples DNA was extracted manually using QiaAmp DNA Blood Midi Kit and the DNeasy Tissue Kit according to manufacturer's protocol (Qiagen, Valencia, CA). DNA from BA10 was extracted on an automated nucleic acid extraction platform Anaprep (Biochain) using a magnetic bead-based extraction method and Tissue DNA Extraction Kit (AnaPrep).

Rats from the University of La Plata (R. Goya lab)

Multiple tissues/cell types (adipose, blood, cerebellum, hippocampus, hypothalamus, liver, neocortex, ovaries, pituitary, skin, substantia nigra): Young (3.7 mo., n=11), Late Adults (LA, 8.0 mo., n=9), Middle-Aged (M-A, 15.7 mo., n=6) and Old (25.5 mo., n=14) female Sprague-Dawley (SD) rats, raised in our Institute, were used. Animals were housed in a temperature-controlled room (22 ± 2ºC) on a 12:12 h light/dark cycle. Food and water were available *ad libitum*.

Tissue sample collection

Before sacrifice by decapitation, rats were weighed, blood was withdrawn from the tail veins with the animals under isoflurane anesthesia and collected in tubes containing 10µl EDTA 0.342 mol/l for 500µl blood. The brain was removed carefully severing the optic and trigeminal nerves and the pituitary stalk (not to tear the pituitary gland), weighed and placed on a cold plate. All brain regions were dissected by a single experimenter (see below). The skull was handed over to a second experimenter in charge of dissecting and weighing the adenohypophysis. The rest of the body was handed to other 2 or 3

experimenters who dissected and collected whole ovaries, a sample of liver tissue, adipose tissue, and skin tissue from the distal portion of tails.

Brain region dissection

Prefrontal cortex, hippocampus, hypothalamus, substantia nigra and cerebellum were rapidly dissected on a cold platform to avoid tissue degradation. After dissection, each tissue sample was immediately placed in a 1.5ml tube and momentarily immersed in liquid nitrogen. The brain dissection protocol was as follows. First a frontal coronal cut was made to discard the olfactory bulb, then the cerebellum was detached from the brain and from the medulla oblongata using forceps. To isolate the medial basal hypothalamus (MBH), brains were placed ventral side up and a second coronal cut was made at the center of the median eminence (−3,6 mm referred to bregma). Part of the MBH was taken from the anterior block of the brain and the other part from the posterior block in both cases employing forceps. The hippocampus was dissected from the cortex in both hemispheres using forceps. This procedure was also performed on the anterior and posterior blocks, alternatively placing the brain caudal side up and rostral side up. To dissect the substantia nigra, in each hemisphere a 1-mm thick section of tissue was removed from the posterior part of the brain (−4,6 mm referred to bregma) using forceps. Finally, the anterior block was placed dorsal side up, to separate the prefrontal cortex. With a sharp scalpel, a cut was made 2 mm from the longitudinal fissure, and another cut was made 5 mm from it. Additionally, two perpendicular cuts were made, 3 mm and 6 mm from the most rostral point, obtaining a 9 mm2 block of prefrontal cortex. This procedure was performed in both hemispheres and the two prefrontal regions collected in a code-labeled tube.

Anterior pituitary

Using forceps, the dura matter that covers gland was removed leaving the organ free on the sella turcica. The neural lobe was carefully separated from the anterior pituitary (AP) which was then carefully lifted with fine curved tip forceps pointing upwards. It was rapidly weighed, then put in a tube and placed momentarily in liquid nitrogen.

Ovaries

The genital apparatus was dissected by cutting the mesentery to isolate the uterine horns, the tubular oviduct, the ovaries and the junction between the anus/rectum and the vulva/vagina, leaving the unit of the sexual organs and the urinary bladder isolated. The ovaries were carefully separated from the oviducts; the fat around the ovaries was also removed. Both gonads were placed in a single Eppendorf tube and momentarily placed in liquid nitrogen.

Liver

Liver tissue extraction was made by cutting a piece of the median lobe (0.5 cm x 0.5 cm). Tissue was placed in a tube and momentarily stored immersed in liquid nitrogen.

Adipose tissue

Adipose tissue samples were obtained from the fatty tissue of the small intestine.

Tail skin

For skin tissue, 5 cm of a distal tail portion were cut with scissors. Skin was separated and hair removed using scalpel. Tissue was placed in a tube and stored as described for other tissues.

DNA was extracted from blood on an automated nucleic acid extraction platform called QiaSymphony (Qiagen) with a magnetic bead-based extraction kit, QIAsymphony DNA

Midi Kit (Qiagen). DNA was extracted from tissue on an automated nucleic acid extraction platform called Anaprep (Biochain) with a magnetic bead-based extraction kit, Tissue DNA Extraction Kit (Biochain). DNA from brain regions was extracted using an automated nucleic acid extraction platform called QIAcube HT (Qiagen) with a column-based extraction kit, QIAamp 96 DNA QIAcube HT Kit (Qiagen).

M17. Dog [61]

For this study, DNA samples were collected from a total of 742 blood samples taken from dogs across 93 different breeds. These samples were generously provided by researchers at the National Human Genome Research Institute (NHGRI). Unfortunately, we did not have access to individual weight data for these dogs.

Ethics

The collection of these samples was conducted in compliance with ethical guidelines and was officially approved by the Animal Care and Use Committee of the Intramural Program of NHGRI at the National Institutes of Health (Protocol #8329254).

Dog lifespan and breed characteristics

Standard breed weight (SBW), height (SBH) and lifespan were aggregated from several sources. SBW and SBH were taken from previously reported values [62,63], which were updated if AKC values differed [64]. If the AKC did not specify SBW or SBH, we used data from Atlas of Dog Breeds of the World [65]. Lifespan estimates were calculated as the average of the standard breed across sexes, compiled from numerous publications consisting primarily of multi-breed surveys of age and cause of death from veterinary clinics and large-scale breed-specific surveys, which are often conducted by purebred dog associations. When available, data were combined across surveys for number of dogs, minimum, maximum, mean, and median age at death. The minimums, maximums,

and medians were averaged across studies to produce a representative lifespan expectation for each breed. For three breeds (American Hairless Terrier, Sloughi, and Ibizan Hound), no published survey data were available. For these breeds, the maximum age expectation was obtained from the American Kennel Club website.

M18. Bats [66]

<u>Ethics</u>

The bat study[66] incorporated several protocols as detailed below:

(i) Procedures involving live animals were in accordance with the guidelines from the American Society of Mammalogists and approved by the University of Maryland Institutional Animal Care and Use Committee (protocols R-05-26, R-08-39, R-10–63, and FR-APR-18-16).

(ii) Other parts of the study received approval from the NEOMED Institutional Animal Care and Use Committee or the Animal Research Ethics Board of McMaster University (AUP# 20-05-20).

(iii) Animal handling methods followed guidelines set by the American Society of Mammalogists and were approved by the University of Maryland Institutional Animal Care and Use Committee (protocols R-91-33, R-93-22, R-94-25, R-01-07, R-11-21, R-13-77, and FR-APR-18-16). These methods were conducted under licenses from the Forestry Division of the Ministry of Agriculture, Land and Fisheries, Trinidad and Tobago.

(iv) Our research in Panama received approval from the Smithsonian Tropical Research Institute and its Animal Care and Use Committee (ACUC, permits: IACUC 100316-0910-12, ACUC 2013-1015-2016).

(v) Additional Details on Animal Capture and Handling in Specific Protocols:

The capture and handling of animals in this study were governed by several permits. In Panama, operations received authorization under permits SE/A-112-13, SE/A-73-14, SE/A-95-15, and SE/A-32-17 from the Autoridad Nacional del Ambiente. We also obtained approval from the Institutional Animal Care and Use Committee of the Smithsonian Tropical Research Institute (2012-0505-2015).

In addition, our animal handling practices adhered to current Costa Rican laws and received approval from the Animal Care Review Committee of SINAC (Sistema Nacional de Áreas de Conservación) and MINAE. The applicable permits included: 272-2003-OFAU, 135-2004-OFAU, 022-2005-OFAU, 108-2006-SINAC, 147-2007-SINAC, 183-2008-SINAC, 187-2009-SINAC, 130-2010-SINAC, 068-2011-SINAC, 115–2012-SINAC, 033–2013-SINAC, SINAC-SE-GASP-PI-R-121–2013, R-006–2015-OT-CONAGEBIO, and SINAC-SE-CUS-PI-R-088–2016.

Wing tissue samples

Wing punches were taken from 778 individually marked animals that were either kept in captivity (15 species) or recaptured as part of long-term field studies (11 species). We excluded 42 samples because we did not have independent evidence to confirm minimum age estimates. For 630 samples the individual was marked shortly after birth, so age estimates were exact. For the remainder, age represented a minimum estimate because the individual was not initially banded as a juvenile. We used minimum age estimates when other evidence, such as tooth wear or time since initial capture, indicated that the minimum age estimate was likely to be close to the real age.

Here, we provide additional information on when and where samples were taken from either captive or free- ranging animals for each of the 26 species of bats used in this study.

Pallid bats, *Antrozous pallidus,* were captured between 2005 and 2008 at six sites in central Oregon (44.94° N, 120.38° W) using mist nets over a water source or outside a night roost or with a handnet on an extension pole outside a day-roosting crevice. Each bat was weighed, measured, and marked with a numbered band. Adults were distinguished from juveniles by closed epiphyseal gaps. Tissue samples were obtained from wing membranes using 3 mm biopsy punches and stored in 95% ethanol until DNA was extracted using a Qiagen DNeasy Tissue Kit. DNA extracts were stored frozen at -80°C. Live animal procedures conformed to the American Society of Mammalogists guidelines and were approved by the University of Maryland Institutional Animal Care and Use Committee (protocols R-05-26 and R-08-39). Bat capture and sampling was conducted with permission of the Pine Creek Conservation Area, the Oregon Department of Fish and Wildlife (permit 081-95), and the John Day Fossil Beds National Monument, National Park Service (permit JODA-2005-SCI-0003).

Wing tissue from Artibeus jamaicensis, Cynopterus brachyotis, Eidolon helvum, Pteropus giganteus, P. hypomelanus, P. poliocephalus, P. pumilus, P. rodricensis, P. vampyrus, and Rousettus aegyptiacus was taken between 2006 and 2017 from bats kept at the Lubee Bat Conservancy, an AZA (Associated Zoos and Aquariums [https://www.aza.org/current-cert]) certified facility, in Gainesville, Florida. The bats are group-housed in twelve 1068 sq. ft. enclosures with indoor temperature-controlled roosting areas and outdoor flight rooms and are fed a diet of fruit, vegetables and

nutritional supplements. Wing tissue biopsies are periodically taken from individually marked animals and kept at -20°C in 95% ethanol. The majority of 243 samples from these species were taken from animals that were born in captivity. DNA was extracted with a Zymo miniprep plus kit.

Wing tissue samples were taken in 2018 from captive Carollia perspicillata housed in a tropical zoo (Papiliorama, Kerzers FR, Switzerland). Approximately 400 bats roost in an artificial cave kept on a reversed light cycle and are fed twice a night with a fruit-based diet. Since 2011, the population has been monitored by capturing individuals using a harp-trap placed at the entrance to the cave. Forearm length, body weight, reproductive status and tooth-wear are recorded from every captured individual. At first capture, individuals are marked on the forearms with a unique combination of three colored plastic rings (A.C. Hughes, UK, size XB). All captures and marking were authorized by the cantonal veterinary service (permits nb:2011_42_FR, 2013_10E_FR, 2014_59_FR). Between July and November 2018, 3mm biopsies were punched on the patagium and hermetically stored in silica gel. Based on the date of first capture and tooth-wear score, the age of each individual sampled was estimated. DNA was extracted with a Zymo miniprep plus kit.

Biopsy punches (2 or 3 mm) were taken from the wing of captive common vampire bats, *Desmodus rotundus,* between 2010 and 2014. Bats were housed and fed blood in flight cages (3 x 2 x 1.5 m) as a captive group at the Cranbrook Institute of Science (24-39 bats, Bloomfield Hills, MI, USA) or at the University of Maryland (7 bats, University of Maryland Institutional Animal Care and Use Committee protocol R-10–63). Age was determined based on zoo birth records. Individuals were born at the Houston Zoo,

Cincinnati Zoo, Chicago Brookfield Zoo, or the Cranbrook Institute of Science. Tissue samples were stored in 95% ethanol prior to DNA extraction using a Qiagen DNeasy kit. DNA extracts were frozen for long-term storage at -80°C. Live animal procedures conformed to the American Society of Mammalogists guidelines and were approved by the University of Maryland Institutional Animal Care and Use Committee (protocol R-10-63).

Wing tissues of big brown bats (*Eptesicus fuscus*) were sampled with a 3 mm biopsy punch from the wing of known age captive bats at Northeast Ohio Medical University (NEOMED; Rootstown, Ohio) in 2018 and 2019. These animals came from a colony previously maintained by Dr. Ellen Covey at the University of Washington, which was started in 2005 with bats caught in North Carolina that were banded according to year of capture or birth. These bats underwent natural hibernation and were exclusively fed an *ab libitum* diet of fresh water and mealworms (*Tenebrio molitor*). In 2014 some of the bats were transported to NEOMED and are now housed indoors on a 12 h light/dark cycle and fed the same *ab libitum* fresh water and mealworm diet. Wing punches were taken from a second colony of *E. fuscus* by Dr. Paul Faure and Lucas Greville in February or August 2020. This colony is kept at McMaster University in Hamilton, Ontario, Canada in an indoor/outdoor enclosure (2.5 x 8.3 x 2.7m) in which the temperature fluctuates with ambient conditions but is kept above freezing by a heater on a thermostat. Known-age animals from this colony were born in captivity from females captured in Ontario. Tissue samples from both colonies were stored in DNA Shield and kept frozen at -20°C prior to DNA extraction with a Zymo miniprep plus kit. Animal use protocols were approved by

the NEOMED Institutional Animal Care and Use Committee or the Animal Research Ethics Board of McMaster University (AUP# 20-05-20).

During July 2019, wing membrane samples were obtained from subadult or adult female and subadult male lesser long-nosed bats, *Leptonycteris yerbabuenae,* with a 4 mm biopsy punch at the entrance of the Pinacate Cave in the Reserva de la Biosfera el Pinacate y Gran Desierto de Altar (31°38'51.6" N, 113°28'53.5" W), Sonora, Mexico. Bats were captured using mist nets (Avinet models: TB02, TB06, TB012; Portland, Maine, USA) set outside caves just prior to when bats emerged to forage. Individuals were sexed, weighed and the forearm measured. To discriminate subadults from adults, age was determined by the degree of fusion of the epiphyses at the metacarpal–phalangeal joint. Tissue samples were stored in DNA/RNA Shield buffer (Zymo Scientific, Irvine, CA 92614, U.S.A.). DNA was extracted with a Zymo miniprep plus kit. Bat tissue samples were collected under permit SGPA/DGVS/06361/17 issued to R. A. Medellín by The Ministry of Environment and Natural Resources.

Samples of velvet free-tailed bats, *Molossus molossus*, come from a long-term study in Gamboa, Panama (09°07’ N 79°41’ W), where the bats roost in crevices in houses. We captured social groups with mist nets (Ecotone, Gydnia, Poland) at the entrance of roosts during evening emergence and individually marked all bats with a subcutaneous passive integrated transponder (Trovan ID-100, Euro ID, Weilerswist, Germany) at first capture. Wing tissue samples were taken with a 3 mm biopsy punch and stored in 96% ethanol until DNA extraction using a Zymo miniprep kit. Capture and handling of animals were carried out under permits SE/A-112-13, SE/A-73-14, SE/A-95-15, and SE/A-32-17 from the Autoridad Nacional del Ambiente in Panama with approval from the Institutional

Animal Care and Use Committee of the Smithsonian Tropical Research Institute (2012-0505-2015)

Little brown bats, *Myotis lucifugus*, were captured as they departed from an attic maternity colony in Chestertown, Maryland (39°12'N, 76°04'W), in September 1996. Captured bats were weighed, measured and banded with individually marked bands. Young of the year were identified by their weight and absence of tooth wear. Wing membrane biopsies were taken and stored in a 5M NaCl with 20% dimethyl sulfoxide solution and kept frozen at -80°C. DNA was extracted with a Zymo miniprep plus kit. Bat capture and handling was approved by the Maryland Department of Natural Resources (permit SCO-30403).

Wing tissue samples were taken from greater mouse-eared bats, *Myotis myotis*, between 2013 and 2018 as part of a long-term mark-recapture study conducted by Bretagne Vivante in Brittany, France (47°35'N, 2°14'W"). Bats were caught using modified harp traps as they left one of five different roosts. Individuals at first capture are fitted with PIT tags to facilitate identification on subsequent recaptures. Measurements taken from each animal include sex, forearm length, weight, and transponder number. Age class (juvenile or adult) was determined by examining the degree of the epiphyseal closure of the metacarpal-phalangeal joints. Wing biopsies were taken with a 3 mm biopsy punch, flash frozen and stored in liquid nitrogen prior to extraction. All procedures were conducted with full ethical approval and permission (AREC-13- 38-Teeling) awarded by the University College Dublin ethics committee and in accordance with permits issued by 'Arrêté' by the Préfet du Morbihan. DNA was extracted from wing biopsies using a Promega Wizard SV DNA extraction kit (catalog no. A2371) or the Qiagen DNeasy Blood and Tissue kit

(Qiagen). Extractions carried out with the Promega kit were partially automated using a Hamilton STAR Deck liquid handling robot.

Adult and juvenile Mexican fishing bats, *Myotis vivesi*, were captured by gloved hand from roosts in talus slopes on Isla Partida Norte in the Gulf of California, Mexico (29°03'N, 113°00'W) during the day between 2015 and 2018. Individuals were measured and banded with numbered metal bands on their forearms for identification upon recapture. In 2018 wing tissue was taken with a 3 mm biopsy punch and preserved in Zymo DNA shield. Bat capture and handling were conducted under permits #7668–15, 2492–17 and #5409-18 from Dirección General de Vida Silvestre, and permits #17–16, 21–17 and 20-18 from Secretaría de Gobernación, and the University of Maryland Institutional Animal Care and Use Committee protocols FR-15-10 and FR-18-20.

Common noctules, Nyctalus noctula, were captured as part of a long-term study at the Seeburgpark in Kreuzlingen, Switzerland (47.649928° N, 9.186123° E) where bats regularly roost in boxes. Each bat was marked with a subcutaneous pit-tag (ID100; Euro ID, Weilerswist, Germany) injected under the dorsal skin. Wing tissue samples were taken with a 3 mm biopsy punch and stored in 96% ethanol until DNA extraction using a Zymo miniprep kit. All handling and sampling of the bats in Switzerland was approved by the Veterinäramt Thurgau (permit FIBL1/12).

Wing tissue samples were taken in September 2018 from lesser spear-nosed bats, *Phyllostomus discolor*, kept in a breeding colony in the Department Biology II of the Ludwig- Maximilians-University in Munich. In this colony animals were kept under semi-natural conditions (12 h day/night cycle, 65 to 70 % relative humidity, 28°C) with free access to food and water. The license to keep and breed *P. discolor* was issued by the

German Regierung von Oberbayern. Under German Law on Animal Protection a special ethical approval is not needed for wing tissue collection. Wing tissue was stored in RNAlater until DNA was extracted using QIAamp® MinElute columns following the manufacturer's instructions. The samples were eluted in 50µl of molecular grade water and concentrated to reduce their volume by approximately 50% using a Speedvac, with the following settings: duration 20 minutes, temperature in the chamber 30°C, H2O (water) mode. Greater spear-nosed bats, Phyllostomus hastatus, were captured and sampled between 1990 and 2018 in Trinidad, Lesser Antilles. Most often, harem groups, which include one adult male plus 15-20 lactating females with pups, were captured during the day from within a solution depression in the ceiling of either Tamana (10.4711°N, 61.1958°W), Caura (10.7019°N, 61.3614°W), or Guanapo cave (10.6942°N, 61.2654°W) using a bucket trap. Captured bats were sexed, measured for size, weight, and tooth wear, and individually marked with stainless steel numbered bands. Age was determined exactly for adults that were recaptured after being banded as pups. Wing biopsy punches (4 mm) were stored frozen at -80°C in either a 5M NaCl with 20% dimethyl sulfoxide solution or Zymo DNA Shield prior to DNA extraction using a Qiagen Puregene or Zymo miniprep plus kit. Frozen samples were selected to maximize the number of known-age individuals with approximately equal numbers at all ages. Animal handling methods follow guidelines by the American Society of Mammalogists and were approved by the University of Maryland Institutional Animal Care and Use Committee (protocols R-91-33, R-93-22, R-94-25, R-01-07, R-11-21, R-13-77, FR-APR-18-16) under licenses from the Forestry Division of the Ministry of Agriculture, Land and Fisheries, Trinidad and Tobago.

Wing tissue samples were taken from greater horseshoe bats, *Rhinolophus ferrumequinum*, by using 3 mm biopsy punches between 2016 and 2018 from wild female bats as part of a long-term study at a maternity colony in Gloucestershire, UK (51.7107°N, 2.2777°W). Bats were captured at the roost with hand nets, and all individuals were weighed, ringed with aluminum alloy rings and morphometric data such as forearm length recorded under licenses (Natural England Project Licenses 2015-9918-SCI-SCI; 2016-23583- SCI-SCI; 2017-30137-SCI-SCI) issued to Roger Ransome. All bats studied were first marked as infants, so we could be certain of their age. Bats were aged between 1-21 years, with the 40 individuals selected in a fairly even manner across this age span. Sampling procedures were conducted under licenses (Natural England 2015-11974-SCI-SCI; 2016-25216-SCI-SCI; 2017- 31148-SCI-SCI) issued to Gareth Jones, with tissue biopsy additionally licensed under Home Office Project Licenses (PPL 30/3025 prior to 2018; P307F1428 from 2018 onwards) and Home.

Tissue samples were stored in silica gel beads and then transferred to a -20C freezer for long-term storage. DNA was extracted from wing biopsies using a Promega Wizard SV DNA extraction kit or a Qiagen DNeasy Blood and Tissue kit. Extractions carried out with the Promega kit were partially automated using a Hamilton STAR Deck liquid handling robot but otherwise followed manufacturer’s instructions.

Samples of proboscis bats, *Rhynchonycteris naso,* came from a long-term study between 2005 and 2016 at La Selva Biological Station in Costa Rica (10° 25´ N, 84° 00´W). Bats were mist-netted in the vicinity of their roosts. Wing tissue was sampled with a 4 mm biopsy punch, individuals were marked with colored plastic bands, sexed, measured and age class determined (juvenile: 0-4 months, subadult: 5-10 months, or adult>10 months).

Age was determined exactly for individuals that were banded as pups and recaptured as adults. Ethanol (80%) was used to preserve tissue samples, and a salt–chloroform procedure or Qiagen BioSprint 96 DNA Blood Kit was used for DNA isolation. Research permits were granted by the MINAE (Ministerio del Ambiente y Energia) and the ACC (Área de Conservación Central). Animal treatment followed the Guide for Care and Use of Laboratory Animals of the National Institutes of Health. Animal handling complied with current Costa Rican laws and was approved by the Animal Care Review Committee of the SINAC (Sistema Nacional de Áreas de Conservación) and MINAE (permits 022-2005-OFAU, 108-2006-SINAC, 147-2007-SINAC, 183-2008-SINAC, 187-2009-SINAC, 130-2010-SINAC and 068-2011-SINAC, 115–2012- SINAC, 033–2013-SINAC, SINAC-SE-GASP-PI-R-121–2013, R-006–2015-OT-CONAGEBIO, SINAC-SE-CUS-PI-R-088–2016).

Samples of greater sac-winged bats, *Saccopteryx bilineata* came from long-term studies in Costa Rica (n = 21 from La Selva Biological Station, 10° 25´ N, 84° 00´W and n = 6 from Santa Rosa National Park, 10° 53′ N, 85° 46′ W) and Panama (n = 4 from the Biological Station Barro Colorado Island (BCI) of the Smithsonian Tropical Research Institute, 9° 9′ N/79° 51′ W) between 1994 and 2016. Bats were captured with mist nets when entering or leaving their day roosts, individually banded with two coloured plastic bands on their forearms, and a wing tissue biopsy sample (4mm) preserved in 80% ethanol was taken. Age was determined exactly for individuals that were banded as pups and recaptured as adults. DNA was extracted with a salt-chloroform procedure or with the Qiagen BioSprint 96 DNA Blood Kit. The process of acquiring data and protocols for capturing and handling bats complied with the current laws of Panama and were

conducted in accordance with the relevant guidelines and regulations. Our study in Panama was approved by the Smithsonian Tropical Research Institute and its Animal Care and Use Committee (ACUC, permits: IACUC 100316-0910-12, ACUC 2013-1015-2016). For research in Costa Rica, permits were granted by the MINAE (Ministerio del Ambiente y Energia), the ACC (Área de Conservación Central) and the ACG (Área de Conservación Guanacaste). Animal treatment followed the Guide for Care and Use of Laboratory Animals of the National Institutes of Health. Animal handling complied with current Costa Rican laws and was approved by the Animal Care Review Committee of the SINAC (Sistema Nacional de Áreas de Conservación) and MINAE (permits 272-2003-OFAU, 135-2004-OFAU, 022-2005-OFAU, 108-2006-SINAC, 147-2007-SINAC, 183-2008-SINAC, 187-2009-SINAC, 130-2010-SINAC and 068-2011-SINAC, 115–2012-SINAC, 033–2013-SINAC, SINAC-SE-GASP-PI-R-121– 2013, R-006–2015-OT-CONAGEBIO, SINAC-SE-CUS-PI-R-088–2016).

Mexican free-tailed bats, *Tadarida brasiliensis*, are housed at Bat World Sanctuary, a licensed non-profit bat rehabilitation facility and accredited by the Global Federation of Animal Sanctuaries (https://www.sanctuaryfederation.org/sanctuaries/bat-world/) in Weatherford, Texas. Most individuals sampled were rescued as pups, although some were rescued as adults, making their exact age unknown. Individuals are group-housed in large indoor enclosures. Wing membrane biopsies (4 mm) were collected by Amanda Lollar in August 2019 and stored in Zymo DNA Shield until DNA was extracted using a Zymo miniprep plus kit.

M19. Cattle [67]

Ethics

All animal procedures were carried out in accordance with the relevant guidelines at each institution. Specifically, procedures related to sample collection in Poland followed the EU Directive of the European Parliament and the Council on the protection of animals used for scientific purposes (22 September 2010; No 2010/63/EU), Polish Parliament Act on Animal Protection (21 August 1997, Dz.U. 1997 nr 111 poz. 724) with further novelization - Polish Parliament Act on the protection of animals used for scientific or educational purposes (15 January 2015, Dz.U. 2015 poz. 266). Blood and oocyte collection were approved by the Local Ethics Committee for Experiments on Animals, University of Warmia and Mazury in Olsztyn, Poland (Agreement No. LKE.065.27.2019). For animal procedures in the USA, approval from the University of Nebraska Institutional Animal Care and Use Committee was obtained (approval number is 1560).

Samples

For this study, we obtained samples from 357 female cattle (Bos Taurus). These animals were housed at two locations: the Dairy Farm of the Institute of Animal Reproduction and Food Research of the Polish Academy of Sciences, located in Wielki Las, Poland, and the Eastern Nebraska Research and Extension Center at the University of Nebraska-Lincoln in Nebraska, USA. We collected samples from a Nebraska herd consisting of Black Angus and composites of varying percentages of Simmental x Angus (Black) or Red Angus. All these animals were certified free from Bovine Herpesvirus Type 1, Bovine Viral Diarrhea/Mucosal Disease, Tuberculosis, and Enzootic Bovine Leucosis. Additionally, we secured 95 skin samples from male cattle for this study.

Blood collection and further processing

The blood samples from both herds were collected during routine animal management activities, during the routine blood collection for disease prevention. Blood samples were taken only from cows in the luteal phase of the estrous cycle. Blood was collected into 8ml PAXgene Blood DNA Tubes (Quiagen, Cat No. 761115) and stored at -80°C until the shipment (USA Veterinary Permission Nr 138809) to the UCLA Technology Center for Genomics & Bioinformatics (Los Angeles, USA) for further analyses.

Oocyte Collection and further processing

In total 80 Bovine ovaries were collected immediately *postmortem* from 40 cows which were selected for routine culling due to management reasons. All cows were in the luteal phase of the estrous cycle. Before the isolation of both ovaries from each cow, blood samples were also collected into 8ml PAXgene Blood DNA Tubes (Quiagen, Cat No. 761115) to generate both sample types from one donor. Isolated ovaries were kept on ice and immediately transported to the laboratory. Afterwards, immature bovine cumulus–oocyte complexes (COCs) were recovered by aspirating ovarian follicles in the diameter of 2–8 mm. Cumulus cells were removed from COCs by pipetting them for 5min in a Petri dish containing 500 µl of Phosphate Buffered Saline (PBS) with 0.1% hyaluronidase (Sigma-Aldrich, St. Louis, MO, USA). Denuded oocytes from every donor were pooled (10-15 immature oocytes/ovary) and then processed for genomic DNA isolation.

M20. Mouse data [68]

The mouse data were sourced from various institutions or studies, as outlined below: (i) UCLA Lab. Animal breeding and husbandry [69], (ii) BXD mice [68] from University of Tennessee Health Science Center, (iii) Growth hormone receptor knockout from the University of Michigan, (iv) Calorie restricted mice from the University of Texas

Southwestern Medical Center [70], (v) South African species, (vi) Apodemus mice[71] , and (vii) Spiny mouse.

Ethics for (i)

All mice were maintained and bred under standard conditions consistent with National Institutes of Health guidelines and approved by the University of California, Los Angeles Institutional Animal Care and Use Committees.

Ethics for (ii)

All animal procedures were in accordance with the protocol approved by the Institutional Animal Care and Use Committee (IACUC) at the University of Tennessee Health Science Center.

Ethics for (iv)

The Institutional Animal Care and Use Committee (IACUC) of the University of Texas Southwestern Medical Center approved the animal protocol (APN 2015-100925), which has been subsequently renewed every 3 years (2018 and 2021).

Ethics for (v)

The Animal Use and Care Committee of the University of Pretoria evaluated and approved the experimental protocol and collection of all samples (ethics clearance number: NAS022/2021, NAS209/2021, NAS021/2020), with DAFF section 20 approval (SDAH-Epi-21051907211, SDAH-Epi-12/11/1/1/8 (2002 LH), SDAH-Epi-20072707050). In addition, permission to capture the various species was obtained from all landowners, and a collecting permit was obtained from the relevant nature conservation authorities (Permit number: Western Cape- CN44-87-13780, CN44-31-2285, Gauteng- CPF6-0124,

Kwa-Zulu Natal- OP1545/2021). Necessary TOPS permits were also acquired for threatened species (Permit number: 68103).

Ethics for (vi)

All animal work was conducted in accordance with the UK Home Office in compliance with the Animals (Scientific Procedures) Act 1986, was approved by the University of Edinburgh Ethical Review Committee and was carried out under the approved UK Home Office Project License PP4913586.

Ethics for (vii)

Animal protocols were approved by the Institutional Animal Care and Use Committee (IACUC) at the University of Kentucky (2019-3254).

UCLA Lab. Animal breeding and husbandry [69]

All mice were maintained and bred under standard conditions consistent with National Institutes of Health guidelines and approved by the University of California, Los Angeles Institutional Animal Care and Use Committees. The cages were maintained on a 12:12 light/dark cycle, with food and water ad lib. Tissues were harvested from discervical mice and fresh frozen on dry ice.

The Tet3 knockout mice were generated at the Jackson Laboratory using CRISPR technology with deletion of exon 4. Tet3 heterozygous knockout mice were crossed with C57BL/6J (JAX 000664) to set up the colony. All mice were maintained and bred under standard conditions consistent with National Institutes of Health guidelines and approved by the University of California, Los Angeles Institutional Animal Care and Use Committees. The cages were maintained on a 12:12 light/dark cycle, with food and water

ad lib. Mice were euthanized by cervical dislocation, cortical and striatal tissues were then quickly dissected out and fresh frozen on dry ice until used for DNA extraction.

Bcl11b heterozygous mouse was obtained from MMRRC at University of California, Davis (Catalog # 046780-UCD) and crossed with C57BL/6J (JAX 000664) to set up the colony. All mice were maintained and bred under standard conditions consistent with National Institutes of Health guidelines and approved by the University of California, Los Angeles Institutional Animal Care and Use Committees. The cages were maintained on a 12:12 light/dark cycle, with food and water ad lib. Mice were euthanized by cervical dislocation, cortical and tissues were then quickly dissected out and fresh frozen on dry ice until used for DNA extraction.

BXD mice [68] from University of Tennessee Health Science Center

Samples for this study were selected from a larger colony of BXD mice that were housed in a specific pathogen-free (SPF) facility at the University of Tennessee Health Science Center (UTHSC). All animal procedures were in accordance with the protocol approved by the Institutional Animal Care and Use Committee (IACUC) at the University of Tennessee Health Science Center. Detailed description of housing conditions and diet can be found in (Roy, 2020; Williams, 2020). Mice were given ad libitum access to water, and either standard laboratory chow (Harlan Teklad; 2018, 18.6% protein, 6.2% fat, 75.2% carbohydrates), or high-fat chow (Harlan Teklad 06414; 18.4% protein, 60.3% fat, 21.3% carbohydrates). Animals were first weighed within the first few days of assignment to either diet, and this was mostly but not always prior to introduction to HFD. Following this, animals were weighed periodically, and a final time (BWF) when animals were humanely euthanized (anesthetized with avertin at 0.02 ml per g of weight, followed by

perfusion with phosphate-buffered saline) at specific ages for tissue collection. The present work utilizes the biobanked liver specimens that were pulverized and stored in -80 °C, and overlaps samples described in (Williams, 2020).

Growth hormone receptor knockout from the University of Michigan

Growth hormone receptor knockout mice (GHR -/- dwarfs) were generated from the University of Michigan (Richard A. Miller). Details about the full-body growth hormone receptor knockout (GHR-KO) were outlined in a previous study[72].

Calorie restricted mice from the University of Texas Southwestern Medical Center [70]

The Institutional Animal Care and Use Committee (IACUC) of the University of Texas Southwestern Medical Center approved the animal protocol (APN 2015-100925), which has been subsequently renewed every 3 years (2018 and 2021).

Animals

C57BL/6J male mice (6 week-old, n = 72) were obtained from the Mouse Breeding Core, Wakeland lab, UT Southwestern Medical Center, Dallas, TX, USA. After 2 weeks of acclimation, mice were individually housed in standard polycarbonate mouse cages (Fisher Scientific, Cat. Nos. 01-288-1B and 01-288-21) with stainless steel running wheel, inside isolation cabinets containing 12 cages each. Temperature and humidity levels were monitored, and the mice were housed under light/dark cycles of LD12:12 h (green LEDs, ~100 lux at the level of the cage floor). Water was provided ad libitum throughout the study.

Mice were fed with round pellets of 300 mg each containing 3.60 Kcal/g (Dustless Precision Pelletsâ, Rodent, Purified, F0075, BioServ, Flemington, NJ, USA). The food

composition is similar to regular mouse chow (18.7% protein, 5.6% fat, 59.1% carbohydrates and 4.7% fiber). Food access was controlled by an automated feeder system designed in our lab with Phenome Technologies Inc., Skokie, IL, USA that precisely controls how much, when, and how often the food is dispensed (Acosta-Rodríguez et al., Cell Metabolism 2017). After six weeks of recording under ad libitum food access, 12 mice/group were randomly assigned to one of 6 feeding conditions (Acosta-Rodriguez et al., under revision): 24h access ad libitum; 70% of baseline ad libitum levels fed at the beginning of the dark (CR-night) or light (CR-day) phase which they consumed in less than 2h (classic CR protocols); or fed under CR evenly spread over 12 hours during the dark (CR-night-12h) or light (CR-day-12h) by releasing one 300 mg pellet every 90 min; or evenly spread over 24 hours (CR-spread) by releasing one 300 mg pellet every 160 min. Mice were weighed during cage change every 21 days, and bedding was checked for any evidence of food spillage. ClockLab Chamber Control Software v3.401 (Actimetrics Inc., Wilmette, IL, USA) was used to schedule and record feeding events, and ClockLab Data Acquisition System v3.209 (Actimetrics Inc., Wilmette, IL, USA) was used to record wheel-running behavior.

Tissue collection and DNA extraction

At 19 months of age, mice were released into constant darkness and samples collected every 4 hours for 48 hours (n=12 mice/condition). Tissues were snap frozen and stored at -80°C until DNA extraction.

Liver tissue was digested using 1mL of digestion buffer (100mM NaCl, 50mM Tris-HCl pH 8, 100mM EDTA pH 8 and 1% SDS) plus 20uL of protein K (20mg/mL) incubated ON at 60ºC. After a RNAse (Ambion, AM2286, 2uL) treatment for 1h at 37ºC, DNA extraction

was performed using UltraPure™ Phenol:Chloroform:Isoamyl Alcohol (25:24:1, v/v, Thermo Fisher, #15593049) and phase lock gel (VWR, # 10847-802) to avoid contamination with interphase, according to manufacturer instructions.

South African species

All biological samples from South Africa were either historical samples (collected prior to the formation of the Animal Use and Care Committee of the University of Pretoria), archived post-mortem samples or collected fresh from animals prior to this study in origin. All samples were stored at -80ºC.

The Animal Use and Care Committee of the University of Pretoria evaluated and approved the experimental protocol and collection of all samples (ethics clearance number: NAS022/2021, NAS209/2021, NAS021/2020), with DAFF section 20 approval (SDAH-Epi-21051907211, SDAH-Epi-12/11/1/1/8 (2002 LH), SDAH-Epi-20072707050). In addition, permission to capture the various species was obtained from all landowners, and a collecting permit was obtained from the relevant nature conservation authorities (Permit number: Western Cape- CN44-87-13780, CN44-31-2285, Gauteng- CPF6-0124, Kwa-Zulu Natal- OP1545/2021). Necessary TOPS permits were also acquired for threatened species (Permit number: 68103).

Study animals

Except for *Heterocephalus glaber, Fukomys damarensis, Atelerix albiventris* and *Echinops telfairi*, which came from captive-raised populations, all other small mammal species were wild-caught in South Africa. While all small subterranean mammals, *Georychus capensis, Bathyergus suillus, Cryptomys hottentotus hottentotus, C. h. pretoriae, C. h. mahali, C. h. natalenesis* and *Amblysomus hottentotus* were captured

using Hickman live traps, baited with a small piece of sweet potato. All traps were monitored for captures every 2-3 hours over the course of the day and left overnight, being checked first thing in the morning.

Apodemus mice[71]

Apodemus mice were culled during routine (yearly) population control, via cervical dislocation confirmed by exsanguination. All animal work was conducted in accordance with the UK Home Office in compliance with the Animals (Scientific Procedures) Act 1986, was approved by the University of Edinburgh Ethical Review Committee and was carried out under the approved UK Home Office Project License PP4913586.

Prof Thomas Little maintains a formerly-wild, but now lab-reared wood mouse colony in standard laboratory conditions at the University of Edinburgh. The colony has been in captivity for many generations, but the wood mice are purposely outbred to maintain genetic diversity. All mice are housed individually in ventilated cages (Techniplast, 1285L) with food and water ad libitum. DNA was extracted using a phenol-chloroform method from ear punches taken from individuals of known age from the colony. We obtained DNA from 48 mice of both sexes, spanning an age range of 88 to 496 days old. This slightly unusual choice of ages arises because we utilised mice that were part of other experiments. Lifespan in wild mice is not known for certain, and many will die from extrinsic mortality (e.g., predation), but in our own fieldwork we recapture 10-20% of tagged mice the following year (Pedersen, unpublished data), meaning wild mice may can live for hundreds of days.

Spiny mouse

Following anesthesia with 4% (v/v) vaporized isoflurane (Henry Schein Animal Health, Dublin, OH), full thickness ear tissue biopsies were collected from spiny mice (Acomys cahirinus) housed in breeding colonies at the University of Kentucky, Lexington, KY or Monash University, Melbourne, Australia. Biopsies were used for ear tissue samples and to produce ear pinna fibroblast cultures. Animal protocols were approved by the Institutional Animal Care and Use Committee (IACUC) at the University of Kentucky (2019-3254). Spiny mouse fetal fibroblasts were derived from embryonic skin following enzymatic digestion with trypsin. Connective tissue fibroblasts from adult spiny mice were derived from ear biopsies and grown at 37 °C in 3% oxygen and 5% CO2 as previously described (Saxena et al., 2019).

M21. Nova Scotia masked shrews (*Sorex cinereus*) [73]

Shrews and small animals from Museum of Biological Diversity at The Ohio State University

Ethics

All wild-caught animals were collected and sacrificed in accordance with protocols approved by The Ohio State University IACUC (Institutional Animal Care and Use Committee) under protocol number 2017A00000036. All wild-caught animals were collected with scientific collecting permits issued from Ohio and Washington and according to guidelines established by the American Society of Mammalogy for the use of wild animals in research (Sikes & Animal Care and Use Committee of the American Society of Mammalogists 2016).

Samples

Tissue samples were taken from archived specimens that are in the process of being deposited in the Museum of Biological Diversity at The Ohio State University.

Tissue samples were acquired from wild animals using multiple collecting procedures, including (1) trapping live animals using Sherman live-traps or pitfalls and followed by euthanasia using isoflurane overdose, (2) sampling of roadkill animals, and (3) sampling of deceased animals received from wildlife rehabilitation centers. Abdominal and pelvic organs were removed from animals and archived in a -80C freezer.

Masked shew samples

Masked shrew samples were collected from five locations: Peterborough County, Ontario and Sandy Cove, North Mountain, Long Island and Bon Portage Island (BPI) in Nova Scotia, Canada. All shrews were trapped within six weeks of each other to limit seasonal morphological changes, such as skull and body size (Lázaro et al., 2019, 2021). DNA was extracted from liver, tail, and fetus using the DNeasy Blood & Tissue Kit from QIAGEN. Shrews were left in dermestid beetle tanks for a week to remove all remaining tissue from the skulls. A Leica EZ4 microscope was used to assess age class for each shrew based on teeth wear according to Pruitt's method (Pruitt, 1954; Rudd, 1955). Age in months was estimated based on the trapping date, the age class, and the known reproductive season of masked shrews assuming a maximum lifespan of ~17 months (Churchfield, 1990). Shrews were sexed by PCR amplification of the SRY gene (Cervantes et al., 2013; Matsubara et al., 2001) using custom primers. Gel

electrophoresis was used to identify successful amplification and thus confirm the presence of the Y chromosome (i.e. male). Age class designations were confirmed based on morphological features (Pruitt, 1954; Rudd, 1955); sex assignments were subsequently validated with the methylation assay(Wang et al., 2021).

M22. Marsupials [74]

The marsupials study involves several marsupial species as well as samples from mice and humans, for contrast analysis or to establish dual-species epigenetic clocks [74].

Ethics for opossum

These procedures are in accordance with the AVMA Guidelines for the Euthanasia of Animals 2013: https://www.avma.org/KB/Policies/Documents/euthanasia.pdf, and all animal procedures were approved by the UCLA IACUC.

Ethics for Tasmanian devils

Ear samples were obtained from Tasmanian devils by the Save the Tasmanian Devil Program (STDP) or the respective zoos, following the STDP's Standard Operating Procedure for trapping and handling. These samples were shared with the University of Sydney for research purposes. The collection of samples aligns with established ethical practices that received approval over 15 years ago. Tasmanian devils housed in zoos are provided with appropriate diets and managed in accordance with the ZAA Husbandry Guidelines for the species[75].

Ethics for Kangaroos and wallabies

The blood samples for kangaroo and wallaby were opportunistically collected from zoo-based animals during routine health exams.

Ethics for human samples

Ethics approval: IRB#15–001,454, IRB#16–000,471, IRB#18–000,315, IRB#16–002,028.

Opossum and mouse tissue samples

Opossum samples come from a pedigreed, breeding colony of gray short-tailed opossums (Monodelphis domestica) that was established by founder individuals purchased from the Southwest Foundation for Biomedical Research. Mouse samples come from a breeding colony of C57BL/6J mice originally purchased from the Jackson Laboratory. Both colonies are maintained by the Sears Lab at UCLA. Opossums were euthanized by CO2 inhalation to effect followed by bilateral thoracotomy. Mice were euthanized by CO2 inhalation to effect followed by cervical dislocation. These procedures are in accordance with the AVMA Guidelines for the Euthanasia of Animals 2013: https://www.avma.org/KB/Policies/Documents/euthanasia.pdf, and all animal procedures were approved by the UCLA IACUC. All tissue samples, e.g., liver, blood, tail, were taken from euthanized opossums and mice and stored at -20 C until use. DNA from samples was extracted and purified using a DNA Miniprep Plus Kit (Zymo), following manufacturer's protocols. PicoGreen fluorescent dsDNA was used to assess the concentration of resulting DNA and concentrations adjusted to 50 to 250 ng/ul. DNA samples were submitted to the Technology Center for Genomics & Bioinformatics at UCLA for generation of DNA methylation data and further analyses.

Tasmanian devils [74]

Samples

Ear samples from Tasmanian devil (*Sarcophilus harrisii*) were collected from individuals in the Tasmanian devil insurance metapopulation, either living as an introduced population on Maria Island (N=29), or in the zoo-based population (N=17). Samples were collected by the Save the Tasmanian Devil Program (STDP), or the respective zoos, under the STDP's Standard Operating Procedure: Trapping and handling wild Tasmanian devils and shared with the University of Sydney. The samples were collected for standard management practice, which received ethics approval over 15 years ago. DNA from these samples were made available for use in this study, no individuals were specifically sampled for the purposes of this research.

Tasmanian devils within the zoo-based insurance population are housed in a range of scenarios from intensive housing as individuals, or as pairs in breeding season; or in large group housed enclosures, ranging from 4-10 males and 4-10 females per enclosure [76]. Tasmanian devils are not known to be a social species and housing within the Tasmanian devil insurance population is under the management of the Zoo and Aquarium Association Australasia [76]. Devils are housed and fed according a variety of diet items and managed according to the ZAA Husbandry Guidelines for the species [75]. Devils living on Maria Island are wild devils and so there is no animal care or maintenance for these individuals other than twice yearly monitoring trapping trips.

Samples were selected for this study to include a range of known age individuals and sexes. DNA was extracted using either a modified phenol-chloroform protocol [77] or the MagAttract HMW DNA kit (Qiagen, Germany; cat: 67563). DNA concentration and quality

were assessed using a Nanodrop 2000 Spectrophotometer (ThermoFisher Scientific) and 0.8% agarose gel electrophoresis for 30 minutes at 90V.

Kangaroo and wallaby tissue samples

The blood samples for kangaroo and wallaby were opportunistically collected from zoo-based animals during routine health exams. We analyzed blood from the following species: Macropus rufus (red kangaroo), Macropus giganteus (Eastern grey kangaroo), Macropus fuliginosus (Western grey kangaroo), and Macropus rufogriseus (red-necked wallaby).

M23. Mammalian liver samples (Diego Villar Lozano and Duncan Odom)

Ethics

The use of all animals in this study was approved by the Animal Welfare and Ethics Review Board under reference number NRWF-DO-02vs and followed the Cancer Research UK Cambridge Institute guidelines for the use of animals in experimental studies. Tissue samples from humans were obtained from Addenbrooke's Hospital at the University of Cambridge under license number 08-H0308-117, specifically for the study "Liver specific transcriptional regulation."

Methods

We obtained mammalian tissue samples from routine euthanasia procedures (e.g. macaque, marmoset, rabbit, cat, dog, horse, and opossum), commercial providers or abattoirs (e.g. cow, pig, ferret, and guinea pig), and research tissue banks from specialty conservation programmes (e.g. cetaceans, Damaraland mole-rat and tissue samples from zoo post-mortem examinations). Tissue sources of these samples have been reported in previous publications [78-80]. In most cases, tissues were obtained immediately

post-mortem (typically within an hour) to maximize experimental quality. Tissues were kept on ice until processed to minimize potential loss of integrity during post-mortem time, and flash-frozen in dry ice. 20-50 mg of tissue was used for extraction of genomic DNA with standard commercial kits (Qiagen), and genomic DNA samples were plated in 96-well plates using a randomised layout obtained with the R package OSAT [81].

M24. Mammalian liver samples from the University of Rochester

Ethics

All experiments were performed according to procedures approved by the University of Rochester Committee on Animal Resources (UCAR), under animal protocol #101939 / UCAR-2017-033. The tissues used in the study were obtained from the Gorbunova and Seluanov tissue bank at the University of Rochester.[82,83].

Supplementary Note 2: List of GEO accession

The data across all species from the Mammalian Methylation Consortium can be downloaded from Gene Expression Omnibus (GEO) using the accession number GSE223748. Subsets of the data can be accessed from individual data sets. Below, we list the GEO accession number (GEO), Title, and sample size (N).

GEO	Title	N
GSE174758	Methylation studies in prairie voles	144
GSE184211	Methylation studies in multiple human tissues N16	661
GSE184213	Methylation studies in multiple human tissues N36	96
GSE184215	Methylation studies in multiple human tissues N47	95
GSE184216	Methylation studies in roe deer	94
GSE184218	Methylation studies in multiple human tissues N66	92
GSE184220	Methylation studies in human and mouse placenta	47
GSE184221	Methylation studies in human blood N94 N68	384
GSE184224	Methylation studies in human cells N76	17
GSE190660	Methylation studies in the vervet monkey	240
GSE190661	Methylation studies in baboons.	325
GSE190662	Methylation studies in strepsirrhini primates	91
GSE190663	Methylation studies in common marmoset	96
GSE190664	Methylation studies in rhesus macaque	283
GSE174544	Methylation studies in yellow-bellied marmots	159
GSE190665	In vivo partial reprogramming in 4F mice	168
GSE174767	Methylation studies in horses	333
GSE184222	Methylation studies in wild ass and Grevy's zebra	12
GSE184223	Methylation studies in plains zebras	118
GSE174777	Methylation studies in naked mole rats. Part 1	289
GSE174778	Methylation studies in mole rats. Part 2	94
GSE173330	Methylation clocks for age estimation in toothed whales and dolphins	545
GSE164127	Genome Methylation Predicts Age and Longevity of Bats	908
GSE147002	DNA methylation profiles from a mouse model of Huntington's disease	72

GSE147003	DNA methylation profiles from a transgenic sheep model of Huntington's disease	168
GSE147004	DNA Methylation Study of Huntington's Disease and Motor Progression in Three Species	348

Supplementary Note 3: Sensitivity analysis of enrichment results in GREAT analysis

It is critical to use a suitable background when it comes to any gene/pathway enrichment study. The wrong choice of background could easily lead to erroneous but highly significant associations due to hidden biases. When it comes to the mammalian array, the choice of the proper background must reflect the following sources of bias. First, limited genome coverage provided by the 37,000 CpGs on the array. For example, the CpGs on the mammalian array cover 6,871 human and 5,659 mouse genes when each CpGs is assigned uniquely to its closest gene neighbor. Second, by design, the mammalian array is biased toward highly conserved genomic regions. To address these biases, we evaluated the GREAT analysis software tool. As illustrated below, we find that GREAT analysis effectively deals with these biases and leads to biologically meaningful insights. In the following, we will report results from two different sensitivity analyses that were inspired by our GREAT enrichment analysis of the top 1,000 age-related CpGs (EWAS of age). Our first sensitivity analysis involved a random set of 1,000 mammalian CpGs. In essence, this evaluates the null hypothesis of no relationship between chronological age and methylation. The most significant (nominal) enrichment p-value was $P=3.9\times10^{-4}$. Note that this p-value is far less significant than the enrichment p-values for age-related CpGs in our article: top 1,000 negative CpGs lead to $P=2.7\times10^{-8}$; top 1,000 positive age-related CpGs lead to $P=2.7\times10^{-266}$. We repeated this analysis with several sets of random 1,000 CpGs and obtained similar results.

Second, we also evaluated the enrichment of the top 1,087 most highly conserved CpGs across 158 mammalian genomes. This sensitivity analysis addresses the concern that highly conserved CpGs could have an increased chance of correlating strongly with chronological age or, conversely, non-conserved (noise) CpGs are expected to have no signal for age and will therefore not be selected in an EWAS of age. This hidden bias would manifest itself as follows: the enrichment analysis of our meta-analysis EWAS for age would be equivalent to the EWAS of highly conserved CpGs. In the following, we provide details that demonstrate that this is not the case. This biologically meaningful set of 1,087 highly conserved CpGs led to highly significant enrichment p-values for gene sets involved in RNA processing, RNA splicing, and lipoprotein particle biosynthesis. Some of the top gene families of these conserved probes include RBM and LDLR. For example, for ontology class "MSigDB Cancer Neighborhood" we find P=5.2x10$^{\mathbf{-19}}$ for "Neighborhood of SMC1L1", P=2.67x10^{-18} for "Neighborhood of TDG", P=1.57x10^{-16} for “Neighborhood of XRCC5”. Highly significant GO Biological Processes include RNA processing (P=1.56x10^{-17}), RNA binding (P=5.90x10^{-16}), mRNA processing (P=1.15x10^{-14}), and RNA splicing (P=3.9x10^{-11}). However, these enrichments are quite distinct from those observed for the EWAS of age. RNA splicing and processing only showed a weak significance (P=0.05–1.4x10^{-3}) in hypomethylated age-related CpGs.. In summary, we did not observe any overlap between the top enrichment terms for the age-related CpGs with those from highly conserved regions (or those from a random set of CpGs). A detailed enrichment analysis of all the CpGs on the mammalian array can be found in Arneson et al[84].

GREAT was not explicitly designed to adjust for the issue of certain CpG's having more power to detect association based on working in more species, but it appears not to be driving categories of enrichment for age. Overall, our sensitivity analysis of the enrichment study demonstrates that GREAT analysis adjusted for potential biases arising from the design of the mammalian array and protected us against spurious associations.

Supplementary Note 4: Human cohorts

Framingham Heart Study Cohort (FHS)

The FHS cohort[85] is a large-scale longitudinal study initiated in 1948, originally designed to explore the common factors and characteristics contributing to cardiovascular disease (CVD) (https://www.framinghamheartstudy.org/index.php). This study initially enrolled participants from the town of Framingham, Massachusetts, who were asymptomatic for overt CVD, heart attack, or stroke at the time of enrollment. In 1971, the FHS Offspring Cohort was established to encompass a second generation of participants, specifically the adult children and their spouses of the original cohort (n=5124), for similar examinations[86]. Participants from the FHS Offspring Cohort were included in our study if they had attended the 8th examination cycle and consented to the use of their molecular data for research purposes. We utilized data from 2,544 participants with available DNA methylation profiles (measured at exam 8), obtained from the group providing Health/Medical/Biomedical consent (IRB, MDS). The FHS data can be accessed through the dbGaP (accession numbers: phs000363.v16.p10 and phs000724.v2.p9). Ascertainment of deaths among FHS participants that occurred prior to January 1, 2013, was carried out through multiple approaches. These included regular interaction with participants for health history updates, surveillance at local hospitals, scrutiny of local newspaper obituaries, and checks against the National Death Index. Documentation such

as death certificates, hospital and nursing home records prior to death, and autopsy reports were sought. In instances where the cause of death was uncertain, interviews with the next of kin were conducted. The cause and date of death were then reviewed and confirmed by a panel of three investigators.

DNA methylation quantification

Peripheral blood samples were collected during the 8th examination. Genomic DNA was extracted from the buffy coat utilizing the Gentra Puregene DNA extraction kit (Qiagen) and subsequently subjected to bisulfite conversion via the EZ DNA Methylation kit (Zymo Research Corporation). The quantification of DNA methylation was executed in two separate laboratory batches employing the Illumina Infinium HumanMethylation450 array (Illumina). Methylation beta values were generated with the Bioconductor minfi package, applying Noob background correction[87].

Women's Health Initiative (WHI)

The Women's Health Initiative, a national landmark study, recruited postmenopausal women aged between 50-79 years into the clinical trials (CT) or observational study (OS) cohorts between 1993 and 1998[88,89]. We incorporated data from 2,017 WHI participants from "*Broad Agency Award 23*" (WHI BA23) which had available phenotype and DNA methylation array data. WHI BA23 is dedicated to the identification of miRNA and genomic biomarkers of coronary heart disease (CHD), with an aim to integrate these biomarkers into diagnostic and prognostic predictors of CHD and associated phenotypes. This study encompasses three WHI sub-cohorts, namely GARNET, WHIMS, and SHARe.

DNA methylation quantification

In brief, bisulfite conversion was performed utilizing the Zymo EZ DNA Methylation Kit (Zymo Research, Orange, CA, USA), followed by hybridization to the

HumanMethylation450k Bead Chip (Illumina, San Diego, CA), and scanning using iScan (Illumina). All these procedures were conducted in accordance with the manufacturers' protocols, employing standard settings. For the determination of DNA methylation levels (β values), the ratio of intensities between methylated (signal A) and un-methylated (signal B) sites was calculated. Specifically, the β value was calculated from the intensity of the methylated (M corresponding to signal A) and un-methylated (U corresponding to signal B) sites, as the ratio of fluorescent signals $\beta = Max(M,0)/[Max(M,0)+Max(U,0)+100]$. Thus, β values range from 0 (completely un-methylated) to 1 (completely methylated).

Supplementary Note 5: Complementary information for Methods

In the following section, we provide an expanded description of **Methods** section in the manuscript, encompassing statistical models, distribution of study variables, and related parameters.

Human epidemiological cohort studies for diet, clinical biomarkers, and lifestyle factors

We conducted a robust correlation analysis (biweight midcorrelation, bicor[90]) between (1) our AgeAccel measures from clocks 2 and 3, and (2) 59 variables encompassing 27 self-reported dietary factors, 9 dietary biomarkers, 17 clinical measurements related to vital signs, metabolic traits, inflammatory markers, cognitive and lung function, central adiposity, leukocyte telomere length, and 6 lifestyle factors. This bicor analysis was applied to individuals from both FHS (up to n=2544) and WHI (up to n=2107), stratified by gender and ethnic category within each respective cohort. The results were consolidated using fixed-effects meta-analysis models, weighted by inverse variance, generating a meta-estimate of bicor and meta P-value.

The clinical biomarkers in FHS offspring cohort were measured during the 8th examination aligned with the measures of DNA methylation profiles. The 9 dietary biomarkers, however, were only available in the WHI cohort, with measurements taken from fasting plasma collected at baseline. Food groups and nutrients considered were comprehensive, encompassing all types and preparation methods; for instance, folic acid included both synthetic and natural forms, and dairy encompassed cheese and all varieties of milk. Further details on the individual diet variables of the WHI cohort can be found in our previous study [91].

Array Converter for Mammalian array

Since the human epidemiological cohort data were generated on a different genomic platform (Illumina 450K array), we developed an imputation scheme for converting measurements between the two platforms. The Array Converter from Human 450K Array to our Mammalian array was developed based on a study of n=141 human blood samples that were profiled using both the mammalian array and the Illumina 450k array.

Next, we randomly split the data into training (80 percent) and test set (20 percent) ensuring that both data sets had a similar age distribution. In the training set, we fit penalized regressions (with elastic net penalty, alpha=0.5) for each mammalian CpG (dependent variable). To ensure that the predicted beta values lie between 0 and 1, we applied a logit transformation to each target mammalian CpG, $y = log_2\left(\frac{M}{1-M}\right)$, where M denotes the beta value. As covariates, we used the subset of CpGs shared between the two array platforms. We did not consider all available covariates/CpGs for a given target mammalian CpG. Rather, we focused on a subset of CpGs located in the genomic interval surrounding each target CpGs with bandwidth w upstream and downstream of the target CpG. We selected the bandwidth $w = 60\ Mb$ since it turned out to maximize the accuracy

(R squared value) of array conversions in the test set. Finally, the sparse coefficient vectors fitted from the penalized regressions were stored as the array converters from 450K CpGs to the mammalian CpGs. For each mammalian CpG, denoted as M, the imputed values are computed using the following formula:

$$\widehat{M} = \frac{2^{\hat{y}}}{1 + 2^{\hat{y}}};\ \hat{y} = X_S\widehat{\beta_S},$$

where the set S denotes the sparse subset of 450K CpGs corresponding to non-zero coefficient values. The accuracy of an imputed CpGs is guided by robust biweight midcorrelation (bicor)[90] and Pearson correlation. We found that 87% CpGs in Clock 2 and 89% CpGs in Clock 3 exceeded the correlation threshold of 0.6. In calculating the Clock estimates, we replaced the methylation levels by 0.5 for the rest of CpGs that did not satisfy the threshold.

Polygenic models for heritability analysis

Previous studies have illustrated that Horvath's human pan-tissue clock[92] and several human epigenetic clocks are heritable[93,94]. To evaluate whether our pan-mammalian Clocks 2 and 3 are heritable as well, we estimated the narrow sense heritability using the polygenic models defined in SOLAR [95] and its R interface solarius [96]. Heritability is defined as the total proportion of phenotypic variance attributable to genetic variation in the polygenic model. The robust polygenic model (with the option of a t-distribution) was used to estimate heritability of the age acceleration measures of Clock 2, Clock 3 as well as Horvath's human pan-tissue clock. The heritability estimate corresponds to the variance component associated with the kinship coefficient that is incorporated in the polygenic model.

EWAS of a single tissue

For the singular cerebral cortex brain region, we simply combined the tissue specific EWAS results across different species using the unweighted Stouffer's method (**Supplementary Data 1.7**). Similarly, we carried out the one-step meta-analysis EWAS of blood, liver, muscle, and skin (**Supplementary Data 1.8--1.11**). We combined blood and spleen samples into one category (blood) because unsupervised hierarchical clustering shows that the two sources of DNA are highly comparable. In an analogous manner, skin and ear samples were combined into one category (skin). Blood EWAS results were combined across six taxonomic orders (n=4,513) including 584 tissues from humans, 742 from dogs, 406 from mice, 36 from killer whales, 143 from bottlenose dolphins, 83 from Asian elephants. Skin EWAS results were combined across seven orders (n=2,363) including 79 from bowhead whales, 667 tissues from 19 bat species, 183 from killer whales, 107 from naked mole rats, 74 from humans. Liver EWAS results were combined across four orders (n=1,063) including 600 mice, 97 from humans, 48 from horses, etc. Muscle EWAS results were combined across three orders (n=354) including 24 from evening bats, 57 from humans, and 21 from naked mole rats, etc. Cerebral cortex EWAS results were combined across two orders (n=375) including, 46 from humans, 48 from vervet monkeys and 15 from naked mole rats.

Analysis of late-replicating domains

To annotate our age-related CpGs based on late-replicating regions, we used the annotations (hg19 and mm10) from Zhou et al. [3], which can be found at https://zwdzwd.github.io/pmd. Based on the partially methylated domain (PMD) and the highly methylated domain (HMDs) annotation, each CpG was assigned to one of three categories: common PMD, common HMD or neither. For hg19, 12527 CpGs (33.5%)

on the mammalian methylation array are in common PMD, 13589 CpGs (36.2%) are in common HMD, 10087 CpGs (26.9%) are in neither group, and 1289 CpGs (3.4%) are missing for the category. For mm10, 2615 CpGs (9%) are in common PMD, 10593 CpGs (35.7%) are in common HMD, 15306 CpGs (51.6%) are in neither group, and 1123 CpGs (3.8%) are missing for the category. We also annotated CpG based on solo status (local sequence context WCGW), which is associated with hypomethylation in PMD regions. Similar to the annotation analysis based on universal chromatin states, we performed one-sided hypergeometric analysis to study the overlap of our age-related CpGs with a) common PMD/HMD structures and (b) solo-WCGW structures: genome-wide (solo-WCGW) and those in the common PMD regions (solo-WCGW common PMDs). The background in our hypergeometric test analysis was chosen according to the underlying genome assembly (hg19 or mm10).

GREAT enrichment analysis

We applied the GREAT analysis software tool[97] to the top 1,000 positively age-related and the top 1,000 negatively age-related CpGs from EWAS of age. GREAT implemented foreground/background hypergeometric tests over genomic regions where we input all 37,492 CpGs of the mammalian array as background and the genomic regions of the 1,000 CpGs as foreground. This yielded hypergeometric P-values not confounded by the number of CpGs within a gene. We performed the enrichment based on the settings (human genome assembly: hg19, Proximal: 5.0 kb upstream, 1.0 kb downstream, plus Distal: up to 50 kb) for about 76,290 gene sets associated with GO terms, MSigDB (including gene sets for upstream regulators), PANTHER, KEGG pathway, disease ontology, gene ontology, and human and mouse phenotypes. We report the gene sets

with FDR<0.05, nominal hypergeometric P-values <0.001, and number of overlapping genes ≥3.

EWAS-TWAS overlap analysis

Our EWAS-TWAS based overlap analysis related the gene sets found by our EWAS of age with the gene sets from our in-house TWAS database. To build the TWAS database, we collected published large-scale bulk or single-cell transcriptomic studies of chronological age. We also downloaded and integrated curated datasets of known aging-related genes from GenAge[98] and Enrichr[99,100] website (Aging_Perturbations_from_GEO, http://amp.pharm.mssm.edu/Enrichr/). The GenAge database includes genes related to aging in human and model organisms (Tacutu et al., 2018). Enrichr (Chen et al., 2013; Kuleshov et al., 2016) using a crowdsourcing method to extract gene expression signatures of aging from GEO. In total, our in-house TWAS database contains more than 700 aging-related gene sets across various species and tissue/cell types. For each TWAS gene set, we pruned genes present in our mammalian array. For each EWAS result, we studied the genomic regions from the top 1,000 CpGs which increased and decreased with age, respectively. CpGs were annotated to their proximal genes based on human genomic location. The gene assignments are highly orthologous across species[101]. To assess the overlap, we performed hypergeometric analysis and reported one-sided P-value for enrichment assessment. The number of background genes in the hypergeometric test was the total number of TWAS species-specific orthologous genes in our mammalian array. The hypergeometric analysis was performed at the gene level to annotate TWAS results. To avoid the bias of the array, we conducted permutation tests using the following steps: 1) Randomly sample 1,000 CpGs among any on the array and conduct EWAS-TWAS

on the permutation CpGs. For each TWAS gene set, a right-tail P-value is calculated. 2) Repeat Step 1 1,000 times to populate a list of P-values and model the resulting null distribution with a Gamma function. The cumulative distribution function (CDF) is also calculated. 3) Calculate the permutation P-value based on the CDF from step 2. In our study, we restricted the TWAS with permutation $P<0.05$, and highlighted the top 10 TWAS gene sets based on the unadjusted hypergeometric P-value. We only reported these TWAS results with ≥5 overlap genes.

EWAS-GWAS overlap analysis

In our EWAS-GWAS overlap analysis, we compiled a broad range of published, large-scale GWAS data. The data spanned various phenotypes including anthropometric traits, behavioral phenotypes, cognitive related traits, fetal growth traits, inflammatory diseases, lipid panel outcomes, metabolic outcomes, neurological diseases, longevity, reproductive aging, six DNAm-based biomarkers, and additional age-related traits. This compilation comprised a total of 102 GWAS results. The six DNAm biomarkers included four epigenetic age acceleration measures derived from 1) pan-tissue epigenetic age adjusted for age-related blood cell counts referred to as intrinsic epigenetic age acceleration (IEAA)[92,102], 2) Hannum's blood-based DNAm age[103], 3) DNAmPhenoAge[104]; and 4) the mortality risk estimator DNAmGrimAge[93], along with DNAm-based estimates of blood cell counts for granulocytes and plasminogen activator inhibitor 1 (PAI1) levels[93]. GWAS summary datasets in VCF format were managed by R gwasvcf [105]. For each GWAS result, we employed the MAGENTA software to calculate an overall GWAS *P*-value at gene level, which is based on the most significant SNP association *P*-value within the gene boundary (± 50 kb) adjusted for gene size, number of SNPs per kb, linkage disequilibrium, and other potential confounders[106]. The MAGENTA analysis was performed in MATLAB

(2017 version). We restricted the analysis to genomic regions of GWAS genes present on the mammalian array. For each EWAS result, we studied the genomic regions from the top 1,000 CpGs with positive and negative age correlations, respectively. To assess the overlap with a test trait, we selected the top 2.5 % genes for each GWAS trait and calculated one-sided hypergeometric *P*-values based on genomic regions. Other thresholds would lead to qualitatively similar results. We report GWAS traits that led to a significant hypergeometric test (nominal *P*-value $<5\times10^{-4}$, Bonferroni corrected $P<0.05$) for any EWAS of age. The number of background genomic regions in the hypergeometric test was based on the overlap between all genes in the GWAS and all genomic regions represented by the mammalian array.

All GWAS results are based on meta-analysis across large-scale human studies. For instance, GWAS of anthropometric traits involved more than 200k individuals from multiple ethnic groups, conducted by the GIANT consortium, https://portals.broadinstitute.org/collaboration/giant/index.php/GIANT_consortium.

The GWAS summary datasets were downloaded from OpenGWAS [105,107,108] (https://gwas.mrcieu.ac.uk/) or obtained from the corresponding study groups. Citations to the respective scientific papers are provided below.

Index	Hg	Category	Trait	Ethnicity	Sex	PMID
1	hg19	Neurodegenerative disorder	Age-related Macular degeneration (AMD)	EUR+ASN	All	23455636
2	hg19	Neurodegenerative disorder	AMD Geographic Atrophy	EUR+ASN	All	23455636
3	hg19	Neurodegenerative disorder	AMD Neovascular	EUR+ASN	All	23455636
4	hg19	Neurodegenerative disorder	Alzheimer's disease	EUR	All	24162737
5	hg18	Longevity	Longevity > 90	EUR	All	24688116

6	hg18	Longevity	Longevity > 85	EUR	All	24688116
7	hg19	Neurodegenerative disorder	Parkinson's disease	EUR	All	19915575
8	hg19	Neuropsychiatric disorder	Schizophrenia	All	All	25056061
9	hg19	Inflammatory diseases	IBD	EUR	All	26192919
10	hg19	Inflammatory diseases	IBD Crohn's disease	EUR	All	26192919
11	hg19	Inflammatory diseases	IBD Ulcerative colitis	EUR	All	26192919
12	hg18	Neuropsychiatric disorder	Bipolar disorder	All	All	21926972
13	hg18	Neuropsychiatric disorder	ADHD	All	All	20732625
14	hg18	Neuropsychiatric disorder	Major depression disorder	EUR	All	22472876
15	hg18	Metabolic outcomes and diseases	Type 2 diabetes	EUR	All	22885922
16	hg18	Metabolic outcomes and diseases	Fasting glucose	EUR	All	22581228
17	hg18	Metabolic outcomes and diseases	Fasting insulin	EUR	All	22581228
18	hg18	GIANT Body fat distribution	Hip AllAncestries	ALL	M&F	25673412
19	hg18	GIANT Body fat distribution	Hip EUR	EUR	M&F	25673412
20	hg18	GIANT Body fat distribution	Hip AllAncestries(Males)	ALL	M	25673412
21	hg18	GIANT Body fat distribution	Hip EUR (Males)	EUR	M	25673412
22	hg18	GIANT Body fat distribution	Hip AllAncestries(Females)	ALL	F	25673412
23	hg18	GIANT Body fat distribution	Hip EUR (Females)	EUR	F	25673412
30	hg18	GIANT Body fat distribution	Waist circumference AllAncestries	ALL	M&F	25673412
31	hg18	GIANT Body fat distribution	Waist circumference EUR	EUR	M&F	25673412
32	hg18	GIANT Body fat distribution	Waist circumference AllAncestries(Males)	ALL	M	25673412
33	hg18	GIANT Body fat distribution	Waist circumference EUR (Males)	EUR	M	25673412

34	hg18	GIANT Body fat distribution	Waist circumference AllAncestries(Females)	ALL	F	25673412
35	hg18	GIANT Body fat distribution	Waist circumference EUR (Females)	EUR	F	25673412
42	hg18	GIANT Body fat distribution	Waist to hip ratio AllAncestries	ALL	M&F	25673412
43	hg18	GIANT Body fat distribution	Waist to hip ratio EUR	EUR	M&F	25673412
44	hg18	GIANT Body fat distribution	Waist to hip ratio AllAncestries(Males)	ALL	M	25673412
45	hg18	GIANT Body fat distribution	Waist to hip ratio EUR (Males)	EUR	M	25673412
46	hg18	GIANT Body fat distribution	Waist to hip ratio AllAncestries(Females)	ALL	F	25673412
47	hg18	GIANT Body fat distribution	Waist to hip ratio EUR (Females)	EUR	F	25673412
54	hg18	GIANT BMI & Height	BMI	EUR	All	25673413
55	hg18	GIANT BMI & Height	Height	EUR	All	20881960
56	hg19	Neurodegenerative disorder	Frontotemporal dementia	EUR	All	24943344
57	hg19	Neurodegenerative disorder	FTD Behavioral variant	EUR	All	24943344
58	hg19	Neurodegenerative disorder	FTD with motor neuron disease	EUR	All	24943344
59	hg19	Neurodegenerative disorder	FTD progressive non-fluent aphasia	EUR	All	24943344
60	hg19	Neurodegenerative disorder	FTD semantic dementia	EUR	All	24943344
61	hg19	Neurodegenerative disorder	Huntington's disease age onset	EUR	All	26232222
62	hg19	Behavioral phenotype	Educational attainment	EUR	All	27225129
63	hg19	Behavioral phenotype	Educational attainment (Males)	EUR	All	27225129
64	hg19	Behavioral phenotype	Educational attainment (Females)	EUR	All	27225129
65	hg18	Reproductive aging	Age at menarche	EUR	All	25231870
66	hg18	Reproductive aging	Age at menopause	EUR	All	26414677
67	hg18	Lipid panel outcomes	HDL		All	24097068
68	hg18	Lipid panel outcomes	LDL		All	24097068

69	hg18	Lipid panel outcomes	Total cholesterol		All	24097068
70	hg18	Lipid panel outcomes	Triglyceride		All	24097068
71	hg18	Reproductive aging	Leukocyte telomere length	EUR	All	23535734
72	hg19	DNAm biomarkers	AgeAccelGrim EUR	EUR	All	34187551
73	hg19	DNAm biomarkers	DNAmGranAdjustedAge EUR	EUR	All	34187551
74	hg19	DNAm biomarkers	AgeAccelHannum EUR	EUR	All	34187551
75	hg19	DNAm biomarkers	DNAmPAI1AdjAge EUR	EUR	All	34187551
76	hg19	DNAm biomarkers	IEAA EUR	EUR	All	34187551
77	hg19	DNAm biomarkers	AgeaccelPhenoAge EUR	EUR	All	34187551
78	hg19	DNAm biomarkers	AgeAccelGrim AFR	AFR	All	34187551
79	hg19	DNAm biomarkers	DNAmGranAdjustedAge AFR	AFR	All	34187551
80	hg19	DNAm biomarkers	AgeAccelHannum AFR	AFR	All	34187551
81	hg19	DNAm biomarkers	DNAmPAI1AdjAge AFR	AFR	All	34187551
82	hg19	DNAm biomarkers	IEAA AFR	AFR	All	34187551
83	hg19	DNAm biomarkers	AgeaccelPhenoAge AFR	AFR	All	34187551
84	hg19	DNAm biomarkers	AgeAccelGrim All	EUR+AFR	All	34187551
85	hg19	DNAm biomarkers	DNAmGranAdjustedAge All	EUR+AFR	All	34187551
86	hg19	DNAm biomarkers	AgeAccelHannum All	EUR+AFR	All	34187551
87	hg19	DNAm biomarkers	DNAmPAI1AdjAge All	EUR+AFR	All	34187551
88	hg19	DNAm biomarkers	IEAA All	EUR+AFR	All	34187551
89	hg19	DNAm biomarkers	AgeAccelPhenoAge All	EUR+AFR	All	34187551
90	hg19	Longevity	Father's attained age	EUR	All	29227965
91	hg19	Longevity	Mother's attained age	EUR	All	29227965
92	hg19	Longevity	Parental attained age	EUR	All	29227965
93	hg19	Age related phenotype	Atrial fibrillation	EUR	All	30061737
94	hg19	Neurodegenerative disorder	Alzheimer's disease	EUR	All	30617256
95	hg19	Cognitive related	Intelligence	EUR	All	29942086
96	hg19	Reproductive aging	AgeAtMenarche	EUR	All	28436984
97	hg19	Neurodegenerative disorder	Huntington's disease motor progression	EUR	All	28642124
98	hg19	Fetal growth	Birth length	EUR	All	34282336
99	hg19	Fetal growth	Infant Ponderal index	EUR	All	34282336
100	hg19	Fetal growth	Birth weight	EUR	All	34282336
101	hg19	Fetal growth	Birth weight fatherGenome	EUR	All	34282336

102	hg19	Fetal growth	Birth weight motherGenome	EUR	All	34282336
EUR: Europeans; AFR: Africans; ASN: Asians.						

Transcription factor binding

We used the CellBase database[109] with the ENCODE[110] transcription factor binding sites. A total of 186 TFs across 70 genes could be mapped to CpGs on our Mammalian array. The Chip-seq analysis of these TFs was done in 17 different cell types depending on the target molecule (**Supplementary Data 11.1**). We annotated a CpG with specific TF if it overlapped with the respective binding site in at least one cell type (in hg19 assembly). TFs with extreme annotation size (number of genes across binding regions < 5 or >2000) were removed, yielding 68 TFs remaining in our analysis.

Mouse single cell ATAC-seq in hematopoietic stem cells

We downloaded the publicly available data (H5, meta and fragment files of Illumina HiSeq 1500 array data) from Itokawa et al.[111] (GSE162662).

Single cell ATAC-seq data were profiled in 4 biologically replicates in young (10 weeks) and old mice (20 months). The ATAC-seq data were managed and analyzed with R Signac[112]. We preprocessed 5,028 cells in the young group and 3,694 cells in the old group using the R Signac/CreateChromatinAssay function for quality controls. We removed cells with fewer than 200 detected features from the analysis, resulting in 4,492 cells for the young group and 3,300 cells for the old group. For the 29,637 CpGs on the mammalian methylation array that map to the mouse genome (mm10) we determined overlapping ATAC seq peak locations using GenomicRanges [113]. Of those, 20,092 CpGs exhibited at least one overlap with an ATAC called peak in both the young and the old group, including 33 out of the 35 positively age-related CpGs. For each CpG/peak location we estimated the proportion of cells with a detectable peak as the ratio of the number of

called peaks divided by the total number of cells (3,694 young cells and 4,492 old cells). The median of the proportion estimate was 0.15% [0.02%,14.1%] in young mice and 0.21% [0.03%, 14.6%] in old mice. We used Fisher's exact test analysis to determine whether differentially accessible locations between young and old animals were enriched with the 33 top positively age-related CpGs (OR >1 indicates a higher proportion in the old group).

Supplementary Note 6: Murine anti-aging studies

None of the samples from the murine anti-aging studies were used in the training set of the universal clocks, i.e., these are truly independent test data. Clocks 2 and 3 were evaluated in five mouse experiments (independent test data): (1) Snell dwarf mice (n=95), (2) growth hormone receptor knock-out experiment 1 (GHRKO, n=71 samples), (3) GHRKO experiment 2 (n=96 samples), (4) three Tet experiments: Tet1 KO (n=64), Tet2 KO (n=65) and Tet3 KO (n=63), and (5) calorie restriction (n=95). These experiments were known to prolong the lifespan of mice. We simply performed t-tests to investigate if these conditions slowed epigenetic aging rates based on our universal clocks. The DNA methylation data in (1) Snell dwarf mice and (3) the GHRKO experiment 2 were profiled in an Illumina 320k customized array for mouse study (deposited in GSE223943 and GSE223944). The other datasets (2, 4 and 5) can be found in Complete dataset: GSE223748. DNA methylation data for the other experiments were profiled in our mammalian array. As noted, our Clock2 (816 CpGs) and Clock3 (760 CpGs) contain a total of 1143 unique mammalian CpGs. Of the 1143 CpGs, 165 sites led to poor quality measurements in the custom 320k array. The methylation levels of these 165 sites were imputed using the median beta values from the 2205 mouse samples that were used for training our universal clocks. Below, we

briefly described the experiments. The details for each experiment are listed in **Supplementary Information, note**.

Snell dwarf experiment (n=95)

We studied multiple tissue types from n=47 Snell dwarf[114,115] and n=48 age matched wild type control mice which were about 6 months old (mean±SD of age =0.52±0.01 years). The Snell dwarf mice for loss of function at Pit1 were generated at the University of Michigan (Richard A. Miller). Experiments on mice raised at the University of Michigan were approved by the University of Michigan's Institutional Animal Care and Use Committee.

Snell dwarf mice, which lack growth hormone, thyroid stimulating hormone, and prolactin, have approximately 30% - 40% longer lifespan (http://www.richmillerlab.com/long-lived-mutants). The extended lifespan related to Snell dwarf mice was previously studied by Flurkey et al. [114]. We applied the mammalian methylation array to blood, cerebral cortex, liver, kidney, spleen and tail. For most tissue types we compared n=8 dwarf mice to n=8 age matched wild type control mice with the exception of cerebral cortex, where we compared 8 dwarf mice to 7 normal control mice.

GHRKO experiment 1: full body growth hormone receptor knock-out (n=71)

We studied several mouse tissue types from a growth hormone receptor knock-out (GHRKO, double knock-out in all tissues) study [116]. The GHRKO mice (GHR -/- dwarfs) were generated at the University of Michigan (Richard A. Miller). Experiments on mice raised at the University of Michigan were approved by the University of Michigan's Institutional Animal Care and Use Committee.

The experiment was based on full-body growth hormone receptor knockout (GHR-KO) as described in details by Coschigano et al., 2003[117]. We applied the mammalian

methylation array to the cerebral cortex (n=12), liver (n=23), kidney (n=24) and hippocampus (n=23). GHRKO mice and control mice were age matched: mean±SD [118] of age is 0.66±0.06 [0.57–0.79] years in the GHRKO group and 0.65±0.06 [0.58–0.79] years in the wild-type control group. To adjust for age, we used the epigenetic age acceleration measure AgeAccel, and related it to genotype (GHRKO versus wild-type) with the non-parametric Kruskal Wallis test.

GHRKO experiment 2: liver-specific growth hormone receptor knock-out (n=96)

In the second GHRKO experiment, the growth hormone receptor was only knocked out in liver tissue[119]. These GHRKO mice (GHR -/- dwarfs) were generated at the University of Michigan (Richard A. Miller). Experiments on mice raised at the University of Michigan were approved by the University of Michigan's Institutional Animal Care and Use Committee. The experiment only knocked out the growth hormone receptors in livers. Unlike the full body GHRKO mice, liver specific GHRKO mice do not exhibit increased lifespan. We applied the mammalian methylation array to blood, cortex, liver, kidney, spleen, and tail (n=96 tissue samples). For each tissue type we compared n=8 liver-specific GHRKO mice with n=8 age matched control mice. The mean±SD [118] of age was 0.51±0.03 [0.48,0.59] years among the 96 samples.

Calorie restriction study (n=95)

We analyzed 95 male liver samples from a calorie restriction[120] (CR) study: n=59 in the CR group versus n=36 in the control group. DNA methylation arrays were profiled in both groups. DNA methylation arrays were profiled in liver samples. All mice in the study, were of the same age (1.57 years) and sex, being male. They were of the C57BL/6J inbred strain. These C57BL/6J male mice were sourced from the Mouse Breeding Core, Wakeland lab, at UT Southwestern Medical Center, Dallas, TX, USA.

Tet genes knock-out study

We analyzed three Tet experiments: *Tet1 KO (n=64)*, *Tet2* KO (n=65) and*Tet3* KO (n=63). Each experiment has approximately the same number of samples in *Tet* heterozygous KO and wild-type genotype at each tissue type. DNA methylation array was profiled in both genotypes at each tissue type: cerebral cortex and striatum. All mice have the same age (0.5 years). *Tet1* and *Tet2* knockout mice were purchased from the Jackson Laboratory (*Tet1* KO: Strain #:017358, B6; 129S4-*Tet1*$^{tm1.1Jae}$/J; *Tet2* KO: Strain #:023359, B6(Cg)-*Tet2*$^{tm1.2Rao}$/J). *Tet3* knockout mice were generated by William Yang's Lab at UCLA using the CRISPR/Cas9 method with deletion of exon 4 at the Jackson Laboratory's Customer Model Generation Core (Founder Strain# 407367, UCLA_GET3672_Tet3_KO_B6J). *Tet3* exon 4 was deleted using the following gRNAs: Up1 AGTATTATTTGGAGACAATT; Up2 TACCAAGAGCAAGTATTATT; Down1 AAATTCCCAAACGGAACCTC; Down2 ACCTGTCCAGAGGTTCCGTT). For each line, the heterozygous knockout mice were bred with C57BL/6J (JAX 000664) to set up the colony. All mice were maintained and bred under standard conditions consistent with National Institutes of Health guidelines and approved by the University of California, Los Angeles Institutional Animal Care and Use Committees. The

Supplementary References

1. Vu, H. & Ernst, J. Universal annotation of the human genome through integration of over a thousand epigenomic datasets. *Genome Biology* **23**, 9 (2022).
2. Davis, C.A. *et al.* The Encyclopedia of DNA elements (ENCODE): data portal update. *Nucleic Acids Res* **46**, D794-D801 (2018).
3. Zhou, W. *et al.* DNA methylation loss in late-replicating domains is linked to mitotic cell division. *Nature Genetics* **50**, 591-602 (2018).
4. Langfelder, P. & Horvath, S. WGCNA: an R package for weighted correlation network analysis. *BMC Bioinformatics* **9**, 559 (2008).
5. Horvath, S. *et al.* Pan-primate DNA methylation clocks. *bioRxiv*, 2020.11.29.402891 (2021).

6. Horvath, S. *et al.* Epigenetic clock and methylation studies in the rhesus macaque. *GeroScience* (2021).
7. Jasinska, A.J. *et al.* Epigenetic clock and methylation studies in vervet monkeys. *GeroScience* (2021).
8. Horvath, S. *et al.* DNA methylation age analysis of rapamycin in common marmosets. *GeroScience* (2021).
9. Schlabritz-Loutsevitch, N.E. *et al.* Metabolic adjustments to moderate maternal nutrient restriction. *British journal of nutrition* **98**, 276-284 (2007).
10. Kavitha, J.V. *et al.* Down-regulation of placental mTOR, insulin/IGF-I signaling, and nutrient transporters in response to maternal nutrient restriction in the baboon. *FASEB journal : official publication of the Federation of American Societies for Experimental Biology* **28**, 1294-1305 (2014).
11. Schlabritz-Loutsevitch, N.E. *et al.* Development of a system for individual feeding of baboons maintained in an outdoor group social environment. *Journal of Medical Primatology* **33**, 117-126 (2004).
12. Zehr, S.M. *et al.* Life history profiles for 27 strepsirrhine primate taxa generated using captive data from the Duke Lemur Center. *Scientific Data* **1**, 140019 (2014).
13. Morgello, S. *et al.* The National NeuroAIDS Tissue Consortium: a new paradigm in brain banking with an emphasis on infectious disease. *Neuropathol Appl Neurobiol* **27**, 326-35. (2001).
14. Horvath, S. *et al.* HIV, pathology and epigenetic age acceleration in different human tissues. *Geroscience* (2022).
15. Horvath, S. *et al.* Perinatally acquired HIV infection accelerates epigenetic aging in South African adolescents. *AIDS (London, England)* **32**, 1465-1474 (2018).
16. Horvath, S. & Ritz, B.R. Increased epigenetic age and granulocyte counts in the blood of Parkinson's disease patients. *Aging (Albany NY)* **7**, 1130-42 (2015).
17. Kabacik, S., Horvath, S., Cohen, H. & Raj, K. Epigenetic ageing is distinct from senescence-mediated ageing and is not prevented by telomerase expression. *Aging (Albany NY)* **10**, 2800-2815 (2018).
18. Ross, C.N. *et al.* The development of a specific pathogen free (SPF) barrier colony of marmosets (Callithrix jacchus) for aging research. *Aging (Albany NY)* **9**, 2544 (2017).
19. Sailer, L.L. *et al.* Pair bonding slows epigenetic aging and alters methylation in brains of prairie voles. *bioRxiv*, 2020.09.25.313775 (2020).
20. Ophir, A.G. Navigating Monogamy: Nonapeptide Sensitivity in a Memory Neural Circuit May Shape Social Behavior and Mating Decisions. *Frontiers in Neuroscience* **11**(2017).
21. Horvath, S. *et al.* Methylation studies in Peromyscus: aging, altitude adaptation, and monogamy. *GeroScience* **44**, 447-461 (2022).
22. Horvath, S. *et al.* DNA methylation aging and transcriptomic studies in horses. *Nat Commun* **13**, 40 (2022).
23. Burns, E.N. *et al.* Generation of an equine biobank to be used for Functional Annotation of Animal Genomes project. *Animal genetics* **49**, 564-570 (2018).
24. Horvath, S. *et al.* DNA methylation clocks tick in naked mole rats but queens age more slowly than nonbreeders. *Nature Aging* **2**, 46-59 (2022).

25. Ke, Z., Vaidya, A., Ascher, J., Seluanov, A. & Gorbunova, V. Novel husbandry techniques support survival of naked mole rat (Heterocephalus glaber) pups. *J Am Assoc Lab Anim Sci* **53**, 89-91 (2014).
26. Tan, L. *et al.* Naked Mole Rat Cells Have a Stable Epigenome that Resists iPSC Reprogramming. *Stem cell reports* **9**, 1721-1734 (2017).
27. Sugrue, V.J. *et al.* Castration delays epigenetic aging and feminizes DNA methylation at androgen-regulated loci. *eLife* **10**, e64932 (2021).
28. Schachtschneider, K.M. *et al.* Epigenetic clock and DNA methylation analysis of porcine models of aging and obesity. *GeroScience* (2021).
29. Robeck, T.R. *et al.* Multi-Tissue Methylation Clocks for Age and Sex Estimation in the Common Bottlenose Dolphin. *Frontiers in Marine Science* **8**(2021).
30. Robeck, T.R. *et al.* Multi-species and multi-tissue methylation clocks for age estimation in toothed whales and dolphins. *Commun Biol* **4**, 642 (2021).
31. Bors, E.K. *et al.* An epigenetic clock to estimate the age of living beluga whales. *Evol Appl* **14**, 1263-1273 (2021).
32. Lockyer, C., Hohn, A.A., Doidge, D.W., Heide-Jørgensen, M.P. & Suydam, R. Age determination in belugas (Delphinapterus leucas in Belugas): a quest for validation of dentinal layering. *Aquat. Mamm* **33**, 293-304 (2007).
33. McGuire, T., Michaud, R., Moisan, M. & Garner, C. Cook Inlet Beluga Whale Biopsy: Field Report for 2016 Feasibility Study. Prepared by LGL Alaska Research Associates, Inc., GREMM, and JBER for NMFS 67 p. (2017).
34. Matkin, C.O., Ellis, G.M., Olesiuk, P.F. & Saulitis, E.L. Association patterns and inferred genealogies of resident killer whales, *Orcinus orca*, in Prince William Sound, Alaska. *Fishery Bulletin* **97**, 900-919 (1999).
35. Matkin, C.O., Ward Testa, J., Ellis, G.M. & Saulitis, E.L. Life history and population dynamics of southern Alaska resident killer whales (*Orcinus orca*). *Marine Mammal Science* **30**, 460-479 (2014).
36. Bigg, M.A. An assessment of killer whale (*Orcinus orca*) stocks off Vancouver Island, British Columbia. *Report of the International Whaling Commission* **32**, 655-666 (1982).
37. Ford, J.K.B., Ellis, G.M. & Balcomb, K.C. *Killer Whales: The natural history and genealogy of Orcinus orca in British Columbia and Washington State. 2nd edition.*, (UBC Press, Vancouver, B.C., 2000).
38. Balcomb, K.C. & Bigg, M.A. Population biology of the three resident killer whale pods in Puget sound and off southern Vancouver Island. in *Behavioural Biology of Killer Whales* (eds. Kerkevold, B.C. & Lockard, J.S.) (Alan R. Liss, Inc., New York, NY, 1986).
39. Bigg, M.A., Olesiuk, P.F., Ellis, G.M., Ford, J.K.B. & Balcomb, K.C. Social Organization and Genealogy of Resident Killer Whales (*Orcinus orca*) in the Coastal Waters of British Columbia and Washington State. in *Individual Recognition of Cetaceans: Use of Photo-Identification and Other Techniques to Estimate Population Parameters*, Vol. Rep Int Whal Comm. Special Issue 12 (ed. Hammond, P.) 383-405 (Cambridge, 1990).
40. Olesiuk, P.F., Bigg, M.A. & Ellis, G.M. Life history and population dynamics of resident killer whales (*Orcinus orca*) in the coastal waters of British Columbia and

Washington State. in *Rep. Int. Whal. Commn.*, Vol. Special Issue 12 209-244 (1990).
41. Olesiuk, P., Ellis, G. & Ford, J. Life history and population dynamics of resident killer whales *Orcinus orca* in the coastal waters of British Columbia. (Nanaimo, B.C., Canada, 2005).
42. Barrett-Lennard, L.G., Smith, T.G. & Ellis, G.M. A cetacean biopsy system using lightweight pneumatic darts, and its effect on the behaviour of killer whales. *Marine Mammal Science* **12**, 14-27 (1996).
43. Parsons, K.M., Durban, J.W. & Claridge, D.E. Comparing two alternative methods for genetic sampling of small cetaceans. *Marine Mammal Science* **19**, 224-231 (2003).
44. Sambrook, J., Fritsch, E.F. & Maniatis, T. *Molecular Cloning: A Laboratory Manual, 2nd edn.*, (Cold Spring Harbor Laboratory Press, New York, 1989).
45. George, J.C. & Thewissen, J. *The bowhead whale: Balaena mysticetus: Biology and human interactions*, (Academic Press, 2020).
46. Lubetkin, S., Zeh, J. & George, J. Statistical modeling of baleen and body length at age in bowhead whales (*Balaena mysticetus*). *Canadian Journal of Zoology* **90**, 915 - 931 (2012).
47. Garde, E., Heide-Jørgensen, M.P., Hansen, S.H., Nachman, G. & Forchhammer, M.C. Age-Specific Growth and Remarkable Longevity in Narwhals (Monodon monoceros) from West Greenland as Estimated by Aspartic Acid Racemization. *Journal of Mammalogy* **88**, 49-58 (2007).
48. Palsbøll, P., Larsen, F. & Sigurd Hansen, E. Samping of skin biopsies from free-ranging large cetaceans in West Greenland: Development of new biopsy tips and bolt designs. *Reports of the International Whaling Commission (special issue)*, 71:79 (1991).
49. Clapham, P.J. Age at attainment of sexual maturity in humpback whales, Megaptera novaeangliae. *Canadian Journal of Zoology* **70**, 1470-1472 (1992).
50. Baraff, L. & Weinrich, M.T. SEPARATION OF HUMPBACK WHALE MOTHERS AND CALVES ON A FEEDING GROUND IN EARLY AUTUMN. *Marine Mammal Science* **9**, 431-434 (1993).
51. Katona, S.K. & Whitehead, H.P. Identifying Humpback Whales using their natural markings. *Polar Record* **20**, 439-444 (1981).
52. Bérubé, M. & Palsbøll, P. Identification of sex in cetaceans by multiplexing with three ZFX and ZFY specific primers. *Mol Ecol* **5**, 283-7 (1996).
53. Glockner, D. Determining the sex of humpback whales in their natural environment. *Communication and Behavior of Whales. AAAS Selected Symposium*, 447-464 in Payne R, editor (1983).
54. Raj, K. *et al.* Epigenetic clock and methylation studies in cats. *GeroScience* (2021).
55. Prado, N.A. *et al.* Epigenetic clock and methylation studies in elephants. *Aging Cell* **20**, e13414 (2021).
56. Pinho, G.M. *et al.* Hibernation slows epigenetic ageing in yellow-bellied marmots. *Nature Ecology & Evolution* **6**, 418-426 (2022).
57. Lemaître, J.-F. *et al.* DNA methylation as a tool to explore ageing in wild roe deer populations. *Molecular Ecology Resources* **n/a**(2021).

58. Larison, B. *et al.* Epigenetic models developed for plains zebras predict age in domestic horses and endangered equids. *Communications Biology* **4**, 1412 (2021).
59. Harley, E.H., Knight, M.H., Lardner, C., Wooding, B. & Gregor, M. The Quagga project: progress over 20 years of selective breeding. *African Journal of Wildlife Research* **39**, 155-163 (2009).
60. Horvath, S. *et al.* Reversing age: dual species measurement of epigenetic age with a single clock. *bioRxiv*, 2020.05.07.082917 (2020).
61. Horvath, S. *et al.* DNA methylation clocks for dogs and humans. *Proceedings of the National Academy of Sciences* **119**, e2120887119 (2022).
62. Plassais, J. *et al.* Whole genome sequencing of canids reveals genomic regions under selection and variants influencing morphology. *Nature Communications* **10**, 1489 (2019).
63. Plassais, J. *et al.* Analysis of large versus small dogs reveals three genes on the canine X chromosome associated with body weight, muscling and back fat thickness. *PLOS Genetics* **13**, e1006661 (2017).
64. TheAmericanKennelClub. *The Complete Dog Book: 20th Edition*, (Howell Book House, New York, NY, 2006).
65. Wilcox, B. & Walkowicz, C. *The Atlas of Dog Breeds of the World*, (T.F.H. Publications, 1995).
66. Wilkinson, G.S. *et al.* DNA methylation predicts age and provides insight into exceptional longevity of bats. *Nature Communications* **12**, 1615 (2021).
67. Kordowitzki, P. *et al.* Epigenetic clock and methylation study of oocytes from a bovine model of reproductive aging. *Aging Cell* **20**, e13349 (2021).
68. Mozhui, K. *et al.* Genetic loci and metabolic states associated with murine epigenetic aging. *eLife* **11**, e75244 (2022).
69. Lu, A.T. *et al.* DNA methylation study of Huntington’s disease and motor progression in patients and in animal models. *Nature communications* **11**, 1-15 (2020).
70. Acosta-Rodríguez, V.A., Rijo-Ferreira, F., Green, C.B. & Takahashi, J.S. Importance of circadian timing for aging and longevity. *Nature Communications* **12**, 2862 (2021).
71. Little, T.J. *et al.* Methylation-Based Age Estimation in a Wild Mouse. *bioRxiv*, 2020.07.16.203687 (2020).
72. Coschigano, K. *et al.* Deletion, but not antagonism, of the mouse growth hormone receptor results in severely decreased body weights, insulin, and insulin-like growth factor I levels and increased life span. *Endocrinology* **144**, 3799-3810 (2003).
73. Cossette, M.-L. *et al.* Differential methylation, epigenetic clocks, and island-mainland divergence in an insectivorous small mammal. *bioRxiv*, 2022.04.14.488253 (2022).
74. Horvath, S. *et al.* Epigenetic clock and methylation studies in marsupials: opossums, Tasmanian devils, kangaroos, and wallabies. *Geroscience* **In Press**(2022).
75. Hogg, C. & Hockley, J. DPIPWE/ZAA husbandry guidelines for Tasmanian devil, Sarcophilus harrisii. *Australia: Zoo and Aquarium Association* (2013).

76. Hogg, C.J., Lee, A.V. & Hibbard, C.J. Managing a metapopulation: intensive to wild and all the places in between. in *Saving the Tasmanian Devil: recovery through science based management* 169-182 (CSIRO Publishing Melbourne, 2019).
77. Sambrook, J. & Russell, D.W. Purification of nucleic acids by extraction with phenol: chloroform. *Cold Spring Harbor Protocols* **2006**, pdb. prot4455 (2006).
78. Villar, D. *et al.* Enhancer evolution across 20 mammalian species. *Cell* **160**, 554-66 (2015).
79. Berthelot, C., Villar, D., Horvath, J.E., Odom, D.T. & Flicek, P. Complexity and conservation of regulatory landscapes underlie evolutionary resilience of mammalian gene expression. *Nat Ecol Evol* **2**, 152-163 (2018).
80. Roller, M. *et al.* LINE retrotransposons characterize mammalian tissue-specific and evolutionarily dynamic regulatory regions. *Genome Biol* **22**, 62 (2021).
81. Yan, L. *et al.* OSAT: a tool for sample-to-batch allocations in genomics experiments. *BMC Genomics* **13**, 689 (2012).
82. Seluanov, A. *et al.* Hypersensitivity to contact inhibition provides a clue to cancer resistance of naked mole-rat. *Proceedings of the National Academy of Sciences* **106**, 19352-19357 (2009).
83. Seluanov, A. *et al.* Telomerase activity coevolves with body mass not lifespan. *Aging Cell* **6**, 45-52 (2007).
84. Arneson, A. *et al.* A mammalian methylation array for profiling methylation levels at conserved sequences. *Nature communications* **In press**(2022).
85. Dawber, T.R., Meadors, G.F. & Moore, F.E., Jr. Epidemiological approaches to heart disease: the Framingham Study. *Am J Public Health Nations Health* **41**, 279-81 (1951).
86. Kannel, W.B., Feinleib, M., McNamara, P.M., Garrison, R.J. & Castelli, W.P. An investigation of coronary heart disease in families. The Framingham offspring study. *Am J Epidemiol* **110**, 281-90 (1979).
87. Aryee, M.J. *et al.* Minfi: a flexible and comprehensive Bioconductor package for the analysis of Infinium DNA methylation microarrays. *Bioinformatics* **30**, 1363-9 (2014).
88. Anderson, G.L. *et al.* Implementation of the Women's Health Initiative study design. *Ann Epidemiol* **13**, S5-17 (2003).
89. WHIConsortium. Design of the Women's Health Initiative clinical trial and observational study. The Women's Health Initiative Study Group. *Control Clin Trials* **19**, 61-109 (1998).
90. Langfelder, P. & Horvath, S. WGCNA: an R package for weighted correlation network analysis. *BMC Bioinformatics* **9**, 559 (2008).
91. Quach, A. *et al.* Epigenetic clock analysis of diet, exercise, education, and lifestyle factors. *Aging (Albany NY)* **9**, 419-446 (2017).
92. Horvath, S. DNA methylation age of human tissues and cell types. *Genome Biol* **14**, R115 (2013).
93. Lu, A.T. *et al.* DNA methylation GrimAge strongly predicts lifespan and healthspan. *Aging (Albany NY)* **11**, 303-327 (2019).
94. Lu, A.T. *et al.* DNA methylation GrimAge version 2. *Aging (Albany NY)* **14**, 9484-9549 (2022).

95. Almasy, L. & Blangero, J. Multipoint quantitative-trait linkage analysis in general pedigrees. *Am J Hum Genet* **62**, 1198-211 (1998).
96. Ziyatdinov, A. *et al.* solarius: an R interface to SOLAR for variance component analysis in pedigrees. *Bioinformatics* **32**, 1901-2 (2016).
97. McLean, C.Y. *et al.* GREAT improves functional interpretation of cis-regulatory regions. *Nat Biotechnol* **28**(2010).
98. Tacutu, R. *et al.* Human Ageing Genomic Resources: new and updated databases. *Nucleic Acids Res* **46**, D1083-D1090 (2018).
99. Chen, E.Y. *et al.* Enrichr: interactive and collaborative HTML5 gene list enrichment analysis tool. *BMC Bioinformatics* **14**, 128 (2013).
100. Kuleshov, M.V. *et al.* Enrichr: a comprehensive gene set enrichment analysis web server 2016 update. *Nucleic Acids Res* **44**, W90-7 (2016).
101. Arneson, A. *et al.* A mammalian methylation array for profiling methylation levels at conserved sequences. *Nature Communications* **13**, 783 (2022).
102. Horvath, S. *et al.* An epigenetic clock analysis of race/ethnicity, sex, and coronary heart disease. *Genome Biol* **17**, 171 (2016).
103. Hannum, G. *et al.* Genome-wide methylation profiles reveal quantitative views of human aging rates. *Mol Cell* **49**, 359-67 (2013).
104. Levine, M.E. *et al.* An epigenetic biomarker of aging for lifespan and healthspan. *Aging (Albany NY)* (2018).
105. Lyon, M. *et al.* The variant call format provides efficient and robust storage of GWAS summary statistics. *bioRxiv*, 2020.05.29.115824 (2020).
106. Segre, A.V., Groop, L., Mootha, V.K., Daly, M.J. & Altshuler, D. Common inherited variation in mitochondrial genes is not enriched for associations with type 2 diabetes or related glycemic traits. *PLoS Genet* **6**(2010).
107. Elsworth, B. *et al.* The MRC IEU OpenGWAS data infrastructure. *bioRxiv*, 2020.08.10.244293 (2020).
108. Hemani, G. *et al.* The MR-Base platform supports systematic causal inference across the human phenome. *Elife* **7**(2018).
109. Bleda, M. *et al.* CellBase, a comprehensive collection of RESTful web services for retrieving relevant biological information from heterogeneous sources. *Nucleic Acids Res* **40**, W609-14 (2012).
110. Davis, C.A. *et al.* The Encyclopedia of DNA elements (ENCODE): data portal update. *Nucleic Acids Research* **46**, D794-D801 (2018).
111. Itokawa, N. *et al.* Epigenetic traits inscribed in chromatin accessibility in aged hematopoietic stem cells. *Nature Communications* **13**, 2691 (2022).
112. Stuart, T., Srivastava, A., Madad, S., Lareau, C.A. & Satija, R. Single-cell chromatin state analysis with Signac. *Nat Methods* **18**, 1333-1341 (2021).
113. Lawrence, M. *et al.* Software for computing and annotating genomic ranges. *PLoS Comput Biol* **9**, e1003118 (2013).
114. Flurkey, K., Papaconstantinou, J., Miller, R.A. & Harrison, D.E. Lifespan extension and delayed immune and collagen aging in mutant mice with defects in growth hormone production. *Proceedings of the National Academy of Sciences* **98**, 6736-6741 (2001).
115. Dominick, G. *et al.* Regulation of mTOR activity in Snell dwarf and GH receptor gene-disrupted mice. *Endocrinology* **156**, 565-75 (2015).

116. Coschigano, K.T., Clemmons, D., Bellush, L.L. & Kopchick, J.J. Assessment of growth parameters and life span of GHR/BP gene-disrupted mice. *Endocrinology* **141**, 2608-13 (2000).
117. Coschigano, K.T. *et al.* Deletion, but not antagonism, of the mouse growth hormone receptor results in severely decreased body weights, insulin, and insulin-like growth factor I levels and increased life span. *Endocrinology* **144**, 3799-810 (2003).
118. Peters, M.J. *et al.* The transcriptional landscape of age in human peripheral blood. *Nat Commun* **6**, 8570 (2015).
119. List, E.O. *et al.* Liver-specific GH receptor gene-disrupted (LiGHRKO) mice have decreased endocrine IGF-I, increased local IGF-I, and altered body size, body composition, and adipokine profiles. *Endocrinology* **155**, 1793-805 (2014).
120. Acosta-Rodríguez, V. *et al.* Circadian alignment of early onset caloric restriction promotes longevity in male C57BL/6J mice. *Science* **376**, 1192-1202 (2022).
