# Supplementary material for: Universal DNA methylation age across mammalian tissues
Source: Nat Aging. 2023 Aug 10;3(9):1144–66. doi: 10.1038/s43587-023-00462-6 (PMC10501909; doi:10.1038/s43587-023-00462-6)
Supplement: Supplementary file 2 — Reporting Summary [file 43587_2023_462_MOESM2_ESM.pdf]

nature portfolio

Corresponding author(s): Steve Horvath
Last updated by author(s): 6/01/2023

# Reporting Summary

Nature Portfolio wishes to improve the reproducibility of the work that we publish. This form provides structure for consistency and transparency in reporting. For further information on Nature Portfolio policies, see our Editorial Policies and the Editorial Policy Checklist.

## Statistics

For all statistical analyses, confirm that the following items are present in the figure legend, table legend, main text, or Methods section.

| n/a | Confirmed | |
|---|---|---|
| ☐ | ☒ | The exact sample size (*n*) for each experimental group/condition, given as a discrete number and unit of measurement |
| ☐ | ☒ | A statement on whether measurements were taken from distinct samples or whether the same sample was measured repeatedly |
| ☐ | ☒ | The statistical test(s) used AND whether they are one- or two-sided<br>*Only common tests should be described solely by name; describe more complex techniques in the Methods section.* |
| ☐ | ☒ | A description of all covariates tested |
| ☐ | ☒ | A description of any assumptions or corrections, such as tests of normality and adjustment for multiple comparisons |
| ☐ | ☒ | A full description of the statistical parameters including central tendency (e.g. means) or other basic estimates (e.g. regression coefficient) AND variation (e.g. standard deviation) or associated estimates of uncertainty (e.g. confidence intervals) |
| ☐ | ☐ | For null hypothesis testing, the test statistic (e.g. *F*, *t*, *r*) with confidence intervals, effect sizes, degrees of freedom and *P* value noted<br>*Give P values as exact values whenever suitable.* |
| ☒ | ☐ | For Bayesian analysis, information on the choice of priors and Markov chain Monte Carlo settings |
| ☒ | ☐ | For hierarchical and complex designs, identification of the appropriate level for tests and full reporting of outcomes |
| ☐ | ☒ | Estimates of effect sizes (e.g. Cohen's *d*, Pearson's *r*), indicating how they were calculated |

*Our web collection on statistics for biologists contains articles on many of the points above.*

## Software and code

Policy information about availability of computer code

| | |
|---|---|
| Data collection | Collections of Mammalian samples are described in Supplementary Information Note 1. Mammalian samples are maintained as Excel spread sheets or Rdata generated in R. |
| Data analysis | Matlab_2017b: MAGENTA analysis for yielding gene-level p values based on GWAS SNP associations in EWAS-GWAS analysis<br>Python 3.10.3: package anndata 0.8.0 for managing human single cell ATAC (sc-ATAC) array data<br>R_4.0.2: Programming language for statistical computing<br>R_sesame_1.3.0: Normalize Illumina Infinium DNA methylation array data<br>R_glmnet_4.1-7 Fit penalized generalized linear models<br>R_WGCNA_1.69: Weighted correlation network analysis for analysis and graphics.<br>R_rGREAT_1.22.0: Enrichment analysis using Genomic Regions Enrichment of Annotations Tool<br>R_gwasvcf_0.1.1: Manage GWAS summary datasets in VCF format<br>R_Signac: Perform quality controls (R_Signac/CreateChromatinAssay function) and data management for mouse sc-ATAC analysis<br>R_GenomicRanges: Overlap locations between peak calling locations from sc-ATAC and probe locations in our Mammalian array.<br>R_ggplot2, R_ComplexHeatmap_2.6.2 and R_gmirror: for figures including box plots, heat maps, and Manhattan plots. |

For manuscripts utilizing custom algorithms or software that are central to the research but not yet described in published literature, software must be made available to editors and reviewers. We strongly encourage code deposition in a community repository (e.g. GitHub). See the Nature Portfolio guidelines for submitting code & software for further information.

## Data

Policy information about availability of data

All manuscripts must include a data availability statement. This statement should provide the following information, where applicable:

- Accession codes, unique identifiers, or web links for publicly available datasets
- A description of any restrictions on data availability
- For clinical datasets or third party data, please ensure that the statement adheres to our policy

The data for all species from the Mammalian Methylation Consortium can be downloaded from Gene Expression Omnibus (GEO) using the accession number GSE223748. To facilitate comparative analyses across species, the consortium applied a single measurement platform (the mammalian methylation array, GPL28271) to n=15,216 DNA samples derived from 70 tissue types of 348 different mammalian species (331 eutherian-, 15 marsupial-, and 2 monotreme species). The 11,754 samples used for training our universal clocks are part of the samples, which are available for age information.
Subset of the data can be accessed from multiple online locations. First, the data are available from https://clockfoundation.org/MammalianMethylationConsortium. Second the data can be downloaded from GEO using the following accession numbers: GSE174758, GSE184211, GSE184213, GSE184215, GSE184216, GSE184218, GSE184220, GSE184221, GSE184224, GSE190660, GSE190661, GSE190662, GSE190663, GSE190664, GSE174544, GSE190665, GSE174767, GSE184222, GSE184223, GSE174777, GSE174778, GSE173330, GSE164127, GSE147002, GSE147003, GSE147004. Additional details can be found in Supplementary information note 2. The mammalian methylation array is available through the non-profit Epigenetic Clock Development Foundation (https://clockfoundation.org/).

## Human research participants

Policy information about studies involving human research participants and Sex and Gender in Research.

**Reporting on sex and gender**

The Sex/Gender variable in the Framingham Heart Study (FHS) Cohort,https://www.framinghamheartstudy.org/index.php, is based on self-report. The FHS data are available in dbGaP (accession number: phs000363.v16.p10 and phs000724.v2.p9).

The Women's Health Initiative (WHI) is a national study that enrolled postmenopausal women aged 50-79 years into the clinical trials (CT) or observational study (OS) cohorts between 1993 and 1998.

**Population characteristics**

We applied our universal Clocks 2 and 3 on 4,651 individuals from (a) the Framingham Heart Study (FHS) offspring cohort (n=2,544 Caucasians, 54% females)and (b) Women's Health Initiative cohort (WHI, n=2107, 100% woman, Supplementary Information, note 4). Methylation levels were profiled in blood samples using Illumina 450k arrays. The FHS cohort had a mean (SD) age of 66.3 (8.9) years at blood draw, with 330 deaths during an average follow-up of 7.8 years. The WHI cohort, which enrolled postmenopausal women aged 50-79 years, consisted of three ethnic groups: 47% of European ancestry (Caucasians), 32% African Americans, and 20% of Hispanic ancestry. These groups exhibited similar age distributions, with a mean (SD) age of 65.4 (7.1) years, and a mean (SD) follow-up time of 16.9 (4.6) years. During the follow-up, 765 women died.

**Recruitment**

We did not recruit any human beings in this study. Rather, we used existing data. The FHS cohort is a large-scale longitudinal study initiated in 1948, originally designed to explore the common factors and characteristics contributing to cardiovascular disease (CVD) (https://www.framinghamheartstudy.org/index.php). This study initially enrolled participants from the town of Framingham, Massachusetts, who were asymptomatic for overt CVD, heart attack, or stroke at the time of enrollment. In 1971, the FHS Offspring Cohort was established to encompass a second generation of participants, specifically the adult children and their spouses of the original cohort (n=5124), for similar examinations. Participants from the FHS Offspring Cohort were included in our study if they had attended the 8th examination cycle and consented to the use of their molecular data for research purposes. We utilized data from 2,544 participants with available DNA methylation profiles (measured at exam 8), obtained from the group providing Health/Medical/Biomedical consent (IRB, MDS). The FHS data can be accessed through the db GaP (accession numbers: phs000363.v16.p10 and phs000724.v2.p9). The Women's Health Initiative, a national landmark study, recruited postmenopausal women aged between 50-79 years into the clinical trials (CT) or observational study (OS) cohorts between 1993 and 199866,67. We incorporated data from 2,017 WHI participants from "Broad Agency Award 23" (WHI BA23) which had available phenotype and DNA methylation array data. WHI BA23 is dedicated to the identification of miRNA and genomic biomarkers of coronary heart disease (CHD), with an aim to integrate these biomarkers into diagnostic and prognostic predictors of CHD and associated phenotypes. This study encompasses three WHI sub-cohorts, namely GARNET, WHIMS, and SHARe.

**Ethics oversight**

The Framingham Heart Study is funded by National Institutes of Health contract N01-HC-25195 and HHSN268201500001I. The laboratory work for this investigation was funded by the Division of Intramural Research, National Heart, Lung, and Blood Institute, National Institutes of Health. The analytical component of this project was funded by the Division of Intramural Research, National Heart, Lung, and Blood Institute, and the Center for Information Technology, National Institutes of Health, Bethesda, MD.

The Women's Health Initiative program is funded by the National Heart, Lung, and Blood Institute, National Institutes of Health, U.S. Department of Health and Human Services through contracts HHSN268201600018C, HHSN268201600001C, HHSN268201600002C, HHSN268201600003C, and HHSN268201600004C. The authors thank the WHI investigators and staff for their dedication, and the study participants for making the program possible. A full listing of WHI investigators can be found at: http://www.whi.org/researchers/Documents%20%20Write%20a%20Paper/WHI%20Investigator%20Long%20List.pdf. The views expressed in this manuscript are those of the authors and do not necessarily represent the views of funding bodies such as the National Heart, Lung, and Blood Institute; the National Institutes of Health; or the U.S. Department of Health and Human Services.

Note that full information on the approval of the study protocol must also be provided in the manuscript.

# Field-specific reporting

Please select the one below that is the best fit for your research. If you are not sure, read the appropriate sections before making your selection.

☐ Life sciences ☐ Behavioural & social sciences ☒ Ecological, evolutionary & environmental sciences

For a reference copy of the document with all sections, see nature.com/documents/nr-reporting-summary-flat.pdf

# Ecological, evolutionary & environmental sciences study design

All studies must disclose on these points even when the disclosure is negative.

**Study description**

Observational data based on existing samples stored in freezers. We generated 11,754 methylation arrays from over 57 tissue-types derived from 185 mammalian species. We aimed to profile animals from the entire age range: from very young to very old. Roughly uniform distribution. We only analyzed tissues from animals whose ages were known with 90% confidence.

**Research sample**

I. We employed a custom methylation array (HorvathMammalMethylChip40) that profiles methylation levels of 36k CpGs with flanking DNA sequences that are highly-conserved across the mammalian class.
II. We obtained such profiles from 11,754 samples from 59 tissue types, derived from 185 mammalian species, representing 19 taxonomic orders and ranging in age from prenatal to 139 years old (bowhead whale).

III. The tissue samples are described in the Supplement and related citations as listed in Supplementary Information, Note 1.
IV. To enhance the reproducibility of our findings we include our updated version of the animal age (anAge) database, which is reported in the supplementary data.
V. Below are the list of ipapers that describe specific species:

1. Horvath, S. et al. Pan-primate DNA methylation clocks. bioRxiv, 2020.11.29.402891 (2021).
2. Horvath, S. et al. Epigenetic clock and methylation studies in the rhesus macaque. GeroScience (2021).
3. Jasinska, A.J. et al. Epigenetic clock and methylation studies in vervet monkeys. GeroScience (2021).
4. Horvath, S. et al. DNA methylation age analysis of rapamycin in common marmosets. GeroScience (2021).
5. Schlabritz-Loutsevitch, N.E. et al. Metabolic adjustments to moderate maternal nutrient restriction. British journal of nutrition 98, 276-284 (2007).
6. Kavitha, J.V. et al. Down-regulation of placental mTOR, insulin/IGF-I signaling, and nutrient transporters in response to maternal nutrient restriction in the baboon. FASEB journal : official publication of the Federation of American Societies for Experimental Biology 28, 1294-1305 (2014).
7. Schlabritz-Loutsevitch, N.E. et al. Development of a system for individual feeding of baboons maintained in an outdoor group social environment. Journal of Medical Primatology 33, 117-126 (2004).
8. Zehr, S.M. et al. Life history profiles for 27 strepsirrhine primate taxa generated using captive data from the Duke Lemur Center. Scientific Data 1, 140019 (2014).
9. Morgello, S. et al. The National NeuroAIDS Tissue Consortium: a new paradigm in brain banking with an emphasis on infectious disease. Neuropathol Appl Neurobiol 27, 326-35. (2001).
10. Horvath, S. et al. HIV, pathology and epigenetic age acceleration in different human tissues. Geroscience (2022).
11. Horvath, S. et al. Perinatally acquired HIV infection accelerates epigenetic aging in South African adolescents. AIDS (London, England) 32, 1465-1474 (2018).
12. Horvath, S. & Ritz, B.R. Increased epigenetic age and granulocyte counts in the blood of Parkinson's disease patients. Aging (Albany NY) 7, 1130-42 (2015).
13. Kabacik, S., Horvath, S., Cohen, H. & Raj, K. Epigenetic ageing is distinct from senescence-mediated ageing and is not prevented by telomerase expression. Aging (Albany NY) 10, 2800-2815 (2018).
14. Ross, C.N. et al. The development of a specific pathogen free (SPF) barrier colony of marmosets (Callithrix jacchus) for aging research. Aging (Albany NY) 9, 2544 (2017).
15. Sailer, L.L. et al. Pair bonding slows epigenetic aging and alters methylation in brains of prairie voles. bioRxiv, 2020.09.25.313775 (2020).
16. Ophir, A.G. Navigating Monogamy: Nonapeptide Sensitivity in a Memory Neural Circuit May Shape Social Behavior and Mating Decisions. Frontiers in Neuroscience 11(2017).
17. Horvath, S. et al. Methylation studies in Peromyscus: aging, altitude adaptation, and monogamy. GeroScience 44, 447-461 (2022).
18. Horvath, S. et al. DNA methylation aging and transcriptomic studies in horses. Nat Commun 13, 40 (2022).
19. Burns, E.N. et al. Generation of an equine biobank to be used for Functional Annotation of Animal Genomes project. Animal genetics 49, 564-570 (2018).
20. Horvath, S. et al. DNA methylation clocks tick in naked mole rats but queens age more slowly than nonbreeders. Nature Aging 2, 46-59 (2022).
21. Ke, Z., Vaidya, A., Ascher, J., Seluanov, A. & Gorbunova, V. Novel husbandry techniques support survival of naked mole rat

(Heterocephalus glaber) pups. J Am Assoc Lab Anim Sci 53, 89-91 (2014).
22. Tan, L. et al. Naked Mole Rat Cells Have a Stable Epigenome that Resists iPSC Reprogramming. Stem cell reports 9, 1721-1734 (2017).
23. Sugrue, V.J. et al. Castration delays epigenetic aging and feminizes DNA methylation at androgen-regulated loci. eLife 10, e64932 (2021).
24. Schachtschneider, K.M. et al. Epigenetic clock and DNA methylation analysis of porcine models of aging and obesity. GeroScience (2021).
25. Robeck, T.R. et al. Multi-Tissue Methylation Clocks for Age and Sex Estimation in the Common Bottlenose Dolphin. Frontiers in Marine Science 8(2021).
26. Robeck, T.R. et al. Multi-species and multi-tissue methylation clocks for age estimation in toothed whales and dolphins. Commun Biol 4, 642 (2021).
27. Bors, E.K. et al. An epigenetic clock to estimate the age of living beluga whales. Evolutionary Applications (2020).
28. Raj, K. et al. Epigenetic clock and methylation studies in cats. GeroScience (2021).
29. Prado, N.A. et al. Epigenetic clock and methylation studies in elephants. Aging Cell 20, e13414 (2021).
30. Pinho, G.M. et al. Hibernation slows epigenetic ageing in yellow-bellied marmots. Nature Ecology & Evolution 6, 418-426 (2022).
31. Lemaître, J.-F. et al. DNA methylation as a tool to explore ageing in wild roe deer populations. Molecular Ecology Resources n/a(2021).
32. Larison, B. et al. Epigenetic models developed for plains zebras predict age in domestic horses and endangered equids. Communications Biology 4, 1412 (2021).
33. Harley, E.H., Knight, M.H., Lardner, C., Wooding, B. & Gregor, M. The Quagga project: progress over 20 years of selective breeding. African Journal of Wildlife Research 39, 155-163 (2009).
34. Horvath, S. et al. Reversing age: dual species measurement of epigenetic age with a single clock. bioRxiv, 2020.05.07.082917 (2020).
35. Horvath, S. et al. Epigenetic clock and methylation studies in dogs. PNAS In Press(2022).
36. Plassais, J. et al. Whole genome sequencing of canids reveals genomic regions under selection and variants influencing morphology. Nature Communications 10, 1489 (2019).
37. Plassais, J. et al. Analysis of large versus small dogs reveals three genes on the canine X chromosome associated with body weight, muscling and back fat thickness. PLOS Genetics 13, e1006661 (2017).
38. TheAmericanKennelClub. The Complete Dog Book: 20th Edition, (Howell Book House, New York, NY, 2006).
39. Wilcox, B. & Walkowicz, C. The Atlas of Dog Breeds of the World, (T.F.H. Publications, 1995).
40. Wilkinson, G.S. et al. DNA methylation predicts age and provides insight into exceptional longevity of bats. Nature Communications 12, 1615 (2021).
41. Kordowitzki, P. et al. Epigenetic clock and methylation study of oocytes from a bovine model of reproductive aging. Aging Cell 20, e13349 (2021).
42. Mozhui, K. et al. Genetic loci and metabolic states associated with murine epigenetic aging. eLife 11, e75244 (2022).
43. Lu, A.T. et al. DNA methylation study of Huntington's disease and motor progression in patients and in animal models. Nature Communications 11, 4529 (2020).
44. Coschigano, K. et al. Deletion, but not antagonism, of the mouse growth hormone receptor results in severely decreased body weights, insulin, and insulin-like growth factor I levels and increased life span. Endocrinology 144, 3799-3810 (2003).
45. Acosta-Rodríguez, V.A., Rijo-Ferreira, F., Green, C.B. & Takahashi, J.S. Importance of circadian timing for aging and longevity. Nature Communications 12, 2862 (2021).
46. Little, T.J. et al. Methylation-Based Age Estimation in a Wild Mouse. bioRxiv, 2020.07.16.203687 (2020).
47. Cossette, M.-L. et al. Differential methylation, epigenetic clocks, and island-mainland divergence in an insectivorous small mammal. bioRxiv, 2022.04.14.488253 (2022).
48. Horvath, S. et al. Epigenetic clock and methylation studies in marsupials: opossums, Tasmanian devils, kangaroos, and wallabies. Geroscience In Press(2022).
49. Hogg, C.J., Lee, A.V. & Hibbard, C.J. Managing a metapopulation: intensive to wild and all the places in between. in Saving the Tasmanian Devil: recovery through science based management 169-182 (CSIRO Publishing Melbourne, 2019).
50. Hogg, C. & Hockley, J. DPIPWE/ZAA husbandry guidelines for Tasmanian devil, Sarcophilus harrisii. Australia: Zoo and Aquarium Association (2013).
51. Sambrook, J. & Russell, D.W. Purification of nucleic acids by extraction with phenol: chloroform. Cold Spring Harbor Protocols 2006, pdb. prot4455 (2006).
52. Villar, D. et al. Enhancer evolution across 20 mammalian species. Cell 160, 554-66 (2015).
53. Berthelot, C., Villar, D., Horvath, J.E., Odom, D.T. & Flicek, P. Complexity and conservation of regulatory landscapes underlie evolutionary resilience of mammalian gene expression. Nat Ecol Evol 2, 152-163 (2018).
54. Roller, M. et al. LINE retrotransposons characterize mammalian tissue-specific and evolutionarily dynamic regulatory regions. Genome Biol 22, 62 (2021).
55. Yan, L. et al. OSAT: a tool for sample-to-batch allocations in genomics experiments. BMC Genomics 13, 689 (2012).
56. Seluanov, A. et al. Hypersensitivity to contact inhibition provides a clue to cancer resistance of naked mole-rat. Proceedings of the National Academy of Sciences 106, 19352-19357 (2009).
57. Seluanov, A. et al. Telomerase activity coevolves with body mass not lifespan. Aging Cell 6, 45-52 (2007).

Sampling strategy

We sampled all mammalian species for which existing tissues were available. These fresh frozen tissue samples were contributed by a large network of investigators from our Mammalian Methylation Consortium.
To guide the quality control (QC) of the study samples, we generated two variables ; the first being a variable indicating the confidence (0 to 100%) in the chronological age estimate of the sample. For example, a low confidence was assigned to samples from wild animals whose ages were estimated based on body length measurements. The epigenetic clocks were trained and evaluated in tissue samples whose confidence exceeded 90% (>=90%). The second quality control variable was an indicator variable (yes/no) that flagged technical outliers or malignant (cancer) tissue. Since we were interested in "normal" aging patterns we excluded tissues from preclinical studies surrounding anti-aging or pro-aging interventions.

Data collection

DNA for methylation profiling was extracted from the tissue samples collected from different species as described in Supplementary Information, Note 1 . After bisulfite conversion and labeling of the DNA, methylation profiles were obtained by hybridizing labeled

| | |
|---|---|
| | DNA to a custom Illumina methylation array (HorvathMammalMethylChip40) and scanning with an Illumina iScan at the UCLA Neuroscience Genomics Core. |
| Timing and spatial scale | The tissue samples were collected over the last 30 years. The data come from many labs all over the world: US, Canada, Europe, Australia, New Zealand, South America. |
| Data exclusions | We excluded about 1900 samples that had insufficient DNA to provide reliable methylation values, low confidence in the chronological age estimate or unknown age. We discovered that samples with concentrations below 6 ng/µl could not be accurately scored at all sites on the array. |
| Reproducibility | 1. We used calibration data (synthetic DNA) to evaluate the accuracy of the methylation measurements (A mammalian methylation array for profiling methylation levels at conserved sequences by A. Arneson 2021 Nat Comm)<br>2. We performed EWAS meta-analysis of age using Stouffer's method estimates from Metal algorithm. In addition, we verified the Stouffer's statistics in our in-house R code.<br>3. The universal clocks were established via elastic net regression models. To assess the accuracies of our clocks, we used 3 approaches: leave-one-fraction-out (LOFO), leave one-species-out (LOSO) cross validation, and data splitting.<br>In LOFO, we randomly split the entire dataset into 10 fractions each of which had the same distribution in species and tissue types. Each penalized regression model was trained in 9 fractions but evaluated in the 10th left out fraction. After circling through the 10 fractions, we arrived at LOFO predictions which were subsequently related to the actual values.<br>The LOSO cross validation approach trained each model on all but one species. The left out species was used a test set. The LOSO approach was used to assess how well the penalized regression models generalize to species that were not part of the training data. To ensure unbiased estimates of accuracy, all aspects of the model fitting were only conducted in the training data in both LOFO and LOSO analysis.<br>4. The reported EWAS p values are significant even after using the most stringent multiple comparison correction (Bonferroni)=0.05/37K based on 37K CpGs on the mammalian array.<br>5. In GREAT enrichment analysis, we performed two different sensitivity analyses that were inspired by our GREAT enrichment analysis of the top 1 thousand age related CpGs (EWAS of age). The results are listed in Supplementary Info, Note 2. Our first sensitivity analysis involved a random set of 1000 CpG mammalian CpGs. Second, we evaluated the enrichment of the top 1087 most highly conserved CpGs across 158 mammalian genomes. This sensitivity analysis addresses the concern that highly conserved CpGs could have an increased chance of correlating strongly with chronological age or, conversely, non-conserved (noise) CpGs are expected to have no signal for age and will therefore not be selected in an EWAS of age.<br>6. In single cell ATAC-seq analysis, to confirm enrichment for the hyper methylated sites showing decrease of chromatin accessibility with age, we randomly selected 1000 sets of 17 ATAC peaks and compared the mean correlation with age of the selected regions to the 1000 sampled sets of regions. |
| Randomization | Not applicable since this is an observational study. |
| Blinding | Blinding was not relevant to our study, because this is an observational study and all available data were used |

Did the study involve field work? ☐ Yes ☒ No

# Reporting for specific materials, systems and methods

We require information from authors about some types of materials, experimental systems and methods used in many studies. Here, indicate whether each material, system or method listed is relevant to your study. If you are not sure if a list item applies to your research, read the appropriate section before selecting a response.

## Materials & experimental systems

| n/a | Involved in the study |
|---|---|
| ☒ | ☐ Antibodies |
| ☒ | ☐ Eukaryotic cell lines |
| ☒ | ☐ Palaeontology and archaeology |
| ☐ | ☒ Animals and other organisms |
| ☒ | ☐ Clinical data |
| ☒ | ☐ Dual use research of concern |

## Methods

| n/a | Involved in the study |
|---|---|
| ☒ | ☐ ChIP-seq |
| ☒ | ☐ Flow cytometry |
| ☒ | ☐ MRI-based neuroimaging |

# Animals and other research organisms

Policy information about studies involving animals; ARRIVE guidelines recommended for reporting animal research, and Sex and Gender in Research

| | |
|---|---|
| Laboratory animals | Details in Supplementary Data S1.2. This study leveraged existing tissue samples or data that had been collected as part of other studies. We profiled tissues from lab animals or animals kept in captivity for research. This includes mouse, rat, opossum, naked mole rat, deer mouse colonies, rhesus macaque, marmosets, vervet monkey. Companion pets: dogs, cats. Agricultural animals: horses, pigs, sheep. |

| | |
|---|---|
| Wild animals | Details in Supplementary Data S1.2. Some samples from zoo animals (e.g. elephants). Samples from zoo-based animals were opportunistically collected and banked during routine health exams. This study also includes samples from wild animals, which were collected in the field: bats, deer. |
| Reporting on sex | As the contributors are engaged in long-term field studies, care was taken to minimize disturbance during all the sample collections for different species. The details are described in species-specific papers. |
| Field-collected samples | As the contributors are engaged in long-term field studies, care was taken to minimize disturbance during all the sample collections for different species. The details are described in species-specific papers. |
| Ethics oversight | Institutional animal care and use protocols, or equivalent information from non-US contributors, is provided in Supplementary Information describing the data and also in the underlying species-specific papers published by Mammalian Methylation Consortium. Non university organizations are certified either by the Associated Zoos and Aquariums (Lubee Bat Conservancy) or by the Global Federation of Animal Sanctuaries (Bat World Sanctuary) or Elephant Taxon Advisory Group and Species Survival Plan. Details in appendix. |

Note that full information on the approval of the study protocol must also be provided in the manuscript.

# Ethics Oversight for Mammalian Methylation Consortium Studies

**M1. Primates [1]**
**Ethics**

This research complied with all relevant ethical regulations overseen by seven ethics review boards. The human skin samples were acquired with informed consent prior to collection of human skin samples with approved by the Oxford Research Ethics Committee in the UK; reference 10/H0605/1. Participants were not compensated. The secondary use of the other de-identified/coded human tissue samples (blood, postmortem tissues) is not interpreted as human subjects research under U.S. Department of Health & Human Services 45 CFR 46. Therefore, the need to obtain written, informed consent from human study participants was waived (secondary use of de-identified tissues). Human samples were covered by University of California Los Angeles IRB#18-000315. All procedures related to non-human primates were approved by different committees: baboons (UTHSCSA Animal Care and Use Committee), strepsirrhini (Duke Institutional Animal Care and Use Committee and the DLC Research Committee), rhesus macaques (Animal Care and Use Committee of the NIA Intramural Program) [2], vervet monkey (UCLA and VA Institutional Animal Care and Use Committees) [3], marmosets (IACUC of UTHSA) [4].

**M2. Prairie voles [5]**
**Ethics**

All experimental procedures were conducted and approved by the Institutional Animal Care and Use Committee (IACUC) of Cornell University (2013-0102) and were in accordance with the guidelines set forth by the National Institutes of Health.

**M3. Horses** [6]
**<u>Ethics</u>**

This collection protocol was approved by the UC Davis Institutional Animal Care and Use Committee (Protocol#19037). All collection protocols were approved by the UC Davis Institutional Animal Care and Use Committee (Protocols #20751 and 21455, respectively).

**M4. Naked mole-rat** [7]
The NMR tissue samples were provided by two different labs: (i) Vera Gorbunova and Andrei Seluanov from the University of Rochester and (ii) Chris Faulkes from the Queen Mary, University of London.

**<u>Ethics for (i)</u>**

All animal experiments were approved and performed in accordance with guidelines set up by the University of Rochester Committee on Animal Resources with protocol number 2009-054 (naked mole-rat).

**<u>Ethics for (ii)</u>**

Naked mole-rats were maintained in the Biological Services Unit at Queen Mary University of London in accordance with UK Government Animal Testing and Research Guidance.

**M5. Sheep** [8]
Sheep DNA samples for this study were derived from two distinct tissues from two strains: ear tissue from New Zealand Merino, and blood from South Australian Merino.

**<u>Ethics for ear samples</u>**

Ear tissue was obtained from females and both intact and castrated male Merino sheep during routine on-farm ear tagging procedures in Central Otago, New Zealand. As a small piece of tissue is removed during the ear tagging process that is usually discarded

by the farmer, we were able to source tissue and record the year of birth without altering animal experience, in accordance with the New Zealand Animal Welfare Act (1999) and the National Animal Ethics Advisory Committee (NAEAC) Occasional Paper No 2 [26].

**Ethics for blood samples**

All protocols involving OVT73 sheep were approved by the Primary Industries and Regions South Australia (PIRSA, Approval number 19/02) Animal Ethics Committee with oversight from the University of Auckland Animal Ethics Committee.

### M6. Pig [9]

**Ethics**

All animal procedures were approved by the University of Illinois and University of Wisconsin Institutional Animal Care and Use Committee, and all animals received humane care according to the criteria outlined in the Guide for the Care and Use of Laboratory Animals.

### M7. Odontocete species [10-12]

**Ethics**

The study was authorized by the management of each institution and was reviewed by their respective zoo research and animal use committees.

### M8. Beluga whales [12]

**Ethics**

Skin tissue samples were collected from carcasses of beluga whales that were beach-cast, stranded dead, or taken during subsistence hunting from 1992 to 2015 in Cook Inlet, Alaska, USA (NMFS Research Permit 932-1905-00/MA-009526 through the Marine Mammal Health and Stranding Response Program).

**M9. Killer whales and bowhead whales**
**Ethics**

For bowhead (*Balaena mysticetus*) subsistence hunts, indigenous hunters had the authorization to conduct hunts and collected samples on behalf of Fisheries and Oceans Canada. Bowhead whale biopsy samples were collected in 2019 under Fisheries and Oceans Canada (DFO) license to Fish for Scientific Purposes (LFSP) S-19/20-1007-NU and Animal Care approval (AUP) FWI-ACC-2019-14. Skin samples from eastern North Pacific killer whales (*Orcinus orca*) were collected as previously described (Ford et al. 2018b) under NMFS General Authorization No. 781–1725, and scientific research permits 781-1824-01, 16163, 532- 1822-00, 532– 1822, 10045, 18786-03, 545-1488, 545-1761, and 15616.

**M10. Humpback whales**
**Ethics**

Skin samples were collected by the Center for Coastal Studies under research permits issued by the U.S., National Marine Fisheries Service (21485, 16325, 20465, 14245, 633-1483, 633-1778, 932-1905), the Canadian Department of Fisheries and Oceans and IACUC #NWAK-18-02.

**M11. Cats** [13]
**Ethics**

Sample collection was approved by the Clinical Research Ethical Review Board of the RVC (URN: 2019 1947-2).

**M12. Elephants** [14]
**Ethics**

This study was authorized by the management of each participating zoo and, where applicable, was reviewed and approved by zoo research committees. In addition, the

study received IACUC approval (#18-29) at the NZP; and endorsement from the elephant Taxon Advisory Group and Species Survival Plan.

**M13. Yellow-bellied marmots** [15]

**Ethics**

Data and samples were collected under the UCLA Institutional Animal Care and Use protocol (2001-191-01, renewed annually) and with permission from the Colorado Parks and Wildlife (TR917, renewed annually).

**M14. Roe deer** [16]

**Ethics**

The protocol of capture and blood sampling under the authority of the Office Français de la Biodiversité (OFB) was approved by the Director of Food, Agriculture and Forest (Prefectoral order 2009–14 from Paris). The land manager of both sites, the Office National des Forêts (ONF), permitted the study of the populations (Partnership Convention ONCFS-ONF dated 2005-12-23). All experiments were performed in accordance with guidelines and regulations of the Ethical Committee of Lyon 1 University (project DR2014-09, June 5, 2014)

**M15. Zebras** [17]

**Ethics**

Plains zebra samples were collected under a protocol approved by the Research Safety and Animal Welfare Administration, University of California Los Angeles: ARC # 2009-090-31, originally approved in 2009.

**M16 Rat** [18]

The rat tissues came from 4 different labs across three countries:(i) India: Nugenics Research in collaboration with School of Pharmacy SVKM's NMIMS University (K. Singh), (ii) United States: University of Tennessee Health Science Center (H. Chen) and

Medical College of Wisconsin (L.C. Solberg Woods), and (iii) Argentina: University of La Plata (R. Goya).

**Ethics for (i)**

The experimental protocols received approval from the Institutional Animal Ethics Committee under two distinct approval numbers. The first is CPCSEA/IAEC/P-75/2018, and the second is CPCSEA/IAEC/P-6/2018. The second approval was granted in accordance with the norms of the Committee for the Purpose of Control and Supervision of Experiments on Animals (CPCSEA), Government of India, complying with the standard guidelines for handling experimental animals. It specifically pertains to the use of male Sprague Dawley rats, aged 8 weeks (200–250 g) and 20 months (400-450g), procured from the National Institute of Bioscience, Pune, India.

**Ethics for (ii)**

All procedures were approved by the Institutional Animal Care and Use Committee of the University of Tennessee Health Science Center or the Medical College of Wisconsin and followed the NIH Guide for the Care and Use of Laboratory Animals.

**Ethics for (iii)**

All experiments with animals were performed in accordance with the Animal Welfare Guidelines of NIH (INIBIOLP’s Animal Welfare Assurance No A5647-01) and approved by our Institutional IACUC (Protocol # P05-02-2017). Ethics committee approval number - CPCSEA/IAEC/P-6/2018.

**M17. Dog [19]**

For this study, DNA samples were collected from a total of 742 blood samples taken from dogs across 93 different breeds. These samples were generously provided by

researchers at the National Human Genome Research Institute (NHGRI). Unfortunately, we did not have access to individual weight data for these dogs.

**Ethics**

The collection of these samples was conducted in compliance with ethical guidelines and was officially approved by the Animal Care and Use Committee of the Intramural Program of NHGRI at the National Institutes of Health (Protocol #8329254).

**M18. Bats [20]**

**Ethics**

The study was approved by the University of Maryland Institutional Animal Care and Use Committee (FR-APR-18-16).

**M19. Cattle [21]**

**Ethics**

All animal procedures were carried out in accordance with the relevant guidelines at each institution. Specifically, procedures related to sample collection in Poland followed the EU Directive of the European Parliament and the Council on the protection of animals used for scientific purposes (22 September 2010; No 2010/63/EU), Polish Parliament Act on Animal Protection (21 August 1997, Dz.U. 1997 nr 111 poz. 724) with further novelization - Polish Parliament Act on the protection of animals used for scientific or educational purposes (15 January 2015, Dz.U. 2015 poz. 266). Blood and oocyte collection were approved by the Local Ethics Committee for Experiments on Animals, University of Warmia and Mazury in Olsztyn, Poland (Agreement No. LKE.065.27.2019). For animal procedures in the USA, approval from the University of Nebraska Institutional Animal Care and Use Committee was obtained (approval number is 1560).

**M20. Mouse data** [22]

The mouse data were sourced from various institutions or studies, as outlined below: (i) UCLA Lab. Animal breeding and husbandry [23], (ii) BXD mice [22] from University of Tennessee Health Science Center, (iii) Growth hormone receptor knockout from the University of Michigan, (iv) Calorie restricted mice from the University of Texas Southwestern Medical Center [24], (v) South African species, (vi) Apodemus mice[25] , and (vii) Spiny mouse.

**Ethics for (i)**

All mice were maintained and bred under standard conditions consistent with National Institutes of Health guidelines and approved by the University of California, Los Angeles Institutional Animal Care and Use Committees.

**Ethics for (ii)**

All animal procedures were in accordance with the protocol approved by the Institutional Animal Care and Use Committee (IACUC) at the University of Tennessee Health Science Center.

Ethics for (iv)

The Institutional Animal Care and Use Committee (IACUC) of the University of Texas Southwestern Medical Center approved the animal protocol (APN 2015-100925), which has been subsequently renewed every 3 years (2018 and 2021).

**Ethics for (v)**

The Animal Use and Care Committee of the University of Pretoria evaluated and approved the experimental protocol and collection of all samples (ethics clearance number: NAS022/2021, NAS209/2021, NAS021/2020), with DAFF section 20 approval (SDAH-Epi-21051907211, SDAH-Epi-12/11/1/1/8 (2002 LH), SDAH-Epi-20072707050).

In addition, permission to capture the various species was obtained from all landowners, and a collecting permit was obtained from the relevant nature conservation authorities (Permit number: Western Cape- CN44-87-13780, CN44-31-2285, Gauteng- CPF6-0124, Kwa-Zulu Natal- OP1545/2021). Necessary TOPS permits were also acquired for threatened species (Permit number: 68103).

**Ethics for (vi)**

All animal work was conducted in accordance with the UK Home Office in compliance with the Animals (Scientific Procedures) Act 1986, was approved by the University of Edinburgh Ethical Review Committee and was carried out under the approved UK Home Office Project License PP4913586.

**Ethics for (vii)**

Animal protocols were approved by the Institutional Animal Care and Use Committee (IACUC) at the University of Kentucky (2019-3254).

**M21. Nova Scotia masked shrews (*Sorex cinereus*) [26]**

**Shrews and small animals from Museum of Biological Diversity at The Ohio State University**

All wild-caught animals were collected and sacrificed in accordance with protocols approved by The Ohio State University IACUC (Institutional Animal Care and Use Committee) under protocol number 2017A00000036. All wild-caught animals were collected with scientific collecting permits issued from Ohio and Washington and according to guidelines established by the American Society of Mammalogy for the use of wild animals in research (Sikes & Animal Care and Use Committee of the American Society of Mammalogists 2016).

**M22. Marsupials and mice [27]**
**<u>Ethics</u>**

These procedures are in accordance with the AVMA Guidelines for the Euthanasia of Animals 2013: https://www.avma.org/KB/Policies/Documents/euthanasia.pdf, and all animal procedures were approved by the UCLA IACUC.

**M23. Mammalian liver samples (Diego Villar Lozano and Duncan Odom)**
**<u>Ethics</u>**

The use of all animals in this study was approved by the Animal Welfare and Ethics Review Board under reference number NRWF-DO-02vs and followed the Cancer Research UK Cambridge Institute guidelines for the use of animals in experimental studies. Tissue samples from humans were obtained from Addenbrooke's Hospital at the University of Cambridge under license number 08-H0308-117, specifically for the study "Liver specific transcriptional regulation."

**M24. Mammalian liver samples from the University of Rochester**
**<u>Ethics</u>**

All experiments were performed according to procedures approved by the University of Rochester Committee on Animal Resources (UCAR), under animal protocol #101939 / UCAR-2017-033. The tissues used in the study were obtained from the Gorbunova and Seluanov tissue bank at the University of Rochester.[28,29]

**Murine anti-aging studies**
**<u>Ethics</u>**

Experiments involving Snell, growth hormone receptor knockout (GHRKO), and liver-specific GHRKO mouse strains (PI: Richard Miller) were conducted at the University of Michigan. These experiments were approved by the University of Michigan's Institutional Animal Care and Use Committee.

**Reference:**


1. Horvath, S. *et al.* Pan-primate DNA methylation clocks. *bioRxiv*, 2020.11.29.402891 (2021).
2. Horvath, S. *et al.* Epigenetic clock and methylation studies in the rhesus macaque. *GeroScience* (2021).
3. Jasinska, A.J. *et al.* Epigenetic clock and methylation studies in vervet monkeys. *GeroScience* (2021).
4. Horvath, S. *et al.* DNA methylation age analysis of rapamycin in common marmosets. *GeroScience* (2021).
5. Sailer, L.L. *et al.* Pair bonding slows epigenetic aging and alters methylation in brains of prairie voles. *bioRxiv*, 2020.09.25.313775 (2020).
6. Horvath, S. *et al.* DNA methylation aging and transcriptomic studies in horses. *Nat Commun* **13**, 40 (2022).
7. Horvath, S. *et al.* DNA methylation clocks tick in naked mole rats but queens age more slowly than nonbreeders. *Nature Aging* **2**, 46-59 (2022).
8. Sugrue, V.J. *et al.* Castration delays epigenetic aging and feminizes DNA methylation at androgen-regulated loci. *eLife* **10**, e64932 (2021).
9. Schachtschneider, K.M. *et al.* Epigenetic clock and DNA methylation analysis of porcine models of aging and obesity. *GeroScience* (2021).
10. Robeck, T.R. *et al.* Multi-Tissue Methylation Clocks for Age and Sex Estimation in the Common Bottlenose Dolphin. *Frontiers in Marine Science* **8**(2021).
11. Robeck, T.R. *et al.* Multi-species and multi-tissue methylation clocks for age estimation in toothed whales and dolphins. *Commun Biol* **4**, 642 (2021).
12. Bors, E.K. *et al.* An epigenetic clock to estimate the age of living beluga whales. *bioRxiv*, 2020.09.28.317610 (2020).
13. Raj, K. *et al.* Epigenetic clock and methylation studies in cats. *GeroScience* (2021).
14. Prado, N.A. *et al.* Epigenetic clock and methylation studies in elephants. *Aging Cell* **20**, e13414 (2021).
15. Pinho, G.M. *et al.* Hibernation slows epigenetic ageing in yellow-bellied marmots. *Nature Ecology & Evolution* **6**, 418-426 (2022).
16. Lemaître, J.-F. *et al.* DNA methylation as a tool to explore ageing in wild roe deer populations. *Molecular Ecology Resources* **n/a**(2021).
17. Larison, B. *et al.* Epigenetic models developed for plains zebras predict age in domestic horses and endangered equids. *Communications Biology* **4**, 1412 (2021).
18. Horvath, S. *et al.* Reversing age: dual species measurement of epigenetic age with a single clock. *bioRxiv*, 2020.05.07.082917 (2020).
19. Horvath, S. *et al.* DNA methylation clocks for dogs and humans. *Proceedings of the National Academy of Sciences* **119**, e2120887119 (2022).
20. Wilkinson, G.S. *et al.* DNA methylation predicts age and provides insight into exceptional longevity of bats. *Nature Communications* **12**, 1615 (2021).
21. Kordowitzki, P. *et al.* Epigenetic clock and methylation study of oocytes from a bovine model of reproductive aging. *Aging Cell* **20**, e13349 (2021).
22. Mozhui, K. *et al.* Genetic loci and metabolic states associated with murine epigenetic aging. *eLife* **11**, e75244 (2022).

23. Lu, A.T. *et al.* DNA methylation study of Huntington's disease and motor progression in patients and in animal models. *Nature communications* **11**, 1-15 (2020).
24. Acosta-Rodríguez, V.A., Rijo-Ferreira, F., Green, C.B. & Takahashi, J.S. Importance of circadian timing for aging and longevity. *Nature Communications* **12**, 2862 (2021).
25. Little, T.J. *et al.* Methylation-Based Age Estimation in a Wild Mouse. *bioRxiv*, 2020.07.16.203687 (2020).
26. Cossette, M.-L. *et al.* Differential methylation, epigenetic clocks, and island-mainland divergence in an insectivorous small mammal. *bioRxiv*, 2022.04.14.488253 (2022).
27. Horvath, S. *et al.* Epigenetic clock and methylation studies in marsupials: opossums, Tasmanian devils, kangaroos, and wallabies. *Geroscience* **In Press**(2022).
28. Seluanov, A. *et al.* Hypersensitivity to contact inhibition provides a clue to cancer resistance of naked mole-rat. *Proceedings of the National Academy of Sciences* **106**, 19352-19357 (2009).
29. Seluanov, A. *et al.* Telomerase activity coevolves with body mass not lifespan. *Aging Cell* **6**, 45-52 (2007).
