# Supplementary material for: Universal DNA methylation age across mammalian tissues
Source: Nat Aging. 2023 Aug 10;3(9):1144–66. doi: 10.1038/s43587-023-00462-6 (PMC10501909; doi:10.1038/s43587-023-00462-6)
Supplement: Supplementary file 4 — Supplementary Code [file 43587_2023_462_MOESM4_ESM.pdf]

## Code availability

The R code below can also be found in our

Github[1] at https://github.com/shorvath/MammalianMethylationConsortium/tree/v2.0.0.

**(1) Universal pan-mammalian clocks.**
The following R code requires one input data (info in the R code) and three csv files that list three universal clocks models in csv format, which can be copied from Data3.1—Data3.3 in Supplementary Data. The info file needs to include ID variable, gestation time in years, age at sexual maturity in years, and maximum lifespan in years. The variables names in the demo R code are SampleID, GestationTimeInYears, averagedMaturity.yrs, and maxAge, respectively. A demonstrated data can be downloaded from our Github[1] at https://github.com/shorvath/MammalianMethylationConsortium/tree/v2.0.0/UniversalPanMammalianClock/ClockParameters. The **R notebook** for clock estimates can be download from here, https://github.com/shorvath/MammalianMethylationConsortium/blob/v2.0.0/UniversalPanMammalianClock/R_code/R_pgm1_threeUniversalClocks.Rmd

**Loglog transformation of Relative Age for clock 2**

Our measure of relative age leverages gestation time and maximum lifespan. We define relative age ($RelativeAge$) and apply the double logarithmic $Loglog$ transformation:

$$RelativeAge = \frac{Age + GestationT}{MaxLifespan + GestationT} \quad (1)$$

$$LoglogAge = -\log(-\log(RelativeAge))\ (2)$$

By definition, $RelativeAge$ is between 0 to 1 and $LoglogAge$ is positively correlated with age. The incorporation of gestation time is not essential. We simply include it to ensure that RelativeAge takes on positive values. We used the double logarithmic transformation to link relative age to the covariates (cytosines) for the following reasons. First, the transformation maps the unit interval to the real line. Second, this transformation ascribes more influence to exceptionally high and low age values (**Extended Data Fig.1a-c**). Third, this transformation is widely used in the context of survival analysis. Fourth, this non-linear transformation worked better than the identity transformation.

## Transformation based on log-linear age for clock 3

Our measure of log-linear age leverages age at sexual maturity (ASM). The transformation has the following properties: it takes the logarithmic form when the chronological age is young and takes the linear form otherwise. It is continuously differentiable at the change.

First, we define a ratio of the age relative to ASM, termed as $RelativeAdultAge$, as the following:

$$RelativeAdultAge = \frac{Age+GestationT}{ASM+GestationT}\ (3),$$

where the addition of *GestationT* ensures that the $RelativeAdultAge$ is always positive.

#
#Rcode

```
#
#
#Universal mammalian clock2 of age
#
#author for developing clock2: Ake T. Lu
#author for developing clock1 and clock3: Zhe Fei
#
rm(list=ls())
options(stringsAsFactors = F)
setwd('')
#
myinput.list=readRDS('mydata_GitHub.Rds')
# The following labels the coefficient values of the three universal clocks
beta.name=c('beta_clock1','beta_clock2','beta_clock3')
y.name=c('Y.pred1','Y.pred2','Y.pred3')
age.name=c('DNAmAgePanMammalianClock1','DNAmAgePanMammalianClock2','DNAmAgePanMammali
anClock3')

#clock2
F2_antitrans_clock2<-function(y,y.maxAge,y.gestation,const=1){
  x0=const*exp(-exp(-1*y))
  x1=x0*(y.maxAge+y.gestation)
  x=x1-y.gestation
  x
}
#
#clock3
#
F1_logli <- function(age1, m1, m2 = m1, c1=1){
  ifelse(age1 >= m1, (age1-m1)/m2 , c1*log((age1-m1)/m2/c1 +1) )
}
#RelativeAdultAge
F2_revtrsf_clock3 <- function(y.pred, m1, m2 = m1, c1=1){
  ifelse(y.pred<0, (exp(y.pred/c1)-1)*m2*c1 + m1, y.pred*m2+m1 )
}

#
# The “loglifn” function shows how to calculate m1 for the transformation
# It is the “a_Logli” in the function

F3_loglifn = function(dat1,b1=1,max_tage = 4,
                      c1=5, c2 = 0.38, c0=0){
n=nrow(dat1)

  age1 =
(dat1$maxAge+dat1$GestationTimeInYears)/(dat1$averagedMaturity.yrs+dat1$GestationTimeInYears)

  a1 = age1/(1+max_tage)
```

```
  dat1$a1_Logli = a1 #x/m1 in manuscript

  a2 = (dat1$GestationTimeInYears + c0)/(dat1$averagedMaturity.yrs)
  dat1$a_Logli = a_Logli = c1*a2^c2
  #m=5*(G/ASM)^0.38 from regression analysis/formula(7)


  x = dat1$Age + dat1$GestationTimeInYears
  t2 = dat1$averagedMaturity.yrs*b1 + dat1$GestationTimeInYears
  x2 = x/t2 #### log(x/t2)
  y = F1_logli(x2, a_Logli, a_Logli)
#
#(2)merge info and metharray beta values
#
glmnet.list=myinput.list[[4]]#The three universal clock prediction models
mycpgs=c(glmnet.list[[1]]$var,glmnet.list[[2]]$var,glmnet.list[[3]]$var)
mycpgs=unique(mycpgs)
mycpgs=mycpgs[mycpgs!='Intercept']
#dat.meth0: number of Mammalian array CpGs (n=37554) x [number of samples+1]
#
dat.meth0=myinput.list[[2]]
dat.meth0=subset(dat.meth0,CGid%in%mycpgs)#only keep the CpGs in the three clocks
dat.meth=t(dat.meth0[,-c(1)])
colnames(dat.meth)=dat.meth0$CGid
dat.meth=data.frame(Basename=colnames(dat.meth0)[-c(1)],dat.meth)
dat.meth$Intercept=1
#
info=merge(by='Basename',info,dat.meth)
#
#(3) predict RelativeAge
#

for(k in 1:3){
glmnet=glmnet.list[[k]]
glmnet$beta=glmnet[,beta.name[k]]
#glmnet$var[1]=ifelse(glmnet$var[1]=="(Intercept)",'Intercept',glmnet$var[1])
temp=as.matrix(subset(info,select=as.character(glmnet$var)))
info[,y.name[k]]=as.numeric(as.matrix(subset(info,select=as.character(glmnet$var)))%*%glmnet$beta)
}
#
#(4) predict clocks
#
#(4.1) Clock 1
info[,age.name[1]]=exp(info[,y.name[k]])-2
#(4.2) Clock 2
info$DNAmRelativeAge=exp(-exp(-1*info[,y.name[2]]))
info[,age.name[2]]=
F2_antitrans_clock2(info[,y.name[2]],info$HighmaxAge,info$GestationTimeInYears,const=1)
```

```
#(4.3) Clock 3
info=F3_loglifn(info)#to compute m estimate for tuning point in the log-linear transformation
info$m1=info$a_Logli
info$DNAmRelativeAdultAge=F2_revtrsf_clock3(info[,y.name[3]], info$m1)
info[,age.name[3]]<-
  info$DNAmRelativeAdultAge *(info$averagedMaturity.yrs + info$GestationTimeInYears) -
info$GestationTimeInYears


  dat1$LogliAge <- y
  return(dat1)
}
#
#(1)generate variable HighmaxAge
#
names(myinput.list)
info=myinput.list[[1]]#The only required variables are SpeciesLatinName and Basename
anage=myinput.list[[3]]
info=merge(by='SpeciesLatinName',info,
subset(anage,select=c(SpeciesLatinName,GestationTimeInYears,averagedMaturity.yrs,maxAge)))
head(info)
#Description for mymax=1.3
#We were concerned that the uneven evidence surrounding the maximum age of different species
#could bias our analysis. While billions of people have been evaluated for estimating
#the maximum age of humans (122.5 years) or mice (4 years),
#the same cannot be said for any other species.
#To address this concern, we made the following assumption:
#the true maximum age is 30% higher than that reported in AnAge
#for all species except for humans and mice (Mus musculus).
#Therefore, we multiplied the reported maximum lifespan of non-human or non-mouse species by #1.3.
#Our predictive models turn out to be highly robust with respect to this assumption.
MYMAX=1.3
info$HighmaxAge=MYMAX*info$maxAge
info$HighmaxAge[info$SpeciesLatinName=='Homo
sapiens']=info$maxAge[info$SpeciesLatinName=='Homo sapiens']
info$HighmaxAge[info$SpeciesLatinName=='Mus
musculus']=info$maxAge[info$SpeciesLatinName=='Mus musculus']
#
#
#
output=subset(info,select=c('Basename','SpeciesLatinName','MammalNumberHorvath','Age','Tissue','DNA
mRelativeAge','DNAmRelativeAdultAge',age.name))
#
x=output$Age
y=output$DNAmAgePanMammalianClock2
cor0=round(cor(x,y),2)
mae0=round(median(abs(x-y)),2)
plot(x,y,xlab='Age',ylab='Clock2: DNAm Age',
```

```
      main=paste0('Bottlenose dolphin (n=',length(x),')\n','cor=',cor0,
               ', MAE=',mae0),
      col='white',xlim=c(0,max(max(x),max(y))+1),
      ylim=c(0,max(max(x),max(y))+1))
text(x,y,label=output$MammalNumberHorvath,col='blue')
```

**(2)Two-stage meta EWAS of age across species**

The following R code is a function to perform two-stage meta EWAS of age. The function requires two input files: all.info lists SampleID, SpeciesLatinName, Age, Tissue and xs.all lists beta values of CpGs with rownames=SampleID.

```
#
#R code
#

rm(list=ls())
library(doParallel)
library(WGCNA)
library(iterators)
F_twostep_stouffer_verbose<-function(all.info,xs.all,sp.cut=15,Y.test='Age'){
  out.sp_tissue=data.frame(table(all.info$SpeciesLatinName,all.info$Tissue))
  names(out.sp_tissue)=c('SpeciesLatinName','Tissue','SpeciesTissue.freq')
  out.sp_tissue=subset(out.sp_tissue,SpeciesTissue.freq>=sp.cut)
  summary(out.sp_tissue)
  cat('dim of sp_tissue',nrow(out.sp_tissue),'\n')
  out.sp_tissue$SpeciesLatinName=as.character(out.sp_tissue$SpeciesLatinName)
  out.sp_tissue$Tissue=as.character(out.sp_tissue$Tissue)
  sp.ntissue=data.frame(table(out.sp_tissue$SpeciesLatinName))
  names(sp.ntissue)=c('SpeciesLatinName','sp.ntissue')
  sp.ntissue2=subset(sp.ntissue,sp.ntissue>1)
  sp.ntissue2=subset(sp.ntissue,sp.ntissue==1)
  #
  out.sp_tissue=out.sp_tissue[order(out.sp_tissue$SpeciesLatinName,out.sp_tissue$SpeciesTissue.freq),]
  out.sp_tissue$SpeciesTissue=paste0(out.sp_tissue$SpeciesLatinName,'_',out.sp_tissue$Tissue)
  out.sp_tissue$sp_tissue.order=1:dim(out.sp_tissue)[1]
  #
  out.sp_tissue.first=subset(out.sp_tissue,!duplicated(SpeciesLatinName))
  out.sp_tissue.first$n1=out.sp_tissue.first$sp_tissue.order
  #
  out.sp_tissue=out.sp_tissue[order(out.sp_tissue$SpeciesLatinName,-out.sp_tissue$SpeciesTissue.freq),]
  out.sp_tissue.last=subset(out.sp_tissue,!duplicated(SpeciesLatinName))
  out.sp_tissue.last$n2=out.sp_tissue.last$sp_tissue.order
  sp_tissue.df=merge(by='SpeciesLatinName',subset(out.sp_tissue.first,select=c(SpeciesLatinName,n1)),
                   subset(out.sp_tissue.last,select=c(SpeciesLatinName,n2)))
  #
  sp_tissue.df$ntissue=sp_tissue.df$n2-sp_tissue.df$n1+1
  out.sp_tissue=out.sp_tissue[order(out.sp_tissue$sp_tissue.order),]
  #
  N.test<-length(out.sp_tissue$SpeciesTissue)
  #
  all.info$Y.test=all.info[,Y.test]
  print(head(all.info$SampleID))
  print(head(rownames(xs.all)))
  ck=sum(as.numeric(head(all.info$SampleID)!=head(rownames(xs.all))))
  if(ck>0) {stop ('SampleID not match')}
```

```
  #
  corr.df=foreach(k=1:N.test,
                  .combine='cbind',
                  .packages=c('doParallel','WGCNA')) %dopar%{
                    tissue.test=out.sp_tissue$SpeciesTissue[k]
                    id.test=which(all.info$SP_Tissue==tissue.test)

ewas.out=standardScreeningNumericTrait(datExpr=xs.all[id.test,],all.info$Y.test[id.test],areaUnderROC=FA
LSE)
                    ewas.out$SE=(1-ewas.out$cor^2)/sqrt((ewas.out$nPresentSamples-2))
                    ewas.out$SE.alt=ewas.out$cor/ewas.out$Z
                    ewas.out$W=1/(ewas.out$SE)^2
                    ewas.out$w0=1
                    ewas.out$sp_tissue=tissue.test

ewas.out$Z.new=ifelse(ewas.out$pvalueStudent>0,abs(qnorm(ewas.out$pvalueStudent/2))*sign(ewas.out
$Z),ewas.out$Z)
                    ewas.out=subset(ewas.out,select=c(cor,Z.new))
                    names(ewas.out)=c(paste0('cor.',tissue.test),paste0('Z.',tissue.test))
                    return(ewas.out)
                  }
  #
  corr.df.z   =data.frame(CpG=names(xs.all),corr.df[,substr(names(corr.df),1,2)=='Z.'])
  corr.df.cor =data.frame(CpG=names(xs.all),corr.df[,substr(names(corr.df),1,2)!='Z.'])
  #
  N.test=nrow(sp_tissue.df)
  for(k in 1:N.test){
    species.test=gsub(sp_tissue.df$SpeciesLatinName[k],pattern=' ',rep='.')
    zs=grep(names(corr.df.z),pattern=species.test)
    z.df=subset(corr.df.z,select=c(zs))
    if(length(zs)==1){
      sp.df=z.df
    }else{
      meta1=apply(z.df,1,sum,na.rm=T)
      meta2=sqrt(sum(ncol(z.df)))
      sp.df=data.frame(meta1/meta2)
      #
    }
    names(sp.df)=as.character(species.test)
    if(k==1) {meta.df=sp.df
    }else{
      meta.df=cbind(meta.df,sp.df)}
  }
  #
  #stage 2
  #
  meta1.all=apply(meta.df,1,sum,na.rm=T)
  meta2.all=sqrt(sum(ncol(meta.df)))
```

```
  metaZ=data.frame(CpG=corr.df.z$CpG,Meta.Z=meta1.all/meta2.all)
  metaZ$Meta.P0=pnorm(-abs(metaZ$Meta.Z))*2
  p1=pnorm(-abs(metaZ$Meta.Z),log=T)
  p2=p1/log(10)
  a=10^(p2-floor(p2))*2
  b=floor(p2)
  b1=ifelse(a>10,b+1, b)
  a1=ifelse(a>10,a/10,a)
  metaZ$Meta.P=as.character(paste0(round(a1,digits=2),'E',b1))
  metaZ$log10P=-1*(log10(a1)+b1)
  out.list=vector(3,mode='list')
  out.list[[1]]=metaZ
  out.list[[2]]=data.frame(CpG=corr.df.z$CpG,meta.df)
  names(out.list[[2]])[-c(1)]=paste0(names(out.list[[2]])[-c(1)],'.Z')
  #
  F1_direction<-function(x){
    direction={}
    for(i in 1:length(x)){
      direction=paste0(direction,ifelse(x[i]>0,'+','-'))
    }
    return(direction)
  }
  out.list[[2]]$Direction=apply(meta.df,1,F1_direction)
  out.list[[3]]=out.sp_tissue
  return(out.list)
}
```

### (3) GREAT analysis

The following R code is a function to send requests to GREAT web server. The input bed file lists 4 variables: chromosome, bp of CpG start (CGstart in hg19), bp of CpG end (CGstart+1), and CpG name.

```
#
#R code
#
library(rGREAT)
output.all={}
job = submitGreatJob(input, bg = background,
                     species              = "hg19",
                     includeCuratedRegDoms = TRUE,
                     rule                 = c("basalPlusExt"),
                     adv_upstream          = 5.0,
                     adv_downstream        = 1.0,
                     adv_span              = 50,
                     request_interval = 300,
                     version="3",
                     max_tries = 10)
#
ontology.all=availableOntologies(job)
```

```
print(ontology.all)

for(k in 1:length(ontology.all)){
  print(ontology.all[k])
  out0.list = tryCatch(getEnrichmentTables(job,ontology=ontology.all[k], download_by =
"tsv"),error=function(e){NULL})
  if(!is.null(out0.list)){
    db0.list=as.list(names(out0.list))
    output<-Map(cbind,Database=db0.list,out0.list)
    output<-do.call('rbind',output)
    output.all=rbind(output.all,output)
  }
}
```

**(4) Universal chromatin state analysis**

The following R code computes the summary statistics for the annotation of our top 1000 positively and negatively age-related CpGs based on universal chromatin state analysis. The code requires two input datasets: (1) hmm lists CpG and its corresponding chromatin state, and (2) summary statistics of our EWAS of age.

```
#
#R code
#
TopNum=1000
out.all={}

  ewas=subset(ewas,CpG%in%hmm$CpG)
  ewas=ewas[order(ewas$rank.Meta.Z),]
  ewas.pos=subset(ewas,Meta.Z>0)
  ewas.pos=ewas.pos[1:TopNum,]
  ewas.neg=subset(ewas,Meta.Z<0)
  ewas.neg=ewas.neg[1:TopNum,]
  #
  ck=is.element(ewas$CpG,hmm$CpG)
  #
  ewas.list=vector(len=2,mode='list')
  names(ewas.list)=c('pos','neg')
  ewas.list[[1]]=ewas.pos
  ewas.list[[2]]=ewas.neg
  for(ilist in 1:2){
    group=names(ewas.list)[[ilist]]
    #
    ewas.test=ewas.list[[ilist]]
    for(i in 1:n.states){
      anno=cpg.list[[i]]
      x.pos.cpg=intersect(anno$CpG,ewas.test$CpG);x.pos=length(x.pos.cpg)
      m=length(anno$CpG)#white balls
      k=TopNum#number of draw
      ntot=nrow(ewas)
      n=ntot-m
```

```
    #odds ratio
    a=x.pos
    b=k-a
    c=m-a
    d=n-b
    odds.pos=(a*d)/(c*b)
    #obser x or > x
    p.enrich.pos=ifelse(odds.pos>=1,phyper(x.pos-1,m=m,n=n,k=k,lower.tail=F)
                   ,phyper(x.pos,m=m,n=n,k=k,lower.tail=T))
    #
    logp.enrich.pos=ifelse(odds.pos>=1,phyper(x.pos-1,m=m,n=n,k=k,lower.tail=F,log.p=T)
                      ,phyper(x.pos,m=m,n=n,k=k,lower.tail=T,log.p=T))
    logp.enrich.pos=logp.enrich.pos
    #
    sign.pos=ifelse(odds.pos>=1,1,-1)
    log10p.enrich.pos= -(logp.enrich.pos/log(10))*sign.pos
    #

out1=data.frame(tissue=TISSUE[k.tissue],state=anno$state[1],group=group,TopNum=TopNum,N.overlap
= x.pos,
                OddsRatio=odds.pos,Hyper.P=p.enrich.pos,log10P=log10p.enrich.pos)
    out.all=rbind(out.all,out1)
    rm(a,b,c,d,x.pos,odds.pos)
    #
  }#end state
 }#end ilist
```

### (5) EWAS-GWAS overlap

The following R code is a function to compute the summary statistics for our EWAS-GWAS overlap analaysis based on hypergeometric tests. The parameters in the function are: ORDER is a dummy variable, aar0 is data frame of EWAS summary statistics that lists the variables as described in head(aars), other0 lists the gene P value generated by MAGENTA, and HGNC gene symbol from a test GWAS, other.name0 is the label of the test GWAS, cutoff is 2.5% in our study, n.topcpg0=1000 (the top number of CpGs) in our study, and background.hg is the background based on the genomic regions in our custom Mammalian array, as described in head(background.hg).

```
#
#R code
#
> head(background.hg)
  HGNC_gene CHR        bp       CGid GeneID
1      A1BG  19  58864846 cg22568540      1
2      A1CF  10  52619664 cg02811585  29974
3      A1CF  10  52573791 cg25496747  29974
4      AACS  12 125578355 cg02591564  65985
6      AAK1   2  69811375 cg05656329  22848
7      AATF  17  35336293 cg04920416  26574
> head(aars)
```

```
            CGid    Meta.Z P HGNC_gene CHR         bp GeneID
17511 cg18141557 58.21023 0    POU3F3   2 105472226   5455
8958  cg09227056 55.57959 0      EVX2   2 176940449 344191
12408 cg12879445 55.28574 0     COX8C  14  93897410 341947
12409 cg12879445 55.28574 0  KIAA1409  14  93897410  57578
15207 cg15682828 55.10546 0      ZIC2  13 100635161   7546
5426  cg05551621 54.09696 0     FOXD3   1  63789233  27022
F_enrich<- function(ORDER,aar0,other0,other.name0,cutoff,n.topcpg0,background.hg=background.hg19){
  aar0=subset(aar0,!is.na(aar0$Meta.Z))
  other0=subset(other0,!is.na(other0$p.other))
  other0.gene=other0

other0=merge(by='HGNC_gene',other0,subset(background.hg,select=c(HGNC_gene,CHR,bp,CGid,HGNC_
gene.amin)))

  other0$genomic_region=paste(as.character(other0$HGNC_gene),as.character(other0$CGid),sep='-')
  #length(unique(other0$GeneID))
  #
  #
  background=other0
  #very important
  ntot=nrow(background)
  #
  aar0=aar0[order(-abs(aar0$Meta.Z)),]
  aar0$genomic_region=paste(as.character(aar0$HGNC_gene),as.character(aar0$CGid),sep='-')
  aar.top=vector(length=2,mode='list')
  aar.top[[1]]=subset(aar0,Meta.Z>0)
  aar.top[[2]]=subset(aar0,Meta.Z<0)
  aar.top[[1]]=aar.top[[1]][1:n.topcpg0,]
  aar.top[[2]]=aar.top[[2]][1:n.topcpg0,]
  names(aar.top)=c('pos','neg')
  #
  other0=other0[order(other0$p.other),]
  other0.gene=other0.gene[order(other0.gene$p.other),]
  #
  output={}

    n2=round(cutoff*dim(other0.gene)[1])#top cutoff genes
    other.gene=other0.gene[1:n2,]
    #
    other=subset(other0,HGNC_gene%in%other.gene$HGNC_gene)
    #
    for(t in 1:2){
      index=is.element(aar.top[[t]]$genomic_region,other$genomic_region)
      x=sum(as.numeric(index))
      m=dim(other)[1]# number white balls/other gwas
      n=ntot-m
      k=dim(aar.top[[t]])[1] # numer of draw
```

```
    #
    p.enrich=    phyper(x-1,m=m,n=n,k=k,lower.tail=F)
    logp.enrich  =phyper(x-1,m=m,n=n,k=k,lower.tail=F,log.p=T)
    log10p.enrich= -(logp.enrich/log(10))
    a1=10^(ceiling(log10p.enrich)-log10p.enrich)
    Hyper.P.scientic=ifelse(p.enrich>=0.1,
                     round(p.enrich,digits=2),
                     paste0(round(a1,1),'e-',ceiling(log10p.enrich)))
    #
    #
    OverlapGenes=paste(unique(aar.top[[t]]$HGNC_gene[index]),collapse =';')
    OverlapGenes.CpG=paste(aar.top[[t]]$CGid[index],collapse =';')
    #
    n.overlapgene=length(unique(aar.top[[t]]$HGNC_gene[index]))
    n.annotation=length(unique((other$HGNC_gene)))
    #
    output0=data.frame(index=ORDER,class=names(aar.top)[t],cutoff=cutoff,GWAS=other.name0,
                n.overlapgene=n.overlapgene,
                n.annotation=n.annotation,
                n.overlapGR=x,
                n.annotationGR=m,
                Overlap=paste(x,m,sep="/"),
                P=p.enrich,log10P=log10p.enrich,P.scientic=Hyper.P.scientic,
                OverlapGenes=OverlapGenes,OverlapGenes.CpG=OverlapGenes.CpG)
    output=rbind(output,output0)
   }


  return(output)
}
```

**(6) Array Converter algorithm**

The following R code is a function to convert Illumina 450k array beta values to Mammalian array beta values. The first parameter lists a vector of Mammalian CpGs to be imputed and the second parameter (InputEPIC) is a data frame that lists elastic net prediction models for converting Human 450K Array to our Mammalian array. A demonstration R code with an example data can be found in https://github.com/shorvath/MammalianMethylationConsortium/tree/main/UniversalPanMammalianClock/R_code/ArrayConverterAlgorithm.

```
#library(vioplot)
#library(glmnet)
#library(WGCNA)
library(foreach)
library(doParallel)
library(tidyverse)
#
ArrayConverter_EPICtoMM <- function(MammalianList = NULL,
```

```
                                    InputEPIC, mval = TRUE,
                                    transf = function(a){a},
                                    invfn = function(a){a}){
  nsamp = ncol(InputEPIC)

  if(mval) {
    transf = mvalue
    invfn = inv_m

  }
  ImpMat = NULL
  for (cgname in MammalianList){
    tmp1 = match(cgname, names(fitall))
    coefmat = fitall[[tmp1]]
    idx1 = match(as.character(coefmat$EPIC_CG[-1]),
                 rownames(InputEPIC))
    if(sum(is.na(idx1)) >0 ) {
      cat(cgname,"Not all EPIC probes for imputation are available.")
      cg_pred = rep(NA, nsamp)
    }
    else {
      xmat = as.matrix(InputEPIC[idx1,])
      if(ncol(xmat)==1){xmat=t(xmat)}
      #print(cgname)
      #print(dim(xmat))
      cg_pred = coefmat$coef[1] + t(xmat)%*%coefmat$coef[-1]
      if(mval) cg_pred = invfn(cg_pred)
    }
    ImpMat = cbind(ImpMat, cg_pred)
  }
  colnames(ImpMat) = MammalianList
  rownames(ImpMat) = colnames(InputEPIC)

  return(ImpMat)
}

mvalue = Vectorize (function(a){
  log(a/(1-a), 2)
}, vectorize.args = c("a"))

inv_m = Vectorize (function(a){
  2^a/(2^a + 1)
}, vectorize.args = c("a"))


corscreen = function(cor1, pick=10000, thr = 1e-3){
  corsort = sort(cor1)
  lower = corsort[pick]
```

```
  upper = rev(corsort)[pick]
  zeros = which(abs(cor1) < thr)
  while (length(zeros)< pick) {
    thr = 2*thr
    zeros = which(abs(cor1) < thr)
  }
  zeros = sample(zeros, pick)

  idxpick = sort(unique(c(which(cor1<= lower), which(cor1>= upper), zeros)))
  return(idxpick)
}


ImpMM_v1 <- function(cgname, idx1, train.idx, test.idx,
                     width = 1e6, geneRegion = FALSE,
                     doplot=FALSE){

  cg1 = as.numeric(normalized_betas_sesame[rownames(normalized_betas_sesame) == cgname,idx1])

  chr = as.character(mm_annot$seqnames[mm_annot$CGid == cgname])

  if(geneRegion){
    geneStart = mm_annot$geneStart[mm_annot$CGid == cgname]
    geneEnd = mm_annot$geneEnd[mm_annot$CGid == cgname]

    cgs_epic = epic_annot$CGid[epic_annot$seqnames == chr &
                               epic_annot$start >= geneStart &
                               epic_annot$start <= geneEnd]

  }else {
    CGstart = mm_annot$CGstart[mm_annot$CGid == cgname]
    cgs_epic = epic_annot$CGid[epic_annot$seqnames == chr &
                               epic_annot$start >= CGstart - width &
                               epic_annot$start <= CGstart + width]

  }

  cgs_epic = intersect(cgs_epic, rownames(betas_epic))
  npred = length(cgs_epic)
  if(npred<2) return(c(npred, rep(median(cg1[train.idx]),length(idx1))))

  epic_mat = betas_epic[cgs_epic, idx1]
  # verboseScatterplot(cg1, betas_epic[cgname, idx1])

  m1 = cv.glmnet(t(epic_mat[,train.idx]), cg1[train.idx], alpha=.5)
  # plot(m1)
  # coef(m1,s="lambda.min")
```

```
  cg_fitted = predict(m1, newx = t(epic_mat), s="lambda.min" )

  if(doplot){
    # verboseScatterplot(cg1, cg_fitted)
    verboseScatterplot(cg1[test.idx], cg_fitted[test.idx])

  }
  return(c(npred,cg_fitted))
}


ImpMM_v4 <- function(cgname, idx1, train.idx, test.idx,
                     width = 1e6, geneRegion = FALSE, use30k = FALSE,
                     transf = function(a){a}, invfn = function(a){a},
                     doplot=FALSE){

  cg1 = as.numeric(normalized_betas_sesame[rownames(normalized_betas_sesame) == cgname,idx1])
  cg1 = transf(cg1)

  chr = as.character(mm_annot$seqnames[mm_annot$CGid == cgname])

  if(geneRegion){
    geneStart = mm_annot$geneStart[mm_annot$CGid == cgname]
    geneEnd = mm_annot$geneEnd[mm_annot$CGid == cgname]

    cgs_epic = epic_annot$CGid[epic_annot$seqnames == chr &
                               epic_annot$start >= geneStart &
                               epic_annot$start <= geneEnd]

  }else if (use30k) {
    cor1 = cor(cg1[train.idx], t(betas_epic[, idx1[train.idx]]))
    idxpick = corscreen(cor1)
    cgs_epic = rownames(betas_epic)[idxpick]

  } else {
    CGstart = mm_annot$CGstart[mm_annot$CGid == cgname]
    cgs_epic = epic_annot$CGid[epic_annot$seqnames == chr &
                               epic_annot$start >= CGstart - width &
                               epic_annot$start <= CGstart + width]

  }

  cgs_epic = intersect(cgs_epic, rownames(betas_epic))
  npred = length(cgs_epic)
  if(npred<2) {
    y = invfn(median(cg1[train.idx]))
    return(c(npred, rep(y,length(idx1)) ))
  }
```

```r
  epic_mat = betas_epic[cgs_epic, idx1]
  # verboseScatterplot(cg1, betas_epic[cgname, idx1])

  m1 = cv.glmnet(t(epic_mat[,train.idx]), cg1[train.idx], alpha=.5)
  # plot(m1)
  # coef(m1,s="lambda.min")

  cg_fitted = predict(m1, newx = t(epic_mat), s="lambda.min" )

  if(doplot){
    # verboseScatterplot(cg1, cg_fitted)
    verboseScatterplot(cg1[test.idx], cg_fitted[test.idx])

  }

  y = invfn(cg_fitted)
  return(c(npred,y))
}

fitMM_v1 = function(cgname, idx1, train.idx, test.idx=NULL,
                    width = 6e7, uselambda = "lambda.min",
                    # transf = function(a){a}, invfn = function(a){a},
                    doplot=FALSE, returnCoef = TRUE){
  mvalue = Vectorize (function(a){
    log(a/(1-a), 2)
  }, vectorize.args = c("a"))

  inv_m = Vectorize (function(a){
    2^a/(2^a + 1)
  }, vectorize.args = c("a"))
  transf = mvalue
  invfn = inv_m

  cg1 = as.numeric(normalized_betas_sesame[rownames(normalized_betas_sesame) == cgname,idx1])
  cg1 = transf(cg1)

  chr = as.character(mm_annot$seqnames[mm_annot$CGid == cgname])

  {
    CGstart = mm_annot$CGstart[mm_annot$CGid == cgname]
    cgs_epic = epic_annot$CGid[epic_annot$seqnames == chr &
                                epic_annot$start >= CGstart - width &
                                epic_annot$start <= CGstart + width]

  }

  cgs_epic = intersect(cgs_epic, rownames(betas_epic))
```

```
  npred = length(cgs_epic)
  if(npred<2) {
    cat("Not enough EPIC CGs for imputation, try longer bandwidth.")
    return(c(npred ))
  }

  epic_mat = t(betas_epic[cgs_epic, idx1])
  m1 = cv.glmnet(epic_mat[train.idx,], cg1[train.idx], alpha=.5)
  if(doplot){
    plot(m1)
  }
  coef1 = coef(m1, s=uselambda)
  coefs = data.frame(EPIC_CG = rownames(coef1)[coef1[,1]!=0],
             coef = coef1[coef1!=0])
  cg_fitted = predict(m1, newx = epic_mat, s=uselambda )
  y = invfn(cg_fitted)

  if(returnCoef) return(coefs)
  else return(y)
}

imputeMM_v1 = function(cglist,
                       doparallel = FALSE){
  }
```


1. Horvath, S., Lu, A.T., Li, C.Z. , Haghani, A., Arneson, A.,Ernest, J. & Mammalian, M.C. [https://zenodo.org/account/settings/github/repository/shorvath/MammalianMethylationConsortium#](https://zenodo.org/account/settings/github/repository/shorvath/MammalianMethylationConsortium#). (2022).
